# Evolution of endogenous retroviruses in the Suidae: evidence for different viral subpopulations in African and Eurasian host species

**DOI:** 10.1186/1471-2148-11-139

**Published:** 2011-05-24

**Authors:** Fabrícia F Nascimento, Jaime Gongora, Michael Charleston, Michael Tristem, Stewart Lowden, Chris Moran

**Affiliations:** 1 Faculty of Veterinary Science, The University of Sydney, NSW 2006, Australia; 2 School of Information Technologies and Centre for Mathematical Biology, The University of Sydney, NSW 2006, Australia; 3 Division of Biology, Imperial College London, Silwood Park, Ascot, Berkshire SL5 7PY, UK; 4 Veterinary Health Research Pty Ltd. Armidale, NSW 2350, Australia; 5 Instituto de Microbiologia Paulo de Góes, Universidade Federal do Rio de Janeiro, Avenida Carlos Chagas Filho, 373, Rio de Janeiro 21941-902, Brazil

## Abstract

**Background:**

Porcine endogenous retroviruses (PERVs) represent remnants of an exogenous form that have become integrated in the domestic pig (*Sus scrofa*) genome. Although they are usually inactive, the capacity of γ1 ERVs to infect human cells *in vitro *has raised concerns about xenotransplantation because the viruses could cross the species barrier to humans. Here we have analyzed the evolution of γ1 ERVs in ten species of Suidae (suids, pigs and hogs) from Eurasia and Africa using DNA sequences for their coding domains (*gag*, *pro*/*pol *and *env *genes). For comparison with γ1 PERVs, we have also analysed γ2 ERVs which in domestic pigs are known to be inactive and do not pose a risk to xenotransplantation.

**Results:**

Phylogenetic analysis using Bayesian inference showed that γ1 and γ2 ERVs have distinctive evolutionary histories. Firstly, two different viral lineages of γ1 ERVs were found and a coevolutionary analysis demonstrated that they correspond broadly to their host phylogeny, one of Eurasian and another of African species, and show no evidence of horizontal transmission. γ2 ERVs, however, show a bush-like evolution, suggesting a rapid viral radiation from a single common ancestor with no correspondence between host and viral evolutionary trees. Furthermore, though γ1 ERV *env *genes do not possess frequent stop codons, γ2 *env *genes do. To understand whether γ1 suid ERVs may be still replicating, we have also evaluated their likely mechanism of proliferation by statistically testing internal to terminal branches using nonsynonymous versus synonymous substitution ratios. Our results suggest that γ1 ERVs are increasing in copy number by reinfection, which requires the translocation of the virus from one cell to another.

**Conclusions:**

Evidence of at least two viral subpopulations was observed in γ1 ERVs from Eurasian and African host species. These results should be taken into account in xenotransplantation since γ1 ERVs appear to be codiverging with their host and maintaining ongoing capacity to infect somatic and germ cells.

## Background

Endogenous retroviruses (ERVs) are the remnants of an exogenous viral form that infected and became integrated in germ cell genomes [1]. ERVs have been found in a variety of vertebrates, including reptiles, amphibian, birds and mammals [2]. However, the occurrence and evolution of ERVs in different organisms are still not well understood and they are sometimes lumped with "junk" DNA of unknown function [3,4].

In the domestic pig and wild boar (*Sus scrofa*) genome, ERVs are referred to as PERVs (porcine endogenous retroviruses) and belong to the β and γ genera comprising several viral groups [5,6]. The γ1 genus is divided in PERV-A, B and C classes on the basis of *env *sequences, while the γ2 genus comprises only PERV-E. Although most PERVs carry mutations that render them functionally inactive, some γ1 PERVs retain the ability to express and can infect human cells *in vitro *[7,8]. The capacity of γ1 ERVs to infect human cells in tissue culture raised serious concerns on the safety of xenotransplantations [9,10] because pigs are the first choice of xenograft donors [11-13]; consequently γ1 ERVs have been extensively studied in *Sus scrofa*.

In contrast, few DNA sequences from γ2 ERVs have been reported to date [5,14,15], although a full length provirus has been described and named PERV-E because of its similarity to the human HERV-E. Furthermore, PERV-E forms a distinct clade with viruses from the HERV-E family and does not cluster with γ1 PERVs [14]. Genetic and expression analyses also have not indicated an obvious infectious risk from γ2 retroviruses for xenotransplantation [15].

At present, little is known about either γ1 or γ2 ERVs in other suid species than *Sus scrofa *[5,16]. Patience et al. [5] screened by PCR for the presence of ERVs (both γ and β genera) in five different suid species, while Niebert and Tönjes [16] sequenced γ1 DNA fragments in seven different suid species. However, they did not perform any evolutionary analyses of ERVs [16]. Clearly both of these studies have left many questions unanswered.

The mechanisms by which γ1 ERVs increase their copy number in suid genomes are also poorly understood and three main mechanisms have been reported for human ERVs (HERVs) [17,18]; retrotransposition in *cis*, complementation in *trans*, and reinfection [19]. The first mechanism involves replication by co-packaging mRNAs in virus-like particles built up from viral proteins encoded by their own *gag *and *pol *genes. As retrotransposition in *cis *does not result in cell to cell translocation, the *env *gene is not required to be functional. Conversely, complementation in *trans *involves replication of two retroviral elements of the same family, with one defective provirus using the functional protein produced by another provirus. Finally, ERVs might form infectious exogenous particles and reinfect either a germ cell or a somatic cell. Because reinfection requires the translocation of viruses from one cell to another, the presence of functional genes is required, although this may also result from complementation in *trans *[17,20]. New ERVs will likely acquire new mutations during reinfection or retrotransposition due to their error prone reverse transcriptase in virus replication [19]. Thus proviruses might occasionally give rise to infectious exogenous forms which can reinfect the animal itself or animals of the same species and sometimes cross to other species, and subsequently integrate in a germ cell [21].

Recently a study of the host phylogeny [22,23] clarified the relationship between Eurasian and African suids based on nuclear and mitochondrial DNA sequences, enabling the study of ERV evolution in these suid species and the evolutionary congruence of host and viral lineages. Here, we analyzed the γ1 ERV coding domains (*gag*, *pro*/*pol *and *env*) sampled from 10 different suid host species from Eurasia and Africa. We have analyzed their evolutionary relationships as well as their likely mechanism of replication. We have also compared the evolution of γ1 and γ2 ERVs using their *env *genes and implication for xenotransplantation.

## Results

### Screening of ERV sequences

A total of 144 partial γ1 ERV sequences comprising the *pol*, *gag *and *env *(classes A, B and C) genes and 40 γ2 *env *were generated (Table 1). One individual for each suid species was analyzed, except for the babirusa, where two animals were analyzed for *env *class E. No γ1 ERV genes were amplified in the babirusa, while *pol *and *env *class A and C were not amplified in the forest hog, and *pol *and *env *class A were not amplified in the Javan warty pig and the desert warthog, respectively. We were able to amplify γ2 ERVs in all of the species.

**Table 1:** List of species of Suidae showing their origin and number of sequences generated.

Origin	Species and subspecies	Common name	Source	Tissue	*gag*	*pol*	***env *A**^**+**^	*env *B	***env *C**^**+**^	*env *E
Africa	*Hylochoerus meinertzhageni*	Forest hog	Uganda	Muscle	3	-	1	3	-	3
	
	*Phacochoerus aethiopicus*	Desert warthog	Kenya	Muscle	3	4	-	3	-	3
	
	*Phacochoerus africanus*	Common warthog	Iwaba Zimbabwe	Muscle	7	7	6	1	-	4
	
	*Potamochoerus larvatus*	Bush-pig	Zimbabwe	Blood	3	4	3	3	-	2
	
	*Potamochoerus porcus*	Red river hog	Duisburg Zoo, Germany	Muscle	4	4	4	3	-	3

Eurasia	*Sus scrofa*	Wild boar	Yorkshire Farm, UK	Blood	4	4	2	4	3	2

Asia Pacific	*Sus barbatus oi*	Western bearded pig	Singapore Zoo, Singapore	Blood	4	4	-	3	4	3
	
	*Sus barbatus barbatus*	Bornean bearded pig	Singapore Zoo, Singapore	Blood	4	3	-	4	6	2
	
	*Sus celebensis*	Sulawesi warty pig	Sulawesi, Indonesia	Muscle	3	3	2	4	3	3
	
	*Sus verrucosus*	Javan warty pig	Poznan Zoo, Poland	Muscle	3	-	-	5	6	4
	
	*Babyrousa babyrussa*	Babirusa	Surabaya Zoo, Indonesia	Blood	-	-	-	-	-	11*
	
**TOTAL**					**38**	**33**	**18**	**33**	**22**	**40**

+ After reorganization of sequences (see text)

* Two animals were analyzed.

Blastn confirmed that amplicons corresponded to ERVs. Primers targeting *env *classes A and C cross amplified. Sequences generated with these primers were initially analyzed together and then separated into A and C according to blastn results. After reorganization, a total of 18 and 22 sequences for class A and C *env *were analyzed. *env *class C was not amplified from any African specimens, obviating the need for the non-parametric test for panmixia for this gene.

Three *gag *and one *env *class E sequences showed indels longer than 50 bp and were removed from alignments to increase accuracy of evolutionary analysis. This allowed a higher number of base pairs to be analyzed because these indels were observed in different positions. After removing these sequences, a total of 141 novel γ1 sequences were analyzed along with 28 sequences from the draft pig genome and 110 sequences from GenBank (Additional file 1). These sequences showed a pairwise genetic distances in expected number of substitutions per site ranging from 0 - 0.10 for *pol*, 0 - 0.11 for *gag*, 0 - 0.17 for *env *class A, 0 - 0.27 for *env *class B, and 0 - 0.15 for *env *class C. Identical sequences were rarely observed, and they mainly reflect identical sequences from GenBank and between Genbank and *Sus scrofa *genome sequences. Non-parametric tests revealed highly significant differentiation between ERVs from African versus Eurasian species (*P *< 0.001). On the other hand, no sequence was removed from γ2 *env *dataset for which pairwise genetic distances ranged from 0.001 - 0.11. The non-parametric test of population subdivision suggested that Eurasian, African and babirusa sequences came from different subpopulations (*P *< 0.02).

In addition, most DNA sequences of γ1 ERV genes lacked stop codons or, when present, were limited to one stop codon per sequence. This contrasted with γ2 *env*, which possessed several stop codons (up to 5 per sequence) and the presence of several indels, some of which were in unique positions and others at conserved positions across Eurasian, African and babirusa sequences, suggesting that they are unlikely to be replicating as *env *is necessary for viral reinfection. Possibly, they might retrotranspose in *cis *if other coding domains were devoid of stop codons, although this is apparently unlikely because *Sus scrofa gag *and *pro*/*pol *also showed stop codons and frame shift mutations. Interestingly, there were three γ2 sequences from African species (two from common warthog and one from desert warthog) and two from babirusa that did not show stop codons.

### Test for detecting recombination

**Figure 1:**
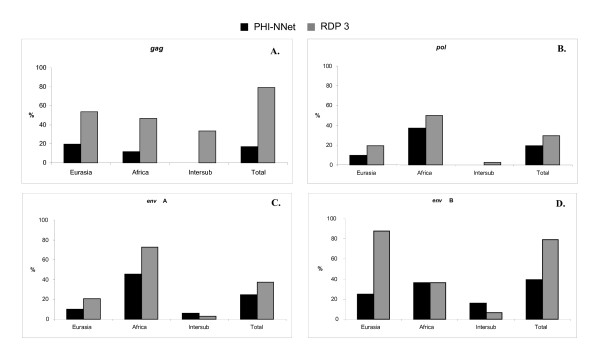
**Frequency of recombinants identified by PHI-NNet algorithm and the RDP 3 software for (A) *gag*, (B) *pol*, (C) *env *class A, (D) *env *class B genes**. Intersub = Frequency of inter subpopulation (Eurasian-African) recombinants after removal of recombinants identified in the separate Eurasian and African datasets.

From 30% to 79% of sequences were identified as recombinants for γ1 genes by the RDP 3 software [24] including some apparent inter-group recombinants (Figure 1). This program also identifies recombination breakpoints, which for our dataset were located at different positions when the aligned viral sequences are compared. Because of that, the intervening non-recombinant fragments of sequence were not useful for phylogenetic reconstructions because more than 50% of sequence length had to be removed from the alignment. Furthermore, in most cases, sequences identified as non-recombinant were not representative of the different suid species available in our dataset. We therefore did not use the non-recombinants identified by RDP 3 for phylogenetic reconstructions.

From 17% to 40% of sequences were identified as recombinants for γ1 genes by the PHI-NNet algorithm [25], and a higher frequency was mainly observed in sequences from African suids but less frequently than with RDP 3 (Figure 1). Apparent inter-group recombinants between Eurasian and African sequences were identified only for *env*, albeit with a low frequency (Figures 1C and 1D). It is interesting to note that approximately 50% of *env *class A sequences in African suids appeared to be recombinants (Figure 1C) while all *env *class C sequences, found only in Eurasian pigs, were likely to be recombinants and thus could not be used for evolutionary analyses. Because this algorithm identified a smaller number of recombinants, we used the observed non-recombinant dataset in our phylogenetic reconstructions.

**Figure 2:**
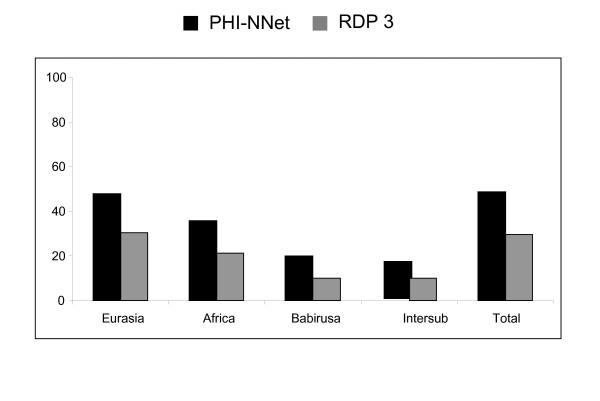
**Frequency of recombinants identified by PHI-NNet algorithm and the RDP 3 software for *env *class E gene**. Intersub = Frequency of inter subpopulation (Eurasian-African-Babirusa) recombinants after removal of recombinants identified in the separate Eurasian, African and Babirusa datasets.

Because approximately 50% of recombinants were observed for γ2 *env*, with even some apparent inter-group recombinants detected by the PHI-NNet algorithm (Figure 2), the non-parametric test was re-run using only the non-recombinant dataset. In this case, the null hypothesis of panmixia could not be rejected (*P *= 0.07), providing no conclusive evidence that γ2 ERVs from Eurasian and African species and babirusa represent different retroviral subpopulations.

### Phylogeny reconstructions

**Figure 3:**
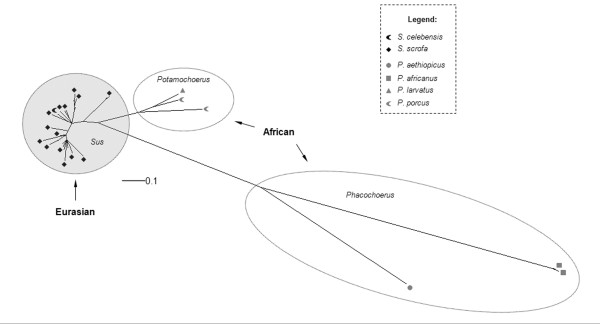
**Unrooted Bayesian inference for *env *class A gene**. For clarity, posterior probability values are not represented, but all main groups showed a value of 0.99 to 1.00. Black symbols represent ERVs from the different Eurasian species and gray symbols represent ERVs from different African species (see legend for detail in coding).

**Figure 4:**
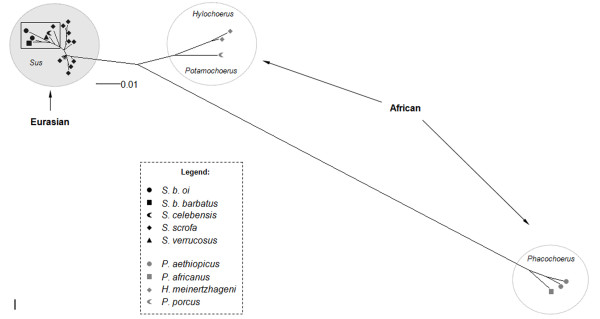
**Unrooted Bayesian inference for *env *class B gene**. For clarity, posterior probability values are not represented, but all main groups showed a value of 0.99 to 1.00. Black symbols represent ERVs from the different Eurasian species and gray symbols represent ERVs from different African species (see legend for detail in coding).

**Figure 5:**
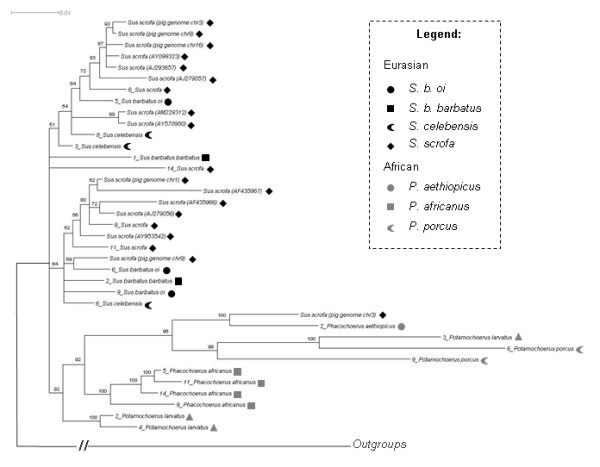
**Rooted Bayesian inference for *pol *gene**. Numbers close to branches indicate posterior probability values above 0.50. Numbers close to names correspond to different clones. Black symbols represent ERVs from the different Eurasian species and gray symbols represent ERVs from different African species (see legend for detail in coding). Outgroup branch is not to scale.

In the Suidae, γ1 ERVs are grouped into two different lineages, one in Eurasian and another in African suids, as indicated by class A and B *env *phylogenies (Figures 3 and 4). Furthermore, the *pol *phylogeny revealed five groups, four comprising only sequences from Eurasian species, and another mainly with African species in a well defined clade (Figure 5). However, one γ1 *pol *sequence present in the draft pig genome grouped with γ1 *pol *sequences of African suids. This sequence was identified as a recombinant by the RDP 3 software but not by the PHI-NNet algorithm.

The clades shown for African class A and B *env *phylogenies (Figures 3 and 4) were generally coincident with African host clades, except for the internal arrangement of *Potamochoerus porcus *and *Hylochoerus meinertzhageni *in the *env *class B phylogeny (Figure 4), which differed from the host tree [22,23] where *Hylochoerus *grouped with *Phacochoerus *instead of *Potamochoerus*. On the other hand, Eurasian γ1 ERVs showed a less conserved arrangement in all phylogenies, with internal groups not corresponding to the host phylogeny [22,23] except for the *env *class B internal arrangement, which was very similar in the host phylogeny (highlighted in Figure 4).

**Figure 6:**
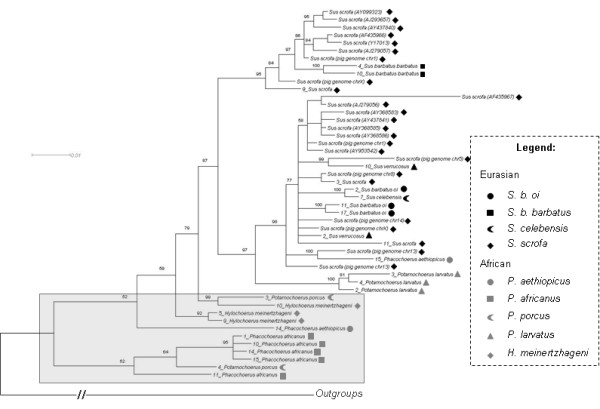
**Rooted Bayesian inference for *gag *gene**. Numbers close to branches indicate posterior probability values above 0.50. Numbers close to names correspond to different clones. Black symbols represent ERVs from the different Eurasian species and gray symbols represent ERVs from different African species (see legend for detail in coding). Outgroup branch is not to scale.

The *gag *phylogeny did not group all African ERVs in a single clade, although most of them were still different from Eurasian ones (highlighted in Figure 6). A similar pattern is also observed when recombinant sequences detected by RDP 3 are also removed from phylogenetic analysis (data not shown). This suggests that recombination is not responsible for the lack of resolution on *gag*. Further conclusions based solely on *gag *should be carefully evaluated. Furthermore, these sequences did not show substitution saturation, with I_ss _much smaller than the critical I_ss _value [26,27].

**Figure 7:**
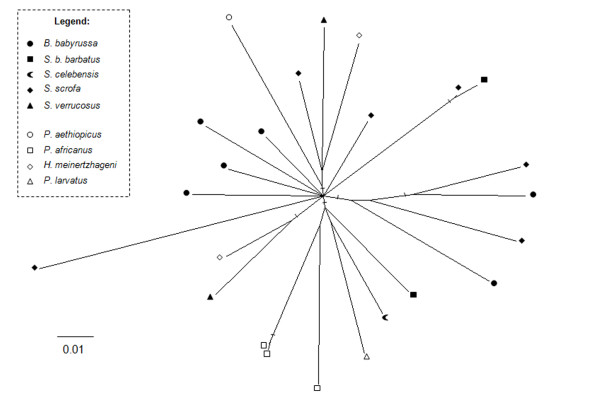
**Unrooted Bayesian inference for *env *class E gene**. Marked branches represent posterior probability values from 0.90-1.00 and posterior probability values for other branches ranged from 0.50-0.70 (not shown for clarity). Black symbols represent ERVs from the different Eurasian species and gray symbols represent ERVs from different African species (see legend for detail in coding).

Conversely, Bayesian analysis for γ2 suid ERVs showed a bush-like pattern (Figure 7) with short internal and long external branches. The same pattern was observed using neighbor-joining, and also with the complete sequence dataset (including recombinants), suggesting that for γ2 ERVs, recombinants were not responsible for the star-like phylogeny. An unresolved phylogeny may also result from data saturation, although γ2 *env *showed very low saturation, with I_ss _= 0.16, below the critical I_ss _value (I_ss.c _= 0.77) [26,27]. Similar results were obtained for first, second and third codon positions. Furthermore, the likelihood-mapping method indicated that the data contain a high amount of net-like (9.1%) and star-like (16.3%) signals, indicating that the tree was not well resolved according to the criteria of Strimmer and von Haeseler [28].

### Mechanism of ERV proliferation

Most sequences of γ1 ERVs did not possess stop codons and/or indels. Furthermore, when present, they were usually located at unique positions in any given sequence. Low frequency of stop codons is evidence that complementation in *trans *has been a rare event [18].

Estimates of ω for each branch in the phylogeny were used for testing the possibility of purifying selection [29,30]. Comparisons of internal branches with external branches ("two-ratio" model) suggested that purifying selection was acting on all genes because of the highly significant differences in log-likelihoods (Table 2). Furthermore, ω was significantly smaller for internal branches than for external branches: 0.34 and 0.78 for *gag*, 0.25 and 0.56 for *pol*, 0.30 and 1.04 for *env *class A, and 0.22 and 1.47 for *env *class B (Table 2).

**Table 2:** *d*_*N*_/*d*_*S *_(ω) ratios and likelihood ratio tests (LRT) for ERV genes in the family Suidae to test whether ω for internal branches are significantly less than 1.

	Two-ratio model				
**Gene**	**Internal** **branches (ω)**	**Terminal** **branches (ω)**	**ℓ**_**1**_	**ℓ**_**0**_	**LRT**^**1**^

*gag*	0.34	0.78	-3235.42	-3253.27	38**

*pol*	0.25	0.56	-5373.26	-5405.94	65**

*env *(class A)	0.30	1.04	-4271.15	-4306.51	70**

*env *(class B)	0.22	1.47	-2470.67	-2515.98	90**

ℓ = log_e_-likelihood

^1 ^LRT = Likelihood ratio test, twice the difference of the log likelihoods[2Δℓ = 2(ℓ_1_-ℓ_0_)]

** Extremely significant (*P *< 0.01; *χ*_1_^2^)

For the γ2 *env *gene, we found statistical support for the "one-ratio" model (*P *= 0.053), which made testing the "two-ratio" model unnecessary.

## Discussion

We found strong evidence that γ1 ERVs from Eurasian and African species based on the *pol *and *env *phylogenies belong to two different lineages. This pattern was also observed for LTR phylogenies sampled from Eurasian and African host species [31]. Host species from Eurasia are more similar to one another, showing shorter branch lengths compared to the more differentiated African host species with longer branch lengths, consistent with traditional taxonomy which puts all Eurasian species in the genus *Sus*, whereas there are several genera recognized in Africa [32-34]. A similar pattern was generally observed in γ1 ERV class A and B *env *phylogenies and for the LTRs, suggesting that proviruses might be codiverging with their respective host species.

The resolution of the *pol *phylogeny into distinct Eurasian and African lineages, which was not found with *gag*, is not a consequence of analysis of longer alignments. Bayesian analysis based on 885 bp of *pol *sequence (the same size as the *gag *alignment) showed an almost identical topology to that observed in Figure 5, showing a single African clade of ERVs (including the one ERV sequence of the draft pig genome). Similarly, when *ca*. 400 bp of *env *is analysed, an almost identical topology of that of Figure 3 is also observed [30].

The *gag *phylogeny is different from the other viral gene phylogenies and to the host phylogeny [22,23]. This may be explained by the gene tree versus species tree problem in which gene phylogenies sometimes conflict with a species tree because individual gene sequences can generate different topologies and consequently conflicting results [35-37], but is also likely to be due simply to the lack of resolution in the *gag *phylogeny which cannot be attributed to the length of base pairs analysed compared to the other genes. The lack of resolution of this gene was also observed by other authors when analysing other retroviruses [for an example see [38,39]]. Alternatively, the DNA sequence conservation of *gag *gene suggests that Gag proteins are important for viral replication. Experimental analysis of Moloney murine leukemia virus showed that mutations of several portions of *gag *were incompatible with viral replication [40], implying strong selection for conservation of the *gag *gene sequence. In this case, while other viral domains might have evolved distinct African and Eurasian sequences, *gag *might have been constrained from doing so.

Class A and B *env *phylogenies showed two likely subpopulations in African suids, one in *Phacochoerus *species, and another in *Potamochoerus *and *Hylochoerus *species more related to ERVs from *Sus *species, (Figures 3 and 4). Amplification of orthologous sequences as well as full length proviral sequences would provide a better basis for comparison, but for reasons of practicality and convenience paralogous sequences amplified by PCR have been routinely used to determine the evolutionary relationship of ERVs in different host species [41-43].

Recombination was apparent in several γ1 and γ2 sequences, presumably as a result of co-packaging of different RNAs in the same viral particle. In *Sus scrofa*, recombination between highly variable *env *sequences is easier to detect than for *gag *and *pol*. Moreover, selection would be also more likely to be operating on *env *than on the other genes because the viral envelope must evolve to escape immune detection, thus making recombination a likely source of adaptative phenotypes. ERVs from African species showed more evidence of recombination than ERVs from Eurasian species, although this apparent difference may be an artifact of the higher differentiation (longer branch lengths) for *pol *and for class A and B *env *in African species. Furthermore, the RDP 3 software identified a higher number of recombinants than the PHI-NNet algorithm for γ1 genes, a fact also observed in HIV by Lamers et al. (2009) and attributed to the molecular models implemented in some RDP 3 methods, to the number of sequences in each analysis and to variation within each subpopulation altering the number of identified recombinants [44]. This might be why the γ1 *pol *sequence from the draft pig genome was identified as recombinant by RDP 3 and non-recombinant by the PHI-NNet algorithm. These discordances obviously result from the different methods of analyses, pointing to the need of improving their consistency and power especially in detecting recombinants between ERV sequences. This improvement is also important to better characterize the inter subpopulation recombinants observed in this study and confirm that they represent genuine recombinant sequences.

We also found evidence for past purifying selection on all γ1 genes. This suggested that retroviruses that remained functional were initially selected and that transposition by reinfection was relevant, while loss of function would have likely resulted in neutral evolution within the host genome ω ≈ 1. Furthermore, the rarity of stop codons and/or indels also suggested that complementation in *trans *and retrotransposition in *cis *were not relevant mechanisms of transposition [17,18].

### Evolutionary comparisons of γ1 and γ2 *env *genes

In contrast to the γ1 phylogeny, γ2 ERV *env *gene showed a bush-like unresolved tree, (Figure 7) confirmed by the likelihood-mapping, either resulting from inadequate sampling or data (soft polytomy) or reflecting the actual evolutionary history of γ2 ERVs in the Suidae (hard polytomy) [45-47]. The bush-like pattern might reflect an incomplete sampling because *env *from ten other suid species is missing in the phylogeny. However, the γ1 ERV *env *phylogenies (Figures 3 and 4) using fewer species showed a better resolved tree with species from Eurasia grouping apart from African suids, suggesting that the number of viral sequences samples from each suid host would not be an explanation for the poor resolution of γ2 ERV *env *phylogeny.

An unresolved phylogeny, like that observed for γ2 ERVs, may also result from data saturation, but the *env *sequences showed very low saturation. Although we cannot rule out the possibility that the γ2 star-like phylogeny resulted from inadequate sampling, this seems unlikely as samples used here were available from ERVs from three different suid lineages observed by Nascimento [22,23]. In this case, a hard polytomy seems more plausible. Although Vandamme [45] emphasised the difficulty of proving the existence of hard polytomies in real life, Poe and Chubb [48], Barth et al. [46], Willerslev et al. [49] and several others [47,50] have observed some apparent examples in different taxa, including birds, mammals, plants and protozoa, and this pattern was also observed for human ERVs [19].

This hard polytomy may then represent a rapid radiation from a single common ancestor giving rise to multiple distinct retroviral lineages almost at the same time [19,45]. These ERVs have subsequently become inactivated resulting in the loss of the last active lineage (also suggested by the presence of several stop codons), following a long period of inactivity leading to the star shaped phylogeny [19].

The reason why distinct evolutionary histories are observed for γ1 and γ2 ERVs is not known. It would be reasonable to attribute it to the evolution of the host defensive mechanisms. However, Katzourakis et al. [19] by modelling ERV evolutionary dynamics demonstrated that such a star shaped phylogeny can be generated by a "null model" in which all parameters are set constant through out time.

## Conclusions

The different lineages of γ1 ERVs in the Suidae are generally congruent with the host phylogeny. This implies that horizontal transmission across these very different host lineages has not occurred. Moreover, the frequent evolutionary occurrence of viral recombination may be relevant to risk analysis of xenotransplantation, especially since viral recombination will favour adaptation and confer capacity for maintaining ongoing infection of somatic and germ cells. This highlights the importance of improving methods to prevent PERVs from crossing the species barriers. Finally, the evolution of γ2 ERVs confirms their lack of risk in xenotransplantation because the star phylogeny suggests loss of the last active element which is also confirmed by the presence of numerous stop codons.

## Methods

### Sample collection, primer design and PCR assays

DNA samples of 12 animals from 10 species and one subspecies of Suidae (Table 1) were analyzed for the presence of ERVs. Oligonucleotide primers targeting conserved regions of the γ1 *gag*, *pro*/*pol *and *env *(classes A, B and C) genes and γ2 *env *(class E; Table 3) were designed using DNA sequences from *Sus scrofa *as input for OLIGO (version 6.8; Molecular Biology Insights). These specific primers were designed aiming to amplify from the other Suidae species similar sequences to *Sus scrofa *and increase the chances to amplify sequences in the same genomic loci. Products were PCR amplified with an initial denaturation at 94°C for 30 sec followed by 35 cycles of denaturation at 94°C for 30 sec, annealing at 55°C or 58°C for 60 sec (for *gag *and other genes respectively), extension at 72°C for 90 sec, and a final extension of 72°C for 5 min. Amplicons were electrophoresed in a 1.5% agarose gel and fragments of expected size (Table 3) were purified following gel band excision using UltraClean™ Gel Spin DNA Purification kit (Mo Bio, Australia).

**Table 3:** Oligonucleotide primer used to amplify and to sequence partial ERV genes.

Primer name	Primer sequences (5' → 3')	Expected size (bp)
gag	CTGTTGTTGAAGCGAAAG	1,080
	TACCTTCAGCCGTGTTG	

pol	ACCCGCTAACCAAAGA	1,578
	TGTCTGACCCGATTACC	

env-A	CCCGAACTCCCATAAACC	1,720
	AAGGCCCAACTGTAAGTAACA*	

env-B	CTGCGGCCTGACATAAC	1,060
	AAGGCCCAACTGTAAGTAACA*	

env-C	GAACCTGGTGGCCTGATCTAT	1,524
	GGCCCAACTGTGAGTAACA	

env-E	ACCTCTTTGCCTGACAATACA	1,320
	TACAAGGCAGGGAACAAGTAG	

pol-intF^1^	CAGACATACCGCTGACTG	n/a

pol-intR^1^	TGTACTGTCATCCGGGTTCTG	n/a

env-A-intF^1^	TGGTATGTCTTGGGGAAT	n/a

* The same reverse primer was used to amplify partial fragments of both *env *classes A and B. Forward primers were specific for each type of *env *gene.

^1. ^Internal primers used only for sequencing.

n/a = not applicable

### Cloning and sequencing

Purified PCR products were cloned using the TOPO TA cloning^® ^kit (Invitrogen, Australia) and DNA from plasmids containing inserts was subsequently extracted with UltraClean™ Mini Plasmid Prep kit (Mo Bio). For each specimen, approximately five purified plasmids were sent to the Australian Genome Research Facility Ltd (AGRF; Brisbane, Australia) for DNA sequencing with primers listed in Table 3. Electropherograms were checked using BIOEDIT (version 7.0.9.0) [51].

### Data mining

DNA sequences were blasted using the blastn option (http://blast.ncbi.nlm.nih.gov/Blast.cgi) to confirm that ERV products had been amplified and to identify all previously reported PERV sequences. The pre-ensembl *Sus scrofa *genome database (http://pre.ensembl.org/index.html; version Sscrofa8) was searched using the blat option for *gag*, *pro*/*pol *and *env*, with best matching sequences being also included in alignments. Only sequences with 100% coverage were used in subsequent analyses. GenBank sequences were from genomic PERV sequences from pigs of various breeds, porcine cell lines, infected human primary cells and transcripts from virus particles released from porcine cell lines. The majority of GenBank sequences do not contain information on genomic position and we could not take this information into account. Sequences from the *Sus scrofa *genome were also not representative of the numerous PERV loci already described. This was because the Sscrofa8 version of the pig genome was not finished and because we have narrowed our search to sequences with 100% coverage.

### Alignment and genetic distance estimations

All novel and available PERV sequences from GenBank and from the draft pig genome were aligned with MUSCLE (version 3.6) [52], with default options and were manually checked. Columns showing gaps in the majority of aligned sequences and nucleotides at the minority of sequences were manually removed. Conversely, columns showing gaps at sites where nucleotides were present in the majority of sequences were not removed, and these gaps were treated as missing data [53]. *pol *and *gag *sequences from murine leukemia virus (AY277737 and EU075329 respectively), gibbon leukemia virus (U60065 and M26927 respectively) and koala retrovirus (AF151794) were used as outgroups. We initially aligned amino acid sequences of outgroups and suid ERVs, and subsequently back-translated to nucleotide using SEAVIEW (version 4.0) [54]. We did not include outgroups when analyzing the *env *genes because of the very low conservation of ERV *env *sequences. Pairwise genetic distances were estimated using the modified Log-Det implemented in MEGA (version 4) [55] in view that it can estimate reliable distances for closely related taxa [56]. Identical sequences, very similar (pairwise genetic distances equal to 0.001) and those with large indels were removed from alignments for a better phylogeny estimation, recombination detection and calculation of ω (see below).

### Test of subpopulation structure and detection of recombination

To test whether viral sequences isolated from Eurasian, African and the babirusa (when available) suids belong to statistically different subpopulations, we explored the datasets with a test for population sub-division developed by Hudson et al. [57] and adapted to study HIV populations by Achaz et al. [58]. This non-parametric test of population subdivision was performed using a web-based interface (http://wwwabi.snv.jussieu.fr/achaz/hudsontest.html) with a nominal significance threshold of 0.05 for accepting or rejecting a null hypothesis for structure. Because recombinants may misplace sequences in a phylogeny [59,60], they were detected using the PHI-NNet algorithm [25]. This could efficiently detect recombination among closely related sequences [25] from Eurasian host species, African host species and the babirusa (when available) and could even suggest Eurasian-African (-babirusa) recombinants. Recombinants were also independently detected using the software RDP 3[24] which implements seven different recombination programs, 1) the original RDP method [61], 2) the GENECONV method [62,63], 3) the MaxChi method [64], 4) the Chimaera method [65], 5) the SiScan method [66] and 7) 3SEQ method [67]. Default settings and the option "auto mask for optimal recombination detection" were used in all analyses.

### Phylogenetic reconstruction

Bayesian inference (BI) using the Markov chain Monte Carlo method was used for phylogenetic analyses of γ1 *gag*, *pol *and *env *and γ2 *env *genes using MR BAYES (version 3.1.2) [68,69]. DNA substitution models were selected by MODELGENERATOR (version 0.84) [70] using the Akaike information criterion (AIC). For a list of DNA substitution models see Table 4. Two and three separate BIs were carried out for γ1 and γ2 genes respectively and compared using Bayes factors (B_10_) as described in Nylander et al. [71]. For each BI two chains were run for a different number of generations (Table 4) and one tree per 100 generations was collected. Convergence and mixing were evaluated using TRACER (version 1.4.1) [72] and the initial 25% of runs was discarded (burn-in). A majority-rule consensus phylogram was constructed according to results of Bayes factor comparisons.

**Table 4:** Bayes factor comparisons between model M_0 _and M_1 _for each ERV gene showing also the number of generations for each Bayesian inference.

Gene	**Model comparison**^1^		Number of generations		2*log_**e **_**B**_**10**_^§^
	**M**_**0**_	**M**_**1**_	**M**_**0**_	**M**_**1**_	
*pol*	*1-HKY+Γ	^+^3-HKY+Γ	3,000,000	6,000,000	0.049

*gag*	*1-K80+Γ	^+^3-K80+Γ	8,000,000	13,000,000	0.033

*env *(class A)	*1-HKY+Γ	^+^3-HKY+Γ	3,000,000	10,000,000	-0.061

*env *(class B)	*1-K80	^+^3-K80	3,000,000	3,000,000	0.016

*env *(class E)	*1-HKY+Γ	*1-GTR+Γ	3,000,000	3,000,000	0.0025
	
	*1-HKY+Γ	^+^3-HKY+Γ	3,000,000	3,000,000	0.0014

^1. ^HKY = Hasegawa-Kishino-Yano model [78]; K80 = Kimura 2-parameter [79]; Γ = gamma distribution.

* 1 = one model for the whole gene.

^+ ^3 = partition of the data per codon position.

^§ ^Values represent twice the log_e _of the Bayes factor (B_10_) in the comparison of models M_0 _and M_1 _(2*log_e _B_10_). Positive values below 2 indicate no evidence against M_0 _[71,80]

### Test of substitution saturation

Because the γ1 *gag *and the γ2 *env *sequences showed unresolved phylogenies, we have calculated an index of DNA substitution saturation (I_ss_) [73] using the program DAMBE (version 5.0.85) [74] to evaluate whether sequences contained phylogenetic signals. Furthermore, because of the star-like γ2 *env *phylogeny, a likelihood-mapping analysis [28] was performed using TREE-PUZZLE[75] which shows the percentage of tree-like, star-like and net-like signals in the dataset [for more information, see [76]].

### Estimations of ω ratios

To evaluate whether γ1 ERVs may be still replicating [18] we have calculated the nonsynonymous (*d*_*N*_) versus synonymous (*d*_*S*_) substitution ratios (ω = *d*_*N*_/*d*_*S*_) for γ1 genes using PAML (version 4.2) [77] according to Belshaw et al. [18]. Internal branches were compared to terminal branches using the "two-ratio" model [30] where the null hypothesis of ω ≥ 1 (neutral or positive selection) was compared with the alternative hypothesis of ω < 1 (purifying selection). Significance was tested by fixing ω at 1 for internal branches and performing a likelihood ratio test (LRT), where twice the difference of the log-likelihoods was compared to critical values of the χ^2 ^distribution for 1 degree of freedom [30].

For γ2 *env *gene, we have compared a single ω for the entire tree (the "one-ratio" model) to a "free-ratio" model (a separate ω ratio for each branch of the phylogeny) [18]. The significance of differences between "one-ratio" and "free-ratio" models was assessed by a LRT for 34 degrees of freedom [30].

Outgroups were removed from the trees before performing these calculations.

### GenBank accession numbers

*env *class E: [GQ906159-GQ906198]; *env *class A: [GQ906199-GQ906216]; *env *class B: [GQ906217-GQ906249]; *env *class C: [GQ906250-GQ906271]; *gag*: [GQ906272-GQ906309]; *pol*: [GQ906310-GQ906342] for sequences generated in this study. For sequences retrieved from GenBank see additional file 1.

Multiple sequence alignments are provided as additional files 2, 3, 4, 5, 6 and 7.

## Authors' contributions

FFN generated data, carried out molecular, evolutionary and statistical analyses and drafted the manuscript. JG participated in the design of the study and provided assistance with interpretation of results. MC provided special input in phylogenetic and co-phylogenetic analyses. MT provided special input into the analysis and interpretation of retroviral data. SL collected and provided genomic DNA samples from most suid host species. CM initiated and oversaw project, provided assistance with interpretation of results and redrafting of manuscript. All authors read and approved the final manuscript.

## Supplementary Material

Additional file 1 **GenBank accession numbers for sequences included in this study**. List of GenBank sequences used in phylogenetic analyses Click here for file

Additional file 2 ***gag *alignment**. *gag *alignment of sequences generated in this study, sequences from GenBank and the draft pig genome Click here for file

Additional file 3 ***pol *alignment**. *pol *alignment of sequences generated in this study, sequences from GenBank and the draft pig genome Click here for file

Additional file 4 ***env *A alignment**. *env *A alignment of sequences generated in this study, sequences from GenBank and the draft pig genome Click here for file

Additional file 5 ***env *B alignment**. *env *B alignment of sequences generated in this study, sequences from GenBank and the draft pig genome Click here for file

Additional file 6 ***env *C alignment**. *env *C alignment of sequences generated in this study, sequences from GenBank and the draft pig genome Click here for file

Additional file 7 ***env *E alignment**. *env *E alignment of sequences generated in this study, sequences from GenBank and the draft pig genome Click here for file
