# Supplementary material for: Evolution of endogenous retroviruses in the Suidae: evidence for different viral subpopulations in African and Eurasian host species
Source: BMC Evol Biol. 2011 May 24;11:139. doi: 10.1186/1471-2148-11-139 (PMC3128044; doi:10.1186/1471-2148-11-139)
Supplement: Additional file 1 — GenBank accession numbers for sequences included in this study. List of GenBank sequences used in phylogenetic analyses [file 1471-2148-11-139-S1.DOC]

Additional file 1 GenBank accession numbers for sequences included in this study

| **Gene** | **N1*** | **N2*** | **GenBank accession numbers** |
| --- | --- | --- | --- |
| *gag* | 13 | 28 | [AF147808], [AF435966], [AF435967], [AJ133816-AJ133818], [AJ279056], [AJ279057], [AJ293656], [AJ293657], [AM229311], [AM229312], [AY056035], [AY099323], [AY099324], [AY368583-AY368586], [AY437840], [AY437841], [AY570980], [AY953542], [DQ996272], [EF133960], [EU523109], [EU789636], [Y17013] |
| *pol* | 8 | 21 | [AF435966], [AF435967], [AJ133816-AJ133818], [AJ279056], [AJ279057], [AJ293656], [AJ293657], [AM229311-AM229313], [AY056035], [AY099323], [AY099324], [AY570980], [AY953542], [DQ996273], [EF133960], [EU523109], [Y17013] |
| *env*  (class A) | 3 | 30 | [EU086221], [AF296168], [AF417222-AF417226], [AF435967], [AJ279056], [AJ288585], [AJ293656], [AY288779], [AY312521], [AY312523], [AY312524], [AY312526], [AY368580-AY368582], [AY368587-AY368589], [AY371067], [EF133960], [EU086219], [EU086220], [EU086222], [EU086224], [EU789636], [Y12238] |
| *env*  (class B) | 3 | 21 | [AJ133816], [AJ133818], [AJ279057], [AJ293657], [AY056024], [AY056026-AY056029], [AY056035], [AY099324], [AY312517], [AY312518], [AY312522], [AY312528-AY312530], [AY312532], [EU523109], [Y12239], [Y17013] |
| *env*  (class C) | 0 | 9 | [AF402660-AF402663], [AF417229], [AM229312], [AM229313], [DQ996276], [EU090250] |
| *env*  (class E) | 8 | 1 | [AF356698] |

* N1 and N2 = number of sequences retrieved from the draft pig genome (version Sscrofa8) and from the GenBank respectively.
