# Supplementary material for: Evolution of endogenous retroviruses in the Suidae: evidence for different viral subpopulations in African and Eurasian host species
Source: BMC Evol Biol. 2011 May 24;11:139. doi: 10.1186/1471-2148-11-139 (PMC3128044; doi:10.1186/1471-2148-11-139)
Supplement: Additional file 2 — gag alignment. gag alignment of sequences generated in this study, sequences from GenBank and the draft pig genome [file 1471-2148-11-139-S2.PDF]

gag alignment (including all sequences)

The first number next to each name represents the clone number.

```
                                     10        20        30        40        50        60        70
                             ....|....|....|....|....|....|....|....|....|....|....|....|....|....|
Seq1 Sscrofa8 chromosome1    ATGGGACAGACGGTGACGA-------CCCCTCTTAGTTTGACTCTCGACCA-TTGGACTGAAGTTAAATC
Seq2 Sscrofa8 chromosome1    ...........A.......-------....C....................-..............G...
Seq3 Sscrofa8 chromosome3    ...........A.......-------....C....................-..............G...
Seq4 Sscrofa8 chromosome5    ...................-------.........................-..................
Seq5 Sscrofa8 chromosome7    ...................-------.........................-..................
Seq6 Sscrofa8 chromosome8    ................T..-------...................T.....-..................
Seq7 Sscrofa8 chromosome13   ...................-------....C....................-..................
Seq8 Sscrofa8 chromosome13   ...................-------....C....................-..................
Seq9 Sscrofa8 chromosome13   ...................-------.........................-..................
Seq10 Sscrofa8 chromosome14  ...................-------.........................-..................
Seq11 Sscrofa8 chromosome16  ...........A.......-------....C....................-..............G...
Seq12 Sscrofa8 chromosomeX   ..........T........-------....................A....-..................
Seq13 Sscrofa8 chromosomeX   ...........A.......-------....C....................-..............G...
Sus scrofa(AF435967)         ...................-------.........................-..................
Sus scrofa(EF133960)         ...................-------....C....................-...........C......
Sus scrofa(AY437840)         ...........A.....T.-------....C....................-..............G...
Sus scrofa(AJ279057)         ...........A.......-------....C....................-..............G...
Sus scrofa(Y17013)           ...........A.......-------....C........A...........-..............G...
Sus scrofa(AY056035)         ...........A.......-------....C....................-..............G...
Sus scrofa(AF147808)         ...........A.......-------....C....................-..............G...
Sus scrofa(EU523109)         ...........A.......-------....C....................-..............G...
Sus scrofa(AF435966)         ...........A.......-------....C........A...........-..............G...
Sus scrofa(AJ133816)         ...........A.......-------....C........A...........-..............G...
Sus scrofa(AJ133818)         ...........A.......-------....C....................-..............G...
Sus scrofa(AJ293657)         ...........A.....T.-------....C....................-..............G...
Sus scrofa(AY099323)         ...........A.....T.-------....C....................-..............G...
Sus scrofa(AJ133817)         ...........A.....T.-------....C....................-..............G...
Sus scrofa(AY099324)         ...........A.....T.-------....C....................-..............G...
Sus scrofa(AM229312)         ...................-------.........................-..................
Sus scrofa(AM229311)         ...................-------.........................-..................
Sus scrofa(DQ996272)         ...................-------.........................-..................
Sus scrofa(AY570980)         ...................-------.........................-..................
Sus scrofa(AY368583)         ...........A.....T.-------.........................-..................
Sus scrofa(AY953542)         ...................-------.........................-..................
Sus scrofa(AY437841)         ...........A.....T.-------.........................-..................
Sus scrofa(AY368585)         ...........A.....T.-------.........................-..................
Sus scrofa(AY368584)         ...........A.....T.-------.........................-..................
Sus scrofa(AY368586)         ...........A.....T.-------.........................-..................
Sus scrofa(AJ279056)         ...................-------.........................-..................
Sus scrofa(EU789636)         ...................-------.........................-..................
```

```
Sus scrofa(AJ293656)            ...................-------.........................-..................
3 Sus scrofa                    ................T..-------...................T.....-..................
6 Sus scrofa                    ...A......T.....T.--------...................T.....-..................
9 Sus scrofa                    ...................-------....C....................-..................
11 Sus scrofa                   ...................-------.........................-..................
3 Sus barbatus barbatus         ...................CCCCCCC....C....................T..................
4 Sus barbatus barbatus         ...........A.......-------....C..............T.....-..............G...
9 Sus barbatus barbatus         ...................------C....C....................-..................
10 Sus barbatus barbatus        ...................-------....C..............T.....-..................
2 Sus barbatus oi               ...................-------....................A....-..................
4 Sus barbatus oi               ...................-------.........................-..................
11 Sus barbatus oi              ...................-------.........................-..................
17 Sus barbatus oi              ...................-------.........................-..................
2 Sus verrucosus                ...................-------.........................-..............G...
10 Sus verrucosus               ...................--CCCCC....C....................T..................
11 Sus verrucosus               ...-...............-------.........................-..................
4 Sus celebensis                ...................-------.....T...................-..................
7 Sus celebensis                ...................-------....................A....-..................
10 Sus celebensis               ...................-------.........................-..............G...
2 Potamochoerus larvatus        ...................-------.........................-..................
3 Potamochoerus larvatus        ...................-------......................G..-..................
4 Potamochoerus larvatus        ...................-------.........................-..................
3 Potamochoerus porcus          ...................-------....C....................-..................
4 Potamochoerus porcus          ...................-------.........................-..................
6 Potamochoerus porcus          .C.................-------.........................-..................
8 Potamochoerus porcus          .......G..T........-------....C...................T-..................
5 Hylochoerus meinertzhageni    ...................-------....C....................-..................
9 Hylochoerus meinertzhageni    ...................-------....C....................-..................
10 Hylochoerus meinertzhageni   ...................-------....C....................-..................
1 Phacochoerus africanus        ...................-------....C..............A.....-..................
4 Phacochoerus africanus        ...................-------....C....................-..................
6 Phacochoerus africanus        ...................-------....C..............A.....-..................
10 Phacochoerus africanus       ...................-------....C..............A.....-..................
11 Phacochoerus africanus       ...................-------....-..............T.....-..................
14 Phacochoerus africanus       ...................-------....C..............A.....-..................
15 Phacochoerus africanus       ...................-------....C..............A.....-..................
5 Phacochoerus aethiopicus      ...................-------....C....................-.......A..........
14 Phacochoerus aethiopicus     ...................-------....C....................-..................
15 Phacochoerus aethiopicus     ....T..............-------....C....................-..................

                                        80        90       100       110       120       130       140
                                ....|....|....|....|....|....|....|....|....|....|....|....|....|....|
Seq1 Sscrofa8 chromosome1       CAGGGCTCATAA-TTTGTCAGTTCAGGTTAAGAAGGGACCTTGGCAGACTTTCTGTGTCTCTGAATGGCC
Seq2 Sscrofa8 chromosome1       ............-.............................................C............
Seq3 Sscrofa8 chromosome3       ............-.............................................C............
Seq4 Sscrofa8 chromosome5       ............-.........................................................
```

Seq5 Sscrofa8 chromosome7	............-...
Seq6 Sscrofa8 chromosome8	............-...
Seq7 Sscrofa8 chromosome13	............-..**C**............
Seq8 Sscrofa8 chromosome13	............-..**C**............
Seq9 Sscrofa8 chromosome13	............-............**T**...............................**C**............
Seq10 Sscrofa8 chromosome14	............-...
Seq11 Sscrofa8 chromosome16	............-..**C**............
Seq12 Sscrofa8 chromosomeX	............-...
Seq13 Sscrofa8 chromosomeX	............-..**C**............
Sus scrofa(AF435967)	............-..**G**............
Sus scrofa(EF133960)	............-..**C**............
Sus scrofa(AY437840)	............-..**C**............
Sus scrofa(AJ279057)	............-..**C**............
Sus scrofa(Y17013)	............-..**C**............
Sus scrofa(AY056035)	............**T**..**C**............
Sus scrofa(AF147808)	............-..**C**............
Sus scrofa(EU523109)	............-..**C**............
Sus scrofa(AF435966)	............-..**C**............
Sus scrofa(AJ133816)	............-..**C**............
Sus scrofa(AJ133818)	............-..**C**............
Sus scrofa(AJ293657)	............-..**C**............
Sus scrofa(AY099323)	............-..**C**............
Sus scrofa(AJ133817)	............-..**C**............
Sus scrofa(AY099324)	............-..**C**............
Sus scrofa(AM229312)	............-...
Sus scrofa(AM229311)	............-...
Sus scrofa(DQ996272)	............-...
Sus scrofa(AY570980)	............-...
Sus scrofa(AY368583)	............-...
Sus scrofa(AY953542)	............-...
Sus scrofa(AY437841)	............-...
Sus scrofa(AY368585)	............-...
Sus scrofa(AY368584)	............-...
Sus scrofa(AY368586)	............-...
Sus scrofa(AJ279056)	............-...
Sus scrofa(EU789636)	............-...
Sus scrofa(AJ293656)	............-...
3 *Sus scrofa*	..**T**.........-...
6 *Sus scrofa*	............-..**C**....**T**.......
9 *Sus scrofa*	............-..**C**............
11 *Sus scrofa*	............-...
3 *Sus barbatus barbatus*	............-.....................**A**.....------------------------------
4 *Sus barbatus barbatus*	............-..**C**............
9 *Sus barbatus barbatus*	............-......**G**.....................................**C**............
10 *Sus barbatus barbatus*	............-......**T**..............**A**......................**C**............
2 *Sus barbatus oi*	............-...

```
4 Sus barbatus oi                      ............-.........................................................
11 Sus barbatus oi                     ............-.........................................................
17 Sus barbatus oi                     ............-............................................C............
2 Sus verrucosus                       ............-..............................................C..........
10 Sus verrucosus                      .G..........-.........................................................
11 Sus verrucosus                      ............-.........................................................
4 Sus celebensis                       ............-.........................................................
7 Sus celebensis                       ............-.........................................................
10 Sus celebensis                      ............-..........................................A.C............
2 Potamochoerus larvatus               ............-............................................C............
3 Potamochoerus larvatus               ............-............................................C...........T
4 Potamochoerus larvatus               ............-............................................C............
3 Potamochoerus porcus                 ............-............................................C............
4 Potamochoerus porcus                 ............-...........................C................C............
6 Potamochoerus porcus                 ............-............................................C............
8 Potamochoerus porcus                 ..........G.-............................................C......G.....
5 Hylochoerus meinertzhageni           ............-...........................C................C............
9 Hylochoerus meinertzhageni           ............-...........................C................C............
10 Hylochoerus meinertzhageni          ............-............................................C............
1 Phacochoerus africanus               ............-.................G...A.....C................C......G.....
4 Phacochoerus africanus               ............-............................................C............
6 Phacochoerus africanus               ............-.................G...A.....C................C......G.....
10 Phacochoerus africanus              ............-.................G...A.....C................C......G.....
11 Phacochoerus africanus              ............-...........................C................C............
14 Phacochoerus africanus              ............-.................G...A.....C................C......G.....
15 Phacochoerus africanus              ............-.................G...A.....C................C......G.....
5 Phacochoerus aethiopicus             ............-...........................------------------------------
14 Phacochoerus aethiopicus            ............-C...........................................C............
15 Phacochoerus aethiopicus            ............-...................G........................C............

                                                150       160       170       180       190       200       210
                                       ....|....|....|....|....|....|....|....|....|....|....|....|....|....|
Seq1 Sscrofa8 chromosome1              GACATTCGATGTTGGATGGCCATCAGAGGGGACCTTTAATTCTGAGATTATCCTGGCTGTTAAAGCAATT
Seq2 Sscrofa8 chromosome1              A............................................A.................G.....C
Seq3 Sscrofa8 chromosome3              A............................................A.................G.....C
Seq4 Sscrofa8 chromosome5              ......................................................................
Seq5 Sscrofa8 chromosome7              ......................................................................
Seq6 Sscrofa8 chromosome8              ......................................................................
Seq7 Sscrofa8 chromosome13             A............................................A.....T............A.....
Seq8 Sscrofa8 chromosome13             A............A........................................................
Seq9 Sscrofa8 chromosome13             ......T...............................................................
Seq10 Sscrofa8 chromosome14            ......................................................................
Seq11 Sscrofa8 chromosome16            A.....A......................................A.................G.....C
Seq12 Sscrofa8 chromosomeX             ......................................................................
Seq13 Sscrofa8 chromosomeX             ......................................................................
Sus scrofa(AF435967)                   ...........G.............A.C...................A......T....G.C........
```

```
Sus scrofa(EF133960)             A.............................................A.....T............A.....
Sus scrofa(AY437840)             A.............................................A.................G.....C
Sus scrofa(AJ279057)             A.............................................A.................G...G.C
Sus scrofa(Y17013)               A.............................................A.................G.....C
Sus scrofa(AY056035)             A.....A.......................................A.................G.....C
Sus scrofa(AF147808)             A.............................................A.................G.....C
Sus scrofa(EU523109)             A.............................................A.................G.....C
Sus scrofa(AF435966)             A.............................................A.................G.....C
Sus scrofa(AJ133816)             A.............................................A.................G.....C
Sus scrofa(AJ133818)             A.............................................A.................G.....C
Sus scrofa(AJ293657)             A.............................................A.................G.....C
Sus scrofa(AY099323)             A.............................................A.................G.....C
Sus scrofa(AJ133817)             A.............................................A.................G.....C
Sus scrofa(AY099324)             A.............................................A.................G.....C
Sus scrofa(AM229312)             .......................................................................
Sus scrofa(AM229311)             .......................................................................
Sus scrofa(DQ996272)             .......................................................................
Sus scrofa(AY570980)             .......................................................................
Sus scrofa(AY368583)             .......................................................................
Sus scrofa(AY953542)             .......................................................................
Sus scrofa(AY437841)             .......................................................................
Sus scrofa(AY368585)             .......................................................................
Sus scrofa(AY368584)             .......................................................................
Sus scrofa(AY368586)             .......................................................................
Sus scrofa(AJ279056)             .......................................................................
Sus scrofa(EU789636)             .......................................................................
Sus scrofa(AJ293656)             .......................................................................
3 Sus scrofa                     .......................................................................
6 Sus scrofa                     ........G.................T.A..........................................
9 Sus scrofa                     A............A.........................................................
11 Sus scrofa                    .................................T............A.......................C
3 Sus barbatus barbatus          ----------------------------------.....................................
4 Sus barbatus barbatus          A.............................................A.......................C
9 Sus barbatus barbatus          ................CA............................A.......................C
10 Sus barbatus barbatus         A.............................................A.......................C
2 Sus barbatus oi                .........................................................T.............
4 Sus barbatus oi                .......A.........................T...........................A.........
11 Sus barbatus oi               .......A.........................T...........................A.........
17 Sus barbatus oi               .......A..........A..............T...........................A.........
2 Sus verrucosus                 .......................................................................
10 Sus verrucosus                .......................................................................
11 Sus verrucosus                .......................................................................
4 Sus celebensis                 .......A.........................T...........................A.........
7 Sus celebensis                 .........................................................T.............
10 Sus celebensis                A.............................................A.......................C
2 Potamochoerus larvatus         A......................................................................
```

```
3 Potamochoerus larvatus         ......T...............................................................
4 Potamochoerus larvatus         ......................................................................
3 Potamochoerus porcus           ....................T.................................................
4 Potamochoerus porcus           .............................................A......T..A..............
6 Potamochoerus porcus           .............................................A......T..A..............
8 Potamochoerus porcus           ....C........................................A......T..A..............
5 Hylochoerus meinertzhageni     ......................................................................
9 Hylochoerus meinertzhageni     ......................................................................
10 Hylochoerus meinertzhageni    ......................................................................
1 Phacochoerus africanus         .............................................A......T.AA..............
4 Phacochoerus africanus         ....................T.................................................
6 Phacochoerus africanus         .............................................A......T.AA..............
10 Phacochoerus africanus        .........C...................................A......T.AA..............
11 Phacochoerus africanus        .............................................A......T..A..............
14 Phacochoerus africanus        .............................................A......T.AA..............
15 Phacochoerus africanus        ...............................G.............A......T.AA..............
5 Phacochoerus aethiopicus       ----------------------------------....................................
14 Phacochoerus aethiopicus      .......A..............................................................
15 Phacochoerus aethiopicus      A............A........................................................

                                         220       230       240       250       260       270       280
                                 ....|....|....|....|....|....|....|....|....|....|....|....|....|....|
Seq1 Sscrofa8 chromosome1        ATTTTTCAGACTGGACCCGGCTCTCATCCCGATCAGGAGCCCTATATCCTTACGTGGCAAGATTTGGCAG
Seq2 Sscrofa8 chromosome1        .............................T........................................
Seq3 Sscrofa8 chromosome3        ..................A..........T........................................
Seq4 Sscrofa8 chromosome5        ..............................A.......................................
Seq5 Sscrofa8 chromosome7        ......................................................................
Seq6 Sscrofa8 chromosome8        ......................................................................
Seq7 Sscrofa8 chromosome13       .............................T..............................A.....A...
Seq8 Sscrofa8 chromosome13       .............................T........................................
Seq9 Sscrofa8 chromosome13       .............................T........................................
Seq10 Sscrofa8 chromosome14      ......................................................................
Seq11 Sscrofa8 chromosome16      .............................T........................................
Seq12 Sscrofa8 chromosomeX      ........................................................A.............
Seq13 Sscrofa8 chromosomeX      .............................T........................................
Sus scrofa(AF435967)             ..................C.....T..T.......C..A..........C......T.....A......A
Sus scrofa(EF133960)             .............................T.................T............A.....A...
Sus scrofa(AY437840)             .............................T........................................
Sus scrofa(AJ279057)             ..................A..........T........................................
Sus scrofa(Y17013)               ..................A..........T................................C.......
Sus scrofa(AY056035)             .............................T........................................
Sus scrofa(AF147808)             .............................T........................................
Sus scrofa(EU523109)             ..................A..........T........................................
Sus scrofa(AF435966)             ..................A..........T................................C.......
Sus scrofa(AJ133816)             ..................A..........T................................C.......
Sus scrofa(AJ133818)             ..................A..........T................................C.......
```

Sus scrofa(AJ293657)	.............................T..
Sus scrofa(AY099323)	.............................T..
Sus scrofa(AJ133817)	.............................T..
Sus scrofa(AY099324)	.............................T..
Sus scrofa(AM229312)	....
Sus scrofa(AM229311)	....
Sus scrofa(DQ996272)	....
Sus scrofa(AY570980)	....
Sus scrofa(AY368583)	....A................
Sus scrofa(AY953542)	..............................A.......................................
Sus scrofa(AY437841)	....G..........
Sus scrofa(AY368585)	....
Sus scrofa(AY368584)	....
Sus scrofa(AY368586)	....
Sus scrofa(AJ279056)	....
Sus scrofa(EU789636)	....
Sus scrofa(AJ293656)	....
3 *Sus scrofa*	....
6 *Sus scrofa*	..................A.................................T.................
9 *Sus scrofa*	..............................A.......................................
11 *Sus scrofa*	.............................T.......................A................
3 *Sus barbatus barbatus*	.............................T.......................A................
4 *Sus barbatus barbatus*	.............................T..........................A.............
9 *Sus barbatus barbatus*	................T............T..
10 *Sus barbatus barbatus*	..................A..........T..
2 *Sus barbatus oi*	....
4 *Sus barbatus oi*	....
11 *Sus barbatus oi*	....
17 *Sus barbatus oi*	....
2 *Sus verrucosus*	....
10 *Sus verrucosus*	....
11 *Sus verrucosus*	....
4 *Sus celebensis*	....
7 *Sus celebensis*	....
10 *Sus celebensis*	..................A..........T..............C.........................
2 *Potamochoerus larvatus*	.............................T.......................A.............T..
3 *Potamochoerus larvatus*	.............................T.......................A.............T..
4 *Potamochoerus larvatus*	.............................T.......................A.............T..
3 *Potamochoerus porcus*	.............................T..
4 *Potamochoerus porcus*	..........T............C......A................T......................
6 *Potamochoerus porcus*	.......................C....G..................T......................
8 *Potamochoerus porcus*	..........T.......A....C.......................T......................
5 *Hylochoerus meinertzhageni*	.............................T..
9 *Hylochoerus meinertzhageni*	.............................T..
10 *Hylochoerus meinertzhageni*	.............................T....................C...................
1 *Phacochoerus africanus*	.......................C.......................T......................

```
4 Phacochoerus africanus        .............................T...G....................................
6 Phacochoerus africanus        .......................C.......................T......................
10 Phacochoerus africanus       .......................C.......................T......................
11 Phacochoerus africanus       ..........T.......A....C......A................T......................
14 Phacochoerus africanus       ...............................................T......................
15 Phacochoerus africanus       .......................C.......................T......................
5 Phacochoerus aethiopicus      ..........T............C........G..............T......................
14 Phacochoerus aethiopicus     ............................T.C................T......................
15 Phacochoerus aethiopicus     .............................T........................................

                                         290       300       310       320       330       340       350
                                ....|....|....|....|....|....|....|....|....|....|....|....|....|....|
Seq1 Sscrofa8 chromosome1       AGGATCCTCCGCCATGGGTTAAACCTTGGCTGAATAAGCCAAGAAAGCCAGGTCCCCGAATTCTGGCTCT
Seq2 Sscrofa8 chromosome1       .A.......................A.....A.....A.......................C........
Seq3 Sscrofa8 chromosome3       .A.......................A.....A.....A.......................C........
Seq4 Sscrofa8 chromosome5       .........................A............................................
Seq5 Sscrofa8 chromosome7       ......................................................................
Seq6 Sscrofa8 chromosome8       .........................A............................................
Seq7 Sscrofa8 chromosome13      .A.............A....T....A.....A.....A.......................C........
Seq8 Sscrofa8 chromosome13      .........................A............................................
Seq9 Sscrofa8 chromosome13      .........................A............................................
Seq10 Sscrofa8 chromosome14     .........................A............................................
Seq11 Sscrofa8 chromosome16     .A.......................A.....A.....A.......................C........
Seq12 Sscrofa8 chromosomeX      .........................A............................................
Seq13 Sscrofa8 chromosomeX      .A.......................A.....A.....A.......................C........
Sus scrofa(AF435967)            ...................C....................CC....AG.CC......C..A.......T.
Sus scrofa(EF133960)            .A.......................A.....A.....A.......................C........
Sus scrofa(AY437840)            .A.......................A.....A.....A.......................C........
Sus scrofa(AJ279057)            .A........A..............A.....A.....A.......................C........
Sus scrofa(Y17013)              .A.......................A.....A.....A.......................C........
Sus scrofa(AY056035)            .A.......................A.....A.....A.......................C........
Sus scrofa(AF147808)            .A.......................A.....A.....A.......................C........
Sus scrofa(EU523109)            .A.......................A.....A.....A.......................C........
Sus scrofa(AF435966)            .A.......................A.....A.....A.......................C........
Sus scrofa(AJ133816)            .A.......................A.....A.....A.......................C........
Sus scrofa(AJ133818)            .A.......................A.....A.....A.......................C........
Sus scrofa(AJ293657)            .A..A....................A.....A.....A.......................C........
Sus scrofa(AY099323)            .A.......................A.....A.....A.......................C........
Sus scrofa(AJ133817)            .A.......................A.....A.....A.......................C........
Sus scrofa(AY099324)            .A.......................A.....A.....A.......................C........
Sus scrofa(AM229312)            .........................A............................................
Sus scrofa(AM229311)            .........................A............................................
Sus scrofa(DQ996272)            .........................A............................................
Sus scrofa(AY570980)            .........................A............................................
Sus scrofa(AY368583)            ......................................................................
Sus scrofa(AY953542)            ......................................................................
```

```
Sus scrofa(AY437841)              ......................................................................
Sus scrofa(AY368585)              ......................................................................
Sus scrofa(AY368584)              ......................................................................
Sus scrofa(AY368586)              ......................................................................
Sus scrofa(AJ279056)              ......................................................................
Sus scrofa(EU789636)              ......................................................................
Sus scrofa(AJ293656)              ......................................................................
3 Sus scrofa                      .........................A............................................
6 Sus scrofa                      ............T............A...........A...................A............
9 Sus scrofa                      ......................G..A.....A.....A.......................C........
11 Sus scrofa                     .........................A...........A................................
3 Sus barbatus barbatus           .A.......................A.....A.....A.....................G..........
4 Sus barbatus barbatus           .A.......................A.....A.....A..........G........A.G..........
9 Sus barbatus barbatus           .A......T................A.....A.....A.....................G..........
10 Sus barbatus barbatus          .A.......................A.....A.....A.......................C........
2 Sus barbatus oi                 ..........A..............A...............................A............
4 Sus barbatus oi                 .............C...........A............................................
11 Sus barbatus oi                .........................A............................................
17 Sus barbatus oi                .........................A............................................
2 Sus verrucosus                  .........................A............................................
10 Sus verrucosus                 .........................A............................................
11 Sus verrucosus                 .........................A............................................
4 Sus celebensis                  .............C...........A............................................
7 Sus celebensis                  ..........A..............A...............................A............
10 Sus celebensis                 .A.......................A.....A.....A.......................C........
2 Potamochoerus larvatus          .A.......................A.....A.....A...................A............
3 Potamochoerus larvatus          .A.......................A...........A...................A............
4 Potamochoerus larvatus          .A.......................A.....A.....A...................A............
3 Potamochoerus porcus            .A.......................A.....A.....A..................T..G.......C..
4 Potamochoerus porcus            .AA.-..C..A..G........G..C.....AC....A.....G.............A.G..........
6 Potamochoerus porcus            .AA.C..C.....G........G..C.....A.....A...................A........T...
8 Potamochoerus porcus            .AA.-..C.A...G........G..C.....A.........................A....G.......
5 Hylochoerus meinertzhageni      .A....................G..C.....A.....A...................A.G..........
9 Hylochoerus meinertzhageni      .A....................G..C.....A.....A...................A.G..........
10 Hylochoerus meinertzhageni     .A.......T...............A.....A.....A..................T..G..........
1 Phacochoerus africanus          .AA.C..C..A..G........G..C.....A.....A.....G.............A............
4 Phacochoerus africanus          .A.......................A.....A.....A..................T..G..........
6 Phacochoerus africanus          .AA.C..C..A..G........G..C.....A.....A.....G.............A............
10 Phacochoerus africanus         .AA.A..C..A..G........G..C.....A.....A.....G.............A............
11 Phacochoerus africanus         .AA.C..C.A...G........G..C.....A.....A...................A........T...
14 Phacochoerus africanus         .AA.C..C..A..G........G..C.....A.....A.....G.............A............
15 Phacochoerus africanus         .AA.C..C..A..G........G..C.....A.....A.....G.............A............
5 Phacochoerus aethiopicus        .AA.C..C.A...G........G..C.....---------------------------------------
14 Phacochoerus aethiopicus       .A.......................C.....A.....A...................A............
15 Phacochoerus aethiopicus       .........................A............................................
```

	360 370 380 390 400 410 420
	....|....|....|....|....|....|....|....|....|....|....|....|....|....|
Seq1 Sscrofa8 chromosome1	**TGGAGAGAAAAACAAACACTCGGCT-AAAAAAGTCAAGCCCTCTCCTCATATCTACCCCGAGATTGAGGA**
Seq2 Sscrofa8 chromosome1	........................**C-G**........**G**............**G**...............**C**.....
Seq3 Sscrofa8 chromosome3	........................**C-G**........**G**............**G**...............**C**.....
Seq4 Sscrofa8 chromosome5	.........................**-G**.....................**-**................**A**.**A**..
Seq5 Sscrofa8 chromosome7	.........................**-G**...
Seq6 Sscrofa8 chromosome8	.........................**-G**...
Seq7 Sscrofa8 chromosome13	...............**G**........**C-**.........**G**.**A**..........**G**.........**T**.....**C**.....
Seq8 Sscrofa8 chromosome13	.........................**-G**............**T**..............................
Seq9 Sscrofa8 chromosome13	.........................**-G**...
Seq10 Sscrofa8 chromosome14	.........................**-G**...
Seq11 Sscrofa8 chromosome16	........................**C-G**........**G**............**G**...............**C**.....
Seq12 Sscrofa8 chromosomeX	.........................**-G**...
Seq13 Sscrofa8 chromosomeX	........................**C-G**........**G**............**G**...............**C**.....
Sus scrofa(AF435967)	.............**C**...........**-G**...
Sus scrofa(EF133960)	...............**G**........**C-**.........**G**.**A**..........**G**...............**C**.....
Sus scrofa(AY437840)	........................**C-G**........**G**...................**T**........**C**.....
Sus scrofa(AJ279057)	........................**C-G**........**G**............**G**..........**A**....**C**.....
Sus scrofa(Y17013)	...**G**....................**C-G**........**G**............**G**...............**C**.....
Sus scrofa(AY056035)	........................**C-G**........**G**............**G**...............**C**.....
Sus scrofa(AF147808)	........................**C-G**........**G**............**G**...............**C**.....
Sus scrofa(EU523109)	........................**C-G**........**G**............**G**...............**C**.....
Sus scrofa(AF435966)	........................**C-G**........**G**............**G**...............**C**.....
Sus scrofa(AJ133816)	........................**C-G**........**G**............**G**...............**C**.....
Sus scrofa(AJ133818)	........................**C-G**........**G**............**G**...............**C**.....
Sus scrofa(AJ293657)	........................**C-G**........**G**...........**GG**...............**C**.....
Sus scrofa(AY099323)	........................**C-G**........**G**............**G**...............**C**.....
Sus scrofa(AJ133817)	........................**C-G**........**G**............**G**...............**C**.....
Sus scrofa(AY099324)	........................**C-G**........**G**............**G**...............**C**.....
Sus scrofa(AM229312)	.........................**-G**...
Sus scrofa(AM229311)	.........................**-G**...
Sus scrofa(DQ996272)	.........................**-G**...
Sus scrofa(AY570980)	.........................**-G**...
Sus scrofa(AY368583)	.........................**-G**...
Sus scrofa(AY953542)	.........................**-G**...
Sus scrofa(AY437841)	.........................**-G**...
Sus scrofa(AY368585)	.........................**-G**...
Sus scrofa(AY368584)	.........................**-G**...
Sus scrofa(AY368586)	.........................**-G**...
Sus scrofa(AJ279056)	.........................**-G**...
Sus scrofa(EU789636)	.........................**-**..
Sus scrofa(AJ293656)	.........................**-G**...
3 *Sus scrofa*	.........................**-G**...
6 *Sus scrofa*	.........................**-G**...
9 *Sus scrofa*	........................**C-G**........**G**............**G**...............**C**.....

```
11 Sus scrofa                      .........................-G.............A.A...........................
3 Sus barbatus barbatus            ........................CA......................C.....................
4 Sus barbatus barbatus            C.......................C-G........G............G...............C.....
9 Sus barbatus barbatus            ........................C-G...........................................
10 Sus barbatus barbatus           ........................C-G........G............G...............C.....
2 Sus barbatus oi                  ......................A..-G...........................................
4 Sus barbatus oi                  ..................T......-G...........................................
11 Sus barbatus oi                 ..................T......-............................................
17 Sus barbatus oi                 ..................T......-G...........................................
2 Sus verrucosus                   .........................-G...........................................
10 Sus verrucosus                  .........................-G...........................................
11 Sus verrucosus                  .........................-G...........................................
4 Sus celebensis                   ..................T......-G...........................................
7 Sus celebensis                   ......................A..-G...........................................
10 Sus celebensis                  ........................C-G........G............G...............C.....
2 Potamochoerus larvatus           ............T............-G...........................................
3 Potamochoerus larvatus           ............T........A...-G...........................................
4 Potamochoerus larvatus           ............T............-G...........................................
3 Potamochoerus porcus             ........................C-.........G............G..........C..........
4 Potamochoerus porcus             ..A.............T.......C-G..........AT.........G.........T...........
6 Potamochoerus porcus             .......................TC-G..........A.A..............................
8 Potamochoerus porcus             ............T............-G...........................................
5 Hylochoerus meinertzhageni       .......-......G.........C-G.....................G.....................
9 Hylochoerus meinertzhageni       .......-................C-G.....................G.....................
10 Hylochoerus meinertzhageni      ........................C-G.....................G.....................
1 Phacochoerus africanus           ..A.............T.......C-...........AT.........G.....................
4 Phacochoerus africanus           ........................C-G.....................G.........T...........
6 Phacochoerus africanus           ..A.............T.......C-...........AT.........G.....................
10 Phacochoerus africanus          ..A.............T.......CA...........AT.........G.....................
11 Phacochoerus africanus          ..A.T...........T.......C-G.....................G.........T...........
14 Phacochoerus africanus          ..A.............T.......C-...........AT.........G.....................
15 Phacochoerus africanus          ..A.............T.......C-...........AT.........G.....................
5 Phacochoerus aethiopicus         ---------...A............-G................................A..........
14 Phacochoerus aethiopicus        .....................A..C-G.....................G.........T...........
15 Phacochoerus aethiopicus        .........................-G............T..............................

                                           430       440       450       460       470       480       490
                                   ....|....|....|....|....|....|....|....|....|....|....|....|....|....|
Seq1 Sscrofa8 chromosome1          GCCGCCGGCTTGGCCGGAACCCCAATCTGTT------CCCCCACCCCCTTATCCGGCACAGGGTGCTGC-
Seq2 Sscrofa8 chromosome1          .......A.................C.....------.................A.............T-
Seq3 Sscrofa8 chromosome3          .......A.................C.....------.................A.............T-
Seq4 Sscrofa8 chromosome5          ...............A...............------......A...............G.........-
Seq5 Sscrofa8 chromosome7          ...............................------................................-
Seq6 Sscrofa8 chromosome8          ...............................------................................-
Seq7 Sscrofa8 chromosome13         .......A.................C.....------.................A..............-
Seq8 Sscrofa8 chromosome13         ...............................------......A...............G.........-
```

```
Seq9 Sscrofa8 chromosome13       ...............................------................................-
Seq10 Sscrofa8 chromosome14      ...A...........................------................T............C..-
Seq11 Sscrofa8 chromosome16      .......A.................C.....------.................A.............T-
Seq12 Sscrofa8 chromosomeX       ...............................------................................-
Seq13 Sscrofa8 chromosomeX       .......A.................C.....------.................A.............T-
Sus scrofa(AF435967)             ...............................------................................-
Sus scrofa(EF133960)             .......A.................C.....------......T..........A..............-
Sus scrofa(AY437840)             .......A.................C.....------.................A.............T-
Sus scrofa(AJ279057)             .......A.................C.....------.................A.............T-
Sus scrofa(Y17013)               .......A.................C.....------.................A.............T-
Sus scrofa(AY056035)             .......A.................C.....------.................A.............T-
Sus scrofa(AF147808)             .......A.................C.....------.................A.............T-
Sus scrofa(EU523109)             .......A.................C.....------.................A.............T-
Sus scrofa(AF435966)             .......A.................C.....------.................A.............T-
Sus scrofa(AJ133816)             .......A.................C.....------.................A.............T-
Sus scrofa(AJ133818)             .......A.................C.....------.................A.............T-
Sus scrofa(AJ293657)             .......A.................C.....------.................A.............T-
Sus scrofa(AY099323)             .......A.................C.....------.................A.............T-
Sus scrofa(AJ133817)             .......A.................C.....------.................A.............T-
Sus scrofa(AY099324)             .......A.................C.....------.................A.............T-
Sus scrofa(AM229312)             ...A...........................------................T............C..-
Sus scrofa(AM229311)             ...A...........................------................T............C..-
Sus scrofa(DQ996272)             ...A...........................------................T............C..-
Sus scrofa(AY570980)             ...A...........................------................T............C..-
Sus scrofa(AY368583)             ............C..................------................................-
Sus scrofa(AY953542)             ...............................------................T...............-
Sus scrofa(AY437841)             ...............................------................................-
Sus scrofa(AY368585)             ...............................------................................-
Sus scrofa(AY368584)             ...............................------................................-
Sus scrofa(AY368586)             .........CG....................------................................-
Sus scrofa(AJ279056)             ...............................------................................-
Sus scrofa(EU789636)             ...............................------................................-
Sus scrofa(AJ293656)             ...............................------................................-
3 Sus scrofa                     ...............................------................................-
6 Sus scrofa                     ..T..............T......G......------T...............T...............-
9 Sus scrofa                     .......A.................C.....------.................A.............T-
11 Sus scrofa                    ..T..T.........................------................T..............T-
3 Sus barbatus barbatus          ..T....A.................C.....CCCCCA...............................A-
4 Sus barbatus barbatus          .......A.................C.....------.................A..............-
9 Sus barbatus barbatus          ...............................------..............C.................-
10 Sus barbatus barbatus         .......A.................C.....------.................A..............-
2 Sus barbatus oi                ..T............A..G............------................................-
4 Sus barbatus oi                ...............................------..............C.................-
11 Sus barbatus oi               ...............................------..............C.................-
17 Sus barbatus oi               ...............................------..............C.................-
2 Sus verrucosus                 ...............................------................................-
```

10 *Sus verrucosus*	...A...........................------................................-
11 *Sus verrucosus*	...............................------................................-
4 *Sus celebensis*	...............................------..............C.................-
7 *Sus celebensis*	..T...............G............------................................-
10 *Sus celebensis*	.......A.................C.....------.................A..............-
2 *Potamochoerus larvatus*	...A.T.........................------.................A..............-
3 *Potamochoerus larvatus*	...A.T.........................------.................A..............-
4 *Potamochoerus larvatus*	...A.T.........................------.................A..............-
3 *Potamochoerus porcus*	.......A.................C.....------................T......C........-
4 *Potamochoerus porcus*	.......A.................C.....------................T.T......G.....-
6 *Potamochoerus porcus*	.......A.................C.....------................T.........G.....-
8 *Potamochoerus porcus*	......A........................------................................-
5 *Hylochoerus meinertzhageni*	.......A.................C.....------..........................G.....-
9 *Hylochoerus meinertzhageni*	.......A.................C.....------..........................G.....-
10 *Hylochoerus meinertzhageni*	...---------.............C.....------...........G...........C........-
1 *Phacochoerus africanus*	.......A.................C.....------................T.T......G.....-
4 *Phacochoerus africanus*	.......A.................C.....------..T........G.....A.....C........-
6 *Phacochoerus africanus*	.......A.................C.....------................T.T......G.....-
10 *Phacochoerus africanus*	.T.....A.................C.....------................T.T......G.....-
11 *Phacochoerus africanus*	A......A.................C.....------................T.........G.....G
14 *Phacochoerus africanus*	.......A.................C.....------................T.T......G.....-
15 *Phacochoerus africanus*	.......A.................C.....------...T............T.T......G.....-
5 *Phacochoerus aethiopicus*	...............................------................T...............-
14 *Phacochoerus aethiopicus*	..T....A...................C...------................T.........G.....-
15 *Phacochoerus aethiopicus*	........................G......------......A...............G.........-

	500 510 520 530 540 550 560
	....|....|....|....|....|....|....|....|....|....|....|....|....|....|
Seq1 Sscrofa8 chromosome1	**GAGGGGACCCTCTGCCCCTCCTGGAGCTCCGGCGGTGGAGGGACCTGCTGCAGGGACTCGGAGCCGGAGG**
Seq2 Sscrofa8 chromosome1	................................T..................C.................A
Seq3 Sscrofa8 chromosome3	................................T..................C.................A
Seq4 Sscrofa8 chromosome5	..................................A...................................
Seq5 Sscrofa8 chromosome7	....
Seq6 Sscrofa8 chromosome8	....T.....
Seq7 Sscrofa8 chromosome13	...................A............T..C...............C........A.........
Seq8 Sscrofa8 chromosome13	....
Seq9 Sscrofa8 chromosome13	................................A...............................T.....
Seq10 Sscrofa8 chromosome14	...........T..
Seq11 Sscrofa8 chromosome16	................................T..................C.................A
Seq12 Sscrofa8 chromosomeX	....
Seq13 Sscrofa8 chromosomeX	................................T..................C.................A
Sus scrofa(AF435967)	....T..............C.......
Sus scrofa(EF133960)	............C......A............T..C...............C........A.........
Sus scrofa(AY437840)	................................T..................C.................A
Sus scrofa(AJ279057)	................................T..................C.................A
Sus scrofa(Y17013)	................................T..................C.................A

```
Sus scrofa(AY056035)           ................................T..................C.................A
Sus scrofa(AF147808)           ................................T..................C.................A
Sus scrofa(EU523109)           ................................T..................C.................A
Sus scrofa(AF435966)           ................................T..................C.................A
Sus scrofa(AJ133816)           ................................T..................C.................A
Sus scrofa(AJ133818)           ................................T..................C.................A
Sus scrofa(AJ293657)           ................................T..................C.................A
Sus scrofa(AY099323)           ................................T..................C.................A
Sus scrofa(AJ133817)           ................................T..................C.................A
Sus scrofa(AY099324)           ................................T..................C.................A
Sus scrofa(AM229312)           ...........T..........................................................
Sus scrofa(AM229311)           ...........T..........................................................
Sus scrofa(DQ996272)           ...........T..........................................................
Sus scrofa(AY570980)           ...........T..........................................................
Sus scrofa(AY368583)           .........................................A............................
Sus scrofa(AY953542)           ......................................................................
Sus scrofa(AY437841)           ......................................................................
Sus scrofa(AY368585)           ......................................................................
Sus scrofa(AY368584)           ......................................................................
Sus scrofa(AY368586)           ......................................................................
Sus scrofa(AJ279056)           ...............................................T..............C.......
Sus scrofa(EU789636)           ......................................................................
Sus scrofa(AJ293656)           ......................................................................
3 Sus scrofa                   ................................................................T.....
6 Sus scrofa                   .G...............................................................A....
9 Sus scrofa                   ................................T..................C.................A
11 Sus scrofa                  ......................................................................
3 Sus barbatus barbatus        ...................................C...............C.......A..........
4 Sus barbatus barbatus        ................................T..................C.................A
9 Sus barbatus barbatus        ......................................................................
10 Sus barbatus barbatus       ................................T..................C.................A
2 Sus barbatus oi              .................................A....................................
4 Sus barbatus oi              ......................................................................
11 Sus barbatus oi             ......................................................................
17 Sus barbatus oi             ......................................................................
2 Sus verrucosus               ..............................-.......................................
10 Sus verrucosus              A.....................................................................
11 Sus verrucosus              ................................T......A...........C.................A
4 Sus celebensis               ......................................................................
7 Sus celebensis               .................................A....................................
10 Sus celebensis              ................................T..................C.................A
2 Potamochoerus larvatus       ...A.A.......................T.....C...............C..................
3 Potamochoerus larvatus       ...A.A.......................T.....C...............C..................
4 Potamochoerus larvatus       ...A.A.......................T.....C...............C..................
3 Potamochoerus porcus         ...................................C...............C..................
4 Potamochoerus porcus         .G..........C......A.......C..A....C......G..C.....C...........T.....A
```

	570 580 590 600 610 620 630
6 *Potamochoerus porcus*	...........................C.......................C...........T.....A
8 *Potamochoerus porcus*	...A.A...........................A.C.............A.C....-.T...........
5 *Hylochoerus meinertzhageni*	...................................C...............C..................
9 *Hylochoerus meinertzhageni*	...................................C...............C..................
10 *Hylochoerus meinertzhageni*	...................................C...............C..................
1 *Phacochoerus africanus*	.G..........C......A.......C.......................C...........T.....A
4 *Phacochoerus africanus*	....A....
6 *Phacochoerus africanus*	.G..........C......A.......C.......................C...........T.....A
10 *Phacochoerus africanus*	.G..........C......A.......C.......................C...........T.....A
11 *Phacochoerus africanus*	.G.........................C..A....C...............C...........T.....A
14 *Phacochoerus africanus*	.G..........C......A.......C.......................C...........T.....A
15 *Phacochoerus africanus*	.G..........C......A.......C.......................C...........T.....A
5 *Phacochoerus aethiopicus*	..AAAAG............................C......G........C..A...............
14 *Phacochoerus aethiopicus*	...........................A.......C..............GC.............A....
15 *Phacochoerus aethiopicus*	....
	....|....|....|....|....|....|....|....|....|....|....|....|....|....|
Seq1 Sscrofa8 chromosome1	**GGCGCCACCCCGGAGCGGACAGACGAGATCGCGACATTACCGCTGCGCACGTACGGCCCTCCCATACCGG**
Seq2 Sscrofa8 chromosome1	..................................T...............C..T...........G....
Seq3 Sscrofa8 chromosome3	..................................T...............C..T...........G....
Seq4 Sscrofa8 chromosome5	....----.A..............
Seq5 Sscrofa8 chromosome7	....
Seq6 Sscrofa8 chromosome8	....
Seq7 Sscrofa8 chromosome13	....C..T...........G..A.
Seq8 Sscrofa8 chromosome13	....
Seq9 Sscrofa8 chromosome13	....-...................
Seq10 Sscrofa8 chromosome14	....C.....
Seq11 Sscrofa8 chromosome16	..................................T...............C..T...........G....
Seq12 Sscrofa8 chromosomeX	....
Seq13 Sscrofa8 chromosomeX	..................................T...............C..T...........G....
Sus scrofa(AF435967)	....
Sus scrofa(EF133960)	....T..........................T..T...............C..T...........G....
Sus scrofa(AY437840)	................A.................................C..T...........G....
Sus scrofa(AJ279057)	....C..T...........G....
Sus scrofa(Y17013)	..................................T...............C..T...........G....
Sus scrofa(AY056035)	..................................T...............C..T...........G....
Sus scrofa(AF147808)	..................................T...............C..T...........G....
Sus scrofa(EU523109)	..................................T...............C..T...........G....
Sus scrofa(AF435966)	..................................T...............C..T...........G....
Sus scrofa(AJ133816)	..................................T...............C..T...........G....
Sus scrofa(AJ133818)	..................................T...............C..T...........G....
Sus scrofa(AJ293657)	..................................T...............C..T...........G..A.
Sus scrofa(AY099323)	..................................T...............C..T...........G..A.
Sus scrofa(AJ133817)	..................................T...............C..T...........G..A.
Sus scrofa(AY099324)	..................................T...............C..T...........G..A.

Sus scrofa(AM229312)	....C.....
Sus scrofa(AM229311)	....C.....
Sus scrofa(DQ996272)	....C.....
Sus scrofa(AY570980)	....C.....
Sus scrofa(AY368583)	....
Sus scrofa(AY953542)	....C.....
Sus scrofa(AY437841)	....N.............
Sus scrofa(AY368585)	....
Sus scrofa(AY368584)	....
Sus scrofa(AY368586)	....
Sus scrofa(AJ279056)	....
Sus scrofa(EU789636)	....
Sus scrofa(AJ293656)	....
3 *Sus scrofa*	....
6 *Sus scrofa*	..G.....................A...A.
9 *Sus scrofa*	..................................T...............C..T...........G....
11 *Sus scrofa*	..........T.....................A.....................................
3 *Sus barbatus barbatus*	...............................T.........C........C..T...........G..A.
4 *Sus barbatus barbatus*	..................................T...............C..T...........G....
9 *Sus barbatus barbatus*	....
10 *Sus barbatus barbatus*	..................................T...............C..T...........G....
2 *Sus barbatus oi*	....
4 *Sus barbatus oi*	....
11 *Sus barbatus oi*	....
17 *Sus barbatus oi*	....
2 *Sus verrucosus*	....
10 *Sus verrucosus*	....T................
11 *Sus verrucosus*	..............-...................T...............C..T...........G....
4 *Sus celebensis*	....G......
7 *Sus celebensis*	.......................T....................A.........................
10 *Sus celebensis*	..................................T...............C..T...........G....
2 *Potamochoerus larvatus*	................A....A...............................T................
3 *Potamochoerus larvatus*	................AA...A...............................T................
4 *Potamochoerus larvatus*	................A....A...............................T..........C.....
3 *Potamochoerus porcus*	...............................T....C.............C.TT...........G....
4 *Potamochoerus porcus*	..T..T.................T...G...T..................C..T...........G....
6 *Potamochoerus porcus*	...A.T.....A...............G...T..................T..T...........G....
8 *Potamochoerus porcus*	....G......C.....A...........G.T...........CA.....C..............G....
5 *Hylochoerus meinertzhageni*	..........T..................T.T..................C.TT...........G....
9 *Hylochoerus meinertzhageni*	..........T........A.........T.T..................C.TT...........G....
10 *Hylochoerus meinertzhageni*	...............................T..................C.TT...........G....
1 *Phacochoerus africanus*	.....T.T........C......T...G...T..................C..T...........G....
4 *Phacochoerus africanus*	................A............................T........................
6 *Phacochoerus africanus*	.....T.T........C......T...G...T..................C..T...........G....
10 *Phacochoerus africanus*	.....T.T........C......T...G...T..................C..T...........G....
11 *Phacochoerus africanus*	.....T.....................G...T.........A........C..T...........G....

```
14 Phacochoerus africanus     .....T.T........C......T...G...T..................C..T...........G....
15 Phacochoerus africanus     .....T.T........C......T...G...T..................C..T...........G....
5 Phacochoerus aethiopicus    ................A......AC.........................C..............G....
14 Phacochoerus aethiopicus   ...A.T.....................G...T..........GC......C..T...........G....
15 Phacochoerus aethiopicus   ......................................................................

                                     640       650       660       670       680       690       700
                              ....|....|....|....|....|....|....|....|....|....|....|....|....|....|
Seq1 Sscrofa8 chromosome1     GGGGCCAATTGCAGCCCCTCCAGTATTGGCCCTTTTCTTCTGCAGATCTCTATAATTGG-AAAACTAACC
Seq2 Sscrofa8 chromosome1     ...........................................................-..........
Seq3 Sscrofa8 chromosome3     ...........................................................-..........
Seq4 Sscrofa8 chromosome5     ...........................................................A..........
Seq5 Sscrofa8 chromosome7     ...........................................................-..........
Seq6 Sscrofa8 chromosome8     ...........................................................-..........
Seq7 Sscrofa8 chromosome13    .A.........................................................-..........
Seq8 Sscrofa8 chromosome13    .........................................................A.-..........
Seq9 Sscrofa8 chromosome13    ...........................................................-..........
Seq10 Sscrofa8 chromosome14   ...........................................................-..........
Seq11 Sscrofa8 chromosome16   ...........................................................-..........
Seq12 Sscrofa8 chromosomeX    ...........................................................-..........
Seq13 Sscrofa8 chromosomeX    ...C....C..................................................-..........
Sus scrofa(AF435967)          ...........................................................-..........
Sus scrofa(EF133960)          ...........................................................-..........
Sus scrofa(AY437840)          ...........................................................-..........
Sus scrofa(AJ279057)          ...........................................................-..........
Sus scrofa(Y17013)            ...........................................................-..........
Sus scrofa(AY056035)          ...........................................................-..........
Sus scrofa(AF147808)          ...........................................................-..........
Sus scrofa(EU523109)          ...........................................................-..........
Sus scrofa(AF435966)          ...........................................................-..........
Sus scrofa(AJ133816)          ...........................................................-..........
Sus scrofa(AJ133818)          ...........................................................-..........
Sus scrofa(AJ293657)          ...........................................................-..........
Sus scrofa(AY099323)          ...........................................................-..........
Sus scrofa(AJ133817)          ...........................................................-..........
Sus scrofa(AY099324)          ...........................................................-..........
Sus scrofa(AM229312)          ...........................................................-..........
Sus scrofa(AM229311)          ...........................................................-..........
Sus scrofa(DQ996272)          ...........................................................-..........
Sus scrofa(AY570980)          ...........................................................-..........
Sus scrofa(AY368583)          ...........................................................-..........
Sus scrofa(AY953542)          ...........................................................-..........
Sus scrofa(AY437841)          ...........................................................-..........
Sus scrofa(AY368585)          ...........................................................-..........
Sus scrofa(AY368584)          ...........................................................-..........
Sus scrofa(AY368586)          ...........................................................-..........
```

```
Sus scrofa(AJ279056)               ...........................................................-..........
Sus scrofa(EU789636)               ...........................................................-..........
Sus scrofa(AJ293656)               ...........................................................-..........
3 Sus scrofa                       ...........................................................-..........
6 Sus scrofa                       ..........A..A....C........................................-..........
9 Sus scrofa                       ...........................................................-..........
11 Sus scrofa                      ..............................T............................-.......C..
3 Sus barbatus barbatus            ..........................G.C....................T........AA...T......
4 Sus barbatus barbatus            ......-...................................................--..........
9 Sus barbatus barbatus            ....................................................AT.....-..........
10 Sus barbatus barbatus           ...........................................................-..........
2 Sus barbatus oi                  ...........................................................-..........
4 Sus barbatus oi                  ...........................................................-..........
11 Sus barbatus oi                 ...........................................................-..........
17 Sus barbatus oi                 ...........................................................-..........
2 Sus verrucosus                   ...........................................................-..........
10 Sus verrucosus                  ...................................C.......................-..........
11 Sus verrucosus                  ...........................................................-..........
4 Sus celebensis                   ...........................................................-..........
7 Sus celebensis                   ...........................................................-..........
10 Sus celebensis                  ...........................................................-..........
2 Potamochoerus larvatus           ...........................................T...............-..........
3 Potamochoerus larvatus           ...........................................T..............A-..........
4 Potamochoerus larvatus           ...........................................T...............-..........
3 Potamochoerus porcus             ...........................................................-..........
4 Potamochoerus porcus             .T...................................C.....................-..........
6 Potamochoerus porcus             A....................................C.....................-..........
8 Potamochoerus porcus             ...........................C..AT...........................-..........
5 Hylochoerus meinertzhageni       ..........................................................--.......G..
9 Hylochoerus meinertzhageni       ..........................................................--.......G..
10 Hylochoerus meinertzhageni      ...........T.............C.....................T...........-..........
1 Phacochoerus africanus           .....................................C.....................-..........
4 Phacochoerus africanus           ...........................................T...............-..........
6 Phacochoerus africanus           .....................................C.....................-..........
10 Phacochoerus africanus          .....................................C.....................-..........
11 Phacochoerus africanus          .............................T.............................-..........
14 Phacochoerus africanus          .....................................C.....................-.......T..
15 Phacochoerus africanus          .....................................C.....................-..........
5 Phacochoerus aethiopicus         ...........................C..A............................-..........
14 Phacochoerus aethiopicus        .............A....................A....................G...-..........
15 Phacochoerus aethiopicus        ...........................................................-..........

                                           710       720       730       740       750       760       770
                                   ....|....|....|....|....|....|....|....|....|....|....|....|....|....|
Seq1 Sscrofa8 chromosome1          ATCCCCCTTTCTCGGAGGATCCCCAACGCCTCACGGGGTTGGTGGAGTCCCTTAT----GTTCTCTCACC
Seq2 Sscrofa8 chromosome1          .......................................................----...........
```

Seq3 Sscrofa8 chromosome3	....----...........
Seq4 Sscrofa8 chromosome5	....................................C...A..............----...........
Seq5 Sscrofa8 chromosome7	....----...........
Seq6 Sscrofa8 chromosome8	.C...----...........
Seq7 Sscrofa8 chromosome13	....----...........
Seq8 Sscrofa8 chromosome13	.......................G............C...A..............----...........
Seq9 Sscrofa8 chromosome13	..........T..----...........
Seq10 Sscrofa8 chromosome14	....----...........
Seq11 Sscrofa8 chromosome16	....----...........
Seq12 Sscrofa8 chromosomeX	....----...........
Seq13 Sscrofa8 chromosomeX	....----...........
Sus scrofa(AF435967)	....----...........
Sus scrofa(EF133960)	....----...........
Sus scrofa(AY437840)	....----...........
Sus scrofa(AJ279057)	....----...........
Sus scrofa(Y17013)	....----...........
Sus scrofa(AY056035)	....----...........
Sus scrofa(AF147808)	....----...........
Sus scrofa(EU523109)	....----...........
Sus scrofa(AF435966)	....----...........
Sus scrofa(AJ133816)	....----...........
Sus scrofa(AJ133818)	....----...........
Sus scrofa(AJ293657)	....----...........
Sus scrofa(AY099323)	....----...........
Sus scrofa(AJ133817)	....----...........
Sus scrofa(AY099324)	....----...........
Sus scrofa(AM229312)	....----...........
Sus scrofa(AM229311)	....----...........
Sus scrofa(DQ996272)	....----...........
Sus scrofa(AY570980)	....----...........
Sus scrofa(AY368583)	....A.............----...........
Sus scrofa(AY953542)	....----...........
Sus scrofa(AY437841)	....----...........
Sus scrofa(AY368585)	....----...........
Sus scrofa(AY368584)	....----...........
Sus scrofa(AY368586)	....----...........
Sus scrofa(AJ279056)	....----...........
Sus scrofa(EU789636)	....----...........
Sus scrofa(AJ293656)	....----...........
3 *Sus scrofa*	.C...----...........
6 *Sus scrofa*	..........T..----...........
9 *Sus scrofa*	....----...........
11 *Sus scrofa*	..........T......................T.....................----...........
3 *Sus barbatus barbatus*	............T.............T...............G............----.......-..T
4 *Sus barbatus barbatus*	...-............-......................................----..........A
9 *Sus barbatus barbatus*	....C.......----...........

```
10 Sus barbatus barbatus         .......................................................----..........A
2 Sus barbatus oi                ..........T............................................----...........
4 Sus barbatus oi                .......................A.................A.............----...........
11 Sus barbatus oi               .......................................................----...........
17 Sus barbatus oi               .......................................................----...........
2 Sus verrucosus                 .......................................................----...........
10 Sus verrucosus                ....................................C...A..............----...........
11 Sus verrucosus                .......................................................----...........
4 Sus celebensis                 .......................A.................A.............----...........
7 Sus celebensis                 ..........T............................................----...........
10 Sus celebensis                ..................................T.C...A...T..........----...........
2 Potamochoerus larvatus         .......................................................----...........
3 Potamochoerus larvatus         G......................................................----...........
4 Potamochoerus larvatus         .......................................................----...........
3 Potamochoerus porcus           .................................................G.....----...........
4 Potamochoerus porcus           .......................................................----...........
6 Potamochoerus porcus           ................A.........T.......A.......----------------------------
8 Potamochoerus porcus           ...........................A...........................----...........
5 Hylochoerus meinertzhageni     .......................................................----...........
9 Hylochoerus meinertzhageni     ...............................................C.......----...........
10 Hylochoerus meinertzhageni    ............T.........................................G----...........
1 Phacochoerus africanus         ..................G....................................----...........
4 Phacochoerus africanus         ..............................................A........----...........
6 Phacochoerus africanus         .......................................................----...........
10 Phacochoerus africanus        .......................................................----...........
11 Phacochoerus africanus        ..................G..............T.....................----...........
14 Phacochoerus africanus        .......................................................----...........
15 Phacochoerus africanus        ................................T......................----...........
5 Phacochoerus aethiopicus       ...................................................C...GTTC...........
14 Phacochoerus aethiopicus      ...T...................................................----...........
15 Phacochoerus aethiopicus      ......................................C..........T.....----...........

                                         780       790       800       810       820       830       840
                                 ....|....|....|....|....|....|....|....|....|....|....|....|....|....|
Seq1 Sscrofa8 chromosome1        AGCCTACTTGGGATGATTGTCAACAGCTGCTGCAGACACTCTTCACAACCGAGGAGCGAGAGAGAATTCT
Seq2 Sscrofa8 chromosome1        ......................................................................
Seq3 Sscrofa8 chromosome3        ......................................................................
Seq4 Sscrofa8 chromosome5        ...........A..........G..........................T...A.A..............
Seq5 Sscrofa8 chromosome7        ......................................................................
Seq6 Sscrofa8 chromosome8        ......................................................................
Seq7 Sscrofa8 chromosome13       .................................................T....................
Seq8 Sscrofa8 chromosome13       ......................G...............................................
Seq9 Sscrofa8 chromosome13       ......................................................................
Seq10 Sscrofa8 chromosome14      ......................................................................
Seq11 Sscrofa8 chromosome16      ......................................................................
Seq12 Sscrofa8 chromosomeX       ......................................................................
```

Seq13 Sscrofa8 chromosomeX	....
Sus scrofa(AF435967)	....
Sus scrofa(EF133960)	....**T**....................
Sus scrofa(AY437840)	....**G**..................
Sus scrofa(AJ279057)	....
Sus scrofa(Y17013)	....**G**......................
Sus scrofa(AY056035)	....
Sus scrofa(AF147808)	....
Sus scrofa(EU523109)	....
Sus scrofa(AF435966)	....
Sus scrofa(AJ133816)	....
Sus scrofa(AJ133818)	....
Sus scrofa(AJ293657)	....
Sus scrofa(AY099323)	....
Sus scrofa(AJ133817)	....
Sus scrofa(AY099324)	....
Sus scrofa(AM229312)	....
Sus scrofa(AM229311)	....
Sus scrofa(DQ996272)	....
Sus scrofa(AY570980)	....
Sus scrofa(AY368583)	....
Sus scrofa(AY953542)	....
Sus scrofa(AY437841)	....
Sus scrofa(AY368585)	....
Sus scrofa(AY368584)	....
Sus scrofa(AY368586)	....
Sus scrofa(AJ279056)	....
Sus scrofa(EU789636)	....
Sus scrofa(AJ293656)	....**A**..........
3 *Sus scrofa*	..........**T**...
6 *Sus scrofa*	....**G**........**T**.............
9 *Sus scrofa*	....
11 *Sus scrofa*	....
3 *Sus barbatus barbatus*	**T**........................**A**................**C**.............**T**.............
4 *Sus barbatus barbatus*	....
9 *Sus barbatus barbatus*	....
10 *Sus barbatus barbatus*	....
2 *Sus barbatus oi*	....
4 *Sus barbatus oi*	....
11 *Sus barbatus oi*	....
17 *Sus barbatus oi*	....
2 *Sus verrucosus*	....**C**............................
10 *Sus verrucosus*	......................**G**..........................**T**.....**A**..............
11 *Sus verrucosus*	....
4 *Sus celebensis*	....**C**...
7 *Sus celebensis*	....

```
10 Sus celebensis                 ......................G..........................T.....A..............
2 Potamochoerus larvatus          ...................................................................C..
3 Potamochoerus larvatus          ...................................................................C..
4 Potamochoerus larvatus          ...................................................................C..
3 Potamochoerus porcus            ......................................................................
4 Potamochoerus porcus            ......................................................................
6 Potamochoerus porcus            ---------------------------...G..G.GT...................T.........C...
8 Potamochoerus porcus            .A...................G................................................
5 Hylochoerus meinertzhageni      ........................................................T.............
9 Hylochoerus meinertzhageni      ........................................................T.............
10 Hylochoerus meinertzhageni     ..................-...................T...............................
1 Phacochoerus africanus          ......................................................................
4 Phacochoerus africanus          ......................................................................
6 Phacochoerus africanus          ......................................................................
10 Phacochoerus africanus         ......................................................................
11 Phacochoerus africanus         .....T....A.....C....G................................................
14 Phacochoerus africanus         ......................................................................
15 Phacochoerus africanus         ......................................................................
5 Phacochoerus aethiopicus        ................G.....................................................
14 Phacochoerus aethiopicus       ....C...................C......A..............................G.......
15 Phacochoerus aethiopicus       ....C.T...........................................A...................

                                            850       860       870       880       890       900       910
                                  ....|....|....|....|....|....|....|....|....|....|....|....|....|....|
Seq1 Sscrofa8 chromosome1         GTTAGAGGCT-----------AGAAAAAA-TGTTCCTGGGGCCGACGGGCGACCCACGCAGTTGCAAAAT
Seq2 Sscrofa8 chromosome1         ..........-----------........-........................................
Seq3 Sscrofa8 chromosome3         ..........-----------........-........................................
Seq4 Sscrofa8 chromosome5         ..........-----------........-...........................A...........C
Seq5 Sscrofa8 chromosome7         ..........-----------........-........................................
Seq6 Sscrofa8 chromosome8         ..........-----------........-........................................
Seq7 Sscrofa8 chromosome13        ..........-----------........-........................................
Seq8 Sscrofa8 chromosome13        ..........-----------........-........................................
Seq9 Sscrofa8 chromosome13        ..........-----------........-............T...........................
Seq10 Sscrofa8 chromosome14       A.........-----------........-.............................G..........
Seq11 Sscrofa8 chromosome16       ..........-----------........-........................................
Seq12 Sscrofa8 chromosomeX        ..........-----------........-........................................
Seq13 Sscrofa8 chromosomeX        ..........-----------........-........................................
Sus scrofa(AF435967)              ..........-----------........-........................................
Sus scrofa(EF133960)              ..........-----------........-........................................
Sus scrofa(AY437840)              ..........-----------........-........................................
Sus scrofa(AJ279057)              ..........-----------........-........................................
Sus scrofa(Y17013)                ..........-----------........-........................................
Sus scrofa(AY056035)              ..........-----------........-........................................
Sus scrofa(AF147808)              ..........-----------........-........................................
Sus scrofa(EU523109)              ..........-----------........-........................................
Sus scrofa(AF435966)              ..........-----------........-........................................
```

Sus scrofa(AJ133816)	..........-----------........-..
Sus scrofa(AJ133818)	..........-----------........-..
Sus scrofa(AJ293657)	..........-----------........-..
Sus scrofa(AY099323)	..........-----------........-..
Sus scrofa(AJ133817)	..........-----------........-..
Sus scrofa(AY099324)	..........-----------........-..
Sus scrofa(AM229312)	**A**.........-----------........-.............................**G**..........
Sus scrofa(AM229311)	**A**.........-----------........-.............................**G**..........
Sus scrofa(DQ996272)	**A**.........-----------........-.............................**G**..........
Sus scrofa(AY570980)	**A**.........-----------........-..
Sus scrofa(AY368583)	..........-----------........-..
Sus scrofa(AY953542)	..........-----------........-..
Sus scrofa(AY437841)	..........-----------........-..........**A**.............................
Sus scrofa(AY368585)	..........-----------........-..
Sus scrofa(AY368584)	..........-----------........-..
Sus scrofa(AY368586)	..........-----------........-..
Sus scrofa(AJ279056)	..........-----------........-..
Sus scrofa(EU789636)	..........-----------........-..
Sus scrofa(AJ293656)	..........-----------........-..
3 *Sus scrofa*	..........-----------........-...............................-........
6 *Sus scrofa*	.......**A**..-----------....**G**...-...................**T**...........-........
9 *Sus scrofa*	..........-----------........-...............................-....**G**...
11 *Sus scrofa*	..........-----------........-..
3 *Sus barbatus barbatus*	..........**TAAAAAAAAAA**.**A**......**A**...........................**A**...-...**G**....
4 *Sus barbatus barbatus*	..........-----------........-................**T**..**T**...........-........
9 *Sus barbatus barbatus*	..........-----------........-...............................-........
10 *Sus barbatus barbatus*	..........-----------........-................**T**..**T**...........-........
2 *Sus barbatus oi*	..........-----------...**G**....-...............................-........
4 *Sus barbatus oi*	..........-----------........-...............................-.......-
11 *Sus barbatus oi*	..........-----------........-...........................**A**...-........
17 *Sus barbatus oi*	..........-----------........-...............................-........
2 *Sus verrucosus*	..........-----------........-....................**A**..........-........
10 *Sus verrucosus*	..........-----------........-.............................**G**.-........
11 *Sus verrucosus*	..........-----------........-...............................-........
4 *Sus celebensis*	..........-----------........-..
7 *Sus celebensis*	..........-----------...**G**....-...............................-........
10 *Sus celebensis*	..........-----------.**A**......-..........**T**....................-........
2 *Potamochoerus larvatus*	**A**.....**A**...-----------........**G**...................**T**...........-........
3 *Potamochoerus larvatus*	**A**.....**A**...-----------........-...........................**A**...-........
4 *Potamochoerus larvatus*	**A**.....**A**...-----------........-...........................**A**...-........
3 *Potamochoerus porcus*	..........-----------........-............**T**.......**A**..........-........
4 *Potamochoerus porcus*	..........-----------........-...............................-........
6 *Potamochoerus porcus*	**A**.........-----------........-...............**T**...**T**......**A**....-........
8 *Potamochoerus porcus*	..........-----------........**G**...............................-.**A**......
5 *Hylochoerus meinertzhageni*	..........-----------........-............**T**..................-........
9 *Hylochoerus meinertzhageni*	..........-----------........-............**T**..................-........

10 *Hylochoerus meinertzhageni*	..........-----------........-....**T**.......**T**......**T**...........-........
1 *Phacochoerus africanus*	..........-----------........**A**...............................-........
4 *Phacochoerus africanus*	..........-----------........-...................**T**...........-........
6 *Phacochoerus africanus*	..........-----------........-...............................-........
10 *Phacochoerus africanus*	..........-----------........-...............................-........
11 *Phacochoerus africanus*	..........-----------........**A**...............................-........
14 *Phacochoerus africanus*	..........-----------........-...............................-........
15 *Phacochoerus africanus*	..........-----------........-...............................-........
5 *Phacochoerus aethiopicus*	..........-----------........-...............................-........
14 *Phacochoerus aethiopicus*	........**T**.-----------........-..........**A**....**T**....**A**..........-........
15 *Phacochoerus aethiopicus*	..........-----------........-.........................**GT**....-........

	....|....
Seq1 Sscrofa8 chromosome1	**GAGATTGAC**
Seq2 Sscrofa8 chromosome1	....
Seq3 Sscrofa8 chromosome3	....
Seq4 Sscrofa8 chromosome5	..**A**......
Seq5 Sscrofa8 chromosome7	....
Seq6 Sscrofa8 chromosome8	....
Seq7 Sscrofa8 chromosome13	....
Seq8 Sscrofa8 chromosome13	....
Seq9 Sscrofa8 chromosome13	....
Seq10 Sscrofa8 chromosome14	....
Seq11 Sscrofa8 chromosome16	....
Seq12 Sscrofa8 chromosomeX	..**A**......
Seq13 Sscrofa8 chromosomeX	....
Sus scrofa(AF435967)	....
Sus scrofa(EF133960)	....
Sus scrofa(AY437840)	....
Sus scrofa(AJ279057)	....
Sus scrofa(Y17013)	....
Sus scrofa(AY056035)	....
Sus scrofa(AF147808)	....
Sus scrofa(EU523109)	....
Sus scrofa(AF435966)	....
Sus scrofa(AJ133816)	....
Sus scrofa(AJ133818)	....
Sus scrofa(AJ293657)	....
Sus scrofa(AY099323)	....
Sus scrofa(AJ133817)	....
Sus scrofa(AY099324)	....
Sus scrofa(AM229312)	....
Sus scrofa(AM229311)	....
Sus scrofa(DQ996272)	....
Sus scrofa(AY570980)	....

Sus scrofa(AY368583)	....
Sus scrofa(AY953542)	....
Sus scrofa(AY437841)	....
Sus scrofa(AY368585)	....
Sus scrofa(AY368584)	....
Sus scrofa(AY368586)	....
Sus scrofa(AJ279056)	....
Sus scrofa(EU789636)	....
Sus scrofa(AJ293656)	....
3 *Sus scrofa*	....-....
6 *Sus scrofa*	....-....
9 *Sus scrofa*	....-....
11 *Sus scrofa*	....
3 *Sus barbatus barbatus*	....-....
4 *Sus barbatus barbatus*	....-....
9 *Sus barbatus barbatus*	**A**...-....
10 *Sus barbatus barbatus*	...**G**-....
2 *Sus barbatus oi*	....-....
4 *Sus barbatus oi*	---------
11 *Sus barbatus oi*	....-....
17 *Sus barbatus oi*	....-....
2 *Sus verrucosus*	....-....
10 *Sus verrucosus*	....-....
11 *Sus verrucosus*	....-....
4 *Sus celebensis*	....
7 *Sus celebensis*	....-....
10 *Sus celebensis*	....-....
2 *Potamochoerus larvatus*	....-....
3 *Potamochoerus larvatus*	....-....
4 *Potamochoerus larvatus*	....-....
3 *Potamochoerus porcus*	....-....
4 *Potamochoerus porcus*	....-....
6 *Potamochoerus porcus*	....-....
8 *Potamochoerus porcus*	....-....
5 *Hylochoerus meinertzhageni*	....-....
9 *Hylochoerus meinertzhageni*	....-....
10 *Hylochoerus meinertzhageni*	....-....
1 *Phacochoerus africanus*	....-....
4 *Phacochoerus africanus*	....-....
6 *Phacochoerus africanus*	....-....
10 *Phacochoerus africanus*	....-....
11 *Phacochoerus africanus*	....-....
14 *Phacochoerus africanus*	....-....
15 *Phacochoerus africanus*	....-....
5 *Phacochoerus aethiopicus*	....-....
14 *Phacochoerus aethiopicus*	....-....

15 *Phacochoerus aethiopicus* .....**C**...
