# Supplementary material for: Evolution of endogenous retroviruses in the Suidae: evidence for different viral subpopulations in African and Eurasian host species
Source: BMC Evol Biol. 2011 May 24;11:139. doi: 10.1186/1471-2148-11-139 (PMC3128044; doi:10.1186/1471-2148-11-139)
Supplement: Additional file 3 — pol alignment. pol alignment of sequences generated in this study, sequences from GenBank and the draft pig genome [file 1471-2148-11-139-S3.PDF]

<u>*pol* alignment (including all sequences)</u>

The first number next to some sequence names represents the clone number.

```
                                            10        20        30        40        50        60        70
                                    ....|....|....|....|....|....|....|....|....|....|....|....|....|....|
Seq1 Sscrofa8 chromosome1           ATTTGATGCTATCAAAAAGGCCCTGCTGAGCGCACCTGCTCTGGCCCTCCCTGACGTGACTAAACCCTTT
Seq2 Sscrofa8 chromosome3           .......................................C..............T..A............
Seq3 Sscrofa8 chromosome3           .........................................................A............
Seq4 Sscrofa8 chromosome8           ......................................................................
Seq5 Sscrofa8 chromosome9           .........................................................A............
Seq6 Sscrofa8 chromosome17          ......................................................T..A........T...
Seq7 Sscrofa8 chromosome17          .................................G....................T..A......A.T...
Seq8 Sscrofa8 chromosome16          .........................................................A............
Sus scrofa(AF435966)                .........................TC...............................CTC.........
Sus scrofa(AJ293656)                ......................................................................
Sus scrofa(AY099323)                .........................................................A............
Sus scrofa(AJ133817)                .........................................................A............
Sus scrofa(AF435967)                ................................................................------
Sus scrofa(AY099324)                .........................................................A............
Sus scrofa(AJ293657)                .........................................................A............
Sus scrofa(AJ133818)                .........................................................A............
Sus scrofa(AJ133816)                .........................................................A............
Sus scrofa(AM229312)                .........................................................A............
Sus scrofa(AM229311)                .........................................................A............
Sus scrofa(AM229313)                .........................................................A............
Sus scrofa(AY953542)                .........................................................A............
Sus scrofa(AY570980)                .........................................................A............
Sus scrofa(AJ279056)                ......................................................................
Sus scrofa(AJ279057)                .........................................................A............
Sus scrofa(AY056035)                .........................................................A............
Sus scrofa(Y17013)                  .........................................................A............
Sus scrofa(EU523109)                .........................................................A............
Sus scrofa(EF133960)                ......................................................T..A........T...
Sus scrofa(DQ996273)                .........................................................A............
6 Sus scrofa                        ......................................................T..A............
9 Sus scrofa                        ......................................................................
11 Sus scrofa                       ......................................................................
14 Sus scrofa                       ......................................................T..A............
1 Sus barbatus barbatus             .........................................................A............
2 Sus barbatus barbatus             ......................................................................
3 Sus barbatus barbatus             ......................................................................
5 Sus barbatus oi                   .....................................................C...A............
6 Sus barbatus oi                   ......................................................................
8 Sus barbatus oi                   .....................................................C...A............
9 Sus barbatus oi                   .....................................................C...A............
```

```
3 Sus celebensis                 .........................................................A............
6 Sus celebensis                 ......................................................................
8 Sus celebensis                 .........................................................A............
2 Potamochoerus larvatus         .........C...............................................A............
3 Potamochoerus larvatus         ...............................T......................T..A............
M3 Potamochoerus larvatus        .........C...............................................A............
4 Potamochoerus larvatus         .........C...............................................A............
4 Potamochoerus porcus           ......................................................T..A............
5 Potamochoerus porcus           ......................................................T..A............
6 Potamochoerus porcus           ......................................................T..A..........G.
9 Potamochoerus porcus           ............T.................T.......................T..A............
1 Phacochoerus africanus         G...........T.........................................T..A............
5 Phacochoerus africanus         G...........T.....................T...................T..A............
9 Phacochoerus africanus         G........C..T......................................G..T..A............
11 Phacochoerus africanus        G...........T.....................T...................T..A............
12 Phacochoerus africanus        G...........T.....................T...................T..A............
14 Phacochoerus africanus        G...........T.........................................T..A............
16 Phacochoerus africanus        G...........T.........................................T..A............
2 Phacochoerus aethiopicus       ............T.........................................T..A............
9 Phacochoerus aethiopicus       ............T.........................................T..A............
16 Phacochoerus aethiopicus      G...........T.........................................T..A............
17 Phacochoerus aethiopicus      G...........T.........................................T..A............

                                          80        90        100       110       120       130       140
                                 ....|....|....|....|....|....|....|....|....|....|....|....|....|....|
Seq1 Sscrofa8 chromosome1        ACCCTTTATGTGGATGAGCGTAAGGGAGTAGCCCGGGGAGTTTTAACCCAAACTCTAGGACCATGGAGGA
Seq2 Sscrofa8 chromosome3        ...................A......G........................T.C................
Seq3 Sscrofa8 chromosome3        ...................................A.................C................
Seq4 Sscrofa8 chromosome8        .....................................................C................
Seq5 Sscrofa8 chromosome9        ...................................A.................C................
Seq6 Sscrofa8 chromosome17       .....................................................C..............A.
Seq7 Sscrofa8 chromosome17       ...............................................A.....C..............A.
Seq8 Sscrofa8 chromosome16       ...................................A.................C................
Sus scrofa(AF435966)             ....................................................T.................
Sus scrofa(AJ293656)             ......................................................................
Sus scrofa(AY099323)             ...................................A.................C................
Sus scrofa(AJ133817)             ...................................A.................C................
Sus scrofa(AF435967)             ......................................................................
Sus scrofa(AY099324)             ...................................A.................C................
Sus scrofa(AJ293657)             ...................................A.................C................
Sus scrofa(AJ133818)             ...................................A.................C................
Sus scrofa(AJ133816)             ...................................A.................C................
Sus scrofa(AM229312)             .....................................................C..............A.
Sus scrofa(AM229311)             .....................................................C..............A.
Sus scrofa(AM229313)             .....................................................C..............A.
Sus scrofa(AY953542)             .....................................................C................
```

```
Sus scrofa(AY570980)                ..................................................
Sus scrofa(AJ279056)                ......................................................................
```

```
                                              150       160       170       180       190       200       210
                                    ....|....|....|....|....|....|....|....|....|....|....|....|....|....|
```

Seq3 Sscrofa8 chromosome3	............................T........................G................
Seq4 Sscrofa8 chromosome8	....G................
Seq5 Sscrofa8 chromosome9	............................T........................G................
Seq6 Sscrofa8 chromosome17	...................................A..................................
Seq7 Sscrofa8 chromosome17	...................................A.................G................
Seq8 Sscrofa8 chromosome16	............................T........................G................
Sus scrofa(AF435966)	....
Sus scrofa(AJ293656)	....
Sus scrofa(AY099323)	............................T........................G....T...........
Sus scrofa(AJ133817)	............................T........................G....T...........
Sus scrofa(AF435967)	....
Sus scrofa(AY099324)	............................T........................G....T...........
Sus scrofa(AJ293657)	............................T........................G................
Sus scrofa(AJ133818)	............................T........................G................
Sus scrofa(AJ133816)	............................T........................G................
Sus scrofa(AM229312)	.......C..
Sus scrofa(AM229311)	.......C..
Sus scrofa(AM229313)	.......C..
Sus scrofa(AY953542)	....G................
Sus scrofa(AY570980)	.......C..
Sus scrofa(AJ279056)	....G................
Sus scrofa(AJ279057)	............................T...
Sus scrofa(AY056035)	............................T........................G................
Sus scrofa(Y17013)	............................T........................G................
Sus scrofa(EU523109)	............................T........................G................
Sus scrofa(EF133960)	...................................A.................G................
Sus scrofa(DQ996273)	.......C..
6 *Sus scrofa*	....G....T...........
9 *Sus scrofa*	....G................
11 *Sus scrofa*	....G................
14 *Sus scrofa*	....
1 *Sus barbatus barbatus*	....G................
2 *Sus barbatus barbatus*	....
3 *Sus barbatus barbatus*	....
5 *Sus barbatus oi*	....G................
6 *Sus barbatus oi*	..........T..G................
8 *Sus barbatus oi*	....G................
9 *Sus barbatus oi*	....G................
3 *Sus celebensis*	....G................
6 *Sus celebensis*	............................T........................G................
8 *Sus celebensis*	....G................
2 *Potamochoerus larvatus*	..........T........T.....A..
3 *Potamochoerus larvatus*	.............................A.............G........TG................
M3 *Potamochoerus larvatus*	..........T........T.....A..
4 *Potamochoerus larvatus*	..........T........T.....A..
4 *Potamochoerus porcus*	.............................A.......................G................

```
5 Potamochoerus porcus        .............................A.......................G................
6 Potamochoerus porcus        .............................A......C................G................
9 Potamochoerus porcus        .........T...........................................G................
1 Phacochoerus africanus      ....................................................TG................
5 Phacochoerus africanus      ....................................................TG................
9 Phacochoerus africanus      ....................................................TG................
11 Phacochoerus africanus     ....................................................TG................
12 Phacochoerus africanus     ....................................................TG................
14 Phacochoerus africanus     ....................................................TG................
16 Phacochoerus africanus     ....................................................TG................
2 Phacochoerus aethiopicus    ....................................................TG................
9 Phacochoerus aethiopicus    ....................................................TG................
16 Phacochoerus aethiopicus   ....................................................TG................
17 Phacochoerus aethiopicus   ....................................................TG................

                                      220       230       240       250       260       270       280
                              ....|....|....|....|....|....|....|....|....|....|....|....|....|....|
Seq1 Sscrofa8 chromosome1     CGCAGCTGTGGCCATACTGGTCAAGGACGCTGACAAATTGACTTTGGGACAGAATATAACTGTAATAGCC
Seq2 Sscrofa8 chromosome3     ......................................................................
Seq3 Sscrofa8 chromosome3     ......................................................................
Seq4 Sscrofa8 chromosome8     ......................................................................
Seq5 Sscrofa8 chromosome9     ......................................................................
Seq6 Sscrofa8 chromosome17    .............................................................A........
Seq7 Sscrofa8 chromosome17    .............................................................A........
Seq8 Sscrofa8 chromosome16    ..............................................A.......................
Sus scrofa(AF435966)          ......................................................................
Sus scrofa(AJ293656)          ......................................................................
Sus scrofa(AY099323)          ......................................................................
Sus scrofa(AJ133817)          ......................................................................
Sus scrofa(AF435967)          ......................................................................
Sus scrofa(AY099324)          ......................................................................
Sus scrofa(AJ293657)          ......................................................................
Sus scrofa(AJ133818)          ......................................................................
Sus scrofa(AJ133816)          ......................................................................
Sus scrofa(AM229312)          ......................................................................
Sus scrofa(AM229311)          ......................................................................
Sus scrofa(AM229313)          ......................................................................
Sus scrofa(AY953542)          ......................................................................
Sus scrofa(AY570980)          ......................................................................
Sus scrofa(AJ279056)          ......................................................................
Sus scrofa(AJ279057)          ......................................................................
Sus scrofa(AY056035)          ..............................................A.......................
Sus scrofa(Y17013)            ..........................................C...........................
Sus scrofa(EU523109)          ......................................................................
Sus scrofa(EF133960)          .............................................................A........
Sus scrofa(DQ996273)          ......................................................................
```

6 *Sus scrofa*	....
9 *Sus scrofa*	....
11 *Sus scrofa*	.............**T**..
14 *Sus scrofa*	.......**C**..
1 *Sus barbatus barbatus*	**T**...**C**.........
2 *Sus barbatus barbatus*	....**G**...........
3 *Sus barbatus barbatus*	....**G**...........
5 *Sus barbatus oi*	....
6 *Sus barbatus oi*	....
8 *Sus barbatus oi*	....
9 *Sus barbatus oi*	....
3 *Sus celebensis*	....
6 *Sus celebensis*	....
8 *Sus celebensis*	....
2 *Potamochoerus larvatus*	................................**G**.....................................
3 *Potamochoerus larvatus*	....**A**.......**T**
M3 *Potamochoerus larvatus*	....
4 *Potamochoerus larvatus*	....
4 *Potamochoerus porcus*	....**A**.......**T**
5 *Potamochoerus porcus*	....**A**.......**T**
6 *Potamochoerus porcus*	....**A**.......**T**
9 *Potamochoerus porcus*	.......**A**...**A**........
1 *Phacochoerus africanus*	...........................**T**..........................**A**....**T**.**A**........
5 *Phacochoerus africanus*	....**A**.......**A**......**A**........
9 *Phacochoerus africanus*	....**A**........
11 *Phacochoerus africanus*	....**A**.......**A**......**A**....**G**...
12 *Phacochoerus africanus*	....**A**.......**A**......**A**........
14 *Phacochoerus africanus*	...........................**T**..........................**A**....**T**.**A**........
16 *Phacochoerus africanus*	...........................**T**..........................**A**......**A**........
2 *Phacochoerus aethiopicus*	....**A**..**G**.....
9 *Phacochoerus aethiopicus*	....**A**..**G**.....
16 *Phacochoerus aethiopicus*	...........................**T**..........................**A**......**A**........
17 *Phacochoerus aethiopicus*	........**C**..................**T**..........................**A**......**A**........
	290 300 310 320 330 340 350
	....|....|....|....|....|....|....|....|....|....|....|....|....|....|
Seq1 Sscrofa8 chromosome1	**CCCCATGCGTTGGAGAACATCGTTCGGCAGCCCCCAGACCGATGGATGACCAACGCCCGCATGACCCACT**
Seq2 Sscrofa8 chromosome3	.......................**C**..
Seq3 Sscrofa8 chromosome3	........**A**..**T**................
Seq4 Sscrofa8 chromosome8	....
Seq5 Sscrofa8 chromosome9	........**A**...
Seq6 Sscrofa8 chromosome17	..............**A**........**C**................................**T**.............
Seq7 Sscrofa8 chromosome17	..............**A**........**C**................................**T**.............
Seq8 Sscrofa8 chromosome16	........**A**...
Sus scrofa(AF435966)	....
Sus scrofa(AJ293656)	....

Sus scrofa(AY099323)	........A...
Sus scrofa(AJ133817)	........A...
Sus scrofa(AF435967)	....
Sus scrofa(AY099324)	........A...
Sus scrofa(AJ293657)	........A...
Sus scrofa(AJ133818)	........A...
Sus scrofa(AJ133816)	........A...
Sus scrofa(AM229312)	........A...
Sus scrofa(AM229311)	........A...
Sus scrofa(AM229313)	........A...
Sus scrofa(AY953542)	....
Sus scrofa(AY570980)	........A...
Sus scrofa(AJ279056)	....
Sus scrofa(AJ279057)	....
Sus scrofa(AY056035)	........A...
Sus scrofa(Y17013)	........A...
Sus scrofa(EU523109)	........A...
Sus scrofa(EF133960)	........A.....A........C................................T...........G.
Sus scrofa(DQ996273)	........A...
6 *Sus scrofa*	........A...
9 *Sus scrofa*	........A...
11 *Sus scrofa*	....
14 *Sus scrofa*	..............A........................T..............................
1 *Sus barbatus barbatus*	.......T...T.T.........G..
2 *Sus barbatus barbatus*	....................T...
3 *Sus barbatus barbatus*	....................T...
5 *Sus barbatus oi*	........A................C.............T..............................
6 *Sus barbatus oi*	.......................C..
8 *Sus barbatus oi*	........A................C.............T..............................
9 *Sus barbatus oi*	....A.............................
3 *Sus celebensis*	....
6 *Sus celebensis*	....
8 *Sus celebensis*	........A...
2 *Potamochoerus larvatus*	.......................C..
3 *Potamochoerus larvatus*	.......T......A........C.................................A............
M3 *Potamochoerus larvatus*	.......................C..
4 *Potamochoerus larvatus*	.......................C..
4 *Potamochoerus porcus*	.......T......A........GT................................A............
5 *Potamochoerus porcus*	.......T......A........GT................................A............
6 *Potamochoerus porcus*	.......T......A........GT..................A.............A............
9 *Potamochoerus porcus*	........A..............C..
1 *Phacochoerus africanus*	.......................C..
5 *Phacochoerus africanus*	.......................C..
9 *Phacochoerus africanus*	.......................C.A..
11 *Phacochoerus africanus*	.......................C..
12 *Phacochoerus africanus*	.......................C..

```
14 Phacochoerus africanus       .......................C..............................................
16 Phacochoerus africanus       .......................C..............................................
2 Phacochoerus aethiopicus      .....................A.C..............................................
9 Phacochoerus aethiopicus      .....................A.C..............................................
16 Phacochoerus aethiopicus     .......................C...........................T..................
17 Phacochoerus aethiopicus     .......................C..............................................

                                         360       370       380       390       400       410       420
                                ....|....|....|....|....|....|....|....|....|....|....|....|....|....|
Seq1 Sscrofa8 chromosome1       ATCAAAGCCTGCTTCTCACAGAGAGGGTCACGTTCGCTCCACCAGCCGCTCTCAACCCTGCCACTCTTCT
Seq2 Sscrofa8 chromosome3       ......................A...A................C..........................
Seq3 Sscrofa8 chromosome3       ...............................T......................................
Seq4 Sscrofa8 chromosome8       ......................................................................
Seq5 Sscrofa8 chromosome9       ...............................T......................................
Seq6 Sscrofa8 chromosome17      ..........................A.......T...................................
Seq7 Sscrofa8 chromosome17      ..........................A.......T...................................
Seq8 Sscrofa8 chromosome16      ...............................T..T...................................
Sus scrofa(AF435966)            ......................................................................
Sus scrofa(AJ293656)            ......................................................................
Sus scrofa(AY099323)            ...............................T......................................
Sus scrofa(AJ133817)            ...............................T......................................
Sus scrofa(AF435967)            ......................................................................
Sus scrofa(AY099324)            ...............................T......................................
Sus scrofa(AJ293657)            ...............................T......................................
Sus scrofa(AJ133818)            ...............................T......................................
Sus scrofa(AJ133816)            ...............................T......................................
Sus scrofa(AM229312)            ......................................................................
Sus scrofa(AM229311)            ......................................................................
Sus scrofa(AM229313)            ......................................................................
Sus scrofa(AY953542)            ......................................................................
Sus scrofa(AY570980)            ......................................................................
Sus scrofa(AJ279056)            ......................................................................
Sus scrofa(AJ279057)            ......................................................................
Sus scrofa(AY056035)            ...............................T..T...................................
Sus scrofa(Y17013)              ...............................T......................................
Sus scrofa(EU523109)            ...............................T......................................
Sus scrofa(EF133960)            ..........................A.......T...................................
Sus scrofa(DQ996273)            ......................................................................
6 Sus scrofa                    ..................................T...................................
9 Sus scrofa                    ......................................................................
11 Sus scrofa                   .........................A............................................
14 Sus scrofa                   ...............................................A......................
1 Sus barbatus barbatus         ..........................................G...........................
2 Sus barbatus barbatus         .........................A............................................
3 Sus barbatus barbatus         .........................A............................................
5 Sus barbatus oi               ......................................................................
```

```
6 Sus barbatus oi            ......................................................................
8 Sus barbatus oi            ......................................................................
9 Sus barbatus oi            ..........A......................................C.....T..............
3 Sus celebensis             ......................................................................
6 Sus celebensis             .....C................................................................
8 Sus celebensis             ......................................................................
2 Potamochoerus larvatus     ..........................A...........................................
3 Potamochoerus larvatus     ...................................................C..................
M3 Potamochoerus larvatus    ..........................A...........................................
4 Potamochoerus larvatus     ..........................A...........................................
4 Potamochoerus porcus       ......................C...A...T......................C................
5 Potamochoerus porcus       ......................C...A...T......................C................
6 Potamochoerus porcus       .........C..............A.A...T.......................................
9 Potamochoerus porcus       ......................................................................
1 Phacochoerus africanus     ......................A...............................................
5 Phacochoerus africanus     ..........................A......................................T....
9 Phacochoerus africanus     ......................A...A.......T............A......................
11 Phacochoerus africanus    ..........................A......................................T....
12 Phacochoerus africanus    ......................A...............................................
14 Phacochoerus africanus    ..........................A......................................T....
16 Phacochoerus africanus    ......................A...............................................
2 Phacochoerus aethiopicus   ......................A...A................C..T..C....................
9 Phacochoerus aethiopicus   ......................A...A................C..T..C....................
16 Phacochoerus aethiopicus  ......................A..........................C....................
17 Phacochoerus aethiopicus  ......................A..........................C....................

                                     430       440       450       460       470       480       490
                             ....|....|....|....|....|....|....|....|....|....|....|....|....|....|
Seq1 Sscrofa8 chromosome1    GCCTGAAGAGACTGATGAACCAGTGACTCATGATTGCCATCAACTATTGATTGAGGAGACTGGGGTCCGC
Seq2 Sscrofa8 chromosome3    ............C................................G.....C..................
Seq3 Sscrofa8 chromosome3    ......................................................................
Seq4 Sscrofa8 chromosome8    ......................................................................
Seq5 Sscrofa8 chromosome9    ......................................................................
Seq6 Sscrofa8 chromosome17   .............................................G.....C..................
Seq7 Sscrofa8 chromosome17   .............................................G.....C..................
Seq8 Sscrofa8 chromosome16   ......................................................................
Sus scrofa(AF435966)         .........................................---..........................
Sus scrofa(AJ293656)         ......................................................................
Sus scrofa(AY099323)         ......................................................................
Sus scrofa(AJ133817)         ......................................................................
Sus scrofa(AF435967)         ......................C...............................................
Sus scrofa(AY099324)         ......................................................................
Sus scrofa(AJ293657)         ......................................................................
Sus scrofa(AJ133818)         ......................................................................
Sus scrofa(AJ133816)         ......................................................................
Sus scrofa(AM229312)         ......................................................................
```

```
Sus scrofa(AM229311)            ......................................................................
Sus scrofa(AM229313)            ......................................................................
Sus scrofa(AY953542)            ......................................................................
Sus scrofa(AY570980)            ......................................................................
Sus scrofa(AJ279056)            .....................................................................A
Sus scrofa(AJ279057)            ......................................................................
Sus scrofa(AY056035)            ......................................................................
Sus scrofa(Y17013)              ......................................................................
Sus scrofa(EU523109)            ......................................................................
Sus scrofa(EF133960)            ...................................................C..................
Sus scrofa(DQ996273)            ......................................................................
6 Sus scrofa                    ......................................................................
9 Sus scrofa                    ......................................................................
11 Sus scrofa                   ......................................................................
14 Sus scrofa                   ......................................................................
1 Sus barbatus barbatus         .............................................G..A..C..................
2 Sus barbatus barbatus         ......................................................................
3 Sus barbatus barbatus         ......................................................................
5 Sus barbatus oi               ...................................................................T..
6 Sus barbatus oi               ......................................................................
8 Sus barbatus oi               ...................................................................T..
9 Sus barbatus oi               ......................................................................
3 Sus celebensis                ........G.............................................................
6 Sus celebensis                ..........G...........................................................
8 Sus celebensis                ....................................................................A.
2 Potamochoerus larvatus        ......................................................................
3 Potamochoerus larvatus        ...........................................T.G....C...................
M3 Potamochoerus larvatus       ......................................................................
4 Potamochoerus larvatus        ......................................................................
4 Potamochoerus porcus          ...........................................T.G.....................T..
5 Potamochoerus porcus          ...........................................T.G.....................T..
6 Potamochoerus porcus          ...........................................T.G.........A...........T..
9 Potamochoerus porcus          ............C..............................T.G........................
1 Phacochoerus africanus        ............C..............................T.G...........A............
5 Phacochoerus africanus        ......................................................................
9 Phacochoerus africanus        .................................................G....................
11 Phacochoerus africanus       ......................................................................
12 Phacochoerus africanus       ............C..............................T.G...........A............
14 Phacochoerus africanus       ......................................................................
16 Phacochoerus africanus       ............C.....G........................T.G...........A............
2 Phacochoerus aethiopicus      .............................................G.....C..................
9 Phacochoerus aethiopicus      .............................................G.....C..................
16 Phacochoerus aethiopicus     ............C..............................T.G...........A............
17 Phacochoerus aethiopicus     ............C..............................T.G...........A............

                                         500       510       520       530       540       550       560
```

```
                              ....|....|....|....|....|....|....|....|....|....|....|....|....|....|
Seq1 Sscrofa8 chromosome1     AAGGACCTTACAGACATA---CCGCTGACTGGAGAAATGTTAACCTGGTTCACTGACGGAAGCAGCTATG
Seq2 Sscrofa8 chromosome3     ..................---...............G..C.G..............T..G..........
Seq3 Sscrofa8 chromosome3     ..................---...............G..C..............................
Seq4 Sscrofa8 chromosome8     ..................---...............G..C..............................
Seq5 Sscrofa8 chromosome9     ..................---...............G..C..............................
Seq6 Sscrofa8 chromosome17    ..........T.......---..A............G..C.G..............T..G..........
Seq7 Sscrofa8 chromosome17    ..........T.......---..A............G..C.G..............T..G..........
Seq8 Sscrofa8 chromosome16    ..................---...............G..C..............................
Sus scrofa(AF435966)          ..................CCG............A....................................
Sus scrofa(AJ293656)          ..................---................................................A
Sus scrofa(AY099323)          ..................---...............G..C..............................
Sus scrofa(AJ133817)          ..................---...............G..C..............................
Sus scrofa(AF435967)          ..................---...............CC.G.............................C
Sus scrofa(AY099324)          ..................---...............G..C..............................
Sus scrofa(AJ293657)          ..................---...............G..C..............................
Sus scrofa(AJ133818)          ..................---...............G..C..............................
Sus scrofa(AJ133816)          ..................---...............G..C..............................
Sus scrofa(AM229312)          ..................---...............G..C..............................
Sus scrofa(AM229311)          ..................---...............G..C..............................
Sus scrofa(AM229313)          ..................---...............G..C..............................
Sus scrofa(AY953542)          ..................---...............G.................................
Sus scrofa(AY570980)          ..................---...............G..C..............................
Sus scrofa(AJ279056)          ..................---...............G.................................
Sus scrofa(AJ279057)          ..................---...............G..C..............................
Sus scrofa(AY056035)          ..................---...............G..C..............................
Sus scrofa(Y17013)            ..................---...............G..C..............................
Sus scrofa(EU523109)          ..................---...............G..C..............................
Sus scrofa(EF133960)          ..........T.......---...............G..C.G..............T..G..........
Sus scrofa(DQ996273)          ..................---...............G..C..............................
6 Sus scrofa                  ..................---...............G..C..............................
9 Sus scrofa                  ..................---...............G................C................
11 Sus scrofa                 ..................---...............G..C..............................
14 Sus scrofa                 ..................---...............G..C................T....A..A.....
1 Sus barbatus barbatus       ..................---...............G..C.G............................
2 Sus barbatus barbatus       ..................---...............G..C..............................
3 Sus barbatus barbatus       ..................---...............G..C..............................
5 Sus barbatus oi             ..................---...............G..C..............................
6 Sus barbatus oi             ..................---...............G..C..............................
8 Sus barbatus oi             ..................---...............G..C..............................
9 Sus barbatus oi             ..................---.............G.G..C..............................
3 Sus celebensis              ..................---...............G..C..............................
6 Sus celebensis              ..................---...............G..C.............C................
8 Sus celebensis              ..................---...............G..C..............................
2 Potamochoerus larvatus      ..................---...........G...G..C..............................
3 Potamochoerus larvatus      .....T...........G---...............G..C.G..............T..G..........
```

```
M3 Potamochoerus larvatus          ..................---...........G...G..C..............................
4 Potamochoerus larvatus           ..................---...........G...G..C
4 Potamochoerus porcus             .....T............---...............G..C.G..............T..G.A..A.....
5 Potamochoerus porcus             .....T............---...............G..C.G..............T..G.A..A.....
6 Potamochoerus porcus             .....T............---...............G..C.G..............G..G.C........
9 Potamochoerus porcus             .....T............---...............G..C.G..............T..G..........
1 Phacochoerus africanus           .....T............---...............G..C.G..............T..G..........
5 Phacochoerus africanus           ..................---..C............G..C.G.....T........T..G..........
9 Phacochoerus africanus           ..................---..C............G..C.G..............T..G.........A
11 Phacochoerus africanus          ..................---..C............G..C.G.....T........T..G..........
12 Phacochoerus africanus          .....T............---...............G..C.G..............T..G..........
14 Phacochoerus africanus          ..................---..C............G..C.G.....T........T..G..........
16 Phacochoerus africanus          .....T............---...............G..C.G.....T........T..G..........
2 Phacochoerus aethiopicus         ..................---...............G..C.G..............T..G..........
9 Phacochoerus aethiopicus         ..................---...............G..C.G..............T..G..........
16 Phacochoerus aethiopicus        ..................---...............G..C.G..............T..G..........
17 Phacochoerus aethiopicus        .....T............---...............G..C.G..............T..G..........

                                            570       580       590       600       610       620       630
                                   ....|....|....|....|....|....|....|....|....|....|....|....|....|....|
Seq1 Sscrofa8 chromosome1          TGGTGGAAGGTAAGAGGATGGCTGGGGCGGCGGTGGTGGACGGGA-CCCGCACGATCTGGGCCAGCAGCC
Seq2 Sscrofa8 chromosome3          .A.....................A.....................C.......T................
Seq3 Sscrofa8 chromosome3          ..........................A..................-...A....................
Seq4 Sscrofa8 chromosome8          .............................................-...A....................
Seq5 Sscrofa8 chromosome9          .............................................-...A....................
Seq6 Sscrofa8 chromosome17         .A.............................A.............-........................
Seq7 Sscrofa8 chromosome17         .A.............................A.............-........................
Seq8 Sscrofa8 chromosome16         .............................................-........................
Sus scrofa(AF435966)               ................C.........C..C........ACG....-........................
Sus scrofa(AJ293656)               .............................................-........................
Sus scrofa(AY099323)               ...............................A.............-........................
Sus scrofa(AJ133817)               ...............................A.............-........................
Sus scrofa(AF435967)               .T..........T.A.........T..C............C....-...C...........G..C..A..
Sus scrofa(AY099324)               ...............................A.............-........................
Sus scrofa(AJ293657)               ...............A.........A...................-........................
Sus scrofa(AJ133818)               .............................................-...A....................
Sus scrofa(AJ133816)               .............................................-...A....................
Sus scrofa(AM229312)               .............................................-........................
Sus scrofa(AM229311)               .............................................-........................
Sus scrofa(AM229313)               .............................................-........................
Sus scrofa(AY953542)               .............................................-........................
Sus scrofa(AY570980)               .............................................-........................
Sus scrofa(AJ279056)               .....A....................C..C...............-........................
Sus scrofa(AJ279057)               .............................................-...........A............
Sus scrofa(AY056035)               .............................................-........................
Sus scrofa(Y17013)                 .............................................-...A....................
```

```
Sus scrofa(EU523109)           .............................................-...A....................
Sus scrofa(EF133960)           .A.............................A...........A.-........................
Sus scrofa(DQ996273)           .............................................-........................
6 Sus scrofa                   .............................................-........................
9 Sus scrofa                   .............................................-........G...............
11 Sus scrofa                  ........................................T....-......T.................
14 Sus scrofa                  ....A....A....................T.........T....-......A.................
1 Sus barbatus barbatus        ......................C........A........T....-........................
2 Sus barbatus barbatus        .............................................-........................
3 Sus barbatus barbatus        .............................................-........................
5 Sus barbatus oi              .............................................-........................
6 Sus barbatus oi              .............................................-........................
8 Sus barbatus oi              .............................................-........................
9 Sus barbatus oi              .............................................-.......T................
3 Sus celebensis               .............................................-........................
6 Sus celebensis               .........................A...................-........................
8 Sus celebensis               .............................................-...A....................
2 Potamochoerus larvatus       .............................................-..........T.............
3 Potamochoerus larvatus       .A..AA........................T..........A...-..T.....................
M3 Potamochoerus larvatus      .............................................-..........T.............
4 Potamochoerus larvatus       .............................................-..........T.............
4 Potamochoerus porcus         .AA.AA...................................A...-..T.....................
5 Potamochoerus porcus         .AA.AA...................................A...-..T.....................
6 Potamochoerus porcus         .AA.AA.........A................A........A...-..T....................A
9 Potamochoerus porcus         ....A.................C.................T....-.............A..........
1 Phacochoerus africanus       ...............................A........T....-........................
5 Phacochoerus africanus       .............................................-........................
9 Phacochoerus africanus       .............................................-........................
11 Phacochoerus africanus      .............................................-........................
12 Phacochoerus africanus      ........................................T....-........................
14 Phacochoerus africanus      .............................................-........................
16 Phacochoerus africanus      ........................................T....-........................
2 Phacochoerus aethiopicus     .A...........A...............A.........G.....-.......T................
9 Phacochoerus aethiopicus     .A...........A...............A.........G.....-.......T................
16 Phacochoerus aethiopicus    ...........................T............T....-........................
17 Phacochoerus aethiopicus    ........................................T....-...........C............

                                        640       650       660       670       680       690       700
                               ....|....|....|....|....|....|....|....|....|....|....|....|....|....|
Seq1 Sscrofa8 chromosome1      TGCCGGAAGGAACTTCAGCACAAAAGGCTGAGCTCATGGCCCTCACGCAAGCTTTGCGGCTGGCCGAAGG
Seq2 Sscrofa8 chromosome3      ..T.......G....................A...G..........A....................G..
Seq3 Sscrofa8 chromosome3      ...................G..................................................
Seq4 Sscrofa8 chromosome8      ........................................................T.............
Seq5 Sscrofa8 chromosome9      ...................G..................................................
Seq6 Sscrofa8 chromosome17     ....A..............G..................................................
Seq7 Sscrofa8 chromosome17     ....A..............G..................................................
```

Seq8 Sscrofa8 chromosome16	...................**G**..
Sus scrofa(AF435966)	....
Sus scrofa(AJ293656)	....
Sus scrofa(AY099323)	...................**G**..
Sus scrofa(AJ133817)	...................**G**..
Sus scrofa(AF435967)	........**A**.**G**.....**CT**.........**G**.........**T**.**GG**....**T**.........**T**.......**G**.**C**....
Sus scrofa(AY099324)	...................**G**..
Sus scrofa(AJ293657)	...................**G**..
Sus scrofa(AJ133818)	...................**G**..
Sus scrofa(AJ133816)	...................**G**..
Sus scrofa(AM229312)	....
Sus scrofa(AM229311)	....
Sus scrofa(AM229313)	....
Sus scrofa(AY953542)	....
Sus scrofa(AY570980)	....
Sus scrofa(AJ279056)	....
Sus scrofa(AJ279057)	...................**G**..
Sus scrofa(AY056035)	...................**G**..
Sus scrofa(Y17013)	...................**G**..
Sus scrofa(EU523109)	...................**G**..
Sus scrofa(EF133960)	....**A**..............**G**...**C**..
Sus scrofa(DQ996273)	....
6 *Sus scrofa*	..................**TG**..
9 *Sus scrofa*	....
11 *Sus scrofa*	....
14 *Sus scrofa*	..........**G**..**A**....
1 *Sus barbatus barbatus*	..........**G**..**A**............
2 *Sus barbatus barbatus*	....
3 *Sus barbatus barbatus*	....
5 *Sus barbatus oi*	...................**G**..
6 *Sus barbatus oi*	...................**C**..
8 *Sus barbatus oi*	...................**G**..
9 *Sus barbatus oi*	...................**G**..
3 *Sus celebensis*	..........**G**........**G**..
6 *Sus celebensis*	....
8 *Sus celebensis*	..........**G**........**G**..
2 *Potamochoerus larvatus*	..........**G**...**A**.....
3 *Potamochoerus larvatus*	..........**G**...**A**....................**G**......................**A**.....**T**.....
M3 *Potamochoerus larvatus*	..........**G**...**A**.....
4 *Potamochoerus larvatus*	..........**G**...**A**.....
4 *Potamochoerus porcus*	....**A**.....**G**........................**G**..........**A**.................**T**.....
5 *Potamochoerus porcus*	....**A**.....**G**........................**G**..........**A**.................**T**.....
6 *Potamochoerus porcus*	..........**G**............................**T**......**A**.................**T**.....
9 *Potamochoerus porcus*	...................................**G**................**C**.................
1 *Phacochoerus africanus*	...................**G**...**G**...........**G**..................................
5 *Phacochoerus africanus*	..........**G**..**T**..................

```
9 Phacochoerus africanus       ....A.....G.....................................................T.....
11 Phacochoerus africanus      ..........G........................................T..................
12 Phacochoerus africanus      ...................................G..................................
14 Phacochoerus africanus      ..........G........................................T..................
16 Phacochoerus africanus      ...................................G.................................A
2 Phacochoerus aethiopicus     ..T.A.....G......A.................G..........A.......................
9 Phacochoerus aethiopicus     ..T.A.....G......A.................G..........A.......................
16 Phacochoerus aethiopicus    ...................G...............G..................................
17 Phacochoerus aethiopicus    ...................G...............G..................................

                                        710       720       730       740       750       760       770
                               ....|....|....|....|....|....|....|....|....|....|....|....|....|....|
Seq1 Sscrofa8 chromosome1      GAAATCCATAAACATTTATACAGACAGCAGGTATGCCTTTGCGACTGCACACGTACACGGGGCCATCTAT
Seq2 Sscrofa8 chromosome3      .....................G................................G..T............
Seq3 Sscrofa8 chromosome3      .....................G................................................
Seq4 Sscrofa8 chromosome8      ......................................................................
Seq5 Sscrofa8 chromosome9      .....................G................................................
Seq6 Sscrofa8 chromosome17     .....................G................................................
Seq7 Sscrofa8 chromosome17     ....................TG................................................
Seq8 Sscrofa8 chromosome16     .....................G................................................
Sus scrofa(AF435966)           ......................................................................
Sus scrofa(AJ293656)           ......................................................................
Sus scrofa(AY099323)           .....................G................................................
Sus scrofa(AJ133817)           .....................G................................................
Sus scrofa(AF435967)           ......................................................................
Sus scrofa(AY099324)           .....................G................................................
Sus scrofa(AJ293657)           .....................G................................................
Sus scrofa(AJ133818)           .....................G................................................
Sus scrofa(AJ133816)           .....................G................................................
Sus scrofa(AM229312)           .....................G...................................T............
Sus scrofa(AM229311)           .....................G...................................T............
Sus scrofa(AM229313)           .....................G...................................T............
Sus scrofa(AY953542)           ......................................................................
Sus scrofa(AY570980)           .....................G...................................T............
Sus scrofa(AJ279056)           ......................................................................
Sus scrofa(AJ279057)           .....................G................................................
Sus scrofa(AY056035)           .....................G................................................
Sus scrofa(Y17013)             .....................G................................................
Sus scrofa(EU523109)           .....................G................................................
Sus scrofa(EF133960)           .....................G................................................
Sus scrofa(DQ996273)           .....................G...................................T............
6 Sus scrofa                   .....................G................................................
9 Sus scrofa                   ......................................................................
11 Sus scrofa                  ......................................................................
14 Sus scrofa                  .....................G................................................
1 Sus barbatus barbatus        ..........G..........G................................................
```

```
2 Sus barbatus barbatus          ..................C...................................................
3 Sus barbatus barbatus          ..................C...................................................
5 Sus barbatus oi                .....................G................................................
6 Sus barbatus oi                .....................G................................................
8 Sus barbatus oi                .....................G................................................
9 Sus barbatus oi                .....................G....A......A....................................
3 Sus celebensis                 .....................G...........C....................................
6 Sus celebensis                 .....................G................................................
8 Sus celebensis                 ...............C.....G...................................T............
2 Potamochoerus larvatus         .....................G................................................
3 Potamochoerus larvatus         .....................T...........................T....GT..............
M3 Potamochoerus larvatus        .....................G................................................
4 Potamochoerus larvatus         .....................G................................................
4 Potamochoerus porcus           ...............C.....T...........................T....GT..............
5 Potamochoerus porcus           ...............C.....T...........................T....GT..............
6 Potamochoerus porcus           ...............C..A..T..............A...............A.G..T.....T......
9 Potamochoerus porcus           ....................TG..T...........A.................G........T......
1 Phacochoerus africanus         .....................G..T...........T.................G........T......
5 Phacochoerus africanus         ....................TG....................A...........................
9 Phacochoerus africanus         .....................G..............................A.......C.........
11 Phacochoerus africanus        ....................TG....................A...........................
12 Phacochoerus africanus        .....................G..T...........T.................G........T......
14 Phacochoerus africanus        ....................TG....................A...........................
16 Phacochoerus africanus        .....................G..T...........T.............C...G........T......
2 Phacochoerus aethiopicus       .....................G................................G...............
9 Phacochoerus aethiopicus       .....................G................................G...............
16 Phacochoerus aethiopicus      .....................G..T...........T.................G........T......
17 Phacochoerus aethiopicus      .....................G..T...........T.................G........T......

                                          780       790       800       810       820       830       840
                                 ....|....|....|....|....|....|....|....|....|....|....|....|....|....|
Seq1 Sscrofa8 chromosome1       AAGCAAAGGGGGTTGCTTACCTCAGCAGGGAGGGAAATAAAGAACAAAGAGGAAATTCTAAGCCTATTAG
Seq2 Sscrofa8 chromosome3       ..A.................................G.C...........AA...........T.G..G.
Seq3 Sscrofa8 chromosome3       ..A...................................................................
Seq4 Sscrofa8 chromosome8       ..........T...........................................................
Seq5 Sscrofa8 chromosome9       ..A...................................................................
Seq6 Sscrofa8 chromosome17      ......................................................................
Seq7 Sscrofa8 chromosome17      ......................................................................
Seq8 Sscrofa8 chromosome16      ..A...................................................................
Sus scrofa(AF435966)             ...........................................C..........................
Sus scrofa(AJ293656)             ......................................................................
Sus scrofa(AY099323)             ..A...................................................................
Sus scrofa(AJ133817)             ..A...................................................................
Sus scrofa(AF435967)             .C....................................................................
Sus scrofa(AY099324)             ..A...................................................................
Sus scrofa(AJ293657)             ..A...................................................................
```

Sus scrofa(AJ133818)	..A...
Sus scrofa(AJ133816)	..A...
Sus scrofa(AM229312)	..A...
Sus scrofa(AM229311)	..A...
Sus scrofa(AM229313)	..A..A..................
Sus scrofa(AY953542)	....
Sus scrofa(AY570980)	..A...
Sus scrofa(AJ279056)	....
Sus scrofa(AJ279057)	..A...T...................
Sus scrofa(AY056035)	..A...
Sus scrofa(Y17013)	..A...
Sus scrofa(EU523109)	..A...
Sus scrofa(EF133960)	....
Sus scrofa(DQ996273)	..A...
6 *Sus scrofa*	..A...
9 *Sus scrofa*	....A............................
11 *Sus scrofa*	....
14 *Sus scrofa*	............................A...
1 *Sus barbatus barbatus*	....
2 *Sus barbatus barbatus*	....
3 *Sus barbatus barbatus*	....
5 *Sus barbatus oi*	..A...
6 *Sus barbatus oi*	....
8 *Sus barbatus oi*	..A...
9 *Sus barbatus oi*	.................C..
3 *Sus celebensis*	....
6 *Sus celebensis*	....T..............................
8 *Sus celebensis*	..A.............C...
2 *Potamochoerus larvatus*	....
3 *Potamochoerus larvatus*	..A......A....A.....................G.C.....T...A..A...........T.G..G.
M3 *Potamochoerus larvatus*	....
4 *Potamochoerus larvatus*	....A.......
4 *Potamochoerus porcus*	..A...........A.....................G.C.....T..................T.G..G.
5 *Potamochoerus porcus*	..A...........A.....................G.C.....T..................T.G..G.
6 *Potamochoerus porcus*	.....G........A.....................G.C.....T................A.T.G..G.
9 *Potamochoerus porcus*	..A..G........A..C..................G.C.....T..............G...T......
1 *Phacochoerus africanus*	.....G........A.....................G.C.....T..............G...T......
5 *Phacochoerus africanus*	....
9 *Phacochoerus africanus*	....G..........
11 *Phacochoerus africanus*	....
12 *Phacochoerus africanus*	.....G........A.....................G.C.....T..............G...T......
14 *Phacochoerus africanus*	....
16 *Phacochoerus africanus*	.....G........A.....................G.C.....T..............G...T......
2 *Phacochoerus aethiopicus*	..A.................................G.C...........A............T.G..G.
9 *Phacochoerus aethiopicus*	..A.................................G.C...........A............T.G..G.
16 *Phacochoerus aethiopicus*	.....G........A.....................G.C.....T..............G...T......

```
17 Phacochoerus aethiopicus     .....G........A.....................G.C.....T..............G...T......

                                         850       860       870       880       890       900       910
                                ....|....|....|....|....|....|....|....|....|....|....|....|....|....|
Seq1 Sscrofa8 chromosome1       AAGCCTTACATTTGCCAAAAAGGCTAGCTATTATACACTGTCCTGGACATCAGAAAGCCAAAGATCCCAT
Seq2 Sscrofa8 chromosome3       .G..........................C........T..C.........................T...
Seq3 Sscrofa8 chromosome3       ..................................................................T...
Seq4 Sscrofa8 chromosome8       ......................................................................
Seq5 Sscrofa8 chromosome9       ..................................................................T...
Seq6 Sscrofa8 chromosome17      ..................................................................T...
Seq7 Sscrofa8 chromosome17      ..................................................................T...
Seq8 Sscrofa8 chromosome16      ..................................................................T...
Sus scrofa(AF435966)            ......................................................................
Sus scrofa(AJ293656)            ......................................................................
Sus scrofa(AY099323)            ..................................................................T...
Sus scrofa(AJ133817)            ..................................................................T...
Sus scrofa(AF435967)            ......................................................................
Sus scrofa(AY099324)            ..................................................................T...
Sus scrofa(AJ293657)            ..................................................................T...
Sus scrofa(AJ133818)            ..................................................................T...
Sus scrofa(AJ133816)            ..................................................................T...
Sus scrofa(AM229312)            .....G.......A............................................T.......T...
Sus scrofa(AM229311)            .....G.......A............................................T.......T...
Sus scrofa(AM229313)            .....G.......A............................................T.......T...
Sus scrofa(AY953542)            ......................................................................
Sus scrofa(AY570980)            .....G.......A..G.........................................T.......T...
Sus scrofa(AJ279056)            ......................................................................
Sus scrofa(AJ279057)            ..................................................................T...
Sus scrofa(AY056035)            ..................................................................T...
Sus scrofa(Y17013)              ..................................................................T...
Sus scrofa(EU523109)            ..................................................................T...
Sus scrofa(EF133960)            ......................................................................
Sus scrofa(DQ996273)            .....G.......A............................................T.......T...
6 Sus scrofa                    ...............................................T......................
9 Sus scrofa                    .............................................C........................
11 Sus scrofa                   ......................................................................
14 Sus scrofa                   .............................................C....................T...
1 Sus barbatus barbatus         ......................................A...........................T...
2 Sus barbatus barbatus         ......................................................................
3 Sus barbatus barbatus         ......................................................................
5 Sus barbatus oi               ..................................................................T...
6 Sus barbatus oi               ......................................................................
8 Sus barbatus oi               ..................................................................T...
9 Sus barbatus oi               .............A........................................................
3 Sus celebensis                ..................................................................T...
6 Sus celebensis                ..............................................G.......................
```

```
8 Sus celebensis              ..................................................................T...
2 Potamochoerus larvatus      ..................................................................T...
3 Potamochoerus larvatus      .G.............T............C........T..C.T...GT..................T...
M3 Potamochoerus larvatus     ..................................................................T...
4 Potamochoerus larvatus      ....T.............................................................T...
4 Potamochoerus porcus        .G.............T............C........T..C.T...GT..................T...
5 Potamochoerus porcus        .G.............T............C........T..C.T...GT..................T...
6 Potamochoerus porcus        .G.............T............C........T.AC.T......................GT...
9 Potamochoerus porcus        .G...C......................C..C.....T..C.G...G...........T.G.....T...
1 Phacochoerus africanus      .G...C...........G..........C..C.....T..C.G...G.............G.....T...
5 Phacochoerus africanus      ..........A.............................................A.........T...
9 Phacochoerus africanus      ..........A.............................................A.........T...
11 Phacochoerus africanus     ..........A.........................G...................A.........T...
12 Phacochoerus africanus     .G...C......................C..C.....T..C.G...G.............G.....T...
14 Phacochoerus africanus     ..........A..................................C..........A.........T...
16 Phacochoerus africanus     .G...C........T.............C..C.....T..C.G...G.............G.....T...
2 Phacochoerus aethiopicus    .G..........................CG.......T..C....C....................T...
9 Phacochoerus aethiopicus    .G..........................CG.......T..C....C....................T...
16 Phacochoerus aethiopicus   .G...C......................C..C.....T..C.G...G.............G.....T...
17 Phacochoerus aethiopicus   .G...C......................C..C.....T..C.G...G.............G.....T...

                                      920       930       940       950       960       970       980
                              ....|....|....|....|....|....|....|....|....|....|....|....|....|....|
Seq1 Sscrofa8 chromosome1     ATCCAGAGGGAACCAGATGGCTGACCGGGTTGCCAAGCAGGCAGCCCAGGGTGTTAACCTTCTGCCTATG
Seq2 Sscrofa8 chromosome3     C....A...A................A...C........A..T..........................A
Seq3 Sscrofa8 chromosome3     ...T..............................................C..................A
Seq4 Sscrofa8 chromosome8     .....................................................................A
Seq5 Sscrofa8 chromosome9     ...T..............................................C..................A
Seq6 Sscrofa8 chromosome17    .....................................................................A
Seq7 Sscrofa8 chromosome17    .....................................................................A
Seq8 Sscrofa8 chromosome16    ...T..............................................C..................A
Sus scrofa(AF435966)          ......................................................................
Sus scrofa(AJ293656)          ......................................................................
Sus scrofa(AY099323)          ...T..............................................C..................A
Sus scrofa(AJ133817)          ...T..............................................C..................A
Sus scrofa(AF435967)          ......................................................................
Sus scrofa(AY099324)          ...T..............................................C..................A
Sus scrofa(AJ293657)          ...T..............................................C..................A
Sus scrofa(AJ133818)          ...T..............................................C..................A
Sus scrofa(AJ133816)          ...T..............................................C..................A
Sus scrofa(AM229312)          .........A...........................................................A
Sus scrofa(AM229311)          .........A...........................................................A
Sus scrofa(AM229313)          .........A...........................................................A
Sus scrofa(AY953542)          ......................................................................
Sus scrofa(AY570980)          .........A...........................................................A
Sus scrofa(AJ279056)          ......................................................................
```

```
Sus scrofa(AJ279057)          ...T........................A.....................C..................A
Sus scrofa(AY056035)          ...T..............................................C..................A
Sus scrofa(Y17013)            ...T..............................................C..................A
Sus scrofa(EU523109)          ...T..............................................C..................A
Sus scrofa(EF133960)          .....................................................................A
Sus scrofa(DQ996273)          .........A...........................................................A
6 Sus scrofa                  ...T..............................................C..................A
9 Sus scrofa                  ......................................................................
11 Sus scrofa                 ......................................................................
14 Sus scrofa                 ..........................A.............................C............A
1 Sus barbatus barbatus       .....................................................................A
2 Sus barbatus barbatus       ........................T.........................C..................A
3 Sus barbatus barbatus       ........................T.........................C..................A
5 Sus barbatus oi             ..AT.................................................................A
6 Sus barbatus oi             ..........................A..........................................A
8 Sus barbatus oi             ..AT.................................................................A
9 Sus barbatus oi             ............T........................................................A
3 Sus celebensis              .....................................................................A
6 Sus celebensis              .....................................................................A
8 Sus celebensis              ...T.................................................................A
2 Potamochoerus larvatus      ..............................C......................................A
3 Potamochoerus larvatus      C..A.A..AA..........----..A...CA..............T...........TC.........A
M3 Potamochoerus larvatus     ..............................C......................................A
4 Potamochoerus larvatus      ..............................C......................................A
4 Potamochoerus porcus        C..A.A...A...........................................................A
5 Potamochoerus porcus        C..A.A...A...........................................................A
6 Potamochoerus porcus        C..A.A.AAA...............T-....A.....................................A
9 Potamochoerus porcus        C...GA...A...........C....A...C..................A..........AT.A.....A
1 Phacochoerus africanus      C..TGA...A...........C....A...C..................A...C......AT.A.....A
5 Phacochoerus africanus      .....................................................................A
9 Phacochoerus africanus      .....................................................................A
11 Phacochoerus africanus     ............T........................................................A
12 Phacochoerus africanus     C..TGA...A...........C....A...C..................A..........AT.A.....A
14 Phacochoerus africanus     .....................................................................A
16 Phacochoerus africanus     C..TGA...A...........C....A...C....G.............A..........AT.A.....A
2 Phacochoerus aethiopicus    C....A...A................A...C.......GA..T..........................A
9 Phacochoerus aethiopicus    C....A...A................A...C.......GA..T..........................A
16 Phacochoerus aethiopicus   C..TGA...A...........C....A...C..................A..........AT.A.....A
17 Phacochoerus aethiopicus   C..TGA..............TC....A...C..................A..........AT.A.....A

                                     990       1000      1010      1020      1030      1040      1050
                              ....|....|....|....|....|....|....|....|....|....|....|....|....|....|
Seq1 Sscrofa8 chromosome1     ATAGAAACACCCAAAGCCCCAGAACCCGGACGACAGTACACCCTAGAAGACTGGCAAGAAATAAAAAAGA
Seq2 Sscrofa8 chromosome3     .......TG..........TG........G.............................G..........
Seq3 Sscrofa8 chromosome3     ........G..................A...............................G..........
Seq4 Sscrofa8 chromosome8     ..................................C........................G..........
```

Seq5 Sscrofa8 chromosome9	........G..................A...............................G..........
Seq6 Sscrofa8 chromosome17	........G..................A...............................G..........
Seq7 Sscrofa8 chromosome17	........G..................A...............................G..........
Seq8 Sscrofa8 chromosome16	........G..................A...............................G..........
Sus scrofa(AF435966)	....
Sus scrofa(AJ293656)	....
Sus scrofa(AY099323)	........G..................A...............................G..........
Sus scrofa(AJ133817)	........G..................A...............................G..........
Sus scrofa(AF435967)	....
Sus scrofa(AY099324)	........G..................A...............................G..........
Sus scrofa(AJ293657)	........G..................A...............................G..........
Sus scrofa(AJ133818)	........G..................A...............................G..........
Sus scrofa(AJ133816)	........G..................A...............................G..........
Sus scrofa(AM229312)	......TG..................A...............................G..........
Sus scrofa(AM229311)	......TG..................A...............................G..........
Sus scrofa(AM229313)	......TG..................A...............................G..........
Sus scrofa(AY953542)	....G..........
Sus scrofa(AY570980)	......TG..................A...............................G..........
Sus scrofa(AJ279056)	....
Sus scrofa(AJ279057)	........G..................A...............................G..........
Sus scrofa(AY056035)	........G..................A...............................G..........
Sus scrofa(Y17013)	........G....G.............A...............................G..........
Sus scrofa(EU523109)	........G..................A...............................G..........
Sus scrofa(EF133960)	....G..........
Sus scrofa(DQ996273)	......TG..................A...............................G..........
6 *Sus scrofa*	........G..................A...............................G..........
9 *Sus scrofa*	....
11 *Sus scrofa*	....
14 *Sus scrofa*	...A....G..G..........
1 *Sus barbatus barbatus*	....G..........
2 *Sus barbatus barbatus*	....C..............G..........
3 *Sus barbatus barbatus*	....C..............G..........
5 *Sus barbatus oi*	........G..................A.......C.......................G..........
6 *Sus barbatus oi*	....G..........
8 *Sus barbatus oi*	........G..................A.......C.......................G..........
9 *Sus barbatus oi*	....G..........
3 *Sus celebensis*	........G..................A...............................G..........
6 *Sus celebensis*	....G..........
8 *Sus celebensis*	........G..................A...............................G..........
2 *Potamochoerus larvatus*	......TG..G..........
3 *Potamochoerus larvatus*	...A.....T....................T.G.......................G..G.....G..A.
M3 *Potamochoerus larvatus*	......TG..G..........
4 *Potamochoerus larvatus*	........G..G..........
4 *Potamochoerus porcus*	..............................T.G.................T.....G..G........A.
5 *Potamochoerus porcus*	..............................T.G.................T.....G..G........A.
6 *Potamochoerus porcus*	........G.....................T..............A..........G..G........A.

```
9 Potamochoerus porcus          .....G..GT....................T.G......G..............................
1 Phacochoerus africanus        .....G..GT................A.....G.....................................
5 Phacochoerus africanus        ........G.....................T............................G..........
9 Phacochoerus africanus        ........G.......T.........T................................G..........
11 Phacochoerus africanus       ........G.....................T............................G..........
12 Phacochoerus africanus       .....G..GT................T.....G.....................................
14 Phacochoerus africanus       ........G.....................T............................G..........
16 Phacochoerus africanus       .....G..GT................T.....G.....................................
2 Phacochoerus aethiopicus      ........G..........................A.......................G........A.
9 Phacochoerus aethiopicus      ........G..........................A.......................G........A.
16 Phacochoerus aethiopicus     .....G..GT................T.....G.....................................
17 Phacochoerus aethiopicus     .....G..GT................T.....G.....................................

                                         1060      1070      1080      1090      1100      1110      1120
                                ....|....|....|....|....|....|....|....|....|....|....|....|....|....|
Seq1 Sscrofa8 chromosome1       TAGACCAGTTCTCTGAAACTCCGGAGGGGACCTGCTATACCTCAGATGGGAAGGAAATCCTGCCCCACAA
Seq2 Sscrofa8 chromosome3       .....TGT........G..C..A................................G..............
Seq3 Sscrofa8 chromosome3       ................G...........................T...A.....................
Seq4 Sscrofa8 chromosome8       ......................................................................
Seq5 Sscrofa8 chromosome9       ................G...........................T.........................
Seq6 Sscrofa8 chromosome17      ................G..........................G..........................
Seq7 Sscrofa8 chromosome17      ................G..........................G..........................
Seq8 Sscrofa8 chromosome16      ................G...........................T.........................
Sus scrofa(AF435966)            ......................................................................
Sus scrofa(AJ293656)            ......................................................................
Sus scrofa(AY099323)            ................G...........................T.........................
Sus scrofa(AJ133817)            ................G...........................T.........................
Sus scrofa(AF435967)            ......................................................................
Sus scrofa(AY099324)            ................G...........................T.........................
Sus scrofa(AJ293657)            ................G...........................T.........................
Sus scrofa(AJ133818)            ................G...........................T.........................
Sus scrofa(AJ133816)            ................G...........................T.........................
Sus scrofa(AM229312)            ................G........A............................................
Sus scrofa(AM229311)            ................G........A............................................
Sus scrofa(AM229313)            ................G........A............................................
Sus scrofa(AY953542)            ................G........A............................................
Sus scrofa(AY570980)            ................G........A............................................
Sus scrofa(AJ279056)            ......................................................................
Sus scrofa(AJ279057)            ................G...........................T.........................
Sus scrofa(AY056035)            ................G...........................T.........................
Sus scrofa(Y17013)              ................G...........................T.........................
Sus scrofa(EU523109)            ................G...........................T.........................
Sus scrofa(EF133960)            ..........................A...........................................
Sus scrofa(DQ996273)            ................G........A............................................
6 Sus scrofa                    ................G...........................T....................A....
9 Sus scrofa                    ......................................................................
```

```
11 Sus scrofa                    ......................................................................
14 Sus scrofa                    ................G....T.........................A.A....................
1 Sus barbatus barbatus          ................G.....A...............................................
2 Sus barbatus barbatus          ......................................................................
3 Sus barbatus barbatus          ......................................................................
5 Sus barbatus oi                ................G...........................T.........................
6 Sus barbatus oi                ......................................................................
8 Sus barbatus oi                ................G...........................T.........................
9 Sus barbatus oi                ......................................................................
3 Sus celebensis                 ................G.....................................................
6 Sus celebensis                 ......................................................................
8 Sus celebensis                 ................G.....................................................
2 Potamochoerus larvatus         ................G.....A...............................................
3 Potamochoerus larvatus         ......G.......AGG.TG..A...A.....................AA.....G.....A........
M3 Potamochoerus larvatus        ................G.....A...............................................
4 Potamochoerus larvatus         ................G.....A..........................A....................
4 Potamochoerus porcus           ......G.......AGG.TG..AA..A.....................AA....................
5 Potamochoerus porcus           ......G.......AGG.TG..AA..A.....................AA....................
6 Potamochoerus porcus           ......G.......AGG.TG..A...A.....................AA...A................
9 Potamochoerus porcus           ....A...........G..C..A.......TT....GC...C....A...................G...
1 Phacochoerus africanus         ....A.G.........G..C..A.......TT....GC...C....A.......................
5 Phacochoerus africanus         ................G.....................................................
9 Phacochoerus africanus         ................G.....................................................
11 Phacochoerus africanus        ................G.....................................................
12 Phacochoerus africanus        ....A.G.........G..C..A.......TT....GC...C....A.......................
14 Phacochoerus africanus        ................G.....................................................
16 Phacochoerus africanus        ....A.G.........G..C..A.......TT....GC...C....A.......................
2 Phacochoerus aethiopicus       ......G.........G..CT..................................G..............
9 Phacochoerus aethiopicus       ......G.........G..CT..................................G..............
16 Phacochoerus aethiopicus      ....A.G.........G..C..A.......TT....GC...C....A.......................
17 Phacochoerus aethiopicus      ....A.G.........G..C..A.......TT....GC...C....A.......................

                                         1130      1140      1150      1160      1170      1180      1190
                                 ....|....|....|....|....|....|....|....|....|....|....|....|....|....|
Seq1 Sscrofa8 chromosome1        AGAAGGGTTAGAATATGTCCAACAGATACATCGTCTAACCCACCTAGGAACTAAACACCTGCAGCAGTTG
Seq2 Sscrofa8 chromosome3        ............G..............G.....................G.......T............
Seq3 Sscrofa8 chromosome3        ....................................C.................................
Seq4 Sscrofa8 chromosome8        ..................................................................C...
Seq5 Sscrofa8 chromosome9        ....................................C.................................
Seq6 Sscrofa8 chromosome17       ......................................................................
Seq7 Sscrofa8 chromosome17       ......................................................................
Seq8 Sscrofa8 chromosome16       ..............................C.....C.................................
Sus scrofa(AF435966)             ..........A...........................................................
Sus scrofa(AJ293656)             ......................................................................
Sus scrofa(AY099323)             ......................................................................
Sus scrofa(AJ133817)             ....................................C.................................
```

Sus scrofa(AF435967)	....
Sus scrofa(AY099324)	....................................C.................................
Sus scrofa(AJ293657)	....................................C.................................
Sus scrofa(AJ133818)	....................................C.................................
Sus scrofa(AJ133816)	....................................C.................................
Sus scrofa(AM229312)	....
Sus scrofa(AM229311)	....
Sus scrofa(AM229313)	....
Sus scrofa(AY953542)	....
Sus scrofa(AY570980)	....
Sus scrofa(AJ279056)	....
Sus scrofa(AJ279057)	....................................C........................T........
Sus scrofa(AY056035)	..............................C.....C.................................
Sus scrofa(Y17013)	....................................C.................................
Sus scrofa(EU523109)	....................................C.................................
Sus scrofa(EF133960)	....
Sus scrofa(DQ996273)	....
6 *Sus scrofa*	..................................T.C.................................
9 *Sus scrofa*	....
11 *Sus scrofa*	....T......................
14 *Sus scrofa*	..........A....................A............................A.........
1 *Sus barbatus barbatus*	....
2 *Sus barbatus barbatus*	....
3 *Sus barbatus barbatus*	....
5 *Sus barbatus oi*	....G..............
6 *Sus barbatus oi*	....
8 *Sus barbatus oi*	....G..............
9 *Sus barbatus oi*	....................G...
3 *Sus celebensis*	....
6 *Sus celebensis*	....
8 *Sus celebensis*	....
2 *Potamochoerus larvatus*	....
3 *Potamochoerus larvatus*	.........G.....C...........G...T.................G....G..T...........A
M3 *Potamochoerus larvatus*	....
4 *Potamochoerus larvatus*	....
4 *Potamochoerus porcus*	.......C................A..G.....................G....G..T...........A
5 *Potamochoerus porcus*	.......C................A..G.....................G....G..T...........A
6 *Potamochoerus porcus*	.......C..A.............A..G..................A..G.......T...........-
9 *Potamochoerus porcus*	.........G.....C...........G.....................G....G..T...........A
1 *Phacochoerus africanus*	.........G.....C...G.......G.....................G....G..T...........A
5 *Phacochoerus africanus*	....
9 *Phacochoerus africanus*	....
11 *Phacochoerus africanus*	....
12 *Phacochoerus africanus*	.........G.....C...G.......G.....................G....G..T...........A
14 *Phacochoerus africanus*	....
16 *Phacochoerus africanus*	.........G.....C...G.......G.....................G....G..T...........A

```
2 Phacochoerus aethiopicus      .A..........G....................................G.......T............
9 Phacochoerus aethiopicus      .A..........G....................................G.......T............
16 Phacochoerus aethiopicus     .........G.....C...G.......G.....................G....G..T...........A
17 Phacochoerus aethiopicus     .........G.....C...G.......G.....................G....G..T...........A

                                         1200      1210      1220      1230      1240      1250      1260
                                ....|....|....|....|....|....|....|....|....|....|....|....|....|....|
Seq1 Sscrofa8 chromosome1       GTCAGAACATCCCCTTATCATGTTCTGAGGCTACCAGGAGTGGCTGACTCGGTGGTCAAACATTGTGTGC
Seq2 Sscrofa8 chromosome3       ................---..........A........................................
Seq3 Sscrofa8 chromosome3       ......................................................................
Seq4 Sscrofa8 chromosome8       ..................................................A...................
Seq5 Sscrofa8 chromosome9       ......................................................................
Seq6 Sscrofa8 chromosome17      .....................................................C................
Seq7 Sscrofa8 chromosome17      ......................................................................
Seq8 Sscrofa8 chromosome16      ..................................................A...................
Sus scrofa(AF435966)            ......................................................................
Sus scrofa(AJ293656)            ......................................................................
Sus scrofa(AY099323)            ......................................................................
Sus scrofa(AJ133817)            ......................................................................
Sus scrofa(AF435967)            ...............................................T......................
Sus scrofa(AY099324)            ......................................................................
Sus scrofa(AJ293657)            ......................................................................
Sus scrofa(AJ133818)            ......................................................................
Sus scrofa(AJ133816)            ......................................................................
Sus scrofa(AM229312)            ......................................................................
Sus scrofa(AM229311)            ......................................................................
Sus scrofa(AM229313)            ......................................................................
Sus scrofa(AY953542)            ...........T...................................T..............C.......
Sus scrofa(AY570980)            ......................................................................
Sus scrofa(AJ279056)            ...............................................T......................
Sus scrofa(AJ279057)            ......................................................................
Sus scrofa(AY056035)            ..................................................A...................
Sus scrofa(Y17013)              ......................................................................
Sus scrofa(EU523109)            ......................................................................
Sus scrofa(EF133960)            ......................................................................
Sus scrofa(DQ996273)            ......................................................................
6 Sus scrofa                    ......................................................................
9 Sus scrofa                    ......................................................................
11 Sus scrofa                   ......................................................................
14 Sus scrofa                   .........................................................T............
1 Sus barbatus barbatus         A.....................................................................
2 Sus barbatus barbatus         ......G...................................T...........................
3 Sus barbatus barbatus         ......G...................................T...........................
5 Sus barbatus oi               .............T........................................................
6 Sus barbatus oi               ....C.................................................................
8 Sus barbatus oi               .............T........................................................
```

```
9 Sus barbatus oi            ......................................................................
3 Sus celebensis             ......................................................................
6 Sus celebensis             ......................................................................
8 Sus celebensis             ......................................................................
2 Potamochoerus larvatus     ......................................................................
3 Potamochoerus larvatus     ..............C.............AAT.......................................
M3 Potamochoerus larvatus    ......................................................................
4 Potamochoerus larvatus     ...........A..........................................................
4 Potamochoerus porcus       ............................AAT...............................–.......
5 Potamochoerus porcus       ............................AAT...............................–.......
6 Potamochoerus porcus       ---.........................AAT...........A...........................
9 Potamochoerus porcus       ............................AAT.................................A.....
1 Phacochoerus africanus     ..............C.............AAT...................A...................
5 Phacochoerus africanus     ................................................G.....................
9 Phacochoerus africanus     ..............................T.................G.....................
11 Phacochoerus africanus    ................................................G.....................
12 Phacochoerus africanus    ..............C.............AAT.......................................
14 Phacochoerus africanus    ................................................G.....................
16 Phacochoerus africanus    ..............C.............AAT.......................................
2 Phacochoerus aethiopicus   .............................A....................A...................
9 Phacochoerus aethiopicus   .............................A....................A...................
16 Phacochoerus aethiopicus  ..............C.............AAT.......................................
17 Phacochoerus aethiopicus  ....A.........C.............AAT.......................................

                                     1270      1280      1290      1300      1310      1320      1330
                             ....|....|....|....|....|....|....|....|....|....|....|....|....|....|
Seq1 Sscrofa8 chromosome1     CCTGCCAGCTGGTTAATGCTAATCCTTCCAGAATACCTCCAGGAAAGAGACTAAGGGGAAGCCACCCAGG
Seq2 Sscrofa8 chromosome3     ..................................G....TG.............................
Seq3 Sscrofa8 chromosome3     ......................................................................
Seq4 Sscrofa8 chromosome8     ..........................................................G...........
Seq5 Sscrofa8 chromosome9     ......................................................................
Seq6 Sscrofa8 chromosome17    ......................................................................
Seq7 Sscrofa8 chromosome17    ......................................................................
Seq8 Sscrofa8 chromosome16    ......................................................................
Sus scrofa(AF435966)         .....................................C................................
Sus scrofa(AJ293656)         ......................................................................
Sus scrofa(AY099323)         ......................................................................
Sus scrofa(AJ133817)         ......................................................................
Sus scrofa(AF435967)         ......................................................................
Sus scrofa(AY099324)         ......................................................................
Sus scrofa(AJ293657)         ......................................................................
Sus scrofa(AJ133818)         ................................G.....................................
Sus scrofa(AJ133816)         ......................................................................
Sus scrofa(AM229312)         ..................................G........G..........................
Sus scrofa(AM229311)         ..................................G........G..........................
Sus scrofa(AM229313)         ..................................G........G..........................
```

```
Sus scrofa(AY953542)              ......................................................................
Sus scrofa(AY570980)              ..................................G........G..........................
Sus scrofa(AJ279056)              ......................................................................
Sus scrofa(AJ279057)              ..............................A.G..........G..............G...........
Sus scrofa(AY056035)              ......................................................................
Sus scrofa(Y17013)                ......................................................................
Sus scrofa(EU523109)              ......................................................................
Sus scrofa(EF133960)              ......................................................................
Sus scrofa(DQ996273)              ..................................G........G..........................
6 Sus scrofa                      ......................................................................
9 Sus scrofa                      ......................................................................
11 Sus scrofa                     ..........................................A...........................
14 Sus scrofa                     ..........................................A...........................
1 Sus barbatus barbatus           ...........A..........................................................
2 Sus barbatus barbatus           ......................................................................
3 Sus barbatus barbatus           ......................................................................
5 Sus barbatus oi                 ......................................................................
6 Sus barbatus oi                 ..............................................A...........G...........
8 Sus barbatus oi                 ......................................................................
9 Sus barbatus oi                 ......................................................................
3 Sus celebensis                  ......................................................................
6 Sus celebensis                  ......................................................................
8 Sus celebensis                  .....................................A................................
2 Potamochoerus larvatus          ......................................................................
3 Potamochoerus larvatus          .........................................................A............
M3 Potamochoerus larvatus         ......................................................................
4 Potamochoerus larvatus          ..............................AG........G.............................
4 Potamochoerus porcus            ........................................G................A............
5 Potamochoerus porcus            ........................................G................A............
6 Potamochoerus porcus            ................G..........A.............A............................
9 Potamochoerus porcus            ..C.....................................G................A............
1 Phacochoerus africanus          ..........C...................AG........G.............................
5 Phacochoerus africanus          ......................................................................
9 Phacochoerus africanus          ......................................................................
11 Phacochoerus africanus         ..........A...........................................................
12 Phacochoerus africanus         ..........C...................AG........G.............................
14 Phacochoerus africanus         ......................................................................
16 Phacochoerus africanus         ..........C...................AG........G.............................
2 Phacochoerus aethiopicus        .................A............C.........G........G............T.......
9 Phacochoerus aethiopicus        .................A............C.........G........G............T.......
16 Phacochoerus aethiopicus       ..........C...................AG........G.............................
17 Phacochoerus aethiopicus       ..........C...................AG........G.............................

                                           1340      1350      1360      1370      1380      1390      1400
                                  ....|....|....|....|....|....|....|....|....|....|....|....|....|....|
Seq1 Sscrofa8 chromosome1         CGCTCACTGGGAAGTGGACTTCACTGAGGTAAAGCCGGCTAAATACGGAAACAAATATCTATTGGTTTTT
```

Seq2 Sscrofa8 chromosome3	**T**..........................**A**..
Seq3 Sscrofa8 chromosome3	....
Seq4 Sscrofa8 chromosome8	....**T**...............................**A**....................**C**............
Seq5 Sscrofa8 chromosome9	....
Seq6 Sscrofa8 chromosome17	....................................**A**.................................
Seq7 Sscrofa8 chromosome17	....................................**A**.................................
Seq8 Sscrofa8 chromosome16	....
Sus scrofa(AF435966)	....
Sus scrofa(AJ293656)	....
Sus scrofa(AY099323)	....
Sus scrofa(AJ133817)	....
Sus scrofa(AF435967)	....
Sus scrofa(AY099324)	....
Sus scrofa(AJ293657)	....
Sus scrofa(AJ133818)	....
Sus scrofa(AJ133816)	....
Sus scrofa(AM229312)	....**C**............
Sus scrofa(AM229311)	....**C**............
Sus scrofa(AM229313)	....**C**............
Sus scrofa(AY953542)	....
Sus scrofa(AY570980)	....
Sus scrofa(AJ279056)	....
Sus scrofa(AJ279057)	.........................**A**.**A**.............................**C**............
Sus scrofa(AY056035)	....
Sus scrofa(Y17013)	....
Sus scrofa(EU523109)	....
Sus scrofa(EF133960)	....
Sus scrofa(DQ996273)	....**C**............
6 *Sus scrofa*	....
9 *Sus scrofa*	....
11 *Sus scrofa*	............................**A**..................................**A**......
14 *Sus scrofa*	............................**A**..................................**A**......
1 *Sus barbatus barbatus*	....................................**A**.................................
2 *Sus barbatus barbatus*	................................**G**.....................................
3 *Sus barbatus barbatus*	................................**G**.....................................
5 *Sus barbatus oi*	....
6 *Sus barbatus oi*	.............**A**.................................**A**.........**C**............
8 *Sus barbatus oi*	....
9 *Sus barbatus oi*	....
3 *Sus celebensis*	........................**C**...
6 *Sus celebensis*	....
8 *Sus celebensis*	....
2 *Potamochoerus larvatus*	....**C**...
3 *Potamochoerus larvatus*	.**A**..............................**G**.....................................
M3 *Potamochoerus larvatus*	....**C**...
4 *Potamochoerus larvatus*	**T**...**A**.......................

4 *Potamochoerus porcus*	...................................T..........A..................G....
5 *Potamochoerus porcus*	...................................T..........A..................G....
6 *Potamochoerus porcus*	.A..
9 *Potamochoerus porcus*	....
1 *Phacochoerus africanus*	....
5 *Phacochoerus africanus*	....
9 *Phacochoerus africanus*	...C..
11 *Phacochoerus africanus*	....A.......................
12 *Phacochoerus africanus*	....
14 *Phacochoerus africanus*	....T...
16 *Phacochoerus africanus*	....
2 *Phacochoerus aethiopicus*	....
9 *Phacochoerus aethiopicus*	....
16 *Phacochoerus aethiopicus*	....
17 *Phacochoerus aethiopicus*	....

	1410 1420 1430 1440 1450 1460
	....|....|....|....|....|....|....|....|....|....|....|....|....|.
Seq1 Sscrofa8 chromosome1	**GTAGACACCTTTTCAGGATGGGTAGAGGCTTATCCTACTAAGAAAGAGACTTCAACCGTGGTGGCT**
Seq2 Sscrofa8 chromosome3	.......................C..
Seq3 Sscrofa8 chromosome3	....
Seq4 Sscrofa8 chromosome8	....
Seq5 Sscrofa8 chromosome9	....
Seq6 Sscrofa8 chromosome17	....
Seq7 Sscrofa8 chromosome17	....A........
Seq8 Sscrofa8 chromosome16	....
Sus scrofa(AF435966)	....
Sus scrofa(AJ293656)	....
Sus scrofa(AY099323)	....
Sus scrofa(AJ133817)	....
Sus scrofa(AF435967)	....
Sus scrofa(AY099324)	....
Sus scrofa(AJ293657)	....
Sus scrofa(AJ133818)	....
Sus scrofa(AJ133816)	....
Sus scrofa(AM229312)	....
Sus scrofa(AM229311)	....
Sus scrofa(AM229313)	....
Sus scrofa(AY953542)	....
Sus scrofa(AY570980)	....
Sus scrofa(AJ279056)	....
Sus scrofa(AJ279057)	....
Sus scrofa(AY056035)	....
Sus scrofa(Y17013)	....
Sus scrofa(EU523109)	....
Sus scrofa(EF133960)	....

Sus scrofa(DQ996273)	....
6 *Sus scrofa*	....
9 *Sus scrofa*	....
11 *Sus scrofa*	....T.........
14 *Sus scrofa*	....T.........
1 *Sus barbatus barbatus*	....
2 *Sus barbatus barbatus*	....
3 *Sus barbatus barbatus*	....
5 *Sus barbatus oi*	....A........
6 *Sus barbatus oi*	....
8 *Sus barbatus oi*	....A........
9 *Sus barbatus oi*	....
3 *Sus celebensis*	....
6 *Sus celebensis*	....
8 *Sus celebensis*	....
2 *Potamochoerus larvatus*	....
3 *Potamochoerus larvatus*	........T....................................A....................
M3 *Potamochoerus larvatus*	....A....................
4 *Potamochoerus larvatus*	........T...
4 *Potamochoerus porcus*	........T.....................................G...................
5 *Potamochoerus porcus*	........T.....................................G...................
6 *Potamochoerus porcus*	A..............A..................................C...............
9 *Potamochoerus porcus*	........T................................A....-...................
1 *Phacochoerus africanus*	........T..............C..
5 *Phacochoerus africanus*	....
9 *Phacochoerus africanus*	....
11 *Phacochoerus africanus*	........T..............C..
12 *Phacochoerus africanus*	........-...
14 *Phacochoerus africanus*	....
16 *Phacochoerus africanus*	........T..............C..
2 *Phacochoerus aethiopicus*	.......................C.................A........................
9 *Phacochoerus aethiopicus*	.......................C.................A........................
16 *Phacochoerus aethiopicus*	........T..............C..
17 *Phacochoerus aethiopicus*	........T..............C..
