# Supplementary material for: Evolution of endogenous retroviruses in the Suidae: evidence for different viral subpopulations in African and Eurasian host species
Source: BMC Evol Biol. 2011 May 24;11:139. doi: 10.1186/1471-2148-11-139 (PMC3128044; doi:10.1186/1471-2148-11-139)
Supplement: Additional file 4 — env A alignment. env A alignment of sequences generated in this study, sequences from GenBank and the draft pig genome [file 1471-2148-11-139-S4.PDF]

env A alignment (including all sequences)

The first number next to some sequence names represents the clone number. A = fragment amplified using primers for *env* A; C = fragment amplified using primers for *env* C.

```
                                     10        20        30        40        50        60        70
                                 ....|....|....|....|....|....|....|....|....|....|....|....|....|....|
Seq1 Sscrofa8 chromosome8        ACCTGGTGGCATGAGTTATATGTCTGCCTTCGATCAGTAATCCCTGGCCTCAATGACCAGGCCACA-CCC
Seq2 Sscrofa8 chromosome10       ..........C........................G...........T.....C............-...
Seq3 Sscrofa8 chromosome13       ..........C....................................T..................-...
Sus scrofa(EU086224)             ..........C................T.......G...........T.....C............-...
Sus scrofa(EU086222)             ..........C................T.......G...........T.....C............-...
Sus scrofa(EU086220)             ..........C....................................T..................-...
Sus scrofa(EU086219)             ..........C....................................T..................-...
Sus scrofa(AJ288585)             ..........C...A................................T..................-...
Sus scrofa(AJ293656)             ..........C...A................................T..................-...
Sus scrofa(EU789636)             ..........C...A................................T..................-...
Sus scrofa(AF435967)             ..........CC..A................................T..................-...
Sus scrofa(AY288779)             ..........C...A................................T..................-...
Sus scrofa(EU086221)             ..........C...A...................................................-...
Sus scrofa(EF133960)             ..........C....................................T..................-...
Sus scrofa(AY368582)             ..........C....................................T..................-...
Sus scrofa(AY368580)             ..........C....................................T..................-...
Sus scrofa(AF417222)             ..........C...A................................T..................-...
Sus scrofa(AF417225)             ..........C...A................................T..................-...
Sus scrofa(AY368587)             .....A....C...A................................T..................-...
Sus scrofa(AY368581)             ..........C...A................................T..................-...
Sus scrofa(AY312524)             ..........C...A.............C..................T..................-...
Sus scrofa(AY371067)             ..........C...A................................T..................-...
Sus scrofa(AY312526)             ..........C...A................................T..................-...
Sus scrofa(AY368588)             ..........C...A................................T..................-...
Sus scrofa(AF417223)             ..........C...A................................T..................-...
Sus scrofa(AF417224)             ..........C...A................................T..................-...
Sus scrofa(AY312521)             ..........CC..A................................T..................-...
Sus scrofa(AJ279056)             ..........CC..A................................T..................-...
Sus scrofa(AY312523)             ..........CC..A................................T..................-...
Sus scrofa(AY368589)             ..........C...A................................T..................-...
Sus scrofa(AF296168)             ..........C....C..C...............TG...........T..T..CAC.AT.T.....-...
Sus scrofa(AF417226)             ..........C...A................................T..................-...
Sus scrofa(Y12238)               ..........C...A................................T..................-...
4 Sus scrofa A                   ..........C....G...............................T....G.............-...
25 Sus scrofa A                  ..........C...A................................T..................-...
6 Sus celebensis A               ..........C....................................T..................C...
8 Sus celebensis A               ..........C....C..C...............TG...G...T...T..T..CAC.AT.T.....-...
1 Potamochoerus larvatus C       ..........C...TC...................G......T....T.....C........T...-...
```

6 *Potamochoerus larvatus* C	..........C...TC..........T.........A..........T..T..C.....A......-...
9 *Potamochoerus larvatus* C	..........C...TC..........T....................T..A..C.....A......-...
3 *Potamochoerus porcus* C	..........C...TC..........T....................T..T..C.....A......-A..
4 *Potamochoerus porcus* A	..........C........................G...........T.....C............-...
5 *Potamochoerus porcus* A	..........C....................................T..................C...
19 *Potamochoerus porcus* A	..........C........................G......T....T.....CA.......T...-...
1 *Phacochoerus africanus* A	..........C....C..C.......T....................T..T..C....GA......-...
4 *Phacochoerus africanus* A	..........C....C..C.......T....................T..T..C....GA......--..
4 *Phacochoerus africanus* C	--...T..T..C....GA......-...
6 *Phacochoerus africanus* A	..........C....C..C.......T....................T..T..C....GA......-..T
7 *Phacochoerus africanus* C	..........C...TC..........T....................T..T..C....GA......-...
12 *Phacochoerus africanus* C	--...T..T..C....GA......-...
8 *Phacochoerus aethiopicus* C	..........C...TC..................TG...........T..T----------.....---T

80 90 100 110 120 130 140

....|....|....|....|....|....|....|....|....|....|....|....|....|....|

Seq1 Sscrofa8 chromosome8	**CCCGATGTACTCCGTGCTTACGGGTTTTATGTTTGCCCAGGACCCCCAAATAATGAAGAATATTGTGGAA**
Seq2 Sscrofa8 chromosome10	....................T...
Seq3 Sscrofa8 chromosome13	....
Sus scrofa(EU086224)	..T........................A..
Sus scrofa(EU086222)	..T........................A..
Sus scrofa(EU086220)	....
Sus scrofa(EU086219)	....
Sus scrofa(AJ288585)	.............................C..
Sus scrofa(AJ293656)	.............................C..
Sus scrofa(EU789636)	.............................C..
Sus scrofa(AF435967)	.............................C..
Sus scrofa(AY288779)	.............................C..
Sus scrofa(EU086221)	....
Sus scrofa(EF133960)	....
Sus scrofa(AY368582)	....
Sus scrofa(AY368580)	....
Sus scrofa(AF417222)	.............................C.................................C......
Sus scrofa(AF417225)	.............................C..
Sus scrofa(AY368587)	.............A...............C..
Sus scrofa(AY368581)	.............................C..
Sus scrofa(AY312524)	.............................C..
Sus scrofa(AY371067)	.............................C..
Sus scrofa(AY312526)	.............................C..
Sus scrofa(AY368588)	.............................C..
Sus scrofa(AF417223)	.............................C..
Sus scrofa(AF417224)	.............................C..
Sus scrofa(AY312521)	.............................C..
Sus scrofa(AJ279056)	.............................C..
Sus scrofa(AY312523)	.............................C..
Sus scrofa(AY368589)	.............................C..

```
Sus scrofa(AF296168)                  ......A............................T........................C.........
Sus scrofa(AF417226)                  .............................C........................................
Sus scrofa(Y12238)                    .............................C........................................
4 Sus scrofa A                        ......................................................................
25 Sus scrofa A                       .............................C........................................
6 Sus celebensis A                    ......................................................................
8 Sus celebensis A                    ......A............................T..............C......A............
1 Potamochoerus larvatus C            ......................................................................
6 Potamochoerus larvatus C            ......A............................T..G..................A............
9 Potamochoerus larvatus C            ......A............................T.....................A............
3 Potamochoerus porcus C              ...............A...................T.....................A............
4 Potamochoerus porcus A              ................................................G.....................
5 Potamochoerus porcus A              ......................................................................
19 Potamochoerus porcus A             ......................................................................
1 Phacochoerus africanus A            ......A............................T..G..................A............
4 Phacochoerus africanus A            ......A............................T..G..................A............
4 Phacochoerus africanus C            ......A............................T..G..................A............
6 Phacochoerus africanus A            ......A............................T..G..................A............
7 Phacochoerus africanus C            ......A............................T..G..................A............
12 Phacochoerus africanus C           ......A............................T..G..................A............
8 Phacochoerus aethiopicus C          .G....AG.A.....T..A................T..................A..A..........G.

                                              150       160       170       180       190       200       210
                                      ....|....|....|....|....|....|....|....|....|....|....|....|....|....|
Seq1 Sscrofa8 chromosome8             ATCCTCAGGATTTCTTTTGCAAGCAATGGAGCTGCGTAACTTCTAATGATGGGAATTGGAAATGGCCAGT
Seq2 Sscrofa8 chromosome10            ......................................................................
Seq3 Sscrofa8 chromosome13            ......................................................................
Sus scrofa(EU086224)                  ..........................G...........................................
Sus scrofa(EU086222)                  ..........................G...........................................
Sus scrofa(EU086220)                  ......................................................................
Sus scrofa(EU086219)                  ......................................................................
Sus scrofa(AJ288585)                  ...............................G......................................
Sus scrofa(AJ293656)                  ...................................A..................................
Sus scrofa(EU789636)                  ......................................................................
Sus scrofa(AF435967)                  ......................................................................
Sus scrofa(AY288779)                  ......................................................................
Sus scrofa(EU086221)                  ......................................................................
Sus scrofa(EF133960)                  ......................................................................
Sus scrofa(AY368582)                  .............................G.......................................C
Sus scrofa(AY368580)                  ......................................................................
Sus scrofa(AF417222)                  ......................................................................
Sus scrofa(AF417225)                  ......................................................................
Sus scrofa(AY368587)                  ......................................................................
Sus scrofa(AY368581)                  ...................................A..................................
Sus scrofa(AY312524)                  ...................................A..................................
Sus scrofa(AY371067)                  ...................................A..................................
```

```
Sus scrofa(AY312526)          ...................................A...............C..................
Sus scrofa(AY368588)          ......................................................................
Sus scrofa(AF417223)          ......................................................................
Sus scrofa(AF417224)          ......................................................................
Sus scrofa(AY312521)          ......................................................................
Sus scrofa(AJ279056)          ......................................................................
Sus scrofa(AY312523)          ......................................................................
Sus scrofa(AY368589)          ......................................................................
Sus scrofa(AF296168)          ....CAG................A..........T.....C............T..............AC
Sus scrofa(AF417226)          ..............C.......................................................
Sus scrofa(Y12238)            ...................................A..................................
4 Sus scrofa A                ......................................................................
25 Sus scrofa A               ......................................................................
6 Sus celebensis A            ......................................................................
8 Sus celebensis A            ....CAG................G..........T.....C.......G.A..T...AA.........AC
1 Potamochoerus larvatus C    ........................................C.............................
6 Potamochoerus larvatus C    ....CGG...........A....A...A......T.....C..C.....C...T....A.........AC
9 Potamochoerus larvatus C    ....CAG................A..........T.....C............T..............AC
3 Potamochoerus porcus C      .....GG...........................T.....C.......G....T..............AC
4 Potamochoerus porcus A      ........................................C.............................
5 Potamochoerus porcus A      ......................................................................
19 Potamochoerus porcus A     ...........................A............C.............................
1 Phacochoerus africanus A    ....CGG................A..........T.....C..C.....C...T..............AC
4 Phacochoerus africanus A    ....CGG................A..........T.....C..C.....C...T..............AC
4 Phacochoerus africanus C    ....CGG................A..C.......T.....C..C.....C...T..............AC
6 Phacochoerus africanus A    ....CGG................A..........T.....C..C.....C...T..............AC
7 Phacochoerus africanus C    ....CGG................A..C.......T.....C..C.....C...T..............AC
12 Phacochoerus africanus C   ....CGG................A..C.......T.....C..C.....C...T..............AC
8 Phacochoerus aethiopicus C  ...TCGG................A...A......T..................T..............AC

                                     220       230       240       250       260       270       280
                              ....|....|....|....|....|....|....|....|....|....|....|....|....|....|
Seq1 Sscrofa8 chromosome8     CTCTCAGCAAGACAGAGTAAGTTACTCTTTTGTTAACAATCCTACCAGTTATAATCAATTTAA-------
Seq2 Sscrofa8 chromosome10    ...............................................................-------
Seq3 Sscrofa8 chromosome13    ...............................................................-------
Sus scrofa(EU086224)          ......................................G........................-------
Sus scrofa(EU086222)          ......................................G........................-------
Sus scrofa(EU086220)          ...............................................................-------
Sus scrofa(EU086219)          ...............................................................-------
Sus scrofa(AJ288585)          ...............................................................-------
Sus scrofa(AJ293656)          ...............................................................-------
Sus scrofa(EU789636)          ...............................................................-------
Sus scrofa(AF435967)          ...............................................................-------
Sus scrofa(AY288779)          ...............................................................-------
Sus scrofa(EU086221)          ...............................................................-------
Sus scrofa(EF133960)          ................T..............................................-------
```

```
Sus scrofa(AY368582)            ...............................................................-------
Sus scrofa(AY368580)            ...............................................................-------
Sus scrofa(AF417222)            ...............................................................-------
Sus scrofa(AF417225)            ...............................................................-------
Sus scrofa(AY368587)            ...............................................................-------
Sus scrofa(AY368581)            ...............................................................-------
Sus scrofa(AY312524)            ...............................................................-------
Sus scrofa(AY371067)            ...............................................................-------
Sus scrofa(AY312526)            ...............................................................-------
Sus scrofa(AY368588)            ...............................................................-------
Sus scrofa(AF417223)            ...............................................................-------
Sus scrofa(AF417224)            ...............................................................-------
Sus scrofa(AY312521)            ...............................................................-------
Sus scrofa(AJ279056)            ...............................................................-------
Sus scrofa(AY312523)            ...............................................................-------
Sus scrofa(AY368589)            ...............................................................-------
Sus scrofa(AF296168)            .....G......T..........T..............G.TA.....................-------
Sus scrofa(AF417226)            ...............................................................-------
Sus scrofa(Y12238)              ...............................................................-------
4 Sus scrofa A                  ...............................................................-------
25 Sus scrofa A                 ...................................G...........................-------
6 Sus celebensis A              ...............................................................-------
8 Sus celebensis A              .....G......T..G.......T.........C......TA........C.G.....C....-------
1 Potamochoerus larvatus C      .....................................G...........A..C..........-------
6 Potamochoerus larvatus C      .....G......T...AA.....TT........C.G..G.TA..TTGA.A.A..CA....A.GGTGATAT
9 Potamochoerus larvatus C      .....G......T..G.......T..............G.TA.....................-------
3 Potamochoerus porcus C        .....G......T..G.......T.........C....G.TA.......A.............-------
4 Potamochoerus porcus A        ......................................G........................-------
5 Potamochoerus porcus A        ...............................................................-------
19 Potamochoerus porcus A       ....................................................C..........-------
1 Phacochoerus africanus A      .....G......T...A......TT........C.G..G.TA.GTTGA.A.A..CA....A.GGCGATAT
4 Phacochoerus africanus A      .....G......T...A......TT........C.G..G.TA.GTTGA.A.A..CA....A.GGCGATAT
4 Phacochoerus africanus C      .....G......T...AC.....TT........C.G..G.TA.GTTGA.A.A..CA....A.GGCGATAT
6 Phacochoerus africanus A      .....G......T...A......TT........C.G..G.TA.GTTGA.A.A..CA....A.GGCGATAT
7 Phacochoerus africanus C      .....G......T...AC.....TT........C.G..G.TA.GTTGA.A.A..CA....A.GGCGATAT
12 Phacochoerus africanus C     .....G......T...AC.....TT........C.G..G.TA.GTTGA.A.A..CA....A.GGCGATAT
8 Phacochoerus aethiopicus C    .....G......T..G..--...T........CC......TA........C.G...G...C..-------

                                       290       300       310       320       330       340       350
                                ....|....|....|....|....|....|....|....|....|....|....|....|....|....|
Seq1 Sscrofa8 chromosome8       --------TTATGGCCATGGGAGATGGAAAGATTGGCAACAGCGGGTACAAAAAGATGTACGAAATAAGC
Seq2 Sscrofa8 chromosome10      --------...CC.........A....................A..........................
Seq3 Sscrofa8 chromosome13      --------..............................................................
Sus scrofa(EU086224)            --------..............................................................
Sus scrofa(EU086222)            --------..............................................................
Sus scrofa(EU086220)            --------..............................................................
```

Sus scrofa(EU086219)	---------..
Sus scrofa(AJ288585)	---------..
Sus scrofa(AJ293656)	---------..
Sus scrofa(EU789636)	---------..
Sus scrofa(AF435967)	---------..
Sus scrofa(AY288779)	---------..
Sus scrofa(EU086221)	---------..
Sus scrofa(EF133960)	---------..
Sus scrofa(AY368582)	---------..
Sus scrofa(AY368580)	---------..
Sus scrofa(AF417222)	---------..
Sus scrofa(AF417225)	---------..
Sus scrofa(AY368587)	---------..
Sus scrofa(AY368581)	---------..
Sus scrofa(AY312524)	---------..
Sus scrofa(AY371067)	---------..
Sus scrofa(AY312526)	---------..
Sus scrofa(AY368588)	---------..
Sus scrofa(AF417223)	---------.....................G..
Sus scrofa(AF417224)	---------..
Sus scrofa(AY312521)	---------..
Sus scrofa(AJ279056)	---------..
Sus scrofa(AY312523)	---------..
Sus scrofa(AY368589)	---------..
Sus scrofa(AF296168)	---------...C..........................GAGA.A.AC......CA------------G..
Sus scrofa(AF417226)	---------..
Sus scrofa(Y12238)	---------..
4 *Sus scrofa* A	---------..
25 *Sus scrofa* A	---------..A.........
6 *Sus celebensis* A	---------..
8 *Sus celebensis* A	---------....---------------TT.......------------..G.G...A.G.AA.T....A.
1 *Potamochoerus larvatus* C	---------...............G..
6 *Potamochoerus larvatus* C	GGCACTGTG.T...........GA.C..TTT.....AA....A...A....------------...C.GA.
9 *Potamochoerus larvatus* C	---------..............A.................GA...........G..C.C..A...C.G..
3 *Potamochoerus porcus* C	---------..............A.................GA...........G..C.C..A...C.G..
4 *Potamochoerus porcus* A	---------..
5 *Potamochoerus porcus* A	---------..
19 *Potamochoerus porcus* A	---------...............G..
1 *Phacochoerus africanus* A	GGCACTGTG.C...........GA.CA.TTT......A....A...A....------------...C.G..
4 *Phacochoerus africanus* A	GGCACTGTG.C...........GA.CA.TTT......A....A...A....------------...C.G..
4 *Phacochoerus africanus* C	GGCACTGTG.C...........GA.CA.TTT......A....A...A....------------..GC.G..
6 *Phacochoerus africanus* A	GGCACTGTG.C...........GA.CA.TTT......A....A...A....------------...C.G..
7 *Phacochoerus africanus* C	GGCACTGTG.C...........GA.CA.TTT......A....A...A....------------..GC.G..
12 *Phacochoerus africanus* C	GGCACTGTG.C...........GA.CA.TTT......A....A...A....------------..GC.G..
8 *Phacochoerus aethiopicus* C	---------.....---------------T.......------------..G.G...A.G.AA.....TA.

Sequence	360 370 380 390 400 410 420
	`....|....|....|....|....|....|....|....|....|....|....|....|....|....|`
Seq1 Sscrofa8 chromosome8	**AAATAAGCTGTCATTCGTTAGACCTAGATTACTTAAAAATAAGTTTCACTGAAAAAGGAAAACAAGAAAA**
Seq2 Sscrofa8 chromosome10	...........A...T..
Seq3 Sscrofa8 chromosome13	....
Sus scrofa(EU086224)	...........A..........T.......................T.......................
Sus scrofa(EU086222)	...........A..........T.......................T.......................
Sus scrofa(EU086220)	....
Sus scrofa(EU086219)	....
Sus scrofa(AJ288585)	..............C...
Sus scrofa(AJ293656)	....
Sus scrofa(EU789636)	....
Sus scrofa(AF435967)	....C........
Sus scrofa(AY288779)	....
Sus scrofa(EU086221)	....
Sus scrofa(EF133960)	....
Sus scrofa(AY368582)	....
Sus scrofa(AY368580)	....
Sus scrofa(AF417222)	....
Sus scrofa(AF417225)	....
Sus scrofa(AY368587)	....
Sus scrofa(AY368581)	....
Sus scrofa(AY312524)	....
Sus scrofa(AY371067)	....
Sus scrofa(AY312526)	....
Sus scrofa(AY368588)	....
Sus scrofa(AF417223)	....
Sus scrofa(AF417224)	....
Sus scrofa(AY312521)	....C........
Sus scrofa(AJ279056)	....C........
Sus scrofa(AY312523)	....C........
Sus scrofa(AY368589)	....
Sus scrofa(AF296168)	..........CTCCC.A.C.............C.G............................G......
Sus scrofa(AF417226)	....
Sus scrofa(Y12238)	....
4 *Sus scrofa* A	...........................G..
25 *Sus scrofa* A	....T...
6 *Sus celebensis* A	....
8 *Sus celebensis* A	.......T..CTC.C...C...............G........................G..........
1 *Potamochoerus larvatus* C	...........A..............................C...............T...........
6 *Potamochoerus larvatus* C	..G.......CA.CGTA.C.....C........................C....................
9 *Potamochoerus larvatus* C	..........CG.C.T..C...............G.....T........C....................
3 *Potamochoerus porcus* C	..G.......AA.C.TA.C.....................--.......CA...................
4 *Potamochoerus porcus* A	...C.......A..............................C...............T...........
5 *Potamochoerus porcus* A	....
19 *Potamochoerus porcus* A	...........A..............................C...............T...........

1 *Phacochoerus africanus* A	..G.G.....CA.CGTA.C.....C.........G..............C....................
4 *Phacochoerus africanus* A	..G.G.....CA.CGTA.C.....C.........G..............C....................
4 *Phacochoerus africanus* C	..G.G.....CA.CGTA.C.....C.........G..............C....................
6 *Phacochoerus africanus* A	..G.G.....CA.CGTA.C.....C.........G..............C....................
7 *Phacochoerus africanus* C	..G.G.....CA.CGTA.C.....C.........G..............C....................
12 *Phacochoerus africanus* C	..G.G.....CA.CGTA.C.....C.........G..............C....................
8 *Phacochoerus aethiopicus* C	..........CTCCC...C.............C.G..G................................
	430 440 450 460 470 480 490
	....|....|....|....|....|....|....|....|....|....|....|....|....|....|
Seq1 Sscrofa8 chromosome8	**TATTCAAAAGTGGGTAAATGGTATGTCTTGGGGAATAGTGTACTATGGAGGCTCTGGGAGAAAGAAAGGA**
Seq2 Sscrofa8 chromosome10	....G.......
Seq3 Sscrofa8 chromosome13	....
Sus scrofa(EU086224)	.....................................A..............A.........G.......
Sus scrofa(EU086222)	.....................................A.....CC.......A.........G.......
Sus scrofa(EU086220)	....
Sus scrofa(EU086219)	....
Sus scrofa(AJ288585)	....
Sus scrofa(AJ293656)	........................A...
Sus scrofa(EU789636)	....
Sus scrofa(AF435967)	....
Sus scrofa(AY288779)	....
Sus scrofa(EU086221)	....
Sus scrofa(EF133960)	....C....................
Sus scrofa(AY368582)	....
Sus scrofa(AY368580)	....
Sus scrofa(AF417222)	....
Sus scrofa(AF417225)	....
Sus scrofa(AY368587)	....A..........
Sus scrofa(AY368581)	........................A...
Sus scrofa(AY312524)	........................A...
Sus scrofa(AY371067)	........................A...
Sus scrofa(AY312526)	........................A...
Sus scrofa(AY368588)	....
Sus scrofa(AF417223)	....
Sus scrofa(AF417224)	....
Sus scrofa(AY312521)	....
Sus scrofa(AJ279056)	....
Sus scrofa(AY312523)	....
Sus scrofa(AY368589)	....
Sus scrofa(AF296168)	.............A....................G...........A........T..G..GGAG.....
Sus scrofa(AF417226)	....
Sus scrofa(Y12238)	........................A...
4 *Sus scrofa* A	...G......................T...
25 *Sus scrofa* A	....
6 *Sus celebensis* A	....

```
8 Sus celebensis A               .....G.......A.........................T.T....A..........---.G.AG..A..
1 Potamochoerus larvatus C       ......G...............................CA...................A..G.......
6 Potamochoerus larvatus C       .....T.......A...............A...GG...........A...........G..GGAG.....
9 Potamochoerus larvatus C       .............A....................G...........A...........G..GGAG.....
3 Potamochoerus porcus C         .............A....................G...........A...........G..GGAG.....
4 Potamochoerus porcus A         ...........T..........................CA...................A..G.......
5 Potamochoerus porcus A         ......................................................................
19 Potamochoerus porcus A        ......................................CA...................A..G.......
1 Phacochoerus africanus A       .....T.......A...................GG...........A...........G..GGAG.....
4 Phacochoerus africanus A       .....T.......A...................GG...........A...........G..GGAG.....
4 Phacochoerus africanus C       .....T.......A...................GG...........A...........G..GGAG.....
6 Phacochoerus africanus A       .....T.......A...................GG...........A...........G..GGAG.....
7 Phacochoerus africanus C       .....T.......A...................GG...........A...........G..GGAG.....
12 Phacochoerus africanus C      .....T.......A...................GG...........A...........G..GGAG.....
8 Phacochoerus aethiopicus C     .....G.......A....................G...........A......T.T..G..GG.......

                                          500       510       520       530       540       550       560
                                 ....|....|....|....|....|....|....|....|....|....|....|....|....|....|
Seq1 Sscrofa8 chromosome8        TCTGTTCTGACTATTCGCCTCAGAATAGAAACTCAGATGGAACCTCCGGTTGCTATAGGACCAAATAAGG
Seq2 Sscrofa8 chromosome10       ...A....A............................................................T
Seq3 Sscrofa8 chromosome13       ................................................A.....................
Sus scrofa(EU086224)             ...............T......................................................
Sus scrofa(EU086222)             ......................................................................
Sus scrofa(EU086220)             ................................................A.....................
Sus scrofa(EU086219)             ................................................A.....................
Sus scrofa(AJ288585)             ......................................................................
Sus scrofa(AJ293656)             ......................................................................
Sus scrofa(EU789636)             ......................................................................
Sus scrofa(AF435967)             ......................................................................
Sus scrofa(AY288779)             ......................................................................
Sus scrofa(EU086221)             .....C................................................................
Sus scrofa(EF133960)             ..........................................G...........................
Sus scrofa(AY368582)             ................................................A.....................
Sus scrofa(AY368580)             ................................................A.....................
Sus scrofa(AF417222)             ......................................................................
Sus scrofa(AF417225)             ......................A....A..........................................
Sus scrofa(AY368587)             ......................................................................
Sus scrofa(AY368581)             ......................................................................
Sus scrofa(AY312524)             ......................................................................
Sus scrofa(AY371067)             ......................................................................
Sus scrofa(AY312526)             ......................................................................
Sus scrofa(AY368588)             ......................................................................
Sus scrofa(AF417223)             ...........................A..........................................
Sus scrofa(AF417224)             ......................................................................
Sus scrofa(AY312521)             ......................................................................
Sus scrofa(AJ279056)             ......................................................................
```

```
Sus scrofa(AY312523)          ......................................................................
Sus scrofa(AY368589)          ......................................................................
Sus scrofa(AF296168)          ...A....A.............................................G...............
Sus scrofa(AF417226)          ..............................G.......................................
Sus scrofa(Y12238)            ......................................................................
4 Sus scrofa A                ......................................................................
25 Sus scrofa A               ......................................................................
6 Sus celebensis A            ..........................................G...........................
8 Sus celebensis A            ...A....A.............................................G.......G.......
1 Potamochoerus larvatus C    ........A........T.C...........................................G......
6 Potamochoerus larvatus C    ...AC...A....................C.......CAAT...C........------------.....
9 Potamochoerus larvatus C    ...A....A....................C.........A..............G.......G.......
3 Potamochoerus porcus C      ...A....A....................C........................G......TG.......
4 Potamochoerus porcus A      ........A........T.............................................G......
5 Potamochoerus porcus A      ..........................................G...........................
19 Potamochoerus porcus A     ........A.......AT.............................................G......
1 Phacochoerus africanus A    ...A....A....................C......GCAAT.T.C........------------.....
4 Phacochoerus africanus A    ...A....A....................C......GCAAT.T.C........------------.....
4 Phacochoerus africanus C    ...A....A...............G....C......GCAAT.T.C........------------.....
6 Phacochoerus africanus A    ...A....A....................C......GCAAT.T.C........------------.....
7 Phacochoerus africanus C    ...A....A...............G....C......GCAAT.T.C........------------.....
12 Phacochoerus africanus C   ...A....A...............G....C......GCAAT.T.C........------------.....
8 Phacochoerus aethiopicus C  ...A....A.........................T...................G.......G.......

                                      570       580       590       600       610       620       630
                              ....|....|....|....|....|....|....|....|....|....|....|....|....|....|
Seq1 Sscrofa8 chromosome8     GTTTGGCCGAACAAGGACCTCCAATCCAAGAACAGAGGCCATCTCCTAACCCCTCTGATTACAATACAAC
Seq2 Sscrofa8 chromosome10    .............................................T......................G.
Seq3 Sscrofa8 chromosome13    ......................................................................
Sus scrofa(EU086224)          .......T.................................................T............
Sus scrofa(EU086222)          .......T.................................................T............
Sus scrofa(EU086220)          ......................................................................
Sus scrofa(EU086219)          ......................................................................
Sus scrofa(AJ288585)          ...............................................G......................
Sus scrofa(AJ293656)          ......................................................................
Sus scrofa(EU789636)          ......................................................................
Sus scrofa(AF435967)          ......................................................................
Sus scrofa(AY288779)          ......................................................................
Sus scrofa(EU086221)          ......................................................................
Sus scrofa(EF133960)          ........A.............................................................
Sus scrofa(AY368582)          ......................................................................
Sus scrofa(AY368580)          ......................................................................
Sus scrofa(AF417222)          .....A............................A...................................
Sus scrofa(AF417225)          ......................................................................
Sus scrofa(AY368587)          ......................................................................
Sus scrofa(AY368581)          ......................................................................
```

```
Sus scrofa(AY312524)                ......................................................................
Sus scrofa(AY371067)                ......................................................................
Sus scrofa(AY312526)                ......................................................................
Sus scrofa(AY368588)                .............................A........................................
Sus scrofa(AF417223)                ......................................................................
Sus scrofa(AF417224)                ......................................................................
Sus scrofa(AY312521)                ......................................................................
Sus scrofa(AJ279056)                ......................................................................
Sus scrofa(AY312523)                ......................................................................
Sus scrofa(AY368589)                ......................................................................
Sus scrofa(AF296168)                TG..AA.T......................G..................T..........T.........
Sus scrofa(AF417226)                ......................................................................
Sus scrofa(Y12238)                  ......................................................................
4 Sus scrofa A                      ......................................................................
25 Sus scrofa A                     ......................................................................
6 Sus celebensis A                  ......................................................................
8 Sus celebensis A                  TA..A............................................T..........T.........
1 Potamochoerus larvatus C          ...................................................................G..
6 Potamochoerus larvatus C          TA..A..T.................................C.C.....T....T.....T.........
9 Potamochoerus larvatus C          TA..A..T................................GC.C.....T..........T.........
3 Potamochoerus porcus C            TA..A..T.................T..............GC.C.....T..........T.........
4 Potamochoerus porcus A            ...................................................................G..
5 Potamochoerus porcus A            ......................................................................
19 Potamochoerus porcus A           ...................................................................G..
1 Phacochoerus africanus A          TA..A..T.................................C.C.....T....T.....T.........
4 Phacochoerus africanus A          TA..A..T.................................C.C.....T....T.....T.........
4 Phacochoerus africanus C          TA..A..T.................................C.C.....T....T.....T.........
6 Phacochoerus africanus A          TA..A..T.................................C.C.....T....T.....T.........
7 Phacochoerus africanus C          TA..A..T.................................C.C.....T....T.....T.........
12 Phacochoerus africanus C         TA..A..T.................................C.C.....T....T.....T.........
8 Phacochoerus aethiopicus C        TA..AA........................G..................T..........T.........

                                            640       650       660       670       680       690       700
                                    ....|....|....|....|....|....|....|....|....|....|....|....|....|....|
Seq1 Sscrofa8 chromosome8           CTCTGGATCAGTCCCCACTGAGCCTAACATCACTA-TTAAAACAGGGGCGAAACTTTTTAGCCTCATCCA
Seq2 Sscrofa8 chromosome10          ...................................-.............A....................
Seq3 Sscrofa8 chromosome13          ...................................-..................................
Sus scrofa(EU086224)                ...................................-..................................
Sus scrofa(EU086222)                ...................................-..................................
Sus scrofa(EU086220)                ...................................-..................................
Sus scrofa(EU086219)                ...................................-..................................
Sus scrofa(AJ288585)                ...................................-........................A.........
Sus scrofa(AJ293656)                ...................................-..................................
Sus scrofa(EU789636)                ...................................-........................A.........
Sus scrofa(AF435967)                ...................................-........................A.........
Sus scrofa(AY288779)                ...................................-........................A.........
```

```
Sus scrofa(EU086221)              ...................................-..................................
Sus scrofa(EF133960)              .............T.....................-..................C...............
Sus scrofa(AY368582)              ...................................-..................................
Sus scrofa(AY368580)              ...................................-..................................
Sus scrofa(AF417222)              ...................................-..................................
Sus scrofa(AF417225)              ...................................-..................................
Sus scrofa(AY368587)              ...................................-........................A.........
Sus scrofa(AY368581)              ...................................-......................C...........
Sus scrofa(AY312524)              ...................................-..................................
Sus scrofa(AY371067)              ...................................-..................................
Sus scrofa(AY312526)              ...................................-..................................
Sus scrofa(AY368588)              ...................................-........................A.........
Sus scrofa(AF417223)              ...................................-........................A.........
Sus scrofa(AF417224)              ...................................-..................................
Sus scrofa(AY312521)              ...................................-........................A.........
Sus scrofa(AJ279056)              ...................................-........................A.........
Sus scrofa(AY312523)              ...................................-........................A.........
Sus scrofa(AY368589)              ...................................-........................A.........
Sus scrofa(AF296168)              .....................A......G..C..G-...........CA..G...C..............
Sus scrofa(AF417226)              ...................................-........................A.........
Sus scrofa(Y12238)                ...................................-..................................
4 Sus scrofa A                    ...................................-..................................
25 Sus scrofa A                   ...................................-..................................
6 Sus celebensis A                ...................................-..................................
8 Sus celebensis A                .....................A......G..C..G-...........CA..G...C..............
1 Potamochoerus larvatus C        ................T..................-....T........A....................
6 Potamochoerus larvatus C        .....AG..............A.............-...........CA..GG..C..C...........
9 Potamochoerus larvatus C        ..TC.A...............A......G......T...........CA..GG..C..C...........
3 Potamochoerus porcus C          .....A...............A......G......-...........CA..GG..C..C...........
4 Potamochoerus porcus A          ................T..................-.............A....................
5 Potamochoerus porcus A          ...................................-..................................
19 Potamochoerus porcus A         ................T..................-..................................
1 Phacochoerus africanus A        .....A...............A.............-..........ACA..GG..C..C...........
4 Phacochoerus africanus A        .....A...............A.............-..........ACA..GG..C..C...........
4 Phacochoerus africanus C        .....A...............A.............-..........ACA..GG.CC..C...........
6 Phacochoerus africanus A        .....A...............A.............-..........ACA..GG..C..C...........
7 Phacochoerus africanus C        .....A...............A.............-..........ACA..GG.CC..C...........
12 Phacochoerus africanus C       .....A...............A.............-..........ACA..GG.CC..C...........
8 Phacochoerus aethiopicus C      .....................A.........C..G-...........CA..G...C..............

                                          710       720       730       740       750       760       770
                                  ....|....|....|....|....|....|....|....|....|....|....|....|....|....|
Seq1 Sscrofa8 chromosome8         GGGAGCTT-TTCAAGCCC-TTAACTCCACGA-CTCCAGAGGCTACCTCTTCTTGTTGGCTTTGCTTAGCT
Seq2 Sscrofa8 chromosome10        ........-.......T.-..........A.-......................................
Seq3 Sscrofa8 chromosome13        ........-.......T.-.........A..-......................................
Sus scrofa(EU086224)              ........-.........-............-......................................
```

```
Sus scrofa(EU086222)            ........-.........-............-......................................
Sus scrofa(EU086220)            ........-.......T.-.........A..-......................................
Sus scrofa(EU086219)            ........-.......T.-.........A..-......................................
Sus scrofa(AJ288585)            ........-.......T.-............-......................................
Sus scrofa(AJ293656)            ........-.......T.-............-......................................
Sus scrofa(EU789636)            ........-.......T.-............-......................................
Sus scrofa(AF435967)            ........-.......T.-............-......................................
Sus scrofa(AY288779)            ........-.......T.-............-......................................
Sus scrofa(EU086221)            ........-.........-............-......................................
Sus scrofa(EF133960)            ........-.......T.-............-......................................
Sus scrofa(AY368582)            ........-.......T.-.........A..-......................................
Sus scrofa(AY368580)            ........-.......T.-.........A..-......................................
Sus scrofa(AF417222)            ........-.......T.-..........A.-.....A................................
Sus scrofa(AF417225)            ........-.......T.-............-......................................
Sus scrofa(AY368587)            ........-.......T.-............-......................................
Sus scrofa(AY368581)            ........-.......T.-............-......................................
Sus scrofa(AY312524)            ........-.......T.-............-......................................
Sus scrofa(AY371067)            ........-.......T.-............-......................................
Sus scrofa(AY312526)            ........-.......T.-............-......................................
Sus scrofa(AY368588)            ........-.......T.-............-......................................
Sus scrofa(AF417223)            ........-.......T.-............-...........................C..........
Sus scrofa(AF417224)            ........-.......T.-............-......................................
Sus scrofa(AY312521)            ........-.......T.-............-......................................
Sus scrofa(AJ279056)            ........-.......T.-............-......................................
Sus scrofa(AY312523)            ........-.......T.-............-......................................
Sus scrofa(AY368589)            ........-.......T.-............-......................................
Sus scrofa(AF296168)            ........-.......T.-............-......................................
Sus scrofa(AF417226)            ........-.......T.-............-......................................
Sus scrofa(Y12238)              ........-.......T.-............-......................................
4 Sus scrofa A                  ........-.......T.-............-......................................
25 Sus scrofa A                 ........-.........-............-.....................................C
6 Sus celebensis A              ........-.......T.-............-....C.................................
8 Sus celebensis A              ........-.......T.-..........A.-...............................TC....C
1 Potamochoerus larvatus C      ........-.......T.-............-......................................
6 Potamochoerus larvatus C      ........-.........-............-G...G..T..C..A........C.....C..TC....C
9 Potamochoerus larvatus C      .....T..C.........T............GT......T..C..A---....X..A..--..TC....C
3 Potamochoerus porcus C        ......C.-.........-............-G......T..C..A........C...T.C..TC....C
4 Potamochoerus porcus A        ........-.......T.-............-..............................A.......
5 Potamochoerus porcus A        ........-.......T.-............-....C.................................
19 Potamochoerus porcus A       ........-.......T.-............-......................................
1 Phacochoerus africanus A      ........-.........-............-G......T..C..A........C.....C..TC....C
4 Phacochoerus africanus A      ........-.........-............-G......T..C..A........C.....C..TC....C
4 Phacochoerus africanus C      ........-.........-............-G......T..C..A........C.....C..TC....C
6 Phacochoerus africanus A      ........-.........-............-G......T..C..A........C.....C..TC....C
7 Phacochoerus africanus C      ........-.........-............-G......T..C..A........C.....C..TC....C
12 Phacochoerus africanus C     ........-.........-............-G......T..C..A........C.....C..TC....C
```

```
8 Phacochoerus aethiopicus C   ........-.......T.-.......A..A.-...............................TC....A

                                        780       790       800       810       820       830       840
                               ....|....|....|....|....|....|....|....|....|....|....|....|....|....|
Seq1 Sscrofa8 chromosome8      TCGGGCCCACCTTACTATG-AGGGAATGGCTAGAG-GAGGGAAATTC-AATGTGACAAAGGAACATAGAG
Seq2 Sscrofa8 chromosome10     ..A................-.-....C........-...........-......................
Seq3 Sscrofa8 chromosome13     ...................-...............-...........-......................
Sus scrofa(EU086224)           ...................-...............-...........-......................
Sus scrofa(EU086222)           ...................-...............-...........-......................
Sus scrofa(EU086220)           ...................-...............-...........-......................
Sus scrofa(EU086219)           ...................-...............-...........-......................
Sus scrofa(AJ288585)           ...................-............C..-...........-......................
Sus scrofa(AJ293656)           ...................-...............-...........-......................
Sus scrofa(EU789636)           ...................-...............-...........-......................
Sus scrofa(AF435967)           ...................-...............-...........-......................
Sus scrofa(AY288779)           ...................-...............-...........-......................
Sus scrofa(EU086221)           ...................-...............-...........-......................
Sus scrofa(EF133960)           ...................-...............-...........-......................
Sus scrofa(AY368582)           ...................-...............-...........-......................
Sus scrofa(AY368580)           ...................-...............-...........-......................
Sus scrofa(AF417222)           ...................-...............-...........-......................
Sus scrofa(AF417225)           ...................-...............-...........-......................
Sus scrofa(AY368587)           ...................-...............-...........-......................
Sus scrofa(AY368581)           ...................-...............-...........-......................
Sus scrofa(AY312524)           ...................-...............-...........-......................
Sus scrofa(AY371067)           ...................-...............-...........-......................
Sus scrofa(AY312526)           ...................-...............-...........-......................
Sus scrofa(AY368588)           ...................-...............-...........-........C.............
Sus scrofa(AF417223)           ...................-...............-...........-......................
Sus scrofa(AF417224)           ...................-...............-...........-......................
Sus scrofa(AY312521)           ...................-...............-...........-......................
Sus scrofa(AJ279056)           ...................-...............-...........-......................
Sus scrofa(AY312523)           ...................-...............-...........-.................C....
Sus scrofa(AY368589)           ...................-...............-...........-......................
Sus scrofa(AF296168)           ...................-...............-...........-......................
Sus scrofa(AF417226)           ...................-...............-...........-......................
Sus scrofa(Y12238)             ...................-...............-...........-......................
4 Sus scrofa A                 ...................-...............-...........-......................
25 Sus scrofa A                ...................-...............-...........-......................
6 Sus celebensis A             ...................-...............-...........-......................
8 Sus celebensis A             .................C.-...A.........G.-A..........-...........A..........
1 Potamochoerus larvatus C     ...................-...............-...........-......................
6 Potamochoerus larvatus C     .T...............C.-....C..........AA...A.....T-G..........A..........
9 Potamochoerus larvatus C     ...................-...............-A..........-...........A.......A..
3 Potamochoerus porcus C       ............A..A.C.-...AC..........-A..........-...........A..........
4 Potamochoerus porcus A       .T.................-.......C....A..-..A.A......-...........-..........
```

```
5 Potamochoerus porcus A           ...................-........C......-...........-......................
19 Potamochoerus porcus A          ...................-...............-....A......-......................
1 Phacochoerus africanus A         .................C.-....C..........-A.........T-G..........A..........
4 Phacochoerus africanus A         .................C.-....C..........-A.........T-G..........A..........
4 Phacochoerus africanus C         .................C.-....C..........-A.........T-G..........A..........
6 Phacochoerus africanus A         .................C.-....C..........-A.........T-G..........A..........
7 Phacochoerus africanus C         .................C.-....C..........-A.........T-G..........A..........
12 Phacochoerus africanus C        .................C.-....C..........-A.........T-G..........A..........
8 Phacochoerus aethiopicus C       ..........A........A...A...........AA.......C..A..C........AA.........

                                           850       860       870       880       890       900       910
                                   ....|....|....|....|....|....|....|....|....|....|....|....|....|....|
Seq1 Sscrofa8 chromosome8          ACCAATGTACATGGGGATCCC-AAAAT-AAGCTTACCCTTACTGAGGTTTCTG-GAAAAGGCACCTGCAT
Seq2 Sscrofa8 chromosome10         .....................-.....-.........................-................
Seq3 Sscrofa8 chromosome13         .....................-.....-.........................-................
Sus scrofa(EU086224)               .....................-.....-.........................-................
Sus scrofa(EU086222)               .....................-.....-.........................-................
Sus scrofa(EU086220)               .....................-.....-.........................-................
Sus scrofa(EU086219)               .....................-.....-.........................-................
Sus scrofa(AJ288585)               .....................-.....-.........................-................
Sus scrofa(AJ293656)               .....................-.....-.........................-................
Sus scrofa(EU789636)               .....................-.....-.........................-.......T........
Sus scrofa(AF435967)               .....................-.....-.........................-................
Sus scrofa(AY288779)               .....................-.....-.........................-................
Sus scrofa(EU086221)               .....................-.....-.........................-................
Sus scrofa(EF133960)               .....................-.....-.........................-................
Sus scrofa(AY368582)               .....................-.....-.........................-................
Sus scrofa(AY368580)               .....................-.....-.........................-................
Sus scrofa(AF417222)               .....................-.....-......................T..-................
Sus scrofa(AF417225)               .....................-.....-.........................-................
Sus scrofa(AY368587)               .....................-C....-.........................-................
Sus scrofa(AY368581)               .....................-.....-.........................-...........CA...
Sus scrofa(AY312524)               .....................-.....-.........................-................
Sus scrofa(AY371067)               .....................-.....-.........................-................
Sus scrofa(AY312526)               .....................-.....-.........................-................
Sus scrofa(AY368588)               .....................-.....-.........................-................
Sus scrofa(AF417223)               .....................-.....-.........................-................
Sus scrofa(AF417224)               .....................-.....-.........................-................
Sus scrofa(AY312521)               ............C........-.....-.........................-................
Sus scrofa(AJ279056)               .....................-.....-.........................-................
Sus scrofa(AY312523)               .....................-.....-.........................-................
Sus scrofa(AY368589)               .....................-.....-.........................-................
Sus scrofa(AF296168)               .....................-.....-.......................C.-................
Sus scrofa(AF417226)               .....................-.....-.........................-................
Sus scrofa(Y12238)                 .....................-.....-.........................-................
4 Sus scrofa A                     .....................-.....-.........................-................
```

```
25 Sus scrofa A                     .....................-.....-.........................-................
6 Sus celebensis A                  .....................-.....-........T................-................
8 Sus celebensis A                  .T...................-.....-...........CG............-..........TACA..
1 Potamochoerus larvatus C          .....................A.....-.........................-................
6 Potamochoerus larvatus C          .T......G............G.....-.................A.......AA...............
9 Potamochoerus larvatus C          .T...................-.....-.........................-................
3 Potamochoerus porcus C            .T......G............-.....-.........................GA...............
4 Potamochoerus porcus A            .....................-.....-.........................-....--..........
5 Potamochoerus porcus A            .....................-.....-.........................-................
19 Potamochoerus porcus A           .....................-.....-.........................-................
1 Phacochoerus africanus A          .T......G............-G....-.................A.......-................
4 Phacochoerus africanus A          .T......G............-G....-.................A.......-................
4 Phacochoerus africanus C          .T......G............-G....-.................A.......-................
6 Phacochoerus africanus A          .T......G............-G....-.................A.......-................
7 Phacochoerus africanus C          .T......G............-G....-.................A.......-................
12 Phacochoerus africanus C         .T......G............-G....-.................A.......-................
8 Phacochoerus aethiopicus C        .T.............T.....-.....A........................A-................

                                            920       930       940       950       960       970       980
                                    ....|....|....|....|....|....|....|....|....|....|....|....|....|....|
Seq1 Sscrofa8 chromosome8           AGGG-AGGGTTCCCCCATCCCACC-AACACCTTTGTAACCACACTGAAGCCTTTAATCGAACCTCTGAGA
Seq2 Sscrofa8 chromosome10          ....-...................-.............................................
Seq3 Sscrofa8 chromosome13          ....-...................-.............................................
Sus scrofa(EU086224)                ....-...................-.............................................
Sus scrofa(EU086222)                ....-...................-.............................................
Sus scrofa(EU086220)                ....-...................-.............................................
Sus scrofa(EU086219)                ....-...................-.............................................
Sus scrofa(AJ288585)                ....-.T.................-.......................C.....................
Sus scrofa(AJ293656)                ....-.T.................-.............................................
Sus scrofa(EU789636)                ....-.T.................-....................A........................
Sus scrofa(AF435967)                ....-.T.................-.............................................
Sus scrofa(AY288779)                ....-.T.................-.............................................
Sus scrofa(EU086221)                ....-...................-.............................................
Sus scrofa(EF133960)                ....-...................-.............................................
Sus scrofa(AY368582)                ....-...................-.............................................
Sus scrofa(AY368580)                ....-...................-.............................................
Sus scrofa(AF417222)                ....-.T.................-.......................................T.....
Sus scrofa(AF417225)                ....-...................-.............................................
Sus scrofa(AY368587)                ....-.T.................-.............................................
Sus scrofa(AY368581)                ....-.T.................-.............................................
Sus scrofa(AY312524)                ....-.T.................-.............................................
Sus scrofa(AY371067)                ....-.T.................-.............................................
Sus scrofa(AY312526)                ....-.T.................-.............................................
Sus scrofa(AY368588)                ....-.T.................-C..........................................A.
Sus scrofa(AF417223)                ....-.T.................-.............................................
Sus scrofa(AF417224)                ....-.T.................-.............................................
```

```
Sus scrofa(AY312521)              ....-.T.................-.............................................
Sus scrofa(AJ279056)              ....-.T.................-.............................................
Sus scrofa(AY312523)              ....-.T.................-.............................................
Sus scrofa(AY368589)              ....-.T.................-.............................................
Sus scrofa(AF296168)              ....-.T.................-.............................................
Sus scrofa(AF417226)              ....-.T.................-.............................................
Sus scrofa(Y12238)                ....-.T.................-.............................................
4 Sus scrofa A                    ....-...................-.............................................
25 Sus scrofa A                   ....-...................-.............................................
6 Sus celebensis A                ....-...................-.............................................
8 Sus celebensis A                ....-...................-.............................................
1 Potamochoerus larvatus C        ....-.....C.............-.............................................
6 Potamochoerus larvatus C        ....A.A.................-....................A........................
9 Potamochoerus larvatus C        ....-...................A..........C..................................
3 Potamochoerus porcus C          ....-.A.T...............-.............................................
4 Potamochoerus porcus A          ....-.....C.............-..........................-..................
5 Potamochoerus porcus A          ....-...................-.............................................
19 Potamochoerus porcus A         ....-.....C.............-.............................................
1 Phacochoerus africanus A        ....-.A.................-...................C.........................
4 Phacochoerus africanus A        ....-.A.................-...................C.........................
4 Phacochoerus africanus C        ....-.A.................-...................C.........................
6 Phacochoerus africanus A        ....-.A.................-...G...............C.........................
7 Phacochoerus africanus C        ....-.A.................-...................C.........................
12 Phacochoerus africanus C       ....-.A.................-...................C.........................
8 Phacochoerus aethiopicus C      ....-..T.....T..........-.............................................

                                          990       1000      1010      1020      1030      1040      1050
                                  ....|....|....|....|....|....|....|....|....|....|....|....|....|....|
Seq1 Sscrofa8 chromosome8         GTC-AGTATCTGGTACCTGGTTATGACAGGTGGTGGGCATGTAATACTGGATTAACCCCTTGTGTTTCCA
Seq2 Sscrofa8 chromosome10        ...-.............C....................................................
Seq3 Sscrofa8 chromosome13        ...-.A................................................................
Sus scrofa(EU086224)              ...-..................................................................
Sus scrofa(EU086222)              ...-..................................................................
Sus scrofa(EU086220)              ...-.A................................................................
Sus scrofa(EU086219)              ...-.A................................................................
Sus scrofa(AJ288585)              ...-C.................................................................
Sus scrofa(AJ293656)              ...-.A................................................................
Sus scrofa(EU789636)              ...-..................................................................
Sus scrofa(AF435967)              ...A.....................-............................................
Sus scrofa(AY288779)              ...-..................................................................
Sus scrofa(EU086221)              ...-..................................................................
Sus scrofa(EF133960)              ...-..................................................................
Sus scrofa(AY368582)              ...-.A................................................................
Sus scrofa(AY368580)              ...-.A................................................................
Sus scrofa(AF417222)              ...-.....T............................................................
Sus scrofa(AF417225)              ...-..................................................................
```

```
Sus scrofa(AY368587)          ...-.A..............................A.................................
Sus scrofa(AY368581)          ...-.A...........................................................C....
Sus scrofa(AY312524)          ...-.A................................................................
Sus scrofa(AY371067)          ...-.A................................................................
Sus scrofa(AY312526)          ...-.A................................................................
Sus scrofa(AY368588)          ...-..................................................................
Sus scrofa(AF417223)          ...-..................................................................
Sus scrofa(AF417224)          ...-..................................................................
Sus scrofa(AY312521)          ...A.....................-............................................
Sus scrofa(AJ279056)          ...A.....................-............................................
Sus scrofa(AY312523)          ...A.....................-............................................
Sus scrofa(AY368589)          ...-..................................................................
Sus scrofa(AF296168)          ...-..................................................................
Sus scrofa(AF417226)          ...-..................................................................
Sus scrofa(Y12238)            ...-.A................................................................
4 Sus scrofa A                ...-..................................................................
25 Sus scrofa A               ...-..................................................................
6 Sus celebensis A            ...-....................................................-------------.
8 Sus celebensis A            A..-.............C....................................................
1 Potamochoerus larvatus C    ...-..................................................................
6 Potamochoerus larvatus C    AC.-........AC...C......A...........A.......C.......................T.
9 Potamochoerus larvatus C    AC.-.........C.............G................C.......................T.
3 Potamochoerus porcus C      AC.-.........C.T.C.........................GC.......................T.
4 Potamochoerus porcus A      ...-......................T...........................................
5 Potamochoerus porcus A      ...-....................................................-------------.
19 Potamochoerus porcus A     ...-..................................................................
1 Phacochoerus africanus A    AC.-.........C...C..........................C.......................T.
4 Phacochoerus africanus A    AC.-.........C...C..........................C.......................T.
4 Phacochoerus africanus C    AC.-.........C...C..........................C.......................T.
6 Phacochoerus africanus A    AC.-.........C...C..........................C.......................T.
7 Phacochoerus africanus C    AC.-.........C...C..............A...........C.......................T.
12 Phacochoerus africanus C   AC.-.........C...C..........................C.......................T.
8 Phacochoerus aethiopicus C  A..-......CA.....C.....C...............................................

                                      1060      1070      1080      1090      1100      1110      1120
                              ....|....|....|....|....|....|....|....|....|....|....|....|....|....|
Seq1 Sscrofa8 chromosome8     CCTTGGTTTTCAACCAAACTAAAGACTTTTGCTTTATGGTCCAAATTGTCCCCCGGGTGTACTACTATCC
Seq2 Sscrofa8 chromosome10    ..C.............................G.....................................
Seq3 Sscrofa8 chromosome13    .G.............................TG.....................................
Sus scrofa(EU086224)          ................................G....................T................
Sus scrofa(EU086222)          ................................G....................T................
Sus scrofa(EU086220)          .G.............................TG.....................................
Sus scrofa(EU086219)          .G.............................TG.....................................
Sus scrofa(AJ288585)          ................................G.............................C.......
Sus scrofa(AJ293656)          ................................G.....................................
Sus scrofa(EU789636)          ................................G.....................................
```

Sus scrofa(AF435967)	................................G.....................................
Sus scrofa(AY288779)	................................G.....................................
Sus scrofa(EU086221)	................................G....................T................
Sus scrofa(EF133960)	................................G.....................................
Sus scrofa(AY368582)	...............................TG.....................................
Sus scrofa(AY368580)	...............................TG.....................................
Sus scrofa(AF417222)	..........................C.....G.....................................
Sus scrofa(AF417225)	................................G.....................................
Sus scrofa(AY368587)	................................G.....................................
Sus scrofa(AY368581)	................................G.....................................
Sus scrofa(AY312524)	................................G.....................................
Sus scrofa(AY371067)	................................G.....................................
Sus scrofa(AY312526)	................................G.....................................
Sus scrofa(AY368588)	................................G.....................................
Sus scrofa(AF417223)	................................G.....................................
Sus scrofa(AF417224)	................................G.....................................
Sus scrofa(AY312521)	................................G.....................................
Sus scrofa(AJ279056)	................................G.....................................
Sus scrofa(AY312523)	................................G.....................................
Sus scrofa(AY368589)	................................G.....................................
Sus scrofa(AF296168)	................................G.....................................
Sus scrofa(AF417226)	................................G.....................................
Sus scrofa(Y12238)	................................G.....................................
4 *Sus scrofa* A	................................G.....................................
25 *Sus scrofa* A	................................G.....................................
6 *Sus celebensis* A	................................G.....................................
8 *Sus celebensis* A	..C.......TG............GT......G.....................A......T...C....
1 *Potamochoerus larvatus* C	..C.......T.....................G..................................C..
6 *Potamochoerus larvatus* C	..C.A..........C.......A.T....A.A.C.........C....................C....
9 *Potamochoerus larvatus* C	..C.A....................T......G.C.........C....................C....
3 *Potamochoerus porcus* C	..C.A..........C.........T......G.C.........C........T.........G.C....
4 *Potamochoerus porcus* A	..C.A...........................A..................................C..
5 *Potamochoerus porcus* A	................................G.....................................
19 *Potamochoerus porcus* A	..C...........................A.G..................................C..
1 *Phacochoerus africanus* A	..C.A..........C.........T......G.C...A.....C....................C....
4 *Phacochoerus africanus* A	..C.A..........C.........T......G.C...A.....C....................C....
4 *Phacochoerus africanus* C	..C.A..........C.........T......G.C...A.....C....................C....
6 *Phacochoerus africanus* A	..C.A..........C.........T......G.C...A.....C....................C....
7 *Phacochoerus africanus* C	..C.A..........C.........T......G.C...A.....C....................C....
12 *Phacochoerus africanus* C	..C.A..........C.........T......G.C...A.....C....................C....
8 *Phacochoerus aethiopicus* C	..C........G.............T......G................................C....

	1130 1140 1150 1160 1170 1180 1190
	....|....|....|....|....|....|....|....|....|....|....|....|....|....|
Seq1 Sscrofa8 chromosome8	CGAAAAAG-------CAGTCCTTGATGAATATGACTATAGATATAATCGGCCAAAAAGAGAGCCCATATC
Seq2 Sscrofa8 chromosome10	........-------.G...............................A............A........

Seq3 Sscrofa8 chromosome13	........-------................................T.............A........
Sus scrofa(EU086224)	........-------...
Sus scrofa(EU086222)	........-------...
Sus scrofa(EU086220)	........-------................................T.............A........
Sus scrofa(EU086219)	........-------................................T.............A........
Sus scrofa(AJ288585)	.......A-------..T
Sus scrofa(AJ293656)	........-------...
Sus scrofa(EU789636)	........-------...
Sus scrofa(AF435967)	........-------...
Sus scrofa(AY288779)	........-------...
Sus scrofa(EU086221)	........-------...
Sus scrofa(EF133960)	........-------...
Sus scrofa(AY368582)	........-------................................T.............C........
Sus scrofa(AY368580)	........-------..........G...................................A........
Sus scrofa(AF417222)	........-------...
Sus scrofa(AF417225)	........-------...
Sus scrofa(AY368587)	........-------...
Sus scrofa(AY368581)	........-------...
Sus scrofa(AY312524)	........-------...
Sus scrofa(AY371067)	........-------...
Sus scrofa(AY312526)	........-------...
Sus scrofa(AY368588)	........-------...
Sus scrofa(AF417223)	........-------...
Sus scrofa(AF417224)	........-------...
Sus scrofa(AY312521)	........-------...
Sus scrofa(AJ279056)	........-------...
Sus scrofa(AY312523)	........-------...
Sus scrofa(AY368589)	........-------...
Sus scrofa(AF296168)	........-------...
Sus scrofa(AF417226)	........-------...
Sus scrofa(Y12238)	........-------...
4 *Sus scrofa* A	........-------...
25 *Sus scrofa* A	........-------.G...
6 *Sus celebensis* A	........-------..A........
8 *Sus celebensis* A	........-------...........A............A........A..........A.A........
1 *Potamochoerus larvatus* C	........-------.G..A........
6 *Potamochoerus larvatus* C	T..T.C..-------...........A....G.....C........C....A..G...G..A........
9 *Potamochoerus larvatus* C	...T.C..-------...........A..........C........C....A......G..A..T.....
3 *Potamochoerus porcus* C	...T.C..-------..C.................C.C........C.A..A......G..A..T.....
4 *Potamochoerus porcus* A	.A......TATTTATT.......A..A.....................A..........A.A........
5 *Potamochoerus porcus* A	........-------..A........
19 *Potamochoerus porcus* A	........-------.G........C...................................A........
1 *Phacochoerus africanus* A	T..T.C..-------......................C........C....A......G..A........
4 *Phacochoerus africanus* A	TA.T.C..-------......................C........C....A......G..A........
4 *Phacochoerus africanus* C	T..T.C..-------......................C........C....A......G..A........
6 *Phacochoerus africanus* A	T..T.C..-------......................C........C....A......G..A........

```
7 Phacochoerus africanus C       T..T.C..-------......................C........C....A......G..A........
12 Phacochoerus africanus C      T..T.C..-------......................C........C....A......G..A........
8 Phacochoerus aethiopicus C     ........-------....................................A.........A...G....

                                         1200      1210      1220      1230      1240      1250      1260
                                 ....|....|....|....|....|....|....|....|....|....|....|....|....|....|
Seq1 Sscrofa8 chromosome8        CCTGACACTAGCTGTAATGCTTGGATTGGGAGTGGCTGCAGGCGTGGGAACAGGAACGGCTGCCCTAATC
Seq2 Sscrofa8 chromosome10       .....................C................................................
Seq3 Sscrofa8 chromosome13       .....................C................................................
Sus scrofa(EU086224)             .....................C................................................
Sus scrofa(EU086222)             .....................C................................................
Sus scrofa(EU086220)             .....................C................................................
Sus scrofa(EU086219)             .....................C................................................
Sus scrofa(AJ288585)             .....................C................................................
Sus scrofa(AJ293656)             .....................C................................................
Sus scrofa(EU789636)             .....................C................................................
Sus scrofa(AF435967)             .....................C................................................
Sus scrofa(AY288779)             .....................C................................................
Sus scrofa(EU086221)             .....................C................................................
Sus scrofa(EF133960)             .....................C..........................T.....................
Sus scrofa(AY368582)             .....................C................................................
Sus scrofa(AY368580)             .....................C................................................
Sus scrofa(AF417222)             .....................C................................................
Sus scrofa(AF417225)             .....................C.....................A..........................
Sus scrofa(AY368587)             .....................C.........A......................................
Sus scrofa(AY368581)             .....................C................................................
Sus scrofa(AY312524)             ...............C.....C................................................
Sus scrofa(AY371067)             .....................C................................................
Sus scrofa(AY312526)             .....................C................................................
Sus scrofa(AY368588)             .....................C................................................
Sus scrofa(AF417223)             .....................C................................................
Sus scrofa(AF417224)             .....................C................................................
Sus scrofa(AY312521)             .....................C......CC........................................
Sus scrofa(AJ279056)             .....................C................................................
Sus scrofa(AY312523)             .....................C................................................
Sus scrofa(AY368589)             .....................C................................................
Sus scrofa(AF296168)             .....................C.........A......................................
Sus scrofa(AF417226)             .....................C................................................
Sus scrofa(Y12238)               .....................C................................................
4 Sus scrofa A                   .....................C................................................
25 Sus scrofa A                  .....................C..................................T.............
6 Sus celebensis A               .....................C.....................A..........................
8 Sus celebensis A               ..............C......C...................................A............
1 Potamochoerus larvatus C       .....................C....................T...........................
6 Potamochoerus larvatus C       ......TT................G......A................G...........C.....G...
9 Potamochoerus larvatus C       .......T................G.....G.................G...........CA....G...
```

3 *Potamochoerus porcus* C	.......T.....A..........G.....G.................G...........C.....G...
4 *Potamochoerus porcus* A	.....................C....................T....A......................
5 *Potamochoerus porcus* A	.....................C.....................A..........................
19 *Potamochoerus porcus* A	.....................C....................T...........................
1 *Phacochoerus africanus* A	......TT................G.....GA................G...........C.....G...
4 *Phacochoerus africanus* A	......TT................G.....GA................G...........C.....G...
4 *Phacochoerus africanus* C	......TT................G.....GA................G...........C.....G...
6 *Phacochoerus africanus* A	......TT................G.....GA................G...........C.....G...
7 *Phacochoerus africanus* C	......TT................G.....GA................G...........C.....G...
12 *Phacochoerus africanus* C	......TT................G.....GA................G...........C.....G...
8 *Phacochoerus aethiopicus* C	.......T......C......C..............................A.................

	1270 1280 1290 1300 1310 1320 1330
	....|....|....|....|....|....|....|....|....|....|....|....|....|....|
Seq1 Sscrofa8 chromosome8	**ACAGGACCACAACAGCTGGAGAAAGGACTTAGTAACCTACATCGAATTGTAACGGAAGATCTCCAAGCCC**
Seq2 Sscrofa8 chromosome10	.................................G....................................
Seq3 Sscrofa8 chromosome13	....
Sus scrofa(EU086224)	....
Sus scrofa(EU086222)	....
Sus scrofa(EU086220)	....................A........................G........................
Sus scrofa(EU086219)	....G........................
Sus scrofa(AJ288585)	........G..N
Sus scrofa(AJ293656)	........G...
Sus scrofa(EU789636)	........G...
Sus scrofa(AF435967)	........G...
Sus scrofa(AY288779)	........G...
Sus scrofa(EU086221)	....
Sus scrofa(EF133960)	....
Sus scrofa(AY368582)	....
Sus scrofa(AY368580)	....
Sus scrofa(AF417222)	........G...
Sus scrofa(AF417225)	........G...
Sus scrofa(AY368587)	........G...
Sus scrofa(AY368581)	........G...
Sus scrofa(AY312524)	........G...
Sus scrofa(AY371067)	........G...
Sus scrofa(AY312526)	........G...
Sus scrofa(AY368588)	........G...
Sus scrofa(AF417223)	........G...
Sus scrofa(AF417224)	........G...............................G.............................
Sus scrofa(AY312521)	........G...
Sus scrofa(AJ279056)	........G...
Sus scrofa(AY312523)	........G...
Sus scrofa(AY368589)	........G...
Sus scrofa(AF296168)	........G...
Sus scrofa(AF417226)	........G...

```
Sus scrofa(Y12238)                ........G.............................................................
4 Sus scrofa A                    ........................................C......C......................
25 Sus scrofa A                   .................................G..................T..........T......
6 Sus celebensis A                ..G...................................................................
8 Sus celebensis A                ..........G...................G..G.G......A.....A...T.................
1 Potamochoerus larvatus C        .................................G...................A...............T
6 Potamochoerus larvatus C        ...........G.....A..C.........G....A......AA....A..........C......A...
9 Potamochoerus larvatus C        ...........G.....A..C.........G..G.G......A.....A..........C..........
3 Potamochoerus porcus C          ...........G.....A..C.........G..G.G......--------------...C......A...
4 Potamochoerus porcus A          .................................G...................A................
5 Potamochoerus porcus A          ..G...................................................................
19 Potamochoerus porcus A         .................A...............G...................A................
1 Phacochoerus africanus A        ..G........G.....A..C.........G..G.G......A.....A..........C......A...
4 Phacochoerus africanus A        ..G........G.....A..C.........G..G.G......A.....A..........C......A...
4 Phacochoerus africanus C        ..G........G.....A..C.........G..G.G......A.....A..........C......A...
6 Phacochoerus africanus A        ..G........G.....A..C.........G..G.G......A.....A..........C....G.A...
7 Phacochoerus africanus C        ..G........G.....A..C.........G..G.G......A.....A..........C......A...
12 Phacochoerus africanus C       ..G........G.....A..C.........G..G.G......A.....A..........C......A...
8 Phacochoerus aethiopicus C      ..........G....T..A.A.........G..G.G......A.....A.....................

                                          1340      1350      1360      1370      1380      1390      1400
                                  ....|....|....|....|....|....|....|....|....|....|....|....|....|....|
Seq1 Sscrofa8 chromosome8         T-AGAAAAATCTGTCAGTAACCTGGAGGAATCCCTAACCTCCTTATCTGAAGTGGTTCTACAGAACAGAA
Seq2 Sscrofa8 chromosome10        .-.....................A..............................................
Seq3 Sscrofa8 chromosome13        .-....................................................................
Sus scrofa(EU086224)              .-....................................................................
Sus scrofa(EU086222)              .-....................................................................
Sus scrofa(EU086220)              .-....................................................................
Sus scrofa(EU086219)              .-....................................................................
Sus scrofa(AJ288585)              .-....................................................................
Sus scrofa(AJ293656)              .-....................................................................
Sus scrofa(EU789636)              .-.....................A..............................................
Sus scrofa(AF435967)              .-....................................................................
Sus scrofa(AY288779)              .-....................................................................
Sus scrofa(EU086221)              .-....................................................................
Sus scrofa(EF133960)              .-....................................................................
Sus scrofa(AY368582)              .-....................................................................
Sus scrofa(AY368580)              .-....................................................................
Sus scrofa(AF417222)              .-....................................................................
Sus scrofa(AF417225)              .-....................................................................
Sus scrofa(AY368587)              .-..............A.....................................................
Sus scrofa(AY368581)              .-....................................................................
Sus scrofa(AY312524)              .-....................................................................
Sus scrofa(AY371067)              .-....................................................................
Sus scrofa(AY312526)              .-....................................................................
Sus scrofa(AY368588)              .-....................................................................
```

```
Sus scrofa(AF417223)               .-....................................................................
Sus scrofa(AF417224)               .-....................................................................
Sus scrofa(AY312521)               .-....................................................................
Sus scrofa(AJ279056)               .-....................................................................
Sus scrofa(AY312523)               .-....................................................................
Sus scrofa(AY368589)               .-....................................................................
Sus scrofa(AF296168)               .-....................................................................
Sus scrofa(AF417226)               .-....................................................................
Sus scrofa(Y12238)                 .-....................................................................
4 Sus scrofa A                     .-....................................................................
25 Sus scrofa A                    .-.............T......AA................................C.............
6 Sus celebensis A                 .-....................................................................
8 Sus celebensis A                 .-...............C...-.A..............T..T....................A....G.
1 Potamochoerus larvatus C         .-.....................A...................................G..........
6 Potamochoerus larvatus C         .-.A.................A.A....G...........T..G......A........A....G.
9 Potamochoerus larvatus C         .-...................A.......G...........T...........A........A....G.
3 Potamochoerus porcus C           .A.A.................A.A....G...........T..G......A........A..G..G.
4 Potamochoerus porcus A           .-.............G.......A........T..........................G..........
5 Potamochoerus porcus A           .-....................................................................
19 Potamochoerus porcus A          .-...........C.........A.............................A....G..........
1 Phacochoerus africanus A         .-...................A.A....G...........T..G......A........A....G.
4 Phacochoerus africanus A         .-...................A.A....G...........T..G......A........A....G.
4 Phacochoerus africanus C         .-...........C.......A.A....G...........T..G......A........A....G.
6 Phacochoerus africanus A         .-...................A.A....G...........T..G......A........A....G.
7 Phacochoerus africanus C         .-...........C.......A.A....G...........T..G......A........A....G.
12 Phacochoerus africanus C        .-...........C.......A.A....G...........T..G......A........A....G.
8 Phacochoerus aethiopicus C       .-...............C...T.A..............T..T....................A....G.

                                            1410      1420      1430      1440      1450      1460      1470
                                   ....|....|....|....|....|....|....|....|....|....|....|....|....|....|
Seq1 Sscrofa8 chromosome8          GGGGGTTAGATCTGTTATTTCT-AAAAGAAGGAGGGTTATGTGTAGCCTTAAAAGAGGAATGCTGCTTTT
Seq2 Sscrofa8 chromosome10         ......................-.........T.....................................
Seq3 Sscrofa8 chromosome13         ......................-...............................................
Sus scrofa(EU086224)               ......................-...............................................
Sus scrofa(EU086222)               ......................-...............................................
Sus scrofa(EU086220)               ......................-...............................................
Sus scrofa(EU086219)               ......................-...............................................
Sus scrofa(AJ288585)               ......................-...............................................
Sus scrofa(AJ293656)               ......................-...............................................
Sus scrofa(EU789636)               ......................-...............................................
Sus scrofa(AF435967)               ......................-...............................................
Sus scrofa(AY288779)               ......................-...............................................
Sus scrofa(EU086221)               ......................-...............................................
Sus scrofa(EF133960)               ......................-...............................................
Sus scrofa(AY368582)               ......................-...............................................
Sus scrofa(AY368580)               ......................-...............................................
```

```
Sus scrofa(AF417222)           ......................-...............................................
Sus scrofa(AF417225)           ......................-...............................................
Sus scrofa(AY368587)           ......................-............A..................................
Sus scrofa(AY368581)           ......................-...............................................
Sus scrofa(AY312524)           ......................-...............................................
Sus scrofa(AY371067)           ......................-...............................................
Sus scrofa(AY312526)           ......................-...............................................
Sus scrofa(AY368588)           ......................-...............................................
Sus scrofa(AF417223)           ......................-...............................................
Sus scrofa(AF417224)           ......................-...............................................
Sus scrofa(AY312521)           ......................-...............................................
Sus scrofa(AJ279056)           ......................-...............................................
Sus scrofa(AY312523)           ......................-...............................................
Sus scrofa(AY368589)           ......................-...............................................
Sus scrofa(AF296168)           ......................-.................C.............................
Sus scrofa(AF417226)           ......................-...............................................
Sus scrofa(Y12238)             ......................-.............................................C.
4 Sus scrofa A                 ......................-...............................................
25 Sus scrofa A                ......................-.........T.......................A.....T.....C.
6 Sus celebensis A             ......................-...............................................
8 Sus celebensis A             .A..A.................A....A....T.....................................
1 Potamochoerus larvatus C     ......................-.........T...A.................................
6 Potamochoerus larvatus C     AA...G.G..............-...G.....T...C....................A...A........
9 Potamochoerus larvatus C     .A...AC...............-...GA....T...C.................................
3 Potamochoerus porcus C       .A...A................-...G.....T...C..........T......................
4 Potamochoerus porcus A       ....A.................-.........T..A..................................
5 Potamochoerus porcus A       ......................-...............................................
19 Potamochoerus porcus A      ......................-.........T........................A............
1 Phacochoerus africanus A     .A...A................-...G.....T...C............................T....
4 Phacochoerus africanus A     .A...A................-...G.....T...C............................T....
4 Phacochoerus africanus C     .A...A................-...G.....T...C............................T....
6 Phacochoerus africanus A     .A...A................-...G.....T...C............................T....
7 Phacochoerus africanus C     .A...A................-...G.....T...C............................T....
12 Phacochoerus africanus C    .A...A................-...G.....T...C............................T....
8 Phacochoerus aethiopicus C   .A..A.................-.........T.......................A.............

                                       1480      1490      1500      1510      1520      1530      1540
                               ....|....|....|....|....|....|....|....|....|....|....|....|....|....|
Seq1 Sscrofa8 chromosome8      ATGTGGATCATTCAGGAGCTATCAGGGACTCCATGAGCAAGCTCAGAGAAAGGTT----AGAAAAACGTC
Seq2 Sscrofa8 chromosome10     .............G.........................................----...........
Seq3 Sscrofa8 chromosome13     .......................................................----...........
Sus scrofa(EU086224)           .......................................................----...........
Sus scrofa(EU086222)           .......................................................----...........
Sus scrofa(EU086220)           ...............................................A.......----...........
Sus scrofa(EU086219)           .......................................................----...........
Sus scrofa(AJ288585)           .......................................................----...G.......
```

```
Sus scrofa(AJ293656)          .......................................................----...........
Sus scrofa(EU789636)          .......................................................----...........
Sus scrofa(AF435967)          .......................................................----...........
Sus scrofa(AY288779)          .......................................................----...........
Sus scrofa(EU086221)          .......................................................----...........
Sus scrofa(EF133960)          .......................................................----.......T...
Sus scrofa(AY368582)          .......................................................----...........
Sus scrofa(AY368580)          .......................................................----...........
Sus scrofa(AF417222)          ......................................................C----...........
Sus scrofa(AF417225)          .......................................................----...........
Sus scrofa(AY368587)          .......................................................----...........
Sus scrofa(AY368581)          .......................................................----...........
Sus scrofa(AY312524)          .......................................................----...........
Sus scrofa(AY371067)          .......................................................----...........
Sus scrofa(AY312526)          .......................................................----...........
Sus scrofa(AY368588)          .......................................................----...........
Sus scrofa(AF417223)          .......................................................----...........
Sus scrofa(AF417224)          .......................................................----...........
Sus scrofa(AY312521)          .......................................................----...........
Sus scrofa(AJ279056)          .......................................................----...........
Sus scrofa(AY312523)          .......................................................----...........
Sus scrofa(AY368589)          .......................................................----...........
Sus scrofa(AF296168)          .......................................................----...........
Sus scrofa(AF417226)          .......................................................----...........
Sus scrofa(Y12238)            ....A.....C........C.....A.................T...........----...G.GG....
4 Sus scrofa A                .......................................................----...........
25 Sus scrofa A               ..........C........C.....A.................T...........----...G.GG....
6 Sus celebensis A            .......................................................----...........
8 Sus celebensis A            .............G.................T.A..A.........GA.......----...........
1 Potamochoerus larvatus C    ......................T.......................G........----.........C.
6 Potamochoerus larvatus C    ....A.................T...A..........T..A..T...........----.A.........
9 Potamochoerus larvatus C    ......................T..............T.....T...........AAAA.A.....A...
3 Potamochoerus porcus C      ............TG........T.......T......T.....T.A........C----.-------...
4 Potamochoerus porcus A      .........................A..........A.........G........----.A.....-...
5 Potamochoerus porcus A      .......................................................----...........
19 Potamochoerus porcus A     ......................T.......................G........----...........
1 Phacochoerus africanus A    ......................T..............T..A..T...........----...........
4 Phacochoerus africanus A    ......................T..............T..A..T...........----...........
4 Phacochoerus africanus C    ......................T..............T..A..T...........----...........
6 Phacochoerus africanus A    ......................T..............T..A..T...........----...........
7 Phacochoerus africanus C    ......................T..............T..A..T...........----...........
12 Phacochoerus africanus C   ......................T..............T..A..T...........----...........
8 Phacochoerus aethiopicus C  .............G......................A......T...A.......----...........

                                       1550      1560      1570      1580      1590      1600
                              ....|....|....|....|....|....|....|....|....|....|....|....|..
```

```
Seq1 Sscrofa8 chromosome8       ACAAAGAAAAAGAGGCTGGCCAAGGATGGTTTGAGGGATGGTTCAACAAGTCCCCATGGGTG
Seq2 Sscrofa8 chromosome10      ...G..........................................................
Seq3 Sscrofa8 chromosome13      ..............................................................
Sus scrofa(EU086224)            ..............................................................
Sus scrofa(EU086222)            ..............................................................
Sus scrofa(EU086220)            ..............................................................
Sus scrofa(EU086219)            ..............................................................
Sus scrofa(AJ288585)            ..............................................................
Sus scrofa(AJ293656)            ..............................................................
Sus scrofa(EU789636)            ..............................................................
Sus scrofa(AF435967)            ..............................................................
Sus scrofa(AY288779)            ..............................................................
Sus scrofa(EU086221)            ..............................................................
Sus scrofa(EF133960)            ..............................................................
Sus scrofa(AY368582)            ..............................................................
Sus scrofa(AY368580)            ..............................................................
Sus scrofa(AF417222)            ..............................................................
Sus scrofa(AF417225)            .A..GA...G....................................................
Sus scrofa(AY368587)            ..............................................................
Sus scrofa(AY368581)            ..............................................................
Sus scrofa(AY312524)            ..............................................................
Sus scrofa(AY371067)            ..............................................................
Sus scrofa(AY312526)            ..............................................................
Sus scrofa(AY368588)            ..............................................................
Sus scrofa(AF417223)            ..............................................................
Sus scrofa(AF417224)            ............................................G.................
Sus scrofa(AY312521)            ..............................................................
Sus scrofa(AJ279056)            ..............................................................
Sus scrofa(AY312523)            ..............................................................
Sus scrofa(AY368589)            ..............................................................
Sus scrofa(AF296168)            ..............................................................
Sus scrofa(AF417226)            ..............................................................
Sus scrofa(Y12238)              GA.GG....G........A...G..G........A.............G...T..T...A..
4 Sus scrofa A                  ..............................................................
25 Sus scrofa A                 GA.GG....G........A...G..G........A.............G...T..T...A..
6 Sus celebensis A              ..............................................................
8 Sus celebensis A              .........G....................................................
1 Potamochoerus larvatus C      .T............................................................
6 Potamochoerus larvatus C      .....A...G.......A.........................................A..
9 Potamochoerus larvatus C      .........G.......AA........................T.............A.A..
3 Potamochoerus porcus C        .........G.......A............................................
4 Potamochoerus porcus A        .T...–........................................................
5 Potamochoerus porcus A        ..............................................................
19 Potamochoerus porcus A       .T...A........................................................
1 Phacochoerus africanus A      .........G.......A.........................................A..
4 Phacochoerus africanus A      .........G.......A.........................................A..
```

4 *Phacochoerus africanus* C ………G.......A...A..

6 *Phacochoerus africanus* A .........G.......A...A..

7 *Phacochoerus africanus* C .........G.......A...A..

12 *Phacochoerus africanus* C .........G.......A...A..

8 *Phacochoerus aethiopicus* C ...........A..A.........T..............A......................
