# Supplementary material for: Evolution of endogenous retroviruses in the Suidae: evidence for different viral subpopulations in African and Eurasian host species
Source: BMC Evol Biol. 2011 May 24;11:139. doi: 10.1186/1471-2148-11-139 (PMC3128044; doi:10.1186/1471-2148-11-139)
Supplement: Additional file 5 — env B alignment. env B alignment of sequences generated in this study, sequences from GenBank and the draft pig genome [file 1471-2148-11-139-S5.PDF]

env B alignment (including all sequences)

The first number next to some sequence names represents the clone number.

```
                                        10        20        30        40        50        60        70
                                ....|....|....|....|....|....|....|....|....|....|....|....|....|....|
Seq1 Sscrofa8 chromosome4       GG-ATTGA-TTCCTACCAACACGCCTAGAAACTCCCCAGGTGTTCCTGTTAAGACAGGACAGAGACTCTT
Seq2 Sscrofa8 chromosome8       ..-.....-.............................................................
Seq3 Sscrofa8 chromosome16      ..-.....-.............................................................
Sus scrofa(AY056025)            ..-.....-............T................................................
Sus scrofa(AJ293657)            ..-.....-.............................................................
Sus scrofa(AY056026)            ..-.....-.............................................................
Sus scrofa(AY312528)            ..-.....-.............................................................
Sus scrofa(AY312530)            C.-.....-.............................................................
Sus scrofa(AY312522)            ..-.....-.............................................................
Sus scrofa(AJ279057)            ..-.....-.............................................................
Sus scrofa(AY056027)            ..-.....-.............................................................
Sus scrofa(AY056035)            ..-.....-.............................................................
Sus scrofa(AY056028)            ..-.....-.............................................................
Sus scrofa(AY056024)            ..-.....-.............................................................
Sus scrofa(AY312529)            ..-.....-.............................................................
Sus scrofa(AY312532)            ..-.....-.............................................................
Sus scrofa(AY312518)            ..-.....-.............................................................
Sus scrofa(AJ133816)            ..-.....-.............................................................
Sus scrofa(AY312517)            ..-.....-.............................................................
Sus scrofa(AJ133818)            ..-.....-.............................................................
Sus scrofa(Y17013)              ..-.....-.............................................................
Sus scrofa(EU523109)            ..-.....-.............................................................
Sus scrofa(AY099324)            ..-.....-.............................................................
Sus scrofa(Y12239)              ..-.....-.............................................................
1 Sus scrofa                    ..-.....T............................C................................
3 Sus scrofa                    ..-.....T.......T...---------------------------------..............A..
5 Sus scrofa                    ..-.....T............................C................................
8 Sus scrofa                    ..-.....-.............................................................
2 Sus barbatus barbatus         ..-.....-...........---------------------------------..............A..
7 Sus barbatus barbatus         ..-.....-.............................A...............................
9 Sus barbatus barbatus         ..-.....T.......................C....C................................
12 Sus barbatus barbatus        ..-.....-.....................................C.......................
3 Sus barbatus oi               ---------------------T................................................
5 Sus barbatus oi               ..T.....-.............................A...............................
8 Sus barbatus oi               ..-.....-........-....................................................
4 Sus verrucosus                ..-.....-........-...T.....C.........C................................
5 Sus verrucosus                ..-.....T............................C................................
6 Sus verrucosus                ..-.....T............................C................................
8 Sus verrucosus                ..-.....T............................C................................
9 Sus verrucosus                ..-.....-........-....................................................
```

```
4 Sus celebensis                ..-.....-.............................A...............................
5 Sus celebensis                ..-.....T............................C................................
6 Sus celebensis                ..-.....T............................C................................
10 Sus celebensis               ..-.....-................C............................................
4 Potamochoerus larvatus        ..-.....-............T...............CC...............................
5 Potamochoerus larvatus        -----...-.......TG...................CA...........G..........A........
10 Potamochoerus larvatus       -------------...TG...................CA......................A........
4 Potamochoerus porcus          ..-...-.-............T.....A.........CC...C...........................
5 Potamochoerus porcus          ..-.....-............T...............CC...............................
8 Potamochoerus porcus          ..-...-.-............T...............CC...............................
1 Hylochoerus meinertzhageni    ..-.....-............................C................................
11 Hylochoerus meinertzhageni   ..-.....-............................C.................G..............
13 Hylochoerus meinertzhageni   ..-.....-............................C................................
4 Phacochoerus africanus        ..-.....-.C.....T....T.T...A.........C.......................A........
7 Phacochoerus aethiopicus      ..-.....-.C.....T....T.TT..A.........C.......................A........
12 Phacochoerus aethiopicus     ..-.....-.C..........T.T...A.........C.......................A........
15 Phacochoerus aethiopicus     ..-.....-.C..........T.T...A.........C.......................A........

                                         80        90       100       110       120       130       140
                                ....|....|....|....|....|....|....|....|....|....|....|....|....|....|
Seq1 Sscrofa8 chromosome4       CAGTCTCATCCAGGGAGCTTTCCAAGCCATCAACTCCACCGACCCTGATGCCACTTCTTCTTGTTGGCTT
Seq2 Sscrofa8 chromosome8       ......................................................................
Seq3 Sscrofa8 chromosome16      ......................................................................
Sus scrofa(AY056025)            ......................................................................
Sus scrofa(AJ293657)            ......................................................................
Sus scrofa(AY056026)            ......................................................................
Sus scrofa(AY312528)            ......................................................................
Sus scrofa(AY312530)            ......................................................................
Sus scrofa(AY312522)            ......................................................................
Sus scrofa(AJ279057)            ......................................................................
Sus scrofa(AY056027)            ......................................................................
Sus scrofa(AY056035)            ......................................................................
Sus scrofa(AY056028)            ......................................................................
Sus scrofa(AY056024)            ......................................................................
Sus scrofa(AY312529)            ....................C.................................................
Sus scrofa(AY312532)            ......................................................................
Sus scrofa(AY312518)            ......................................................................
Sus scrofa(AJ133816)            ......................................................................
Sus scrofa(AY312517)            ......................................................................
Sus scrofa(AJ133818)            ......................................................................
Sus scrofa(Y17013)              ......................................................................
Sus scrofa(EU523109)            ......................................................................
Sus scrofa(AY099324)            ......................................................................
Sus scrofa(Y12239)              ......................................................................
1 Sus scrofa                    .............................................C........................
3 Sus scrofa                    ..........................T.C................C..............C.........
```

```
5 Sus scrofa                        .............................................C........................
8 Sus scrofa                        .............................................C........................
2 Sus barbatus barbatus             ..........................T.C...........C....C..............C.........
7 Sus barbatus barbatus             .............................................C........................
9 Sus barbatus barbatus             .............................................C.......---..............
12 Sus barbatus barbatus            .............................................C........................
3 Sus barbatus oi                   .............................A...............C........................
5 Sus barbatus oi                   .............................................C........................
8 Sus barbatus oi                   .............................................C........................
4 Sus verrucosus                    T................T......................A..T.C.............T..........
5 Sus verrucosus                    .............................................C.......---..............
6 Sus verrucosus                    .............................................C.......---..............
8 Sus verrucosus                    .............................................C.......---..............
9 Sus verrucosus                    .............................................C........................
4 Sus celebensis                    .............................................C........................
5 Sus celebensis                    .............................................C........................
6 Sus celebensis                    .............................................C.......---..............
10 Sus celebensis                   .............................................C........................
4 Potamochoerus larvatus            .................................T......A....C........C...............
5 Potamochoerus larvatus            T.........T...........T.............T........C........................
10 Potamochoerus larvatus           T.........T...........T.............T........C........................
4 Potamochoerus porcus              .................................T...G..A....C....................A...
5 Potamochoerus porcus              .................................T......A....C........................
8 Potamochoerus porcus              .................................T......A....C........................
1 Hylochoerus meinertzhageni        .............................................C........................
11 Hylochoerus meinertzhageni       .............................................C........................
13 Hylochoerus meinertzhageni       .............................................C........................
4 Phacochoerus africanus            ............A.....C..T..G...C.A..T.....AAC...GA...TT..C..A...........C
7 Phacochoerus aethiopicus          .C..........A.....C..T..G...C.A..T.....AAC...G....TT..C..A...........C
12 Phacochoerus aethiopicus         ............A.....C..T..G...C.A..T.....AAC...GA...TT..C..A...........C
15 Phacochoerus aethiopicus         ............A.....C..T..G...C.A..T.....AAC...GA...TT..C..A...........C

                                            150       160       170       180       190       200       210
                                    ....|....|....|....|....|....|....|....|....|....|....|....|....|....|
Seq1 Sscrofa8 chromosome4           TGTCTATCCTCAGGGCCTCCTTATTATGAGGGGATGGCTAAAGAAGGAAAATTCAATGTGACCAAAGAGC
Seq2 Sscrofa8 chromosome8           ......................................................................
Seq3 Sscrofa8 chromosome16          ......................................................................
Sus scrofa(AY056025)                ......................................................................
Sus scrofa(AJ293657)                ......................................................................
Sus scrofa(AY056026)                ......................................................................
Sus scrofa(AY312528)                ......................................................................
Sus scrofa(AY312530)                ......................................................................
Sus scrofa(AY312522)                ......................................................................
Sus scrofa(AJ279057)                ......................................................................
Sus scrofa(AY056027)                ......................................................................
Sus scrofa(AY056035)                ......................................................................
```

Sus scrofa(AY056028)	....
Sus scrofa(AY056024)	....
Sus scrofa(AY312529)	....
Sus scrofa(AY312532)	....
Sus scrofa(AY312518)	....
Sus scrofa(AJ133816)	....
Sus scrofa(AY312517)	....
Sus scrofa(AJ133818)	....
Sus scrofa(Y17013)	....
Sus scrofa(EU523109)	....
Sus scrofa(AY099324)	....
Sus scrofa(Y12239)	....
1 *Sus scrofa*	................................**A**.......**G**......**G**......................
3 *Sus scrofa*	................................**A**.......**G**......**G**....................**A**.
5 *Sus scrofa*	................................**A**.......**G**......**G**......................
8 *Sus scrofa*	......................................**C**...............................
2 *Sus barbatus barbatus*	................................**A**.......**G**......**G**....................**A**.
7 *Sus barbatus barbatus*	....
9 *Sus barbatus barbatus*	................................**A**.......**G**......**G**......................
12 *Sus barbatus barbatus*	....
3 *Sus barbatus oi*	....
5 *Sus barbatus oi*	....
8 *Sus barbatus oi*	....
4 *Sus verrucosus*	................................**A**.......**G**......**G**......................
5 *Sus verrucosus*	................................**A**.......**G**......**G**......................
6 *Sus verrucosus*	................................**A**.......**G**......**G**......................
8 *Sus verrucosus*	................................**A**.......**G**......**G**......................
9 *Sus verrucosus*	....
4 *Sus celebensis*	....
5 *Sus celebensis*	................................**A**.......**G**......**G**......................
6 *Sus celebensis*	................................**A**.......**G**......**G**......................
10 *Sus celebensis*	....
4 *Potamochoerus larvatus*	.........**A**...................**A**.............**C**...**G**.....................**T**
5 *Potamochoerus larvatus*	.................**C**...........**A**..**A**..**A**....**GG**.....**G**............**C**.......**A**.
10 *Potamochoerus larvatus*	.................**C**...........**A**..**A**..**A**....**GG**.....**G**....................**A**.
4 *Potamochoerus porcus*	.........**A**.....................**A**...........**C**...**G**......................
5 *Potamochoerus porcus*	.........**A**...................**A**..........**T**..**C**...**G**......................
8 *Potamochoerus porcus*	.........**A**....**A**............................**C**...**G**......................
1 *Hylochoerus meinertzhageni*	................................**A**.......**G**......**G**.......**G**..............
11 *Hylochoerus meinertzhageni*	................................**A**.......**G**......**G**......................
13 *Hylochoerus meinertzhageni*	................................**A**.......**G**......**G**.......**G**..............
4 *Phacochoerus africanus*	..**CT**..**G**.**T**..**G**..**C**..**A**.....**C**........**A**.......**G**..**G**...**G**.........**A**....**A**..**G**..**A**.
7 *Phacochoerus aethiopicus*	..**CT**..**G**.**T**..**G**..**C**..**A**.....**C**........**A**.......**G**..**G**...**G**..............**A**..**G**..**A**.
12 *Phacochoerus aethiopicus*	..**CT**..**G**.**T**..**G**..**C**..**A**.....**C**........**A**.......**G**..**G**...**G**..............**A**..**G**..**A**.
15 *Phacochoerus aethiopicus*	..**CT**..**G**.**T**..**G**..**C**..**A**.....**C**........**A**.......**G**..**G**...**G**..............**A**..**G**..**A**.

```
                                           220       230       240       250       260       270       280
                                  ....|....|....|....|....|....|....|....|....|....|....|....|....|....|
Seq1 Sscrofa8 chromosome4         ATAGAAATCAATGTACATGGGGGTCCCGAAATAAGCTTACCCTCACTGAAGTTTCCGGGAAGGGGACATG
Seq2 Sscrofa8 chromosome8         ......................................................................
Seq3 Sscrofa8 chromosome16        ......................................................................
Sus scrofa(AY056025)              ......................................................................
Sus scrofa(AJ293657)              ......................................................................
Sus scrofa(AY056026)              G.....................................................................
Sus scrofa(AY312528)              G.....................................................................
Sus scrofa(AY312530)              G.....................................................................
Sus scrofa(AY312522)              G.....................................................................
Sus scrofa(AJ279057)              G.....................................................................
Sus scrofa(AY056027)              ......................................................................
Sus scrofa(AY056035)              ......................................................................
Sus scrofa(AY056028)              G.....................................................................
Sus scrofa(AY056024)              ......................................................................
Sus scrofa(AY312529)              ......................................................................
Sus scrofa(AY312532)              ......................................................................
Sus scrofa(AY312518)              ......................................................................
Sus scrofa(AJ133816)              ......................................................................
Sus scrofa(AY312517)              ......................................................................
Sus scrofa(AJ133818)              ......................................................................
Sus scrofa(Y17013)                ......................................................................
Sus scrofa(EU523109)              ......................................................................
Sus scrofa(AY099324)              ......................................................................
Sus scrofa(Y12239)                ......................................................................
1 Sus scrofa                      .....................................C................................
3 Sus scrofa                      .................C....................................................
5 Sus scrofa                      .....................................C................................
8 Sus scrofa                      ...........................................................G..........
2 Sus barbatus barbatus           ......................................................................
7 Sus barbatus barbatus           ................................................T...............A.....
9 Sus barbatus barbatus           .....................................C.................T..............
12 Sus barbatus barbatus          ......................................................................
3 Sus barbatus oi                 ......................................................................
5 Sus barbatus oi                 ................................................................A.....
8 Sus barbatus oi                 ......................................................................
4 Sus verrucosus                  ...C..............................A......T............................
5 Sus verrucosus                  .....................................C.................T..............
6 Sus verrucosus                  .....................................C.................T..............
8 Sus verrucosus                  .....................................C.................T..............
9 Sus verrucosus                  ......................................................................
4 Sus celebensis                  ......................................................................
5 Sus celebensis                  .....................................C.................T..............
6 Sus celebensis                  ............................G........C.................T..............
10 Sus celebensis                 ......................................................................
```

```
4 Potamochoerus larvatus          .......................................................T..............
5 Potamochoerus larvatus          ...............................C...........A.....G.....T..A..A.....C..
10 Potamochoerus larvatus         .......C.......................C...........A.....G.....T..A..A.....C..
4 Potamochoerus porcus            ..................................A....................T..A...........
5 Potamochoerus porcus            .......................................................T..............
8 Potamochoerus porcus            .......................................................T..............
1 Hylochoerus meinertzhageni      ..............................G.......................................
11 Hylochoerus meinertzhageni     ......................................................................
13 Hylochoerus meinertzhageni     ..............................G.......................................
4 Phacochoerus africanus          .....G.C..............A....A...............T.....G.....T.-----------..
7 Phacochoerus aethiopicus        .....G.C..............A....A...............T.....G.....T..A..A..C..C..
12 Phacochoerus aethiopicus       .....G.C..............A....A...............T.....G.....T..A..A..C..C..
15 Phacochoerus aethiopicus       .....G.C..............A....A...............T.....G.....T..A..A..C..C..

                                          290       300       310       320       330       340       350
                                  ....|....|....|....|....|....|....|....|....|....|....|....|....|....|
Seq1 Sscrofa8 chromosome4         CATAGGAAAAGCTCCCCCATCCCACCAACACCTTTGCTA-------------------------------
Seq2 Sscrofa8 chromosome8         .......................................-------------------------------
Seq3 Sscrofa8 chromosome16        .......................................-------------------------------
Sus scrofa(AY056025)              .......................................-------------------------------
Sus scrofa(AJ293657)              .....................................A.-------------------------------
Sus scrofa(AY056026)              .......................................-------------------------------
Sus scrofa(AY312528)              ......................................G-------------------------------
Sus scrofa(AY312530)              ......................................G-------------------------------
Sus scrofa(AY312522)              ......................................G-------------------------------
Sus scrofa(AJ279057)              ......................................G-------------------------------
Sus scrofa(AY056027)              .......................................-------------------------------
Sus scrofa(AY056035)              .......................................-------------------------------
Sus scrofa(AY056028)              .......................................-------------------------------
Sus scrofa(AY056024)              .......................................-------------------------------
Sus scrofa(AY312529)              .......................................-------------------------------
Sus scrofa(AY312532)              .......................................-------------------------------
Sus scrofa(AY312518)              .......................................-------------------------------
Sus scrofa(AJ133816)              .......................................-------------------------------
Sus scrofa(AY312517)              .......................................-------------------------------
Sus scrofa(AJ133818)              .......................................-------------------------------
Sus scrofa(Y17013)                .......................................-------------------------------
Sus scrofa(EU523109)              .......................................-------------------------------
Sus scrofa(AY099324)              .......................................-------------------------------
Sus scrofa(Y12239)                .......................................-------------------------------
1 Sus scrofa                      .....................................A.-------------------------------
3 Sus scrofa                      .....................................A.-------------------------------
5 Sus scrofa                      .....................................A.-------------------------------
8 Sus scrofa                      .......................................-------------------------------
2 Sus barbatus barbatus           .....................................A.-------------------------------
7 Sus barbatus barbatus           .......................................-------------------------------
```

```
9 Sus barbatus barbatus             .....................................A.-------------------------------
12 Sus barbatus barbatus            .......................................-------------------------------
3 Sus barbatus oi                   .......................................-------------------------------
5 Sus barbatus oi                   .......................................-------------------------------
8 Sus barbatus oi                   .......................................-------------------------------
4 Sus verrucosus                    .....................................A.-------------------------------
5 Sus verrucosus                    .....................................A.-------------------------------
6 Sus verrucosus                    .....................................A.-------------------------------
8 Sus verrucosus                    .....................................A.-------------------------------
9 Sus verrucosus                    .......................................-------------------------------
4 Sus celebensis                    .......................................-------------------------------
5 Sus celebensis                    .......................................-------------------------------
6 Sus celebensis                    ..................................C..A.-------------------------------
10 Sus celebensis                   .......................................-------------------------------
4 Potamochoerus larvatus            .......................................-------------------------------
5 Potamochoerus larvatus            T..........T..........T.......T..C...A.-------------------------------
10 Potamochoerus larvatus           T..........T..........T.......T..C...A.-------------------------------
4 Potamochoerus porcus              .....A.................................-------------------------------
5 Potamochoerus porcus              .......................................-------------------------------
8 Potamochoerus porcus              .......................................-------------------------------
1 Hylochoerus meinertzhageni        ..........A..........................A.TAGCACTATGGTTTATGAGCAGGAAAAACTC
11 Hylochoerus meinertzhageni       ..........A..........................A.TAGCACTATGGTTTATGAGCAGGAAAAACTC
13 Hylochoerus meinertzhageni       ..........A..........................A.TAGCACTATGGTTTATGAGCAGGAAAAACTC
4 Phacochoerus africanus            ......G.GG.T........................TA.-------------------------------
7 Phacochoerus aethiopicus          ......G.GG.T........................TA.-------------------------------
12 Phacochoerus aethiopicus         ......G.GG.T........................TA.-------------------------------
15 Phacochoerus aethiopicus         ......G.GG.T........................TA.-------------------------------

                                            360       370       380       390       400       410       420
                                    ....|....|....|....|....|....|....|....|....|....|....|....|....|....|
Seq1 Sscrofa8 chromosome4           -----------------TAGTACTGTGGTTTATGAGCAGGCCTCAGAAAATCAGTATTTAGTACCTGGTT
Seq2 Sscrofa8 chromosome8           -----------------.....................................................
Seq3 Sscrofa8 chromosome16          -----------------.....................................................
Sus scrofa(AY056025)                -----------------..................A..................................
Sus scrofa(AJ293657)                -----------------.....................................................
Sus scrofa(AY056026)                -----------------.....................................................
Sus scrofa(AY312528)                -----------------.....................................................
Sus scrofa(AY312530)                -----------------.....................................................
Sus scrofa(AY312522)                -----------------.....................................................
Sus scrofa(AJ279057)                -----------------.....................................................
Sus scrofa(AY056027)                -----------------.....................................................
Sus scrofa(AY056035)                -----------------.....................................................
Sus scrofa(AY056028)                -----------------.....................................................
Sus scrofa(AY056024)                -----------------.....................................................
Sus scrofa(AY312529)                -----------------.....................................................
Sus scrofa(AY312532)                -----------------.....................................................
```

```
Sus scrofa(AY312518)            -----------------.....................................................
Sus scrofa(AJ133816)            -----------------.....................................................
Sus scrofa(AY312517)            -----------------.....................................................
Sus scrofa(AJ133818)            -----------------.....................................................
Sus scrofa(Y17013)              -----------------.....................................................
Sus scrofa(EU523109)            -----------------.....................................................
Sus scrofa(AY099324)            -----------------.....................................................
Sus scrofa(Y12239)              -----------------.....................................................
1 Sus scrofa                    -----------------.......A..................T..........................
3 Sus scrofa                    -----------------.......A........A....................................
5 Sus scrofa                    -----------------.......A..................T..........................
8 Sus scrofa                    -----------------.....................................................
2 Sus barbatus barbatus         -----------------.......A........A....................................
7 Sus barbatus barbatus         -----------------.....................................................
9 Sus barbatus barbatus         -----------------.......A..................T..........................
12 Sus barbatus barbatus        -----------------.....................................................
3 Sus barbatus oi               -----------------.......................................C.............
5 Sus barbatus oi               -----------------.....................................................
8 Sus barbatus oi               -----------------.....................................................
4 Sus verrucosus                -----------------.......A.............................................
5 Sus verrucosus                -----------------.......A..................T..G.......................
6 Sus verrucosus                -----------------.......A..................T..G.......................
8 Sus verrucosus                -----------------.......A..................T..G.......................
9 Sus verrucosus                -----------------.....................................................
4 Sus celebensis                -----------------.....................................................
5 Sus celebensis                -----------------.....................................................
6 Sus celebensis                -----------------.......A..................T..........................
10 Sus celebensis               -----------------.....................................................
4 Potamochoerus larvatus        -----------------.......A.........................C...................
5 Potamochoerus larvatus        -----------------.GA...CACACG....A.CAGCT...........T.A..C..G..G.....C.
10 Potamochoerus larvatus       -----------------.GA...CACACG....A.CAGCT.............A..C..G..G.....C.
4 Potamochoerus porcus          -----------------.......A.........................C...............A...
5 Potamochoerus porcus          -----------------.......A.........................C..............A....
8 Potamochoerus porcus          -----------------.......A.........................C...................
1 Hylochoerus meinertzhageni    CCCCAACGCCTTTGCAG...C...A.......................T.....................
11 Hylochoerus meinertzhageni   CCCCAACACCTTTGCAG...C...A.............................................
13 Hylochoerus meinertzhageni   CCCCAACGCCTTTGCAG...C...A.......................T.....................
4 Phacochoerus africanus        -----------------CCAC..G.AA.CC.T.A.T.GAA....T..G.G.......C.G..........
7 Phacochoerus aethiopicus      -----------------CCAC.TG.AA.CC.T.A.T.GAA....T..G.G.......C.G..........
12 Phacochoerus aethiopicus     -----------------CCAC.TA.AA.CC.T.A.T.GAA....T..G.G.......C.G..........
15 Phacochoerus aethiopicus     -----------------CCAC.TA.AA.CC.T.A.T.GAA....T..G.G.......C.G..........

                                        430       440       450       460       470       480       490
                                ....|....|....|....|....|....|....|....|....|....|....|....|....|....|
Seq1 Sscrofa8 chromosome4       GTAACAGGTGGTGGGCATGCAATACTGGGTTAACCCCCTGTGTTTCCACCTCAGTCTTCAACCAATCCAA
Seq2 Sscrofa8 chromosome8       A.....................................................................
```

Seq3 Sscrofa8 chromosome16	**A**...
Sus scrofa(AY056025)	**A**...**T**...........
Sus scrofa(AJ293657)	**A**...
Sus scrofa(AY056026)	**A**...
Sus scrofa(AY312528)	**A**..........................**C**..
Sus scrofa(AY312530)	**A**...
Sus scrofa(AY312522)	**A**...
Sus scrofa(AJ279057)	**A**...
Sus scrofa(AY056027)	**A**...
Sus scrofa(AY056035)	**A**...
Sus scrofa(AY056028)	**A**...
Sus scrofa(AY056024)	**A**...
Sus scrofa(AY312529)	**A**..**T**......................
Sus scrofa(AY312532)	**A**...
Sus scrofa(AY312518)	**A**...
Sus scrofa(AJ133816)	**A**...
Sus scrofa(AY312517)	**A**...............................**C**.....................................
Sus scrofa(AJ133818)	**A**..**T**......................
Sus scrofa(Y17013)	**A**..**T**......................
Sus scrofa(EU523109)	**A**...
Sus scrofa(AY099324)	**A**...
Sus scrofa(Y12239)	**A**...
1 *Sus scrofa*	**A**....**T**.**A**..........................**T**...........**T**...**A**.......**T**...........
3 *Sus scrofa*	**A**.................................**T**...**C**.......**T**...**A**.......**T**...........
5 *Sus scrofa*	**A**....**T**.**A**..........................**T**...........**T**...**A**.......**T**...........
8 *Sus scrofa*	**A**...
2 *Sus barbatus barbatus*	**A**.................................**T**...........**T**...**A**.......**T**...........
7 *Sus barbatus barbatus*	**A**.................**A**...
9 *Sus barbatus barbatus*	**A**....**T**............................**T**...........**T**...**A**.......**T**...........
12 *Sus barbatus barbatus*	**A**...
3 *Sus barbatus oi*	**A**...
5 *Sus barbatus oi*	**A**...
8 *Sus barbatus oi*	**A**...
4 *Sus verrucosus*	**A**.................................**T**...........**T**...**A**......--...........
5 *Sus verrucosus*	**A**....**T**............................**T**...........**T**...**A**.......**T**...........
6 *Sus verrucosus*	**A**....**T**............................**T**...........**T**...**A**.......**T**...........
8 *Sus verrucosus*	**A**....**T**............................**T**...........**T**...**A**.......**T**...........
9 *Sus verrucosus*	**A**...
4 *Sus celebensis*	**A**..**T**..........
5 *Sus celebensis*	**A**...
6 *Sus celebensis*	**A**....**T**............................**T**...........**T**...**A**.......**T**...........
10 *Sus celebensis*	**A**...
4 *Potamochoerus larvatus*	**A**...**T**.......................
5 *Potamochoerus larvatus*	**A**...**T**.......................
10 *Potamochoerus larvatus*	**A**....**C**..........**T**..**T**...........**G**...**T**.**T**.....**C**..**TG**..**A**....**T**..**T**........**T**..
4 *Potamochoerus porcus*	**A**.........**A**...................................**T**.......................

```
5 Potamochoerus porcus            A.............................................T.......................
8 Potamochoerus porcus            A.............................................T.......................
1 Hylochoerus meinertzhageni      A.................................T...........T...A.......T...........
11 Hylochoerus meinertzhageni     A.................................T...........T...A.......T...........
13 Hylochoerus meinertzhageni     A.................................T...........T...A.......T...........
4 Phacochoerus africanus          A.G................T........A........T............CTG..T.....T...A.T..
7 Phacochoerus aethiopicus        A.G................T........A........T............CTG..T.....T...A.T..
12 Phacochoerus aethiopicus       A.G................T........A........T............CTG..T.....T...A.T..
15 Phacochoerus aethiopicus       A.G................T........A........T............CTG..T.....T...A.T..

                                           500       510       520       530       540       550       560
                                  ....|....|....|....|....|....|....|....|....|....|....|....|....|....|
Seq1 Sscrofa8 chromosome4         AGATTTCTGTGTCATGGTCCAAATCGTCCCCCGAGTGTACTACCATCCTGAGGAAGTGGTCCTTGATGAA
Seq2 Sscrofa8 chromosome8         ......................................................................
Seq3 Sscrofa8 chromosome16        ......................................................................
Sus scrofa(AY056025)              ......................................................................
Sus scrofa(AJ293657)              ......................................................................
Sus scrofa(AY056026)              .............................................C........................
Sus scrofa(AY312528)              .............................................C........................
Sus scrofa(AY312530)              .............................................C........................
Sus scrofa(AY312522)              .............................................C........................
Sus scrofa(AJ279057)              .............................................C........................
Sus scrofa(AY056027)              ......................................................................
Sus scrofa(AY056035)              ......................................................................
Sus scrofa(AY056028)              ......................................................................
Sus scrofa(AY056024)              ......................................................................
Sus scrofa(AY312529)              ......................................................................
Sus scrofa(AY312532)              ......................................................................
Sus scrofa(AY312518)              ......................................................................
Sus scrofa(AJ133816)              ......................................................................
Sus scrofa(AY312517)              ......................................................................
Sus scrofa(AJ133818)              ......................................................................
Sus scrofa(Y17013)                ......................................................................
Sus scrofa(EU523109)              .....................................................G................
Sus scrofa(AY099324)              .........................A............................................
Sus scrofa(Y12239)                ......................................................................
1 Sus scrofa                      .....................GC........T.G..AC...................A...A.C.....G
3 Sus scrofa                      .........C...........GC..........G..A....................A...A.C.....G
5 Sus scrofa                      .....................GC........T.G..AC...................A...A.C.....G
8 Sus scrofa                      .........................A............................................
2 Sus barbatus barbatus           .....................GC..........G..A....................A...A.......G
7 Sus barbatus barbatus           ......................................................................
9 Sus barbatus barbatus           .....................GC........T.G..AC...................A...A.C.....G
12 Sus barbatus barbatus          ......................................................................
3 Sus barbatus oi                 ...............................-......................................
5 Sus barbatus oi                 ......................................................................
```

```
8 Sus barbatus oi                 ......................................................................
4 Sus verrucosus                  .....................GC..........G..A....................ACA.A.C.....G
5 Sus verrucosus                  .....................GC........T.G..AC...................A...A.C.....G
6 Sus verrucosus                  .....................GC........T.G..AC...................A...A.C.....G
8 Sus verrucosus                  .....................GC........T.G..AC...................A...A.C.....G
9 Sus verrucosus                  ......................................................................
4 Sus celebensis                  ......................................................................
5 Sus celebensis                  .................................................................T....
6 Sus celebensis                  .....................GC........T.G..AC...................A...A.C.....G
10 Sus celebensis                 ......................................................................
4 Potamochoerus larvatus          ......................................................................
5 Potamochoerus larvatus          ..........................C...........................................
10 Potamochoerus larvatus         ............T.........T....T.....G..A..T.................A..GG.G..C.C.
4 Potamochoerus porcus            ...................................................................A..
5 Potamochoerus porcus            .....................GC..........G..A....................A...A.C.....G
8 Potamochoerus porcus            ......................................................................
1 Hylochoerus meinertzhageni      .....................GC..........GC.A....................A...A.C.....G
11 Hylochoerus meinertzhageni     .....................GC..........GC.A....................A...A.C.....G
13 Hylochoerus meinertzhageni     .....................GC..........GC.A....................A...A.C.....G
4 Phacochoerus africanus          ...C..T.....T...........T...........A..T................CAA..A..AG...G
7 Phacochoerus aethiopicus        ...C..T.....T...........T...........A..T................CAA..A..AG...G
12 Phacochoerus aethiopicus       ...C..T.....T...........T...........A..T................CAA..A..AG...G
15 Phacochoerus aethiopicus       ...C..T.....T...........T...........A..T................CAA..A..AG...G

                                          570       580       590       600       610       620       630
                                  ....|....|....|....|....|....|....|....|....|....|....|....|....|....|
Seq1 Sscrofa8 chromosome4          TATGACTATCGGTATAACCGACCAAAAAGAGAACCCGTATCCCTT-ACCCTAGCTGTAATGCTCGGATTA
Seq2 Sscrofa8 chromosome8          .............................................-........................
Seq3 Sscrofa8 chromosome16         .............................................-........................
Sus scrofa(AY056025)              .......................G.....................-........................
Sus scrofa(AJ293657)              .............................................-........................
Sus scrofa(AY056026)              .......................G.....................-........................
Sus scrofa(AY312528)              .......................G.....................-........................
Sus scrofa(AY312530)              .......................G.....................-........................
Sus scrofa(AY312522)              .......................G.....................-........................
Sus scrofa(AJ279057)              .......................G.....................-........................
Sus scrofa(AY056027)              .............................................-........................
Sus scrofa(AY056035)              .............................................-........................
Sus scrofa(AY056028)              .......................G.....................-........................
Sus scrofa(AY056024)              .............................................-........................
Sus scrofa(AY312529)              .............................................-........................
Sus scrofa(AY312532)              .............................................-........................
Sus scrofa(AY312518)              .............................................-........................
Sus scrofa(AJ133816)              .............................................-........................
Sus scrofa(AY312517)              .............................................-........................
Sus scrofa(AJ133818)              .............................................-........................
```

```
Sus scrofa(Y17013)              .............................................-........................
Sus scrofa(EU523109)            .............................................-........................
Sus scrofa(AY099324)            .............................................-........................
Sus scrofa(Y12239)              .............................................-........................
1 Sus scrofa                    ............CC..C...CT..........G.....GA.....A........C..T..A..A......
3 Sus scrofa                    ......C....ACCA.C...TT..........G.....G......-...........C.....T......
5 Sus scrofa                    ............CC..C...CT..........G.....GA.....-........C..T..A..A......
8 Sus scrofa                    .......................G.....................-........................
2 Sus barbatus barbatus         ......C....ACCA.C.T.TT..........G.....G......-...........C.....T......
7 Sus barbatus barbatus         ......................TG.....................-........................
9 Sus barbatus barbatus         ............CC..C...CT..........G.....GA.....-........C..T..A..A......
12 Sus barbatus barbatus        .......................G.....................T........................
3 Sus barbatus oi               ......................TG....A................-........................
5 Sus barbatus oi               ......................TG.....................-........................
8 Sus barbatus oi               .......................G.....................-........................
4 Sus verrucosus                ............CC..C...CT..........G.....GA.....-........C..T..A..T......
5 Sus verrucosus                ............CC..C...CT..........G.....GA.....-........C..T..A..A......
6 Sus verrucosus                ............CC..C...CT..........G.....GA.....-........C..T..A..A......
8 Sus verrucosus                ............CC..C...CT..........G.....GA.....-........C..T..A..T......
9 Sus verrucosus                ......................TG.....................-........................
4 Sus celebensis                ......................TG.....................-........................
5 Sus celebensis                ......................TG.....................-........................
6 Sus celebensis                ............CC..C...CT..........G.....GA.....-C.......C..T..A..A......
10 Sus celebensis               .......................G.G...................-........................
4 Potamochoerus larvatus        .........T.............G............A........-..................A.....
5 Potamochoerus larvatus        .........T.............G............A........-..................A.....
10 Potamochoerus larvatus       ........C..ACC..C...GAG......G.....TA.G......-..T..G..C..T.....T......
4 Potamochoerus porcus          .........T..........................A....T...-........................
5 Potamochoerus porcus          ............CC..C...CT..........G...T.GA.....-........C..T.....T......
8 Potamochoerus porcus          .........T..........................A....T...-........................
1 Hylochoerus meinertzhageni    ............CC..C...C...........G.....GA....C-........C..T..A..T.A....
11 Hylochoerus meinertzhageni   ............CC..C...C...........G.....GA.....-........C..T..A..T......
13 Hylochoerus meinertzhageni   ............CC..C...C...........G.....GA....C-........C..T..A..T.A....
4 Phacochoerus africanus        .....T.......C..CT..CT..............TCGG.....-........C..T.....T......
7 Phacochoerus aethiopicus      .....T.......C..CT..CT..............TCGG.....-........C..T.....T......
12 Phacochoerus aethiopicus     .....T...T...C..CT..CT..............TCGG.....-........C..T.....T......
15 Phacochoerus aethiopicus     .....T...T...C..CT..CT..............TCGG.....-........C..T.....T......

                                         640       650       660       670       680       690       700
                                ....|....|....|....|....|....|....|....|....|....|....|....|....|....|
Seq1 Sscrofa8 chromosome4       GGGACGGCCGTTGGCGTAGGAACAGGGACAGCTGCCCTGATCACAGGACCACAGCAGCTAGAGAAAGGAC
Seq2 Sscrofa8 chromosome8       ......................................................................
Seq3 Sscrofa8 chromosome16      ..............................A.......................................
Sus scrofa(AY056025)            ......................................................................
Sus scrofa(AJ293657)            ......................................................................
Sus scrofa(AY056026)            ...............A......................................................
```

Sus scrofa(AY312528)	...............A..
Sus scrofa(AY312530)	...............A...C......
Sus scrofa(AY312522)	...............A..
Sus scrofa(AJ279057)	...............A..
Sus scrofa(AY056027)	..............................A.......................................
Sus scrofa(AY056035)	..............................A.......................................
Sus scrofa(AY056028)	....
Sus scrofa(AY056024)	....
Sus scrofa(AY312529)	....
Sus scrofa(AY312532)	....
Sus scrofa(AY312518)	....
Sus scrofa(AJ133816)	....
Sus scrofa(AY312517)	....
Sus scrofa(AJ133818)	....
Sus scrofa(Y17013)	....
Sus scrofa(EU523109)	....
Sus scrofa(AY099324)	....
Sus scrofa(Y12239)	....
1 *Sus scrofa*	....T...---...T..G..G...A.......
3 *Sus scrofa*	..AGT.A...CC..T..G..G..C...G.............................T....A.......
5 *Sus scrofa*	....T...---...T..G..G...A.......
8 *Sus scrofa*	....................G...
2 *Sus barbatus barbatus*	..AGT.A...CC..T.C-------...G.............................T....A.......
7 *Sus barbatus barbatus*	....
9 *Sus barbatus barbatus*	....T...---...T..G..G...A.......
12 *Sus barbatus barbatus*	T.................T.......T...
3 *Sus barbatus oi*	............T.T...
5 *Sus barbatus oi*	....
8 *Sus barbatus oi*	....
4 *Sus verrucosus*	....T.....---.T..G..A.......
5 *Sus verrucosus*	....T...---...T..G..G...A..G....
6 *Sus verrucosus*	....T...---...T..G..G...A..G....
8 *Sus verrucosus*	....T...---...T..G..G...A..G....
9 *Sus verrucosus*	....
4 *Sus celebensis*	....
5 *Sus celebensis*	.....................G..
6 *Sus celebensis*	....T...---...T..G..G...............................G.........A.......
10 *Sus celebensis*	....
4 *Potamochoerus larvatus*	....A.......
5 *Potamochoerus larvatus*	....A.......
10 *Potamochoerus larvatus*	..AGTAA...CC..A.....T.....A..G..A...T..........G.........T....-.....G.
4 *Potamochoerus porcus*	.....A....C...
5 *Potamochoerus porcus*	....T.....---.T..G..G...A.......
8 *Potamochoerus porcus*	.....A....C...
1 *Hylochoerus meinertzhageni*	...GT.---..C..T..G..G...A.......
11 *Hylochoerus meinertzhageni*	...GT.---..C..T..G..G...A.......

```
13 Hylochoerus meinertzhageni    ...GT.---..C..T..G..G.........................................A.......
4 Phacochoerus africanus         ---------.CC..TA....G..G..A...........A..T............................
7 Phacochoerus aethiopicus       ---------.CC..TA....G..G..AG..A.......A..T..................A.........
12 Phacochoerus aethiopicus      ---------.CC..TA....G..G..A...A.......A..T............................
15 Phacochoerus aethiopicus      ---------.CC..TA....G..G..A...A.......A..T............................

                                         710       720       730       740       750       760       770
                                 ....|....|....|....|....|....|....|....|....|....|....|....|....|....|
Seq1 Sscrofa8 chromosome4        TTGGTGAGCTACATGCGGCCATGACAGAAGATCTCCGAGCCTTAGAG--------GAGTCTGTTAGCAAC
Seq2 Sscrofa8 chromosome8        ...............................................--------...............
Seq3 Sscrofa8 chromosome16       ...............................................--------...............
Sus scrofa(AY056025)             ...............................................--------...............
Sus scrofa(AJ293657)             ...............................................--------...............
Sus scrofa(AY056026)             ...............................................--------...............
Sus scrofa(AY312528)             ...............................................--------...............
Sus scrofa(AY312530)             ...............................................--------...............
Sus scrofa(AY312522)             ...............................................--------...............
Sus scrofa(AJ279057)             ...............................................--------...............
Sus scrofa(AY056027)             ...............................................--------...............
Sus scrofa(AY056035)             ...............................................--------...............
Sus scrofa(AY056028)             ...............................................--------...............
Sus scrofa(AY056024)             ...............................................--------...............
Sus scrofa(AY312529)             C..............................................--------...............
Sus scrofa(AY312532)             ...............................................--------...............
Sus scrofa(AY312518)             ...............................................--------...............
Sus scrofa(AJ133816)             ...............................................--------...............
Sus scrofa(AY312517)             ...............................................--------...............
Sus scrofa(AJ133818)             ...............................................--------...............
Sus scrofa(Y17013)               ...............................................--------...............
Sus scrofa(EU523109)             ...............................................--------...............
Sus scrofa(AY099324)             ...............................................--------...............
Sus scrofa(Y12239)               ...............................................--------...............
1 Sus scrofa                     ......................A........................--------..A.......A....
3 Sus scrofa                     ....C.C..C..................G.....T..G..A..G...--------..A............
5 Sus scrofa                     ......................A........................--------..A.......A....
8 Sus scrofa                     .................A.............................--------...............
2 Sus barbatus barbatus          ....C.C..C..................G.....T..G..A..G...--------..A.......A....
7 Sus barbatus barbatus          ...............................................--------...............
9 Sus barbatus barbatus          ...............T...................T...........--------..A.......A....
12 Sus barbatus barbatus         ...................A...........................--------..A............
3 Sus barbatus oi                ..............................G................--------...............
5 Sus barbatus oi                ...................-...........................--------...............
8 Sus barbatus oi                ...................A.....G.....................--------..A............
4 Sus verrucosus                 ................A..............................--------..A............
5 Sus verrucosus                 ...............T...................T...........--------..A.......A....
6 Sus verrucosus                 ...............T...................T...........--------..A.......A....
```

```
8 Sus verrucosus                ...............T...................T...........---------.A.......A....
9 Sus verrucosus                ...............................................---------..............
4 Sus celebensis                ...............................................---------..............
5 Sus celebensis                ...............................................---------..............
6 Sus celebensis                ...............T...................T...........---------.A.......A....
10 Sus celebensis               ...................A...........................---------.A............
4 Potamochoerus larvatus        ...............T...............................---------..............
5 Potamochoerus larvatus        ...............T............................T..---------..............
10 Potamochoerus larvatus       ....A.GC..G..G..A..........GG..............G...---------.A............
4 Potamochoerus porcus          ...............T..............................A---------.A............
5 Potamochoerus porcus          ..........C...........................A........---------.A............
8 Potamochoerus porcus          .....A.........T...............................---------.A............
1 Hylochoerus meinertzhageni    ......................................A........---------.A............
11 Hylochoerus meinertzhageni   ......................................A........---------.A............
13 Hylochoerus meinertzhageni   ......................................A........---------.A............
4 Phacochoerus africanus        .......C......CGAATTG.A.............A....C.....ATCTAGAAA.A.....C..T...
7 Phacochoerus aethiopicus      .......C......CGAATTG.A.............A....C....A--------A.A.....C..T...
12 Phacochoerus aethiopicus     .......C......CGAATTG.A.............A....C....A--------A.A.....C..T...
15 Phacochoerus aethiopicus     .......C......CGAATTG.A.............A....C....A--------A.A.....C..T...

                                        780       790       800       810       820       830       840
                                ....|....|....|....|....|....|....|....|....|....|....|....|....|....|
Seq1 Sscrofa8 chromosome4       CTAGAAGAGTCCCTGACTTCTTTGTCTGAAGTGGTTCTACAGAACCGGAGGGGATTAGATCTGCTGTTTC
Seq2 Sscrofa8 chromosome8       ......................................................................
Seq3 Sscrofa8 chromosome16      ......................................................................
Sus scrofa(AY056025)            ......................................................................
Sus scrofa(AJ293657)            ......................................................................
Sus scrofa(AY056026)            ......................................................................
Sus scrofa(AY312528)            ......................................................................
Sus scrofa(AY312530)            ......................................................................
Sus scrofa(AY312522)            ......................................................................
Sus scrofa(AJ279057)            ......................................................................
Sus scrofa(AY056027)            ......................................................................
Sus scrofa(AY056035)            ......................................................................
Sus scrofa(AY056028)            ......................................................................
Sus scrofa(AY056024)            .............................G........................................
Sus scrofa(AY312529)            ......................................................................
Sus scrofa(AY312532)            ......................................................................
Sus scrofa(AY312518)            ......................................................................
Sus scrofa(AJ133816)            ......................................................................
Sus scrofa(AY312517)            ......................................................................
Sus scrofa(AJ133818)            ......................................................................
Sus scrofa(Y17013)              ......................................................................
Sus scrofa(EU523109)            ......................................................................
Sus scrofa(AY099324)            ......................................................................
Sus scrofa(Y12239)              ......................................................................
```

```
1 Sus scrofa                           ........A....................................T........................
3 Sus scrofa                           ........A........CC..........G..A..C................................C.
5 Sus scrofa                           ........A....................................T........................
8 Sus scrofa                           ......................................................................
2 Sus barbatus barbatus                ........A.............................................................
7 Sus barbatus barbatus                ......................................................................
9 Sus barbatus barbatus                ........A.............................................................
12 Sus barbatus barbatus               ......................................................................
3 Sus barbatus oi                      ......................................................................
5 Sus barbatus oi                      ......................................................................
8 Sus barbatus oi                      .............................................T........................
4 Sus verrucosus                       ........A........................A...........T.................--.....
5 Sus verrucosus                       ........A.............................................................
6 Sus verrucosus                       ........A.............................................................
8 Sus verrucosus                       ........A.............................................................
9 Sus verrucosus                       ......................................................................
4 Sus celebensis                       ......................................................................
5 Sus celebensis                       ......................................................................
6 Sus celebensis                       ........A.............................................................
10 Sus celebensis                      .............................................T........................
4 Potamochoerus larvatus               ..........T...T.....C.................................................
5 Potamochoerus larvatus               ..........T...T.....C.................................................
10 Potamochoerus larvatus              .....G..A...T.A..C..C...........A...T....A..T.AT..A...C.......CT.A....
4 Potamochoerus porcus                 ..............T.......................................................
5 Potamochoerus porcus                 T.......A.............................................................
8 Potamochoerus porcus                 .............CT.......................................................
1 Hylochoerus meinertzhageni           ........A.............................................................
11 Hylochoerus meinertzhageni          ........A.............................................................
13 Hylochoerus meinertzhageni          ........A.............................................................
4 Phacochoerus africanus               .....G........A..C..C........................A.A.....G......A..T.A....
7 Phacochoerus aethiopicus             .....G........A.AC..C..............C.........A.A.....G......T..T.A....
12 Phacochoerus aethiopicus            .....G........A..C..C..............C.........A.A.....N......T..T.A....
15 Phacochoerus aethiopicus            .....G........A..C..C..............C.........A.A.....G......T..T.A....

                                                850       860       870       880       890       900       910
                                       ....|....|....|....|....|....|....|....|....|....|....|....|....|....|
Seq1 Sscrofa8 chromosome4              TAAGAG-AAGGTGGGTTATGTGCAGCCTTAAAAGAAGAATGTTGCTTCTATGTAGATCACTCAGGAGCCA
Seq2 Sscrofa8 chromosome8              ......-...............................................................
Seq3 Sscrofa8 chromosome16             ......-...............................................................
Sus scrofa(AY056025)                   ......-...............................................................
Sus scrofa(AJ293657)                   ......-...............................................................
Sus scrofa(AY056026)                   ......-...............................................................
Sus scrofa(AY312528)                   ......-...............................................................
Sus scrofa(AY312530)                   ......-...............................................................
Sus scrofa(AY312522)                   ......-...............................................................
Sus scrofa(AJ279057)                   ......-...............................................................
```

Sus scrofa(AY056027)	......-...
Sus scrofa(AY056035)	......-...
Sus scrofa(AY056028)	......-...
Sus scrofa(AY056024)	......-...
Sus scrofa(AY312529)	......-...
Sus scrofa(AY312532)	......-...
Sus scrofa(AY312518)	......-...
Sus scrofa(AJ133816)	......-...
Sus scrofa(AY312517)	......-...
Sus scrofa(AJ133818)	......-...
Sus scrofa(Y17013)	......-...
Sus scrofa(EU523109)	......-...
Sus scrofa(AY099324)	......-...
Sus scrofa(Y12239)	......-...
1 *Sus scrofa*	...**A**..-........................**G**.....................**G**................
3 *Sus scrofa*	...**A**..-.........................**G**.......................**C**.....**G**.......
5 *Sus scrofa*	...**A**..-........................**G**.....................**G**................
8 *Sus scrofa*	......-...
2 *Sus barbatus barbatus*	...**A**..-.............**C**..........**G**.....................**G**................
7 *Sus barbatus barbatus*	......-..**C**......
9 *Sus barbatus barbatus*	...**A**..-........................**G**.....................**G**................
12 *Sus barbatus barbatus*	......-...
3 *Sus barbatus oi*	......-.............**G**..**C**......
5 *Sus barbatus oi*	......-..**C**......
8 *Sus barbatus oi*	......-..**T**......
4 *Sus verrucosus*	...**A**..-...**CG**.......**TG**.......
5 *Sus verrucosus*	...**A**..-........................**G**.....................**G**................
6 *Sus verrucosus*	...**A**..-........................**G**.....................**G**................
8 *Sus verrucosus*	...**A**..-........................**G**.....................**G**.........**C**......
9 *Sus verrucosus*	......-..**C**......
4 *Sus celebensis*	......-...
5 *Sus celebensis*	......-....................**C**..
6 *Sus celebensis*	...**A**..-........................**G**.....................**G**................
10 *Sus celebensis*	......-...**A**...
4 *Potamochoerus larvatus*	......-..**G**................
5 *Potamochoerus larvatus*	......-..**G**................
10 *Potamochoerus larvatus*	...**A**..-....**A**.**A**.........**G**.....**G**..**G**...........**T**...........**C**......**T**.**G**....
4 *Potamochoerus porcus*	......-.............................**A**................**G**................
5 *Potamochoerus porcus*	...**A**..-.......................---....................**G**........**G**.......
8 *Potamochoerus porcus*	......-..**G**................
1 *Hylochoerus meinertzhageni*	...**A**..-..**A**...**G**........**G**.......
11 *Hylochoerus meinertzhageni*	...**A**..-..**G**........**G**.......
13 *Hylochoerus meinertzhageni*	...**A**..-..**A**...**G**........**G**.......
4 *Phacochoerus africanus*	...**A**..-.......**A**.......**T**..................**G**................**G**....**C**....**T**.
7 *Phacochoerus aethiopicus*	...**A**..-.......**A**.......**T**...**T**.
12 *Phacochoerus aethiopicus*	...**A**..**A**.......**A**.......**T**...**T**.

```
15 Phacochoerus aethiopicus   ...A..-.......A.......T.............................................T.

                                       920       930       940       950       960       970       980
                              ....|....|....|....|....|....|....|....|....|....|....|....|....|....|
Seq1 Sscrofa8 chromosome4     TCAGAGACTCCATGAGCAAGCTTAGAG-AAAGGTTAGAGAGGCGTCGAAGGGAAAGAGAGGCTGACCAGG
Seq2 Sscrofa8 chromosome8     ...........................-..........................................
Seq3 Sscrofa8 chromosome16    ...........................-..........................................
Sus scrofa(AY056025)          ...........................-..........................................
Sus scrofa(AJ293657)          ...........................-..........................................
Sus scrofa(AY056026)          ...........................-..........................................
Sus scrofa(AY312528)          ...........................-..........................................
Sus scrofa(AY312530)          ...........................-..........................................
Sus scrofa(AY312522)          ...........................-..........................................
Sus scrofa(AJ279057)          ...........................-..........................................
Sus scrofa(AY056027)          ...........................-..........................................
Sus scrofa(AY056035)          ...........................-..........................................
Sus scrofa(AY056028)          ...........................-..........................................
Sus scrofa(AY056024)          ...........................-..........................................
Sus scrofa(AY312529)          ...........................-..........................................
Sus scrofa(AY312532)          ...........................-..........................................
Sus scrofa(AY312518)          ...........................-..........................................
Sus scrofa(AJ133816)          ...........................-..........................................
Sus scrofa(AY312517)          ...........................-..........................................
Sus scrofa(AJ133818)          ...........................-..........................................
Sus scrofa(Y17013)            ...........................-..........................................
Sus scrofa(EU523109)          ...........................-..........................................
Sus scrofa(AY099324)          ...........................-..........................................
Sus scrofa(Y12239)            ...........................-..........................................
1 Sus scrofa                  ...........................-.............A............................
3 Sus scrofa                  ....G..T........T..........-.............A............................
5 Sus scrofa                  ...........................-.............A............................
8 Sus scrofa                  ................G..........-..........................................
2 Sus barbatus barbatus       .................G.........-.............A............................
7 Sus barbatus barbatus       ...........................-..........................................
9 Sus barbatus barbatus       ...........................-.............A.......A....................
12 Sus barbatus barbatus      ...........................-..........................................
3 Sus barbatus oi             ...........................-..........................................
5 Sus barbatus oi             ...........................-.......................C..........C.......
8 Sus barbatus oi             ...........................-.....................A....................
4 Sus verrucosus              ........................T..-.............A..................T.........
5 Sus verrucosus              ...........................-.....A.......A.......T....................
6 Sus verrucosus              ...........................A.....A.......A............................
8 Sus verrucosus              ...........................-.....A.......A............................
9 Sus verrucosus              ...........................-.......................................-..
4 Sus celebensis              ...........................-.................................G........
5 Sus celebensis              ...........................-..........................................
```

```
6 Sus celebensis                 ...........................-.............A............................
10 Sus celebensis                ...........................-..........................................
4 Potamochoerus larvatus         ...........................-...................................A......
5 Potamochoerus larvatus         ...........................-...................................A......
10 Potamochoerus larvatus        ..C........................-.............A........A...................
4 Potamochoerus porcus           ...........................-.............A........A...................
5 Potamochoerus porcus           ...........................-.............A........----................
8 Potamochoerus porcus           ...........................-.............A........A...................
1 Hylochoerus meinertzhageni     C................T.........-.............A........A...........G.......
11 Hylochoerus meinertzhageni    C................T.........-......A......A........A...........G..-....
13 Hylochoerus meinertzhageni    C................T.........-.............A........A...........G.......
4 Phacochoerus africanus         ....G.....T................-............AAT..T.T.AA........A...A....A.
7 Phacochoerus aethiopicus       ....G.....T................-............AA.....T.A.........AA..A....A.
12 Phacochoerus aethiopicus      ....G.....T....C...........-............AA....AT.A.........AA..A....A.
15 Phacochoerus aethiopicus      ....G.....T....C...........-............AA....AT.A.........AA..A....A.

                                          990       1000      1010      1020      1030      1040
                                 ....|....|....|....|....|....|....|....|....|....|....|....|.
Seq1 Sscrofa8 chromosome4        GGTGGTTTGAAGGATGGTTCAACAG--------------GTCTCCTTGGATGACCACCCTG
Seq2 Sscrofa8 chromosome8        .........................--------------......................
Seq3 Sscrofa8 chromosome16       .........................--------------......................
Sus scrofa(AY056025)             .........................--------------......................
Sus scrofa(AJ293657)             .........................--------------......................
Sus scrofa(AY056026)             .........................--------------......................
Sus scrofa(AY312528)             .........................--------------......................
Sus scrofa(AY312530)             .........................--------------......................
Sus scrofa(AY312522)             .........................--------------......................
Sus scrofa(AJ279057)             .........................--------------......................
Sus scrofa(AY056027)             .........................--------------......................
Sus scrofa(AY056035)             .........................--------------......................
Sus scrofa(AY056028)             .........................--------------......................
Sus scrofa(AY056024)             .........................--------------......................
Sus scrofa(AY312529)             ........A................--------------......................
Sus scrofa(AY312532)             .........................--------------......................
Sus scrofa(AY312518)             .........................--------------......................
Sus scrofa(AJ133816)             .........................--------------......................
Sus scrofa(AY312517)             .........................--------------......................
Sus scrofa(AJ133818)             ........A................--------------......................
Sus scrofa(Y17013)               .........................--------------......................
Sus scrofa(EU523109)             .........................--------------......................
Sus scrofa(AY099324)             .........................--------------......................
Sus scrofa(Y12239)               .........................--------------......................
1 Sus scrofa                     .........................--------------.............---------
3 Sus scrofa                     .........................GATGGTTGAAGGAT.GT.............G.....
5 Sus scrofa                     .........................--------------......................
8 Sus scrofa                     .........................--------------......................
```

2 *Sus barbatus barbatus*	.........................--------------......................
7 *Sus barbatus barbatus*	.................**C**.......--------------......................
9 *Sus barbatus barbatus*	.........................--------------......................
12 *Sus barbatus barbatus*	.........................--------------......................
3 *Sus barbatus oi*	.........................--------------......................
5 *Sus barbatus oi*	.........................--------------......................
8 *Sus barbatus oi*	......................-..--------------......................
4 *Sus verrucosus*	.........................--------------......................
5 *Sus verrucosus*	.........................--------------......................
6 *Sus verrucosus*	.........................--------------......................
8 *Sus verrucosus*	.........................--------------......................
9 *Sus verrucosus*	.........................--------------......................
4 *Sus celebensis*	.........................--------------......................
5 *Sus celebensis*	.........................--------------......................
6 *Sus celebensis*	.........................--------------......................
10 *Sus celebensis*	.........................--------------......................
4 *Potamochoerus larvatus*	.........................--------------......................
5 *Potamochoerus larvatus*	....................**G**....--------------......................
10 *Potamochoerus larvatus*	.........................--------------.............**G**........
4 *Potamochoerus porcus*	.........................--------------......................
5 *Potamochoerus porcus*	.........................--------------......................
8 *Potamochoerus porcus*	.................**C**.......--------------......................
1 *Hylochoerus meinertzhageni*	.........................--------------......................
11 *Hylochoerus meinertzhageni*	.........................--------------......................
13 *Hylochoerus meinertzhageni*	.........................--------------......................
4 *Phacochoerus africanus*	.**A**........**G**.............**A**--------------...**C**..**A**...**G**.........**C**.
7 *Phacochoerus aethiopicus*	.**A**........**G**.............**A**--------------...**C**..**A**...**GC**..........
12 *Phacochoerus aethiopicus*	.**A**........**G**.............**A**--------------...**C**..**A**...**GC**..........
15 *Phacochoerus aethiopicus*	.**A**........**G**.............**A**--------------...**C**..**A**...**GC**..........
