# Supplementary material for: Evolution of endogenous retroviruses in the Suidae: evidence for different viral subpopulations in African and Eurasian host species
Source: BMC Evol Biol. 2011 May 24;11:139. doi: 10.1186/1471-2148-11-139 (PMC3128044; doi:10.1186/1471-2148-11-139)
Supplement: Additional file 6 — env C alignment. env C alignment of sequences generated in this study, sequences from GenBank and the draft pig genome [file 1471-2148-11-139-S6.PDF]

env C alignment (including all sequences)

The first number next to some sequence names represents the clone number. A = fragment amplified using primers for *env* A; C = fragment amplified using primers for *env* C.

```
                                         10        20        30        40        50        60        70
                                ....|....|....|....|....|....|....|....|....|....|....|....|....|....|
Sus scrofa(AM229313)            GAACCTGGTGGCCTGATCTATACGTTTGCCTCAGATCAGTTATTCCTAGTCT---------GACCTCACC
Sus scrofa(AM229312)            ....................................................---------.........
Sus scrofa(EU090250)            ....................................................---------.........
Sus scrofa(AF417229)            --..............AT....T..C.....TC.......A..C...G....CAATGACCA.G..A....
Sus scrofa(AF402660)            ....................................................---------.........
Sus scrofa(AF402661)            ....................................................---------.........
Sus scrofa(AF402662)            ....................................................---------.........
Sus scrofa(AF402663)            -.....................T..C..............C...........---------.......A.
Sus scrofa(DQ996276)            -.....................T..C..............C...........---------.......A.
1 Sus scrofa C                  ......................T.............................---------......G..
8 Sus scrofa C                  ......................T.............................---------......G..
13 Sus scrofa C                 ......................T.............................---------......G..
1 Sus barbatus barbatus A       ......................T.............................---------......G..
2 Sus barbatus barbatus A       ......................T.............................---------......G..
11 Sus barbatus barbatus A      ......................T.............................---------......G..
12 Sus barbatus barbatus C      ......................T..C..............C...........---------.......A.
14 Sus barbatus barbatus C      ......................T..C..............C...........---------.......A.
17 Sus barbatus barbatus C      ......................T..C..............C...........---------.C..CACA.
2 Sus barbatus oi A             ......................T.............................---------......G..
2 Sus barbatus oi C             ......................T..C..............C...........---------.......A.
3 Sus barbatus oi C             ......................T..C..............C...........---------.......A.
5 Sus barbatus oi C             ......................T..C..............C...........---------.C..CACA.
1 Sus verrucosus C              ............T.........T..C.........A....C...........---------.C..CACA.
3 Sus verrucosus A              ......................T..C..............C...........---------.......A.
3 Sus verrucosus C              ......................T..C..............C...........---------.C..CACA.
5 Sus verrucosus A              ......................T.............................---------.........
5 Sus verrucosus C              ......................T..C..............C...........---------.......A.
7 Sus verrucosus A              ......................T..C..............C...........---------.......A.
2 Sus celebensis C              ......................T..C..............C...........---------.......A.
14 Sus celebensis C             ......................T..C..............C...........---------.......A.
18 Sus celebensis C             ......................T..C..............C...........---------.......A.

                                         80        90       100       110       120       130       140
                                ....|....|....|....|....|....|....|....|....|....|....|....|....|....|
Sus scrofa(AM229313)            CCCAGATATCCTCCATGCTCACGGATTTTATGTTTGCCCAGGACCACCAAATAATGGAAAACATTGCGGA
Sus scrofa(AM229312)            ......................................................................
Sus scrofa(EU090250)            ......................................................................
Sus scrofa(AF417229)            ...C...G.A....G....T....G.....C.........................A.G...........
Sus scrofa(AF402660)            ......................................................................
```

Sus scrofa(AF402661)	....
Sus scrofa(AF402662)	....
Sus scrofa(AF402663)	..............G....T...C.AT...
Sus scrofa(DQ996276)	..............G....T...C.AT...
1 *Sus scrofa* C	..............G....T..
8 *Sus scrofa* C	..............G....T..
13 *Sus scrofa* C	..............G....T..
1 *Sus barbatus barbatus* A	..............G....T..
2 *Sus barbatus barbatus* A	..............G....T..
11 *Sus barbatus barbatus* A	..............G....T..
12 *Sus barbatus barbatus* C	..............G....T...C..T...
14 *Sus barbatus barbatus* C	..............G....T...C..T...
17 *Sus barbatus barbatus* C	..............GCA..T..........C.........................A......C..T...
2 *Sus barbatus oi* A	....A.........G....T..
2 *Sus barbatus oi* C	..............G....T...C..T...
3 *Sus barbatus oi* C	..............G...CT...C..T...
5 *Sus barbatus oi* C	..............GCA..T..........C.........................A......C..T...
1 *Sus verrucosus* C	.....G........G....T..A..C..T...
3 *Sus verrucosus* A	..............G....TC..C..T...
3 *Sus verrucosus* C	.....G........G....T..A..C..T...
5 *Sus verrucosus* A	..............G....T.........................G........................
5 *Sus verrucosus* C	..............G....T..GC..T...
7 *Sus verrucosus* A	..............G....T...C..T...
2 *Sus celebensis* C	..............G....T..............................G............C..T...
14 *Sus celebensis* C	..............G....T...C..T...
18 *Sus celebensis* C	..............G....T...C..T...

	150 160 170 180 190 200 210
	....|....|....|....|....|....|....|....|....|....|....|....|....|....|
Sus scrofa(AM229313)	**AATCCCAGAGATTTCTTTTGTAAACAATGGAACTGTGTAACCTCTAATGATGGATATTGGAAATGGCCAA**
Sus scrofa(AM229312)	....
Sus scrofa(EU090250)	....
Sus scrofa(AF417229)	....
Sus scrofa(AF402660)	....
Sus scrofa(AF402661)	....
Sus scrofa(AF402662)	....
Sus scrofa(AF402663)	.....T.............AC..........G.....................GA..C.A..........
Sus scrofa(DQ996276)	.....T.............AC..........G......................A..C.A..........
1 *Sus scrofa* C	......C........................G......................................
8 *Sus scrofa* C	......C........................G......................................
13 *Sus scrofa* C	......C........................G......................................
1 *Sus barbatus barbatus* A	......C........................G......................................
2 *Sus barbatus barbatus* A	......C........................G......................................
11 *Sus barbatus barbatus* A	......C........................G......................................
12 *Sus barbatus barbatus* C	.....T..............C..........G.....................GA..C............
14 *Sus barbatus barbatus* C	.....T..............C..........G.....................GA..C............

```
17 Sus barbatus barbatus C   ...T.T..............C..........G.....................GA..C............
2 Sus barbatus oi A          ......C........................G......................................
2 Sus barbatus oi C          .....T..............C..G.......G.....................GA..C............
3 Sus barbatus oi C          .....T..............C..........G.....................GA..C............
5 Sus barbatus oi C          ...T.T..............C..........G.....................GA..C............
1 Sus verrucosus C            .....T..............C..........G.....................GAGCC............
3 Sus verrucosus A            .....T..............C..........G.....................GA..C............
3 Sus verrucosus C            .....T..............C..........G.....................GAGCC............
5 Sus verrucosus A            ...............................G..............................G.......
5 Sus verrucosus C            .....T..............C..........G.....................GA..C............
7 Sus verrucosus A            .....T..............C..........G.....................GA..C............
2 Sus celebensis C            .....T..............C..........G.....................GA..C............
14 Sus celebensis C           .....T..............C..........G..A..................GA..C............
18 Sus celebensis C           .....T..............C..........G....................AGA..C............

                                    220       230       240       250       260       270       280
                             ....|....|....|....|....|....|....|....|....|....|....|....|....|....|
Sus scrofa(AM229313)         CCTCTCAGCAGGATAGGGTAAGTTTTTCTTATGTCAACACCTATA----CCAGCTCTGGACAATTTAATT
Sus scrofa(AM229312)         .............................................----.....................
Sus scrofa(EU090250)         .............................................----.....................
Sus scrofa(AF417229)         .............................................----.....................
Sus scrofa(AF402660)         .............................................----.....................
Sus scrofa(AF402661)         .............................................----.....................
Sus scrofa(AF402662)         .............................................----.....................
Sus scrofa(AF402663)         ......T...............C...............C..A...ACCC..G.AC.....A..CG.C..-
Sus scrofa(DQ996276)         ......T...............C...............C..A...ACCA..G.AC.....A..CG.C..-
1 Sus scrofa C               .............................................----....T....A...........
8 Sus scrofa C               .............................................----....T....A...........
13 Sus scrofa C              .............................................----....T....A...........
1 Sus barbatus barbatus A    .............................................----....T....A...........
2 Sus barbatus barbatus A    .............................................----....T....A...........
11 Sus barbatus barbatus A   .............................................----....T....A...........
12 Sus barbatus barbatus C   ......T...............C...............C..A...ACCG..G.AC....GA..CG.C..-
14 Sus barbatus barbatus C   ......T...............C...............C..A...ACCG..G.AC....GA..CG.C..-
17 Sus barbatus barbatus C   ......T...............C...............C..A...ACCG..G.AC....GA...G.C..-
2 Sus barbatus oi A          .............................................----....T.T..A...........
2 Sus barbatus oi C          ......T...............C...............C..A...ACCG..G.AC....GA..CG.C..-
3 Sus barbatus oi C          ......T...............C...............C..A...ACCG..G.AC....GA..CG.C..-
5 Sus barbatus oi C          ......T...............C...............C..A.C.ACCG..G.AC....GA...G.C.C-
1 Sus verrucosus C            ......T...............C...............C..A...ACCG....AC.CAT.T..CG.C..-
3 Sus verrucosus A            ......T...............C...............C..AG..ACCG..G.AC....GA..CG.C..-
3 Sus verrucosus C            ......T...............C...............C..A...ACCG....AC.CAT.T..CG.C..-
5 Sus verrucosus A            .............................................----....T................
5 Sus verrucosus C            ......T...............C...............C..A...ACCG..G.AC....GA..CG.C..-
7 Sus verrucosus A            ......T...............C...............C..A...ACCG..G.AC....GA..CG.C..-
2 Sus celebensis C            ......T...............C...............C..A...ACCG..G.AC....GA..CG.C..-
```

```
14 Sus celebensis C              ......T...............C...............C..A...ACCG..G.AC....GA..CG.C..-
18 Sus celebensis C              ......T...............C...............C..A...ACCG..G.AC....GA..CA.C..-

                                         290       300       310       320       330       340       350
                                 ....|....|....|....|....|....|....|....|....|....|....|....|....|....|
Sus scrofa(AM229313)             ACCTGACCTGGATTAGAACTGGAAG---CCCCAAGTGCTCTCCTTCAGACCTAGATTACCTAAAAATAAG
Sus scrofa(AM229312)             .........................---..........................................
Sus scrofa(EU090250)             .........................---...............C..........................
Sus scrofa(AF417229)             .........................---..........................................
Sus scrofa(AF402660)             .........................---..........................................
Sus scrofa(AF402661)             .........................---..........................................
Sus scrofa(AF402662)             .........................---..........................................
Sus scrofa(AF402663)             -----..AG.T.CCC-.TT...------------...T.T...C..............T..T........
Sus scrofa(DQ996276)             -----..AG.T.CC.-TT....GT.TTTT...TC...T.T...C..............T..T........
1 Sus scrofa C                   ............C............---......A...........T............T..........
8 Sus scrofa C                   ............C............---......A...........T............T..........
13 Sus scrofa C                  ............C............---......A...........T............T..........
1 Sus barbatus barbatus A        ............C............---......A...........T............T..........
2 Sus barbatus barbatus A        ............C............---......A...........T............T..........
11 Sus barbatus barbatus A       ............C............---......A...........T............T..........
12 Sus barbatus barbatus C       -----..AG.T.CC.-TT....------------...T.T...C..............T..T........
14 Sus barbatus barbatus C       -----..AG.T.CC.-TT....------------...T.T...C..............T..T........
17 Sus barbatus barbatus C       -----..AG.T.C..-TT....------------...T.T...C..............T...........
2 Sus barbatus oi A              ............C............---......A...........T............T..........
2 Sus barbatus oi C              -----..AG.T.CC.-TT....------------...T.T...C..............T..T........
3 Sus barbatus oi C              -----..AG.T.CC.-TT....------------...T.T...C..............T..T........
5 Sus barbatus oi C              -----..AG.T.C..-TT....------------...T.T...C..............T...........
1 Sus verrucosus C               -----..AG.T..C.-TT....------------...T.T...C............C.T..T........
3 Sus verrucosus A               -----..AG.T.CC.-TT....------------...T.T...C.GG...........T..T........
3 Sus verrucosus C               -----..AG.T..C.-TT....------------...T.T...C..............T..T........
5 Sus verrucosus A               ..........C.C............---.T........................................
5 Sus verrucosus C               -----..AG.T.CC.-TT....------------...T.T...C..............T..T........
7 Sus verrucosus A               -----..AG.T.CC.-TT...C------------...T.T...C..............T..T........
2 Sus celebensis C               -----..AG.T.CC.-TT....------------...T.T...C..............T..T........
14 Sus celebensis C              -----..AG.T.CC.-TT....------------...T.T...C..............T..T........
18 Sus celebensis C              -----..AG.T.CC.-TT....------------...T.T...C..............T..T........

                                         360       370       380       390       400       410       420
                                 ....|....|....|....|....|....|....|....|....|....|....|....|....|....|
Sus scrofa(AM229313)             TTTCACTGAGAAAGGAAAACAAGAAAATATCCTAAAATGGGTAAATGGTATGTCTTGGGGAATGGTATAT
Sus scrofa(AM229312)             ......................................................................
Sus scrofa(EU090250)             ......................................................................
Sus scrofa(AF417229)             ......................................................................
Sus scrofa(AF402660)             ......................................................................
Sus scrofa(AF402661)             ......................................................................
Sus scrofa(AF402662)             ......................................................................
```

Sus scrofa(AF402663)	`......C..A...AA.........................A.............C........AA.....`
Sus scrofa(DQ996276)	`......C..A...AA.........................A.............C........AA.....`
1 *Sus scrofa* C	`...................................................A.......A..........`
8 *Sus scrofa* C	`...................................................A.......A..........`
13 *Sus scrofa* C	`...................................................A.......A..........`
1 *Sus barbatus barbatus* A	`...................................................A............A.....`
2 *Sus barbatus barbatus* A	`...................................................A............A.....`
11 *Sus barbatus barbatus* A	`...................................................A............A.....`
12 *Sus barbatus barbatus* C	`......C..A............................................C........AA.....`
14 *Sus barbatus barbatus* C	`......C..A............................................C........AA.....`
17 *Sus barbatus barbatus* C	`......C..A............................................C........AA.....`
2 *Sus barbatus oi* A	`...................................................A..................`
2 *Sus barbatus oi* C	`......C..A............................................C........AA.....`
3 *Sus barbatus oi* C	`......C..A............................................C........AA.....`
5 *Sus barbatus oi* C	`......C..A............................................C........AA.....`
1 *Sus verrucosus* C	`......C..A..............G.............................C........AA.....`
3 *Sus verrucosus* A	`......C..A.T..........................................C........AA.....`
3 *Sus verrucosus* C	`......C..A..............T.............................C........AA.....`
5 *Sus verrucosus* A	`...................................................A..................`
5 *Sus verrucosus* C	`......C..A............................................C........AA.....`
7 *Sus verrucosus* A	`......C..A............................................C........AA.....`
2 *Sus celebensis* C	`......C..A............................................C........AA.....`
14 *Sus celebensis* C	`......C..A............................................C........AA.....`
18 *Sus celebensis* C	`......C..A............................................C....A...AA.....`

	`         430       440       450       460       470       480       490`
	`....|....|....|....|....|....|....|....|....|....|....|....|....|....|`
Sus scrofa(AM229313)	**TATGGAGGCTCGGGTAAACAACCAGGCTCCATTCTAACTATTCGCCTCAAAATAAACCAGCTGGAGCCTC**
Sus scrofa(AM229312)	`......................................................................`
Sus scrofa(EU090250)	`......................................................................`
Sus scrofa(AF417229)	`......................................................................`
Sus scrofa(AF402660)	`......................................................................`
Sus scrofa(AF402661)	`......................................................................`
Sus scrofa(AF402662)	`......................................................................`
Sus scrofa(AF402663)	`...AC...T.G..AC.G.....................C..C..A..T.......G......A.......`
Sus scrofa(DQ996276)	`...AC...T.G..AC.G.....................C..C..A..T.......G......A.......`
1 *Sus scrofa* C	`......................................................................`
8 *Sus scrofa* C	`......................................................................`
13 *Sus scrofa* C	`.......................................G..............................`
1 *Sus barbatus barbatus* A	`.............................T........................................`
2 *Sus barbatus barbatus* A	`.............................T........................................`
11 *Sus barbatus barbatus* A	`.............................T........................................`
12 *Sus barbatus barbatus* C	`....C...T.G...C.G.....................C..C..A..T.......G..............`
14 *Sus barbatus barbatus* C	`....C...T.G...C.G.....................C..C..A..T.......G..............`
17 *Sus barbatus barbatus* C	`....C...T.G...C.G.....................C..C..A..T.......G..............`
2 *Sus barbatus oi* A	`......................................................................`

2 *Sus barbatus oi* C	....C...T.G...C.G.....................C..C..A..T.......G..............
3 *Sus barbatus oi* C	....C...T.G...C.G.....................C..C..A..T.......G..............
5 *Sus barbatus oi* C	....C...T.G...C.G...............C.....C..C.AA..T.......G..............
1 *Sus verrucosus* C	....C...T.-...C.G.....................C..C..A..T.......G..............
3 *Sus verrucosus* A	....C...T.G...C.G.....................C..C..A..T.......G..............
3 *Sus verrucosus* C	....C...T.G...C.G.....................C..C..A..T.......G..............
5 *Sus verrucosus* A	...A..
5 *Sus verrucosus* C	....C...T.G...C.G.....................C..C..A..T.......G..............
7 *Sus verrucosus* A	....C...T.G...C.G.....................C..C..A..T.......G..............
2 *Sus celebensis* C	....C...T.G...C.G.....................C..C..A..T.......G..............
14 *Sus celebensis* C	....C...T.G...C.G.....................C..C..A..T.......G..............
18 *Sus celebensis* C	....C...T.G...C.G.....................C..C..A..T.......G..............

	500 510 520 530 540 550 560
	....|....|....|....|....|....|....|....|....|....|....|....|....|....|
Sus scrofa(AM229313)	**CAATGGCTATAGGACCAAATACGGTCTTGACGGGTCAAAGACCCCCAACCCAAGGACCAGGACCATCCTC**
Sus scrofa(AM229312)	....
Sus scrofa(EU090250)	....G.................
Sus scrofa(AF417229)	.........C..
Sus scrofa(AF402660)	....G
Sus scrofa(AF402661)	....
Sus scrofa(AF402662)	....
Sus scrofa(AF402663)	................G...........A............A........------.............A
Sus scrofa(DQ996276)	................G...........A............A........------..............
1 *Sus scrofa* C	...........................C.............T.....G..................T..T
8 *Sus scrofa* C	....T.....G..................T..T
13 *Sus scrofa* C	....T.....G..................T..T
1 *Sus barbatus barbatus* A	....T.....G..................T..T
2 *Sus barbatus barbatus* A	....T.....G..................T..T
11 *Sus barbatus barbatus* A	....T.....G..................T..T
12 *Sus barbatus barbatus* C	........G.......G...........A............A........------..............
14 *Sus barbatus barbatus* C	........G.......G...........A............A........------..............
17 *Sus barbatus barbatus* C	........G.......G........................T........................T..T
2 *Sus barbatus oi* A	....T.....G..................T..T
2 *Sus barbatus oi* C	........G......TG...........A............A........------..............
3 *Sus barbatus oi* C	........G.......G...........A............A........------..............
5 *Sus barbatus oi* C	........G.......G...........A.A..........A........------..............
1 *Sus verrucosus* C	........G.......G...........A............A........------..............
3 *Sus verrucosus* A	........G.......G...........A............A........------.............T
3 *Sus verrucosus* C	........G.......G...........A............A........------..............
5 *Sus verrucosus* A	......................A........................G......................
5 *Sus verrucosus* C	........G.......G...........A............A........------..............
7 *Sus verrucosus* A	........G.......G...........A............A........------..............
2 *Sus celebensis* C	........G.......G...........A............A........------..............
14 *Sus celebensis* C	........G.......G...........A............A........------..............
18 *Sus celebensis* C	........G.......G...........A............A........------..............

```
                                        570       580       590       600       610       620       630
                               ....|....|....|....|....|....|....|....|....|....|....|....|....|....|
Sus scrofa(AM229313)           TAACATAACTTCTGGATCAGACCCCACTGAGTCTAACAGCACGACTAAAATGGGGCAAAACTTTTTAGC
Sus scrofa(AM229312)           ......................................................................
Sus scrofa(EU090250)           ...................................G..................................
Sus scrofa(AF417229)           ......................................................................
Sus scrofa(AF402660)           ......................................................................
Sus scrofa(AF402661)           ......................................................................
Sus scrofa(AF402662)           ...................................G..................................
Sus scrofa(AF402663)           .G.T.........AA..T................................C....A.............T
Sus scrofa(DQ996276)           .G.T.........AA..T................................C....A.............T
1 Sus scrofa C                 .......................................T..........C....A..............
8 Sus scrofa C                 .......................................T..........C....A..............
13 Sus scrofa C                .......................................T..........C....A..............
1 Sus barbatus barbatus A      ...........T...........................T..........C....A..........A...
2 Sus barbatus barbatus A      ...........T...........................T..........C....A..........A...
11 Sus barbatus barbatus A     ...........T...........................T..........C....A..........A...
12 Sus barbatus barbatus C     .G.T..........A..T................................C..................T
14 Sus barbatus barbatus C     .G.T..........A..T................................C..................T
17 Sus barbatus barbatus C     .......................................T..........C....A..............
2 Sus barbatus oi A            .......................................T..........C....A..............
2 Sus barbatus oi C            .G.T..........A..T................................C..................T
3 Sus barbatus oi C            .G.T..........AG.T................................C..................T
5 Sus barbatus oi C            .G.T..........A..T...................................................T
1 Sus verrucosus C             .G.T..........A..T................................C..................T
3 Sus verrucosus A             ............................A..........T..........CA...A..............
3 Sus verrucosus C             .G.T..........A..T................................C..................T
5 Sus verrucosus A             ............................................T.....CA..................
5 Sus verrucosus C             .G.T..........A..T.......C...........................................T
7 Sus verrucosus A             .G.T..........A.GT.......C...........................................T
2 Sus celebensis C             .G.T..........A..T................................C..................T
14 Sus celebensis C            .G.T..........A..T...................................................T
18 Sus celebensis C            .G.T..........A..T................................C....A.............T

                                        640       650       660       670       680       690       700
                               ....|....|....|....|....|....|....|....|....|....|....|....|....|....|
Sus scrofa(AM229313)           CTCATCCAGGGAGCTTTTCAAGCTCTTAACTCCACGACTCCAGAGGCTACCTCTTCTTGTTGGCTATGCT
Sus scrofa(AM229312)           ......................................................................
Sus scrofa(EU090250)           ......................................................................
Sus scrofa(AF417229)           ......................................................................
Sus scrofa(AF402660)           .............................................A........................
Sus scrofa(AF402661)           ......................................................................
Sus scrofa(AF402662)           ......................................................................
Sus scrofa(AF402663)           .................................................................T....
Sus scrofa(DQ996276)           .................................................................T....
```

```
1 Sus scrofa C               .............A.........A.........................................T....
8 Sus scrofa C               .............A.........A.........................................T....
13 Sus scrofa C              .............A.........A.........................................T....
1 Sus barbatus barbatus A    .......................A.........................................T....
2 Sus barbatus barbatus A    .......................A.........................................T....
11 Sus barbatus barbatus A   .......................A.........................................T....
12 Sus barbatus barbatus C   .................................................................T....
14 Sus barbatus barbatus C   .................................................................T....
17 Sus barbatus barbatus C   .......................A.........................................T....
2 Sus barbatus oi A          .......................A.........................................T....
2 Sus barbatus oi C          .....................................................C...........T....
3 Sus barbatus oi C          .................................................................T....
5 Sus barbatus oi C          ..A..............................................................T....
1 Sus verrucosus C           .................................................................T....
3 Sus verrucosus A           ...................................A.............................T....
3 Sus verrucosus C           .................................................................T....
5 Sus verrucosus A           .................................................................T....
5 Sus verrucosus C           .........T.......................................................T....
7 Sus verrucosus A           .........T.......................................................T....
2 Sus celebensis C           ............................................................C....T....
14 Sus celebensis C          .................................................................T....
18 Sus celebensis C          .................................................................T....

                                     710       720       730       740       750       760       770
                             ....|....|....|....|....|....|....|....|....|....|....|....|....|....|
Sus scrofa(AM229313)         T-GGCTTCGGGCCCACCTTACTATGAAGGAATGGCTAGAAGAGGGAAATTCAATGTGACAAAAGAACATA
Sus scrofa(AM229312)         .-....................................................................
Sus scrofa(EU090250)         .-A....T..............................................................
Sus scrofa(AF417229)         .-A....T..............................................................
Sus scrofa(AF402660)         .-A....T..............................................................
Sus scrofa(AF402661)         .-A....T..............................................................
Sus scrofa(AF402662)         .-A....T..............................................................
Sus scrofa(AF402663)         .-AA....................A.GAA........A.....AA..................A......
Sus scrofa(DQ996276)         .-AA....................A.GAA........A.....AA..................A......
1 Sus scrofa C               .-A.......................G............G......................G...T...
8 Sus scrofa C               .-A.......................G............G......................G...T...
13 Sus scrofa C              .-A.......................G............G......................G...T...
1 Sus barbatus barbatus A    .-A.......................G............G......................G...T...
2 Sus barbatus barbatus A    .-A.......................G............G......................G...T...
11 Sus barbatus barbatus A   .-A.......................G............G......................G...T...
12 Sus barbatus barbatus C   .-A.......................G..........A................................
14 Sus barbatus barbatus C   .-A.......................G..........A................................
17 Sus barbatus barbatus C   .-A.......................G............G......................G...T...
2 Sus barbatus oi A          .TA.......................G............G.......T..............G...T...
2 Sus barbatus oi C          .-A....................A..G..........A................................
3 Sus barbatus oi C          .-A.......................G..........A................................
```

```
5 Sus barbatus oi C            .-A.....................G..............................A.......G.......
1 Sus verrucosus C             .AA.....................G............G.........................G.......
3 Sus verrucosus A             .-A.....A...............-....C.......G.........................G.......
3 Sus verrucosus C             .AA.....................G............G.........................G.......
5 Sus verrucosus A             .-A.....................G.........G..G..............................
5 Sus verrucosus C             .-A.....................G..........A................A...............
7 Sus verrucosus A             .-A.....................G..........A................A...............
2 Sus celebensis C             .-A.....................G..........A................................
14 Sus celebensis C            .-A.....................G..........A................................
18 Sus celebensis C            .-A..............-........-.A.......A.........-......................

                                        780       790       800       810       820       830       840
                               ....|....|....|....|....|....|....|....|....|....|....|....|....|....|
Sus scrofa(AM229313)           GAGACCAATGCACATGGGGATCCCAAAATAAGC-TTACCCTTACTGAGGTTTCTGGAAAA-GGCACCTGC
Sus scrofa(AM229312)           .................................-..........................-.........
Sus scrofa(EU090250)           .G...............................-..........................-.........
Sus scrofa(AF417229)           ..........T......................-..................T.......-.........
Sus scrofa(AF402660)           .................................-..........................-.........
Sus scrofa(AF402661)           .................................-..........................-.........
Sus scrofa(AF402662)           .................................-..........................-.........
Sus scrofa(AF402663)           ..........T......................-.........................G-.A.......
Sus scrofa(DQ996276)           ..........T......................-.........................G-.A.......
1 Sus scrofa C                 ..........T......................-....T.....................-.........
8 Sus scrofa C                 ..........T......................-..........................-.........
13 Sus scrofa C                ..........T......................-..........................-.........
1 Sus barbatus barbatus A      ..........T......................-..........................-.........
2 Sus barbatus barbatus A      ..........T......................-..........................-.........
11 Sus barbatus barbatus A     ..........T......................-..........................-.........
12 Sus barbatus barbatus C     ..........T......................-................C........G-.........
14 Sus barbatus barbatus C     ..........T......................-................C........G-.........
17 Sus barbatus barbatus C     ..........T......................-..........................-.........
2 Sus barbatus oi A            ..........T..........T..C....T...T..........................-.........
2 Sus barbatus oi C            ..........T................TA....-................C........G-.........
3 Sus barbatus oi C            ..........T......................-................C........G-.........
5 Sus barbatus oi C            ..........T.T....................-.........................G-........T
1 Sus verrucosus C             ..........T......................-..........................G.........
3 Sus verrucosus A             ..........T......................-......................G...-.........
3 Sus verrucosus C             ..........T......................-.........................G-.........
5 Sus verrucosus A             ..........T......................-.........................G-.........
5 Sus verrucosus C             ..........T..........T...........-.........................G-.........
7 Sus verrucosus A             ..........T......................-.........................G-.........
2 Sus celebensis C             ..........T..........-...........-................C........G-.........
14 Sus celebensis C            ..........T......................-................C........G-.........
18 Sus celebensis C            ....-.....T......................-................-........G-.........

                                        850       860       870       880       890       900       910
```

```
                              ....|....|....|....|....|....|....|....|....|....|....|....|....|....|
Sus scrofa(AM229313)          ATAGG-AAAGGTTCCCCCATCCCACCAACACCTTTGTAACCAC-ACTG-AAGCCTTTAATC-AAACCTCT
Sus scrofa(AM229312)          .....-.....................................-....-............-........
Sus scrofa(EU090250)          .....-.....................................-....-............-........
Sus scrofa(AF417229)          .....-.....................................-....-............-........
Sus scrofa(AF402660)          .....-.....................................-....-............-........
Sus scrofa(AF402661)          .....-.....................................-....-............-........
Sus scrofa(AF402662)          .....-.....................................-....-............-........
Sus scrofa(AF402663)          ...AA-.............................AC......-....-............-........
Sus scrofa(DQ996276)          ...AA-.......T.....................AC......-....-............-........
1 Sus scrofa C                .....-G.G..........................A.......-....-............-G.......
8 Sus scrofa C                .....-G.G..........................A.......-....-............-G.......
13 Sus scrofa C               .....-G.G..........................A.......-....-............-G.......
1 Sus barbatus barbatus A     .....-G.G..................................-....-............-G.......
2 Sus barbatus barbatus A     .....-G.G..................................-....-............-G.......
11 Sus barbatus barbatus A    .....-G.G..................................-....-............-G.......
12 Sus barbatus barbatus C    .....-..............................C......-....-............-........
14 Sus barbatus barbatus C    .....-..............................C......-....-............-........
17 Sus barbatus barbatus C    .....-G.G..................................-....-............-G.......
2 Sus barbatus oi A           .....-G.G..................................-....-............-G.......
2 Sus barbatus oi C           .....-..............................C......-....-............-........
3 Sus barbatus oi C           .....-..............................C......-....-............-........
5 Sus barbatus oi C           .....-G.............................C......-....-............-....A...
1 Sus verrucosus C            .....A..............................C......A....A............A....A...
3 Sus verrucosus A            .....-G.G..................................-....-............-G.......
3 Sus verrucosus C            .....-..............................C......-....-............-.G..A...
5 Sus verrucosus A            .....-..............................C......-....-............-....A...
5 Sus verrucosus C            .....-..............................C......-....-............-....A...
7 Sus verrucosus A            ..G..-..............................C......-....-.....C......-....A...
2 Sus celebensis C            .....-..............................C......-....-............-........
14 Sus celebensis C           .....-..............................C......-....-............-........
18 Sus celebensis C           ...A.-..............................C......-....-.........G..-........

                                      920       930       940       950       960       970       980
                              ....|....|....|....|....|....|....|....|....|....|....|....|....|....|
Sus scrofa(AM229313)          G-AGAGTCAATATCTGGTACCTGGTTATGACAGGTGGTGGGCATGTAATACTGGATTAAC-CCCTTGTGT
Sus scrofa(AM229312)          .-..........................................................-.........
Sus scrofa(EU090250)          .-..........................................................-.........
Sus scrofa(AF417229)          .-..........................................................-.........
Sus scrofa(AF402660)          .-..........................................................-.........
Sus scrofa(AF402661)          .-..........................................................-.........
Sus scrofa(AF402662)          .-..........................................................-.........
Sus scrofa(AF402663)          .-..........................................................-.........
Sus scrofa(DQ996276)          .-..........................................................-.........
1 Sus scrofa C                .-.......G..................................................-.........
8 Sus scrofa C                .-.......G.............C....................................T..T......
```

```
13 Sus scrofa C                 .-.......G..................................................-.........
1 Sus barbatus barbatus A       .-.......G..................................................-.........
2 Sus barbatus barbatus A       .-.......G..................................................-.........
11 Sus barbatus barbatus A      .-.......G..................................................-.........
12 Sus barbatus barbatus C      .-..........................................................-.........
14 Sus barbatus barbatus C      .-..........................................................-.........
17 Sus barbatus barbatus C      .-.......G..................................................-.........
2 Sus barbatus oi A             .-.......G..................................................-.........
2 Sus barbatus oi C             .-..............................C...........................-.........
3 Sus barbatus oi C             .-..........................................................-.........
5 Sus barbatus oi C             .-.......................................................G..-.........
1 Sus verrucosus C              .A.A........................................................-.........
3 Sus verrucosus A              .-.......G..C...............................................-.........
3 Sus verrucosus C              .-.A........................................................-.........
5 Sus verrucosus A              .-.A........................................................-.........
5 Sus verrucosus C              .-..........................................................-.........
7 Sus verrucosus A              .-..........................................................-.........
2 Sus celebensis C              .-..........................................................-.........
14 Sus celebensis C             .-..........................................................-.........
18 Sus celebensis C             .-..................................................A.......-.........

                                         990       1000      1010      1020      1030      1040      1050
                                ....|....|....|....|....|....|....|....|....|....|....|....|....|....|
Sus scrofa(AM229313)            TTCCACCTTGGTTTTTAACCAAACTAAAGATTTTTGCATTATGGTCCAAATTGTTCCCCGAGTGTATTAC
Sus scrofa(AM229312)            ......................................................................
Sus scrofa(EU090250)            ......................................................................
Sus scrofa(AF417229)            ......T...............................................................
Sus scrofa(AF402660)            ......T...............................................................
Sus scrofa(AF402661)            ......................................................................
Sus scrofa(AF402662)            ......................................................................
Sus scrofa(AF402663)            ...............C..............C....A........A..............C...A......
Sus scrofa(DQ996276)            ...............C..............C....A...........................A......
1 Sus scrofa C                  ...............C...A..........C......G................C.....G.....C...
8 Sus scrofa C                  ...............C...A..........C......G................C.....G.....C...
13 Sus scrofa C                 ...........C...C...A..........C......G................C.....G.....C...
1 Sus barbatus barbatus A       ...............C..............C......G................C.....G.....C...
2 Sus barbatus barbatus A       ...............C..............C......G................C.....G.....C...
11 Sus barbatus barbatus A      ...............C..............C......G................C.....G.....C...
12 Sus barbatus barbatus C      ...............C...T..........C.......................................
14 Sus barbatus barbatus C      ...............C...T..........C.......................................
17 Sus barbatus barbatus C      ...............C..............C......G............C...C.....G.....C...
2 Sus barbatus oi A             ...............C..............C......G................C.....G.....C...
2 Sus barbatus oi C             ...............C...T..........C.......................................
3 Sus barbatus oi C             ...............C...T..........C.......................................
5 Sus barbatus oi C             ...............C.......A......C.......................................
1 Sus verrucosus C              ...............C..............C...C...................................
```

```
3 Sus verrucosus A          .......C.......C..............C.....TG................C.....G.....C...
3 Sus verrucosus C          ...............C..............C...C...................................
5 Sus verrucosus A          ...............C..............C...C...................................
5 Sus verrucosus C          ...............C..............C.......................................
7 Sus verrucosus A          ...............C..............C...................................C...
2 Sus celebensis C          ...............C...T..........C.......................................
14 Sus celebensis C         ...............C...T..........C.......................................
18 Sus celebensis C         ...............C..............C.......................................

                                    1060      1070      1080      1090      1100      1110      1120
                            ....|....|....|....|....|....|....|....|....|....|....|....|....|....|
Sus scrofa(AM229313)        TATCCCGAAAAAGCAATCCTTGATAAATATGACTACAGAAATCATCGACAAAAGAGAGAACCCATATCTC
Sus scrofa(AM229312)        ......................................................................
Sus scrofa(EU090250)        ........................G.............................................
Sus scrofa(AF417229)        ........................G...........G.................................
Sus scrofa(AF402660)        ........................G.............................................
Sus scrofa(AF402661)        ........................G.............................................
Sus scrofa(AF402662)        ........................G.............................................
Sus scrofa(AF402663)        ......A.G...A....T..C...G.......T.....G..C.............A.A..........C.
Sus scrofa(DQ996276)        ......A.G...A....T..C...G.......T.....G..C.............A.A..........C.
1 Sus scrofa C              ............A..G........G..........T...T..............................
8 Sus scrofa C              ............A..G........G..........T...T..............................
13 Sus scrofa C             ............A..G........G..........T...T..............................
1 Sus barbatus barbatus A   ............A..G........G..........T...T..............................
2 Sus barbatus barbatus A   ............A..G........G..........T...T..............................
11 Sus barbatus barbatus A  ............A..G........G..........T...T..............................
12 Sus barbatus barbatus C  ........G........T..C...G.......T.....G..C..........................C.
14 Sus barbatus barbatus C  ........G........T..C...G.......T.....G..C.....G....................C.
17 Sus barbatus barbatus C  ............A..G........G..........T...T..............................
2 Sus barbatus oi A         ............A..G........G..........T...T..............................
2 Sus barbatus oi C         ........G........T..C...G.......T.....G..C.G........................C.
3 Sus barbatus oi C         ........G........T..C...G.......T.....G..C..........................C.
5 Sus barbatus oi C         ........G........T..C...G.......T.....G..C..........................C.
1 Sus verrucosus C          ........G........T..C...G.......T.....G..C..........................C.
3 Sus verrucosus A          ..............GG........G..........T...T..A....G.C...A..............C.
3 Sus verrucosus C          ........G........T..C...G.......T.....G..C..........................C.
5 Sus verrucosus A          ........G........T..C...G.......T.....G..C..........................C.
5 Sus verrucosus C          ........G........T..C...G.......T.....G..C..........................C.
7 Sus verrucosus A          ........G........T..C...G.......T.....G..C..........................C.
2 Sus celebensis C          ........G........T..C...G.......T.....G..C..........................C.
14 Sus celebensis C         ........G........T..C...G.......T.....G..C..........................C.
18 Sus celebensis C         ........G........T..C...G.......T.....G..C...............A..........C.

                                    1130      1140      1150      1160      1170      1180      1190
                            ....|....|....|....|....|....|....|....|....|....|....|....|....|....|
Sus scrofa(AM229313)        TGACACTTGCTGTGATGCTCGGACTTGGAGTGGCAGCAGGTGTAGGAACAGGAACAGCTGCCCTGGTCAC
```

Sus scrofa(AM229312)	....
Sus scrofa(EU090250)	....
Sus scrofa(AF417229)	....
Sus scrofa(AF402660)	....
Sus scrofa(AF402661)	....
Sus scrofa(AF402662)	....
Sus scrofa(AF402663)	.......C..A..A...........C......AT.A.......GA..........T..A..TT.A..T..
Sus scrofa(DQ996276)	.......C..A..A...........C......AT.A.......GA..........T..A..TT.A..T..
1 *Sus scrofa* C	........A...
8 *Sus scrofa* C	........A...C...........
13 *Sus scrofa* C	........A...
1 *Sus barbatus barbatus* A	....G.
2 *Sus barbatus barbatus* A	....G.
11 *Sus barbatus barbatus* A	....G.
12 *Sus barbatus barbatus* C	.......C.....A...........C.......T.........G...........T..A..TT.A..T..
14 *Sus barbatus barbatus* C	.......C.....A...........C.......T.........G...........T..A..TT.A..T..
17 *Sus barbatus barbatus* C	..........................A...G.
2 *Sus barbatus oi* A	....G.
2 *Sus barbatus oi* C	..T....C.....A...........C.......T.........G...........T..A..TT.A..T..
3 *Sus barbatus oi* C	..T....C.....A...........C.......T.........G.....G.....T..A..TT.A..T..
5 *Sus barbatus oi* C	.......C.....A...........C...............A.G...........T..A..TT.A..T..
1 *Sus verrucosus* C	.......C.....A...........C...A.............G...........T..A..TT.A..T..
3 *Sus verrucosus* A	.......A.....A.........T.G........T.....C..G...........G........AA....
3 *Sus verrucosus* C	.......C.....A...........C...A.............G...........T..A..TT.A..T..
5 *Sus verrucosus* A	.......C.....A...........C.......T.........G...........T..A..TT.A..T..
5 *Sus verrucosus* C	.......C.....A...........C.......T.........G...........T..A..TT.A..T..
7 *Sus verrucosus* A	.......C.....A...........C.......T.........G...........T..A..TT.A..T..
2 *Sus celebensis* C	.......C.....A...........C.......T......C..G..............A..TT.A..T..
14 *Sus celebensis* C	.......C.....A...........C.......T.........G...........T..A..TT.A..T..
18 *Sus celebensis* C	.......C.....A...........C...A...T.A...................T..A..TT.A..T..
	1200 1210 1220 1230 1240 1250 1260
	....|....|....|....|....|....|....|....|....|....|....|....|....|....|
Sus scrofa(AM229313)	**GGGACCACAGCAGCTAGAAACAGGACTTAGTAACCTACATCGAATTGTAACAGAAGATCTCCAAGCCCTA**
Sus scrofa(AM229312)	....
Sus scrofa(EU090250)	....
Sus scrofa(AF417229)	....
Sus scrofa(AF402660)	....
Sus scrofa(AF402661)	....
Sus scrofa(AF402662)	....
Sus scrofa(AF402663)	A.....T..................................A...........G.A..............
Sus scrofa(DQ996276)	A.....T..................................A...........G.A..............
1 *Sus scrofa* C	....
8 *Sus scrofa* C	....
13 *Sus scrofa* C	....
1 *Sus barbatus barbatus* A	..................................T...................................

```
2 Sus barbatus barbatus A     ..................................T...................................
11 Sus barbatus barbatus A    ..................................T...................................
12 Sus barbatus barbatus C    A.....T...............................................................
14 Sus barbatus barbatus C    A.....T...............................................................
17 Sus barbatus barbatus C    ..................................T...................................
2 Sus barbatus oi A           ..................................T...................................
2 Sus barbatus oi C           A.....T...............................................................
3 Sus barbatus oi C           A.....T...............................................................
5 Sus barbatus oi C           A.....T.....................................................T.........
1 Sus verrucosus C            A.....T.............................................A.................
3 Sus verrucosus A            A........A.....G..G.A..........G...................G..................
3 Sus verrucosus C            A.....T.............................................A.................
5 Sus verrucosus A            A.....T...............................................................
5 Sus verrucosus C            A.....T...............................................................
7 Sus verrucosus A            A.....T...............................................................
2 Sus celebensis C            A.....T...............................................................
14 Sus celebensis C           A.....T...............................................................
18 Sus celebensis C           AA....T...............................................................

                                       1270      1280      1290      1300      1310      1320      1330
                              ....|....|....|....|....|....|....|....|....|....|....|....|....|....|
Sus scrofa(AM229313)          GAAAAATCTGTCAGTAACCTGGAGGAATCCCTAACCTCCTTATCTGAAGTAGTCCTACAGAATAGAAGAG
Sus scrofa(AM229312)          ......................................................................
Sus scrofa(EU090250)          ......................................................................
Sus scrofa(AF417229)          ......................................................................
Sus scrofa(AF402660)          ......................................................................
Sus scrofa(AF402661)          ......................................................................
Sus scrofa(AF402662)          ......................................................................
Sus scrofa(AF402663)          A......................AA............................T..........A..A..
Sus scrofa(DQ996276)          A......................AA............................T..........A..A..
1 Sus scrofa C                ..................................................G..T........C.....G.
8 Sus scrofa C                ................G.................................G..T........C.....G.
13 Sus scrofa C               ..................................................G..T........C.....G.
1 Sus barbatus barbatus A     ..................................................G..T........C.....G.
2 Sus barbatus barbatus A     ..................................................G..T........C.....G.
11 Sus barbatus barbatus A    ..................................................G..T........C.....G.
12 Sus barbatus barbatus C    ......................................................................
14 Sus barbatus barbatus C    ......................................................................
17 Sus barbatus barbatus C    ................................G.....T...............................
2 Sus barbatus oi A           ..................................................G..T........C.....G.
2 Sus barbatus oi C           ......................................................................
3 Sus barbatus oi C           ......................................................................
5 Sus barbatus oi C           ................................G.....T...............................
1 Sus verrucosus C            ......................................................................
3 Sus verrucosus A            ....................A.............................G..T........C.......
3 Sus verrucosus C            ......................................................................
```

```
5 Sus verrucosus A               ......................................................................
5 Sus verrucosus C               ......................................................................
7 Sus verrucosus A               ......................................................................
2 Sus celebensis C               ...................C..................................................
14 Sus celebensis C              ......................................................................
18 Sus celebensis C              A.....................................................................

                                          1340      1350      1360      1370      1380      1390      1400
                                 ....|....|....|....|....|....|....|....|....|....|....|....|....|....|
Sus scrofa(AM229313)             GGTTAGATTTATTATTTCTAAAAGAAGGAGGATTATGTGTAGCCTTGAAGGAGGAATGCTGTTTTTATGT
Sus scrofa(AM229312)             ......................................................................
Sus scrofa(EU090250)             ......................................................................
Sus scrofa(AF417229)             ......................................................................
Sus scrofa(AF402660)             ......................................................................
Sus scrofa(AF402661)             ......................................................................
Sus scrofa(AF402662)             ......................................................................
Sus scrofa(AF402663)             .......................A..A..A................A......A................
Sus scrofa(DQ996276)             .......................A..A..A................A......A................
1 Sus scrofa C                   ........C.G....................G..............A..A...........C........
8 Sus scrofa C                   ........C.G....................G..............A..A...........C........
13 Sus scrofa C                  ........C.G....................G..............A..A...........C........
1 Sus barbatus barbatus A        ........C.G....................G..............A..A...........C........
2 Sus barbatus barbatus A        ........C.G....................G..............A..A...........C........
11 Sus barbatus barbatus A       ........C.G....................G..............A..A...........C........
12 Sus barbatus barbatus C       ......................................................................
14 Sus barbatus barbatus C       ......................................................................
17 Sus barbatus barbatus C       ...........C..........................................................
2 Sus barbatus oi A              ........C.G....................G..............A..A........A..C........
2 Sus barbatus oi C              ......................................................................
3 Sus barbatus oi C              ..........................................................G...........
5 Sus barbatus oi C              ...........C..........................................................
1 Sus verrucosus C               ...........C..........................................................
3 Sus verrucosus A               ........C.G.................T..G..............A..A..A.....T..C..C.....
3 Sus verrucosus C               ...........C..........................................................
5 Sus verrucosus A               ......................................................................
5 Sus verrucosus C               ......................................................................
7 Sus verrucosus A               ...........C..........................................................
2 Sus celebensis C               ......................................................................
14 Sus celebensis C              ......................................................................
18 Sus celebensis C              ......................................................................

                                          1410      1420      1430      1440      1450      1460      1470
                                 ....|....|....|....|....|....|....|....|....|....|....|....|....|....|
Sus scrofa(AM229313)             GGATCATTCAGGGGCCATCAGAGACTCCATGAACAAGCTTAGAGAAAGGTTGGAGAAGCGTCGAAGGGAA
Sus scrofa(AM229312)             ...........................................A..........................
Sus scrofa(EU090250)             ....................................A.................................
```

Sus scrofa(AF417229)	.....................G..
Sus scrofa(AF402660)	....
Sus scrofa(AF402661)	....................................A.................................
Sus scrofa(AF402662)	....................................A.................................
Sus scrofa(AF402663)	A..A.A.............A............
Sus scrofa(DQ996276)	A..A.A.............A............
1 *Sus scrofa* C	............A..T.....G..........G......C...........A..A..A....AC.A....
8 *Sus scrofa* C	............A..T.....G..........G......C...........A..A..A....AC.A....
13 *Sus scrofa* C	............A..T.....G..........G......C...........A..A..A....AC.A....
1 *Sus barbatus barbatus* A	............A..T.....G.................C...........A..A..A....AC.AA...
2 *Sus barbatus barbatus* A	............A..T.....G.................C...........A..A..A....AC.AA...
11 *Sus barbatus barbatus* A	............A..T.....G.................C...........A..A..A....AC.AA...
12 *Sus barbatus barbatus* C	................................G.....................................
14 *Sus barbatus barbatus* C	................................G.....................................
17 *Sus barbatus barbatus* C	................................G..................A..................
2 *Sus barbatus oi* A	............A..T.....G..........G......C...........A..A..A....AC.AA...
2 *Sus barbatus oi* C	................................G...........................C.........
3 *Sus barbatus oi* C	................................G.....................................
5 *Sus barbatus oi* C	................................G..................A..................
1 *Sus verrucosus* C	..........T.....................G..................A..............A...
3 *Sus verrucosus* A	A.....C.....A...................G..................A....G.............
3 *Sus verrucosus* C	................................G..................A..............A...
5 *Sus verrucosus* A	................................G................C....................
5 *Sus verrucosus* C	................................G............................T........
7 *Sus verrucosus* A	................................G..................A..............A...
2 *Sus celebensis* C	.........G..
14 *Sus celebensis* C	................................G.....................................
18 *Sus celebensis* C	................................G..........A........A.........A...A...

	1480 1490 1500 1510 1520 ....|....|....|....|....|....|....|....|....|....|.
Sus scrofa(AM229313)	**AAGGAAACTACTCAAGGGTGGTTTGAGGGATGGTTCAACAGGTCTCCTTGG**
Sus scrofa(AM229312)	....
Sus scrofa(EU090250)	....
Sus scrofa(AF417229)	....
Sus scrofa(AF402660)	....
Sus scrofa(AF402661)	....
Sus scrofa(AF402662)	....
Sus scrofa(AF402663)	...............A.A......A.AA.......................
Sus scrofa(DQ996276)	...............A.A......A.AA.......................
1 *Sus scrofa* C	..A..GG..GGC.....A......................A...C..A...
8 *Sus scrofa* C	..A..GG..GGC.....A......................A...C..A...
13 *Sus scrofa* C	..A..GG..GGC.....A......................A...C..A...
1 *Sus barbatus barbatus* A	..A..GG..GGC.....A......................A...C..A...
2 *Sus barbatus barbatus* A	..A..GG..GGC.....A......................A...C..A...
11 *Sus barbatus barbatus* A	..A..GG..GGC.....A......................A...C..A...

12 *Sus barbatus barbatus* C	....
14 *Sus barbatus barbatus* C	....
17 *Sus barbatus barbatus* C	..........................**A**..**G**..............**C**......
2 *Sus barbatus oi* A	..**A**..**GG**..**GGC**.....**A**......................**A**...**C**..**A**...
2 *Sus barbatus oi* C	....
3 *Sus barbatus oi* C	....
5 *Sus barbatus oi* C	..........................**A**.................**C**......
1 *Sus verrucosus* C	..........................**A**........................
3 *Sus verrucosus* A	.**GA**..**GG**..**GAC**..**G**...........**A**......**C**.................
3 *Sus verrucosus* C	..........................**A**........................
5 *Sus verrucosus* A	..........................**A**........................
5 *Sus verrucosus* C	....
7 *Sus verrucosus* A	..........................**A**........................
2 *Sus celebensis* C	....
14 *Sus celebensis* C	....
18 *Sus celebensis* C	..**A**..
