# Supplementary material for: Evolution of endogenous retroviruses in the Suidae: evidence for different viral subpopulations in African and Eurasian host species
Source: BMC Evol Biol. 2011 May 24;11:139. doi: 10.1186/1471-2148-11-139 (PMC3128044; doi:10.1186/1471-2148-11-139)
Supplement: Additional file 7 — env E alignment. env E alignment of sequences generated in this study, sequences from GenBank and the draft pig genome [file 1471-2148-11-139-S7.PDF]

env E alignment (including all sequences)

The first number next to some sequence names represents the clone number. The second number next to the *Babyrousa babyrussa* represents the animal number, which were two animals analyzed.

```
                                           10        20        30        40        50        60        70
                                   ....|....|....|....|....|....|....|....|....|....|....|....|....|....|
Seq1 Ssrcofa8 chromosome1          CCGGAAGCTGGTCAGTCAACGCACAGCTAAAGCTTGAGAAAACGGCTGCCTGTGAGCCCAAATGGAAGGA
Seq2 Ssrcofa8 chromosome2          ..A.................................TG...G.........G..T.A..........................
Seq3 Ssrcofa8 chromosome2          .......................TG...G.........G..T...................G........
Seq4 Ssrcofa8 chromosome6          ........................G...G.........G..T...T........................
Seq5 Ssrcofa8 chromosome7          ........................G...G.........G..T....A.................A.....
Seq6 Ssrcofa8 chromosome9          ........................G...G.........G..T.A...........A..............
Seq7 Ssrcofa8 chromosomeX          .......................TG...G............TT...........................
Seq8 Ssrcofa8 chromosomeX          ........................G...G.........G..T.........T..................
Sus scrofa(AF356698)               ..A....................TG...G............T.A..........................
3 Sus scrofa                       .T......................G...G.........G..T...T........................
7 Sus scrofa                       ......................................................................
1 Sus barbatus barbatus            ........................G...G.........G..T....A.................A.....
2 Sus barbatus barbatus            ........................G...G.........G..T....AA..C...................
7 Sus barbatus oi                  ...................T....G...G.........G..T....A.T.....................
5 Sus barbatus oi                  .......................TG...G.........G..T...................G........
10 Sus barbatus oi                 .......................TG...G.........G..T...................G........
1 Sus verrucosus                   ........................G...G.........G..T.A...........A..............
3 Sus verrucosus                   ........................G...G.........G..T.A..A.G...............A.....
4 Sus verrucosus                   ............................G.........G..T............................
10 Sus verrucosus                  ....................A.......G............T............................
1 Sus celebensis                   ..A...................................................................
5 Sus celebensis                   G.A.........................G.........G..T.A..A.......................
7 Sus celebensis                   .......................TG...G.........G..T...................G........
8 Potamochoerus larvatus           ..A.................A....A..G...T.....G..T....A.T.....................
10 Potamochoerus larvatus          ...............A........G...G.........GC.TT...A.......................
2 Potamochoerus porcus             ........................G...G.........GC.T....................C.......
4 Potamochoerus porcus             ...................A....G...G.........G..TG...A.................A.....
6 Potamochoerus porcus             ........................G...G............TT.........A.................
1 Hylochoerus meinertzhageni       ............T.......A..GG...G.........G..T.A...........A..............
4 Hylochoerus meinertzhageni       ............T.......A..GG...G.........G..T.A...........A..............
5 Hylochoerus meinertzhageni       ..A.....................G...G.........GC.T............................
3 Phacochoerus africanus           ........................G...G.........G..T....A.................A.....
4 Phacochoerus africanus           ........................G...G.........G..T.A..A.......................
5 Phacochoerus africanus           ........................G...G.........G..T.A..A.......................
7 Phacochoerus africanus           .......................TG................TT.....................A.....
4 Phacochoerus aethiopicus         ........................G...G.........G..TT...........................
6 Phacochoerus aethiopicus         ........................T................TT.....................A.....
7 Phacochoerus aethiopicus         ........................G...G.........G..T.A...........A..............
1 2 Babyrousa babyrussa            ..A................T...TG...G.........G..T.A...........A..............
```

```
2 2 Babyrousa babyrussa          .......................TG...G.........G..T.A..........................
3 2 Babyrousa babyrussa          ..A................T...TG...G.........G..T.A...........A..............
4 2 Babyrousa babyrussa          .......................TG...G.........G..T.A..........................
1 24 Babyrousa babyrussa         ..A.................A.......G.......C.G..T.A.........................C
3 24 Babyrousa babyrussa         ..A..............C......G..AG.........G..T.....................A......
4 24 Babyrousa babyrussa         ........................G...G.........G.GT............................
5 24 Babyrousa babyrussa         .............G..........G...G.........G..T............................
6 24 Babyrousa babyrussa         ........................G...G.........G..T.....................A......
7 24 Babyrousa babyrussa         .......................AG...G.........G..T............................
9 24 Babyrousa babyrussa         .......................TG...G.........G..T.A..........................

                                          80        90        100       110       120       130       140
                                 ....|....|....|....|....|....|....|....|....|....|....|....|....|....|
Seq1 Ssrcofa8 chromosome1        GGGTTTCAAAATAAACATTCACATTGACGGAAGAGGCCTTGACCCTGGAGCTGTGCTACATT--------
Seq2 Ssrcofa8 chromosome2        ..............G....TG......T.....................A.......G....--------
Seq3 Ssrcofa8 chromosome2        ...........G..G....TG......T.A...................A............--------
Seq4 Ssrcofa8 chromosome6        ....................G................T.................T......--------
Seq5 Ssrcofa8 chromosome7        T...........................------------------................TCCAGAGA
Seq6 Ssrcofa8 chromosome9        ..............G....TG.......A..........A......................--------
Seq7 Ssrcofa8 chromosomeX        ................................A.............................--------
Seq8 Ssrcofa8 chromosomeX        .A............................................................--------
Sus scrofa(AF356698)             ..............G.....G........A........................A.......--------
3 Sus scrofa                     ....................G.......C..........................T......--------
7 Sus scrofa                     ..............................................................--------
1 Sus barbatus barbatus          T...........................------------------................TCCAGAGA
2 Sus barbatus barbatus          ...................TG...............T............A.........C..--------
7 Sus barbatus oi                ...........................T..................................--------
5 Sus barbatus oi                ..............G....TG......T.A...................A............--------
10 Sus barbatus oi               ..............G....TG......T.A...................A............--------
1 Sus verrucosus                 ..............G....TG..................A......................--------
3 Sus verrucosus                 ..............G.....G.......A.................................--------
4 Sus verrucosus                 ..............G....TG......T....A..........T..................--------
10 Sus verrucosus                .C.................TG.C..A.T.A...................A............--------
1 Sus celebensis                 ..............................................................--------
5 Sus celebensis                 ..............G....TG.........................................--------
7 Sus celebensis                 ..............G....TG......T.A...................A............--------
8 Potamochoerus larvatus         ..............G.....G......T.....G......A......C..............--------
10 Potamochoerus larvatus        ..............G.....G.........................................--------
2 Potamochoerus porcus           ..............G.....G.........G.................G.............--------
4 Potamochoerus porcus           ....................G....A...A..............T.................--------
6 Potamochoerus porcus           ..............G.....G........A........................A.......--------
1 Hylochoerus meinertzhageni     ..A...........G.....G......T.....................A............--------
4 Hylochoerus meinertzhageni     ..A...........G.....G......T.....................A............--------
5 Hylochoerus meinertzhageni     ..............G.....G.........................................--------
3 Phacochoerus africanus         ....................G....A...A..............T.................--------
4 Phacochoerus africanus         ..............G.....G....A...A..A.............................--------
```

```
5 Phacochoerus africanus     ..............G.....G....A...A..A.............................--------
7 Phacochoerus africanus     ..............G.....G.........................................--------
4 Phacochoerus aethiopicus   ..............G.....G......T..................................--------
6 Phacochoerus aethiopicus   ..............G....TG......T........G.........................--------
7 Phacochoerus aethiopicus   ..............G.....G.......A..........A......................--------
1 2 Babyrousa babyrussa      ..............G.....G..................A......................--------
2 2 Babyrousa babyrussa      A.............G....TG......T....A.............................--------
3 2 Babyrousa babyrussa      ..............G.....G..................A......................--------
4 2 Babyrousa babyrussa      A.............G....TG......T....A.............................--------
1 24 Babyrousa babyrussa     ..............G.............A.................................--------
3 24 Babyrousa babyrussa     ................................A.............................--------
4 24 Babyrousa babyrussa     ..............................................................--------
5 24 Babyrousa babyrussa     ..............G.....G......T..................................--------
6 24 Babyrousa babyrussa     ..............G.....G......T..........................A.......--------
7 24 Babyrousa babyrussa     ..............G.....G.........................................--------
9 24 Babyrousa babyrussa     A.............G....TG......T....A.............................--------

                                     150       160       170       180       190       200       210
                             ....|....|....|....|....|....|....|....|....|....|....|....|....|....|
Seq1 Ssrcofa8 chromosome1    -TCCAGAGAATCACATTTGCGCTCCAAGCTGCCTCTCACAGTGTCTTTCATTCCTTTTATAAAGAGAT-A
Seq2 Ssrcofa8 chromosome2    -.............G....T.....................G..................G.......-.
Seq3 Ssrcofa8 chromosome2    -.............G....GC..--................G..........................-.
Seq4 Ssrcofa8 chromosome6    -........................................CA.................G.......-.
Seq5 Ssrcofa8 chromosome7    A...........GTG..........................C..........................-.
Seq6 Ssrcofa8 chromosome9    -............TG.....A....................G..................G.......-.
Seq7 Ssrcofa8 chromosomeX    -.............G....T........G............G..........................-.
Seq8 Ssrcofa8 chromosomeX    -.............G....T.....................G..................G.......A.
Sus scrofa(AF356698)         -......A......G..........................G..................G.......-.
3 Sus scrofa                 -........................................CA....A............G.......-.
7 Sus scrofa                 -...................................................................-.
1 Sus barbatus barbatus      A...........GTG..........................C..........................-.
2 Sus barbatus barbatus      -............TG....T........G...T........CA.................G.......-.
7 Sus barbatus oi            -.............G.....A....................G..................G.......A.
5 Sus barbatus oi            -.............G.....C..--.....T..........G......................C...-.
10 Sus barbatus oi           -.............G.....C..--....CT..........G......................C...-.
1 Sus verrucosus             -............TG..........................G..................G.......-.
3 Sus verrucosus             -.............G.............................................G......C-.
4 Sus verrucosus             -.............G..........................CA.................G.......-.
10 Sus verrucosus            -......A......G.....A....................G.....................A....-.
1 Sus celebensis             -...................T....................G..........................-.
5 Sus celebensis             -.............G....T.....................C..................G.......-.
7 Sus celebensis             -.............G.....C..--.....T..........G......................C.G.-.
8 Potamochoerus larvatus     -.............G....T.....................CA.........-.......G.......-.
10 Potamochoerus larvatus    -.............G..........................C..................G.......-G
2 Potamochoerus porcus       -.............G..........................C..................G....A..-G
4 Potamochoerus porcus       -..T..........G....T.....................CA.................G.......-.
```

```
6 Potamochoerus porcus           -......A......G.....A.......T............G..................G.......-.
1 Hylochoerus meinertzhageni     -............TG..A.......................G..T.......................--
4 Hylochoerus meinertzhageni     -............TG..A.......................G..T.......................--
5 Hylochoerus meinertzhageni     -.............G..........................CA.................G.......-.
3 Phacochoerus africanus         -.TT..........G....T................T....C..................G.......-.
4 Phacochoerus africanus         -........G....G..........................C..................G.......-.
5 Phacochoerus africanus         -........G....G..........................C..................G.......-.
7 Phacochoerus africanus         -................A.A.....................G..................G.......-.
4 Phacochoerus aethiopicus       -..A.........GG...A.A....................G..........................-.
6 Phacochoerus aethiopicus       -.............G....A.....................G.......C..........G.......-.
7 Phacochoerus aethiopicus       -............TG..........................G..................G.......-.
1 2 Babyrousa babyrussa          -............TG....T.......T.............G..................G......G-.
2 2 Babyrousa babyrussa          -.............G....T.....................G..........................-.
3 2 Babyrousa babyrussa          -............TG....T.......T.............G..................G......G-.
4 2 Babyrousa babyrussa          -.............G....T.....................G..........................-.
1 24 Babyrousa babyrussa         -............TG....T....T.T..............G..........................-.
3 24 Babyrousa babyrussa         -.............G....T.....................G..........................-.
4 24 Babyrousa babyrussa         -.............G.....A....................G..........................-T
5 24 Babyrousa babyrussa         -............AG.............G............C..................G.......-.
6 24 Babyrousa babyrussa         -............TG..........................G.......................A..-.
7 24 Babyrousa babyrussa         -.............G..........................C..................G.......-.
9 24 Babyrousa babyrussa         -.............G....T.....................G..........................-.

                                          220       230       240       250       260       270       280
                                 ....|....|....|....|....|....|....|....|....|....|....|....|....|....|
Seq1 Ssrcofa8 chromosome1        AGAAACACCCCACCTCCCATTCTGGCCACAACTAAGAACTTGTTCTTAGCCCTGGCAGAAACTATTGCAC
Seq2 Ssrcofa8 chromosome2        .A....................C...............................................
Seq3 Ssrcofa8 chromosome2        ...................A........T.........................................
Seq4 Ssrcofa8 chromosome6        ......................C...........C...................A...........A...
Seq5 Ssrcofa8 chromosome7        ......................C........T......................................
Seq6 Ssrcofa8 chromosome9        .A...T................CA..............................................
Seq7 Ssrcofa8 chromosomeX        .A....................C...............................................
Seq8 Ssrcofa8 chromosomeX        .A....................CCA.............................................
Sus scrofa(AF356698)             .A.......................T...........................A...A............
3 Sus scrofa                     ......................C...........C...................A...........A...
7 Sus scrofa                     ......................................................................
1 Sus barbatus barbatus          ......................C........T.............-........................
2 Sus barbatus barbatus          ..........T...........C........................................G......
7 Sus barbatus oi                .A........T...........CA............................C.................
5 Sus barbatus oi                ............................T.........................................
10 Sus barbatus oi               ............................T.........................................
1 Sus verrucosus                 .A...T................C...............................................
3 Sus verrucosus                 .............................................................A....A...
4 Sus verrucosus                 ......................CC..............................................
10 Sus verrucosus                .A..........................T............................A....--..A...
1 Sus celebensis                 ...................................................T.C................
```

```
5 Sus celebensis                .A....................C...............................................
7 Sus celebensis                ............................T.........................................
8 Potamochoerus larvatus        ......................................................................
10 Potamochoerus larvatus       ...........T..A....................C....................C.............
2 Potamochoerus porcus          ......................C...............................................
4 Potamochoerus porcus          ......................................................................
6 Potamochoerus porcus          .A.......................T...............................A............
1 Hylochoerus meinertzhageni    --....................CC....T.........................................
4 Hylochoerus meinertzhageni    --....................CC....T.........................................
5 Hylochoerus meinertzhageni    ......................C...............................................
3 Phacochoerus africanus        ......................C...............................................
4 Phacochoerus africanus        ......................C............T..................................
5 Phacochoerus africanus        ......................C...............................................
7 Phacochoerus africanus        .A....................C...............................................
4 Phacochoerus aethiopicus      .....................TC..................A..................G.....A...
6 Phacochoerus aethiopicus      .A....................C........................G......................
7 Phacochoerus aethiopicus      .A...T................C......T........................................
1 2 Babyrousa babyrussa         .A....................CA..............................................
2 2 Babyrousa babyrussa         ......................C.....T.........T...............................
3 2 Babyrousa babyrussa         .A....................CA..............................................
4 2 Babyrousa babyrussa         ......................C.....T.........T...............................
1 24 Babyrousa babyrussa        .A....................................................................
3 24 Babyrousa babyrussa        ..................................................................A...
4 24 Babyrousa babyrussa        .A....................C...............................................
5 24 Babyrousa babyrussa        ......................C...............................................
6 24 Babyrousa babyrussa        .A....................C...............................A...........A...
7 24 Babyrousa babyrussa        ......................C...............................................
9 24 Babyrousa babyrussa        ......................C.....T.........T...............................

                                         290       300       310       320       330       340       350
                                ....|....|....|....|....|....|....|....|....|....|....|....|....|....|
Seq1 Ssrcofa8 chromosome1       AAACTCTCAAGGTCTCCTCTTGTTATATATGTGGGGGAACAAACACAGGAGACCACTGGCCTTGGCAAGC
Seq2 Ssrcofa8 chromosome2       ..........................G....C........G.............................
Seq3 Ssrcofa8 chromosome2       ..........A...............G.G..C....A..TG.............................
Seq4 Ssrcofa8 chromosome6       ..........................G....C......................................
Seq5 Ssrcofa8 chromosome7       ..........................G.............G.............................
Seq6 Ssrcofa8 chromosome9       .........................C.....CA......TG.........T...................
Seq7 Ssrcofa8 chromosomeX       ...T......................G...AC......................................
Seq8 Ssrcofa8 chromosomeX       .............T.T..........G.............................C.............
Sus scrofa(AF356698)            ..........................G....C........G.............................
3 Sus scrofa                    ..........................G....C......................................
7 Sus scrofa                    ......................................................TT......C.......
1 Sus barbatus barbatus         ..........................G...........................................
2 Sus barbatus barbatus         ........................G.G....C......................................
7 Sus barbatus oi               ...........A..............G.T...........G.....................A.......
5 Sus barbatus oi               ..........A...............G.G..C....A..TG.............................
```

```
10 Sus barbatus oi               ..........A...............G.G..C....A..TG.............................
1 Sus verrucosus                 .........................CG...C.......TG.............................
3 Sus verrucosus                 .....T....................G.........................G.................
4 Sus verrucosus                 ..........................G....C...........TG...................C.....
10 Sus verrucosus                ..........................G.........................G.................
1 Sus celebensis                 ..................T...................................................
5 Sus celebensis                 ..........................GC...C......................................
7 Sus celebensis                 ..........A...............G.G..C....A..TG.............................
8 Potamochoerus larvatus         ..........................GC...C.......TG..T..........................
10 Potamochoerus larvatus        ..........................G....C........G..............G.......A......
2 Potamochoerus porcus           ..........................G....C........G..................T..........
4 Potamochoerus porcus           ........T...............................G.............................
6 Potamochoerus porcus           ..........................G.............G.............................
1 Hylochoerus meinertzhageni     ..........................G....C.......A.G............................
4 Hylochoerus meinertzhageni     ..........................G....C.......A.G............................
5 Hylochoerus meinertzhageni     ..........................G.............G......................A......
3 Phacochoerus africanus         ..........................G....C..A...................................
4 Phacochoerus africanus         .......G..................G....C......................................
5 Phacochoerus africanus         .......G..................G....C......................................
7 Phacochoerus africanus         ..........................G.G.............G...........................
4 Phacochoerus aethiopicus       .................G........G....CA.......G.............................
6 Phacochoerus aethiopicus       .....-....................G.G.........................................
7 Phacochoerus aethiopicus       .............---------------...C.......TG.............................
1 2 Babyrousa babyrussa          .........................CG...C.......TG.............................
2 2 Babyrousa babyrussa          ..........................G....C.......TG.............................
3 2 Babyrousa babyrussa          .........................CG...C.......TG.............................
4 2 Babyrousa babyrussa          ..........................G....C.......TG.............................
1 24 Babyrousa babyrussa         ........................T.GC...CC.......G...............CA............
3 24 Babyrousa babyrussa         ..........................G....CA......TG.............................
4 24 Babyrousa babyrussa         ..........................G....CA......TG.............................
5 24 Babyrousa babyrussa         ..........................G.............G.............................
6 24 Babyrousa babyrussa         ..........................G....C.......TG.............................
7 24 Babyrousa babyrussa         .......TG.................G.............G.............................
9 24 Babyrousa babyrussa         ..........................G....C.......TG.............................

                                          360       370       380       390       400       410       420
                                 ....|....|....|....|....|....|....|....|....|....|....|....|....|....|
Seq1 Ssrcofa8 chromosome1        CAGAAAACTGAACCCATTAGAGCCTCATAATGAGACTACATACCCCCAACTCACCAATGGGCTATGGCTC
Seq2 Ssrcofa8 chromosome2        ..........G..........................G.......................G........
Seq3 Ssrcofa8 chromosome2        ....G..A..G..............A.......A...G................TG.....G........
Seq4 Ssrcofa8 chromosome6        ....G.G...G...............C..........G.......................G........
Seq5 Ssrcofa8 chromosome7        ..........GG....................................G............G........
Seq6 Ssrcofa8 chromosome9        ....G...G.G..........................G........T..............G........
Seq7 Ssrcofa8 chromosomeX        ....G.....G..........A.................................G.....G.G......
Seq8 Ssrcofa8 chromosomeX        ..........G..........................G.................G.....G........
Sus scrofa(AF356698)             ....G..A..G..........................G................TG.....G........
```

```
3 Sus scrofa                     ...GG.G...G...............C..........G.......................G........
7 Sus scrofa                     ...GG.........TG.....................GT......................G........
1 Sus barbatus barbatus          ..........GG....................................G............G........
2 Sus barbatus barbatus          ....G.....G..................C.....T.G.............T.........G........
7 Sus barbatus oi                ....G................................G.................G.....GC.......
5 Sus barbatus oi                ....G..A..G..............A.......A...G................TG.....G........
10 Sus barbatus oi               ....G..A..G..............A.......A...G................TG.....G........
1 Sus verrucosus                 ....G...A.G..........................G.......................G........
3 Sus verrucosus                 ....G.G...G..........................G.......................G........
4 Sus verrucosus                 ....G.G...G..........................G.......................G........
10 Sus verrucosus                ....G.G...G...................................................G........
1 Sus celebensis                 ....G.....G..........................G...........A....TG..A..G........
5 Sus celebensis                 ....G.....G.......G..A...............G.....A..................G........
7 Sus celebensis                 ....G..A..G..............A.......A...G................TG.....G........
8 Potamochoerus larvatus         ....G.....G.......................C..G.......T................G........
10 Potamochoerus larvatus        ....G.G...G.................G........G....T...................G........
2 Potamochoerus porcus           ....G.G...G..........................G.................G.....G........
4 Potamochoerus porcus           .............A.......................G.................G.....G........
6 Potamochoerus porcus           ....G..A..G..........................G................TG.....G........
1 Hylochoerus meinertzhageni     ....G.....G..........................G.................G.....G........
4 Hylochoerus meinertzhageni     ....G.....G..........................G.................G.....G........
5 Hylochoerus meinertzhageni     ....G.G...G..........................G..........C.............G........
3 Phacochoerus africanus         ....G.....G..........................G.......................G........
4 Phacochoerus africanus         .....................................G.................G.....G........
5 Phacochoerus africanus         .....................................G.................G.....G........
7 Phacochoerus africanus         ....G.....G..........................G.......................G........
4 Phacochoerus aethiopicus       ....G..A..G..........................G.......................GC.......
6 Phacochoerus aethiopicus       ....G.....G..........................G.....T.................G........
7 Phacochoerus aethiopicus       ....G.....G..........................G.......................G......C.
1 2 Babyrousa babyrussa          ....G..T..G..........................G.........CT............G........
2 2 Babyrousa babyrussa          ..........G...................A......G................TG.....G........
3 2 Babyrousa babyrussa          ....G.....G..........................G.........CT............G........
4 2 Babyrousa babyrussa          ..........G...................A......G................TG.....G........
1 24 Babyrousa babyrussa         ....G.....G..........................G........T..............G........
3 24 Babyrousa babyrussa         ..A.......G..........................G.................G.....G........
4 24 Babyrousa babyrussa         ..........G..........................G.................G...A.G........
5 24 Babyrousa babyrussa         ..........G.........G................G...........A..T..G.....G........
6 24 Babyrousa babyrussa         ....G.....G..................................T..G......G.....G........
7 24 Babyrousa babyrussa         ....G.....G..........................G.......................G........
9 24 Babyrousa babyrussa         ..........G...................A......G................TG.....G........

                                          430       440       450       460       470       480       490
                                 ....|....|....|....|....|....|....|....|....|....|....|....|....|....|
Seq1 Ssrcofa8 chromosome1        CTCAAAACTGCTATTATTGGAAAAAATTGTCTTACGAGTTGGGGAGGATGGTTTAATGTTTCAGTTGAAA
Seq2 Ssrcofa8 chromosome2        ....G...............G.G..........G....GC........CA..........G.....A...
Seq3 Ssrcofa8 chromosome2        .........C.......A..G............G....GC........C..................GG.
```

Seq4 Ssrcofa8 chromosome6	....G............A..G.G..........G....GC........C..................G..
Seq5 Ssrcofa8 chromosome7	....G............A..G...........GG....GC..............................
Seq6 Ssrcofa8 chromosome9	....G........AC..A..G............G....GCA..........................G..
Seq7 Ssrcofa8 chromosomeX	....G...............G.G..........G....GC........C..................G..
Seq8 Ssrcofa8 chromosomeX	..............C.....G............G....G.........C...C.............A...
Sus scrofa(AF356698)	...........C.....A..G............G....-C........CA....................
3 *Sus scrofa*	....G............A..G.G..........G....GC........C...............A..G..
7 *Sus scrofa*	....G.......G....A..G.G...CCA....GT...G..............C..A........C.G..
1 *Sus barbatus barbatus*	....G............A..G...........GG....GC..............................
2 *Sus barbatus barbatus*	....G............A..G.G...C.....GG....GCA.......C..................G..
7 *Sus barbatus oi*	..................A.G.T..........GT...G............................G..
5 *Sus barbatus oi*	.................A..G............G....G.........C..................G..
10 *Sus barbatus oi*	.................A..G............G....GC...........................G..
1 *Sus verrucosus*	....G.........C..A..G............G....GC...........................G..
3 *Sus verrucosus*	.................A..G.G..........GT...GC........C...C.........G..A.G..
4 *Sus verrucosus*	....G............A..G.G..........G....GCA.......C..................G..
10 *Sus verrucosus*	....G............A..G.G..........G....G.........CA...........G.....G..
1 *Sus celebensis*	.........A.........AG............GT...G...........................A...
5 *Sus celebensis*	....G...............G.G..........GA...GCA..........................G..
7 *Sus celebensis*	.................A..G............G....GC........C..................G..
8 *Potamochoerus larvatus*	....G.........G..A..G.G..........G....GCA.......C.................A...
10 *Potamochoerus larvatus*	....G............A..G.G..........G....GC...A.......................G..
2 *Potamochoerus porcus*	....G...............G.G..........G....GC...A.......................G..
4 *Potamochoerus porcus*	....................G.G..........G....GC...........................G..
6 *Potamochoerus porcus*	.................A..G............G....GC......A.......................
1 *Hylochoerus meinertzhageni*	.................A..G........C...GT...GC...........................G..
4 *Hylochoerus meinertzhageni*	.................A..G........C...GT...GC...........................G..
5 *Hylochoerus meinertzhageni*	....G............A..G.G..........G....GC........C..................G..
3 *Phacochoerus africanus*	....G.G..A.......A..G.G..........GT...GC........C.............G..A.G..
4 *Phacochoerus africanus*	......................G..........G....GC...........................G..
5 *Phacochoerus africanus*	......................G..........G....GC...........................G..
7 *Phacochoerus africanus*	.C..G............A..G.G..........G....GCA.......C................A.G..
4 *Phacochoerus aethiopicus*	....G............A..G............G.A..G.........C..........G.......G..
6 *Phacochoerus aethiopicus*	....G............A..G.G..........G....GCA.......CA...............A.G..
7 *Phacochoerus aethiopicus*	....G.........C..A..G............G....GCA.......C..................G..
1 2 *Babyrousa babyrussa*	....G............A..G.G..........G.A..GC........C..................G..
2 2 *Babyrousa babyrussa*	.C..............CA..G.G..........GT...GC......................GG....G..
3 2 *Babyrousa babyrussa*	....G............A..G.G..........G.A..GC........C..................G..
4 2 *Babyrousa babyrussa*	.C..............CA..G.G..........GT...GC......................GG....G..
1 24 *Babyrousa babyrussa*	....G...............G.G..........G....GC.......................A...G..
3 24 *Babyrousa babyrussa*	....G............A..G.G..........GT...G.........CA.................G..
4 24 *Babyrousa babyrussa*	....G..A......C..A..G.G..........G....GCA.......C.....G............G..
5 24 *Babyrousa babyrussa*	.................A..G..G.........GT...GC........C..................G..
6 24 *Babyrousa babyrussa*	...................AG.G..........G....G............................G..
7 24 *Babyrousa babyrussa*	....G............A..G.G..........G....G.....G...C..................G..
9 24 *Babyrousa babyrussa*	.C..............CA..G.G..........GT...GC......................GG....G..

```
                                       500       510       520       530       540       550       560
                               ....|....|....|....|....|....|....|....|....|....|....|....|....|....|
Seq1 Ssrcofa8 chromosome1      GCCTAACATGTTT---AGGCCTAAGATATTATAATCTGACTGCCC-AAAAGACTCAATGGTGGTCATCCT
Seq2 Ssrcofa8 chromosome2      .............---.........G...................-..G...................T.
Seq3 Ssrcofa8 chromosome2      .............---..A......G...................-........................
Seq4 Ssrcofa8 chromosome6      ..........A..ATA.....G...G......G............-........................
Seq5 Ssrcofa8 chromosome7      .............---....G....G...................-.................C......
Seq6 Ssrcofa8 chromosome9      .............---......G..G...................-C...T.............T.....
Seq7 Ssrcofa8 chromosomeX      .............---.........G...................-........................
Seq8 Ssrcofa8 chromosomeX      .............---.........G.G.........A.......-........................
Sus scrofa(AF356698)           ..-..........---.........G...................-........................
3 Sus scrofa                   ..........A..ATA.....G...G......G............-........................
7 Sus scrofa                   ............----.....G...G.T.................-..........G...........-.
1 Sus barbatus barbatus        .............---....G....G...................-.................C......
2 Sus barbatus barbatus        .............---.........G...................-............A...........
7 Sus barbatus oi              .............---.........G.....A..C..........-T............T..........
5 Sus barbatus oi              .............---..A......G...................-........................
10 Sus barbatus oi             .............---..A......G...................-........................
1 Sus verrucosus               .............---......G..G...................-..G...................TG
3 Sus verrucosus               .........A...---.C.......G...................-........................
4 Sus verrucosus               ..T..........---......G..G......G............-........................
10 Sus verrucosus              .............---.........G...................-........................
1 Sus celebensis               .............---.........G...........A.......-T...T-..................
5 Sus celebensis               .............---.........G........C..........-........................
7 Sus celebensis               .............---..A......G...................-........................
8 Potamochoerus larvatus       .............---.........G...................-........................
10 Potamochoerus larvatus      ..T..........---.........G...................-.................C......
2 Potamochoerus porcus         ..T..........---.........G...................-.................C......
4 Potamochoerus porcus         A............---.........G...................-........................
6 Potamochoerus porcus         ....G........---.........G...................A........................
1 Hylochoerus meinertzhageni   .....G.......---.C.......G.G.................-.............T..........
4 Hylochoerus meinertzhageni   .....G.......---.C.......G.G.................-.............T..........
5 Hylochoerus meinertzhageni   ..T..........---......G..G--.................-........................
3 Phacochoerus africanus       ..T..........---........AG...................-........................
4 Phacochoerus africanus       A.T..........---.........G................T..-........................
5 Phacochoerus africanus       A.T..........---.........G................T..-........................
7 Phacochoerus africanus       ..T..........---......G...........G..........-........................
4 Phacochoerus aethiopicus     .............---...T.....G.....C........G....-........................
6 Phacochoerus aethiopicus     ..T..........---......G..G........G..........-........................
7 Phacochoerus aethiopicus     .............---.........G...............T...-....T.............T.....
1 2 Babyrousa babyrussa        .............---.........G...............T...-........................
2 2 Babyrousa babyrussa        ..T..........---.........G...................-........................
3 2 Babyrousa babyrussa        .............---.........G...............T...-........................
4 2 Babyrousa babyrussa        ..T..........---.........G...................-........................
1 24 Babyrousa babyrussa       ...........C.---.........G...................-........................
```

```
3 24 Babyrousa babyrussa        .............---.........GA.................-........................
4 24 Babyrousa babyrussa        ..T..........---.........G..................-........................
5 24 Babyrousa babyrussa        .............---.........G..................-........................
6 24 Babyrousa babyrussa        .............---.........G..................-......T..G..............
7 24 Babyrousa babyrussa        .............---.........G..................-T...............A.......
9 24 Babyrousa babyrussa        ..T..........---.........G..................-........................

                                        570       580       590       600       610       620       630
                                ....|....|....|....|....|....|....|....|....|....|....|....|....|....|
Seq1 Ssrcofa8 chromosome1       CCAATCTCTCAGAGCCCAAACCTAATCCCTTGTCTAACTTTTCCCTCCTTTGACAAGCATGGGAAGATAT
Seq2 Ssrcofa8 chromosome2       ......A............G..............................C...................
Seq3 Ssrcofa8 chromosome2       .....TG............G..............................C...................
Seq4 Ssrcofa8 chromosome6       .T....G............G.........................A....C...................
Seq5 Ssrcofa8 chromosome7       ...................G.........----............A...CC..TG.............--
Seq6 Ssrcofa8 chromosome9       ....C..............G.........C....C..T.......A...C.....G...........C.C
Seq7 Ssrcofa8 chromosomeX       ..............................................................A.......
Seq8 Ssrcofa8 chromosomeX       ........................................................A....AA.......
Sus scrofa(AF356698)            T...............G..G..............................C...........A.......
3 Sus scrofa                    .T....G............G.........................A....C...................
7 Sus scrofa                    ...GC.............GG.........----............A...CC....G........G.....
1 Sus barbatus barbatus         ...................G.........----............A...CC..TG...............
2 Sus barbatus barbatus         ......A............G............---------....A....C...................
7 Sus barbatus oi               .....................A......................T........A.G......A..C....
5 Sus barbatus oi               .....TG............G..............................C...................
10 Sus barbatus oi              .....TG............G..............................C...................
1 Sus verrucosus                ..T.C..............G.........C....C..T.......A...C.....G...........C.C
3 Sus verrucosus                ......G............G.T...........................................AG...
4 Sus verrucosus                ......G............G..C......................A....C...................
10 Sus verrucosus               ......G....A.......G....................C.........C...................
1 Sus celebensis                ..........................................T.......C...................
5 Sus celebensis                .....TG............G.........................A...A....................
7 Sus celebensis                .....TG............G..............................C...................
8 Potamochoerus larvatus        ......G............G.........................A....CA....A.......T.....
10 Potamochoerus larvatus       ...................G......T..----............A...CC..AG........G......
2 Potamochoerus porcus          .................G.G......T..----............A...CC..AG........G......
4 Potamochoerus porcus          ......G............G............C............A....C...................
6 Potamochoerus porcus          ..............T....G..........................................A.......
1 Hylochoerus meinertzhageni    ...G..A.........T..G..............................C................G..
4 Hylochoerus meinertzhageni    ...G..A.........T..G..............................C................G..
5 Hylochoerus meinertzhageni    .....TG............G.................A.......A....C...................
3 Phacochoerus africanus        ......G............G.........................A..........A.............
4 Phacochoerus africanus        ......A......................................A....C...................
5 Phacochoerus africanus        ......A......................................A....C...................
7 Phacochoerus africanus        ......G............G.........................A....C...................
4 Phacochoerus aethiopicus      ...................G............C.................CA..................
6 Phacochoerus aethiopicus      .G....G............G.........................A....C...................
```

```
7 Phacochoerus aethiopicus       ....C..............G.........C....C..T.......A...C.....G...........C.C
1 2 Babyrousa babyrussa          ....C..............G.........-....C..T.......AT..C.....G..............
2 2 Babyrousa babyrussa          ......G......T..T..G.........................A....C...........A.T....C
3 2 Babyrousa babyrussa          ....C..............G.........-....C..T.......AT..C.....G..............
4 2 Babyrousa babyrussa          ......G......T..T..G.........................A....C...........A.T....C
1 24 Babyrousa babyrussa         A.....G............G.........................A....CA........A.A.......
3 24 Babyrousa babyrussa         ..................................................G...................
4 24 Babyrousa babyrussa         ......G............G...............C.........A....C.....A.............
5 24 Babyrousa babyrussa         ...................GT........................A....C...................
6 24 Babyrousa babyrussa         ......G.......G....G......A.................A....C...................
7 24 Babyrousa babyrussa         .............................................A....C...................
9 24 Babyrousa babyrussa         ......G.A....T..T..G.........................A....C...........A.T....C

                                        640       650       660       670       680       690       700
                                 ....|....|....|....|....|....|....|....|....|....|....|....|....|....|
Seq1 Ssrcofa8 chromosome1        ------CAGTGCTGATATCCAGTGGCGGGCACCAAAGGGA-TTATATTGGATTTGTAAA--AAAAATGGC
Seq2 Ssrcofa8 chromosome2        ------....A..A...........T..............-...............GGG--.........
Seq3 Ssrcofa8 chromosome2        ------....A..A...........T..............-.......AT......-GG--G........
Seq4 Ssrcofa8 chromosome6        ------....A..A............A.C...........-...............-GG--.........
Seq5 Ssrcofa8 chromosome7        -------......A............A.............-...............-G.--.........
Seq6 Ssrcofa8 chromosome9        CTCTGA.......A.......A..................-.......A.......-G.--.......T.
Seq7 Ssrcofa8 chromosomeX        ------..C.A..A...........T..............-...............-G.--.........
Seq8 Ssrcofa8 chromosomeX        ------..................................-...............-G.--.........
Sus scrofa(AF356698)             -------------A............A.............-G.......A......-GG--.........
3 Sus scrofa                     ------....A..A............A.C...........-...............-GG--.........
7 Sus scrofa                     ------T..............A............G.....-...............-GG--.........
1 Sus barbatus barbatus          -------......A............A.............-...............-G.--.........
2 Sus barbatus barbatus          ------....A..A..........................-...............-GG--.........
7 Sus barbatus oi                ------....A..A...C........A.............-...............-..--.........
5 Sus barbatus oi                ------....A..A...........T............A.-....G..AT......-GG--G........
10 Sus barbatus oi               ------....A..A...........T............A.-.......AT......-GG--G........
1 Sus verrucosus                 CTCTGA.......A.......A...T..............-.......A.......-G.--.......T.
3 Sus verrucosus                 ------....A..AC.........................-...............-..--.........
4 Sus verrucosus                 -------......A.........A....C...........-...............-GG--.........
10 Sus verrucosus                ------....A..A.......T..................-...............-GGAA.........
1 Sus celebensis                 ------...................T..............-...............-..--........T
5 Sus celebensis                 ------....A..A..........................-...............-GG--.........
7 Sus celebensis                 ------....A..A...........T............A.-.......AT......-GG--G........
8 Potamochoerus larvatus         ------....A..A...........T..............-...............-GG--.........
10 Potamochoerus larvatus        -------......A...C......................-...............-GG--G........
2 Potamochoerus porcus           -------......A..........................-...............-GG--G........
4 Potamochoerus porcus           ------....A..A..........................-.....C...G.....-G.--.G.......
6 Potamochoerus porcus           ------....A..A..........................-...............-GG--G........
1 Hylochoerus meinertzhageni     ------....A..A....G...C.................-...............GGG--.........
4 Hylochoerus meinertzhageni     ------....A..A....G...C.................-...............GGG--.........
5 Hylochoerus meinertzhageni     -------......A..........................-.....C.........-GG--....G....
```

```
3 Phacochoerus africanus       ------....A..A..........................-...............-..--.........
4 Phacochoerus africanus       ------....A..A............A.A...........-....G..........-G.--.........
5 Phacochoerus africanus       ------....A..A............A.A...........-....G..........-G.--.........
7 Phacochoerus africanus       ------....A..A..........................-...............-GG--.........
4 Phacochoerus aethiopicus     ------.......A............A.............-...............-G.--.........
6 Phacochoerus aethiopicus     ------....A..A.....................C....-...............-GG--.........
7 Phacochoerus aethiopicus     CTCTGA.......A.......A..................-...............-GG--.......T.
1 2 Babyrousa babyrussa        ------.......A.......A..................-...............-GG--.........
2 2 Babyrousa babyrussa        ------....A..A..........................T...............-GG--.........
3 2 Babyrousa babyrussa        ------.......A.......A..................-...............-GG--.........
4 2 Babyrousa babyrussa        ------....A..A..........................-...............GG.--.........
1 24 Babyrousa babyrussa       ------..A....A............A.............-...............-G.--.........
3 24 Babyrousa babyrussa       ------.......A..........T.A.............-...C...........-..--.........
4 24 Babyrousa babyrussa       ------....A..A....G.......A.............-...............-GG--.........
5 24 Babyrousa babyrussa       ------....A..A..........................-............C..-G.--.........
6 24 Babyrousa babyrussa       ------....A..A............A.............-...............-G.--.........
7 24 Babyrousa babyrussa       ------....A..A..........................-...............-GG--.........
9 24 Babyrousa babyrussa       ------....A..A..........................-...............GG.--.........

                                       710       720       730       740       750       760       770
                               ....|....|....|....|....|....|....|....|....|....|....|....|....|....|
Seq1 Ssrcofa8 chromosome1      CTCTTCCATACT--CCCCCC----CAAATTGGGCTGGGACATGT-GTACTGGGAACAATCAGACCTTCCT
Seq2 Ssrcofa8 chromosome2      ..A.........--......----..............G.....-.........................
Seq3 Ssrcofa8 chromosome2      ..A.........---.....----..C...........G.....-..............G..........
Seq4 Ssrcofa8 chromosome6      ..A.........---.....----...C..........G.....-.........................
Seq5 Ssrcofa8 chromosome7      ..A....T....---.....----TG............G.....-.........................
Seq6 Ssrcofa8 chromosome9      ..A.........--......----.C............G...A.G..........A...T..........
Seq7 Ssrcofa8 chromosomeX      ..A.........---.....----....................-....................C....
Seq8 Ssrcofa8 chromosomeX      ..A.........---.T...----.C............G..C..-.........................
Sus scrofa(AF356698)           ..A.........---.....----..............G.....-.........................
3 Sus scrofa                   ..A.........---.....----...C..........G.....-.........................
7 Sus scrofa                   ..A.........----....----.C.T......C...G.....-....................A....
1 Sus barbatus barbatus        ..A..T.T....---.....----TG............G.....-.........................
2 Sus barbatus barbatus        ..A.........--......----..............G.....-.........................
7 Sus barbatus oi              ..A.........CC......----...G..........G.....-.........................
5 Sus barbatus oi              ..A.........---.....----..............G.....-.........................
10 Sus barbatus oi             ..A.........---.....----..............G.....-.........................
1 Sus verrucosus               ..A.........---.....----.C............G...A.G.........................
3 Sus verrucosus               ..A.........--......----...G..........G.....-.........................
4 Sus verrucosus               ..A.........---.....----....................-.........................
10 Sus verrucosus              ..A.........--....T.----........A.....G.....-.........................
1 Sus celebensis               ..A.........--......----....................-.........................
5 Sus celebensis               ..A.........--......----...........A..G...A.-.........................
7 Sus celebensis               ..A.........---.....----..............G.....-.........................
8 Potamochoerus larvatus       ..A.........CC......----..............G.....-.........................
10 Potamochoerus larvatus      ..A.........---.....----....................-.........................
```

```
2 Potamochoerus porcus          ..A.........---.....----....................-.........................
4 Potamochoerus porcus          ..A.........---.....----..............G.....-.........................
6 Potamochoerus porcus          ..A.........---.....----....................-.........................
1 Hylochoerus meinertzhageni    ..A.........---.....----..............G.....-.........................
4 Hylochoerus meinertzhageni    ..A.........---.....----..............G.....-.........................
5 Hylochoerus meinertzhageni    ..A.........---.....----....................-................T........
3 Phacochoerus africanus        ..A.........---..T..----....................-.........................
4 Phacochoerus africanus        ..A.........---.....----..............G.....-..........A..............
5 Phacochoerus africanus        ..A.........---.....----..............G.....-.........................
7 Phacochoerus africanus        ..A.........---.....----A.............G.....-.C.......................
4 Phacochoerus aethiopicus      ..A.........--......----.C............G...A.-.......A.........G.......
6 Phacochoerus aethiopicus      ..A.........---.....----A.............G.....-.........................
7 Phacochoerus aethiopicus      ..A.........---.....----.C............G...A.-......................T..
1 2 Babyrousa babyrussa         ..A......G..--......CCCT.C............G.....-.........................
2 2 Babyrousa babyrussa         ..A.........--......----..............G.....-T.T..................C...
3 2 Babyrousa babyrussa         ..A......G..--......CCCT.C............G.....-.........................
4 2 Babyrousa babyrussa         ..A.........--......----..............G.....-T.T..................C...
1 24 Babyrousa babyrussa        ..A......G..---.....CCCC..............G.....-.........................
3 24 Babyrousa babyrussa        ..A.........---.....----..............G.....-....................C....
4 24 Babyrousa babyrussa        ..A.........---.T...----..............G.....-........................C
5 24 Babyrousa babyrussa        ..A-........---.....----.-..................-A......A.................
6 24 Babyrousa babyrussa        ..A.........---.....----....................-A.........AC.............
7 24 Babyrousa babyrussa        ..A.........---.....----..............G.....-.........................
9 24 Babyrousa babyrussa        ..A.........--......----..............G.....-T.T..................C...

                                         780       790       800       810       820       830       840
                                ....|....|....|....|....|....|....|....|....|....|....|....|....|....|
Seq1 Ssrcofa8 chromosome1       TCTTCCTGCTCCTACTGTCACTATGGGAGCAACTGGGAATTCCTATATATAGGATACAGGGTGCCAAAAG
Seq2 Ssrcofa8 chromosome2       ............CG.........G..............G..........C...GC..........C.G..
Seq3 Ssrcofa8 chromosome2       ..........T.C..........G..........A...G.......G......GC..........C.G..
Seq4 Ssrcofa8 chromosome6       ............C..........G.A............G..............GC..........C.G..
Seq5 Ssrcofa8 chromosome7       ............C..........G.........A....G..............GC..........C.G..
Seq6 Ssrcofa8 chromosome9       ............CG.........G..............G..........C...GC..........CTG..
Seq7 Ssrcofa8 chromosomeX       ............C..........G....A........................GC..........C.G..
Seq8 Ssrcofa8 chromosomeX       ............CG.........G..............G..............GC..........C.G..
Sus scrofa(AF356698)            ............CG.........G.............................-C..........C.G..
3 Sus scrofa                    ............C..........G.A............G..............GC..........C.G..
7 Sus scrofa                    ............C.T........G..............G..............GC......C...C.G..
1 Sus barbatus barbatus         ............C..........G.........A....G..............GC..........C.G..
2 Sus barbatus barbatus         ............CG.........G..............G..............GC..........C.G..
7 Sus barbatus oi               .............G.........G..-...........G..............GC..........C.G..
5 Sus barbatus oi               ..........T.C..........G..........A...G.......G......GC..........C.G..
10 Sus barbatus oi              ..........T.C..........G..........A...G.......G......GC..........C.G..
1 Sus verrucosus                .T...T......CG.........G..............G..........C...GC..........C.G..
3 Sus verrucosus                .............G.........G..-...........G..............GC..........C.G..
4 Sus verrucosus                ............CG.........G..A...........G..............GC..........C.G..
```

```
10 Sus verrucosus                  .............G.........G..............G..........C...GC..........C.G..
1 Sus celebensis                   .............G.........................G...............................
5 Sus celebensis                   ............CG.........G..............G.........--...GC..........C.G..
7 Sus celebensis                   ..........T.C..........G..........A...G.......G.......GC..........C.G..
8 Potamochoerus larvatus           ............CG.........G..............G..............GC..........C.G..
10 Potamochoerus larvatus          ............CG.........G..A.A.........G..........C...GC..........C.G..
2 Potamochoerus porcus             ............CG.........G..A.A.........G..........C...GC..........C.G..
4 Potamochoerus porcus             ............CG.........G..............G..............GC..........C.G..
6 Potamochoerus porcus             ............CG.........G..A.A.........G..........C...GC..........C.G..
1 Hylochoerus meinertzhageni       ............CG.........G..............G..........C...GC..........C.G..
4 Hylochoerus meinertzhageni       ............CG.........G..............G..........C...GC..........C.G..
5 Hylochoerus meinertzhageni       ............CG.........G..A...........G.......G.......GC..........C.G..
3 Phacochoerus africanus           ............CG.....G...G..............G..............GC..........C.G..
4 Phacochoerus africanus           .............G.....T...GA.............G..............GC..........C.G..
5 Phacochoerus africanus           .............G.....T...GA.............G..............GC..........C.G..
7 Phacochoerus africanus           ............C..........G..............G..............GC........T.C.G..
4 Phacochoerus aethiopicus         .....G......C...A......G..A.A........CG..............GC..........C.G..
6 Phacochoerus aethiopicus         ............C..........G..............G..............GC........T.C.G..
7 Phacochoerus aethiopicus         ............CG.........G..............G..........C...GC..........C.G..
1 2 Babyrousa babyrussa            ............CG.........G..............G..........C...GC..........C.G..
2 2 Babyrousa babyrussa            .............G.........G.....A........G..........C...GC.......T..C.G..
3 2 Babyrousa babyrussa            ............CG.........G..............G..........C...GC..........C.G..
4 2 Babyrousa babyrussa            .............G.........G.....A........G..........C...GC.......T..C.G..
1 24 Babyrousa babyrussa           ............CG.........G..............G..............GC..........C.G..
3 24 Babyrousa babyrussa           ............CG.........G....A.........G..............GC......CA..C.G..
4 24 Babyrousa babyrussa           ............C..........G.A............G..............GC..........C.G..
5 24 Babyrousa babyrussa           .A..........C..........G.A............G..............GC..........C.G..
6 24 Babyrousa babyrussa           ............CG.........G..............G..............GC..........C.G..
7 24 Babyrousa babyrussa           ............CG.........G..............G..............GC..........C.G..
9 24 Babyrousa babyrussa           .............G.........G.....A........G..........C...GC.......T..C.G..

                                           850       860       870       880       890       900       910
                                   ....|....|....|....|....|....|....|....|....|....|....|....|....|....|
Seq1 Ssrcofa8 chromosome1          AGACCAGAGGGCTCTCCAAATTGGAGTCTGGGAAGATGATGCCCGGCCCCCAGAATGGATAATTCAATAT
Seq2 Ssrcofa8 chromosome2          .....G....................A....A.........AAT...........C..............
Seq3 Ssrcofa8 chromosome2          .....G....................A...AA.........AAT...........C..............
Seq4 Ssrcofa8 chromosome6          .....G....................AG...A........AAAT...........C..............
Seq5 Ssrcofa8 chromosome7          .A..TG....................A....A........AAAT...........CA.............
Seq6 Ssrcofa8 chromosome9          ..........................A....A.........AAT...........A.A............
Seq7 Ssrcofa8 chromosomeX          .....G....................A....A.........AAT...........C..............
Seq8 Ssrcofa8 chromosomeX          .....G....A...............A....A.......T.AAT...........CA.............
Sus scrofa(AF356698)               .....G....................A....A.........AAT.........G................
3 Sus scrofa                       .A..TG....................A....A........AAAT...........CA.............
7 Sus scrofa                       ....TGA...........G.......A....A.........AAT..............---.........
1 Sus barbatus barbatus            .A..TG..A.............T...A....A........AAAT...........CA.............
2 Sus barbatus barbatus            ..........A...............A...AA..A......GAT..........................
```

```
7 Sus barbatus oi                .....G....C...........A...A....A........AAAT..........................
5 Sus barbatus oi                .....G....................A...AA.........AAT...........C..............
10 Sus barbatus oi               .....G....................A...AA.........AAT...........C..............
1 Sus verrucosus                 .....G...................AA....A.........AAT...........C..............
3 Sus verrucosus                 .....G....C...........A...A....A........AAAT...........C..............
4 Sus verrucosus                 .....G......C..T..........A.............AAAT...T....A..C..............
10 Sus verrucosus                .....G.......G............A....A.........AAT...........A.A............
1 Sus celebensis                 .....G....................A...........................................
5 Sus celebensis                 ....TG....................A....A........AAAT..........................
7 Sus celebensis                 .....G....................A...AA.........AAT...........C..............
8 Potamochoerus larvatus         .....GA.......C.......C...A....A.........AAT..........................
10 Potamochoerus larvatus        .....G....................A...........G.AAAT.......T...C..............
2 Potamochoerus porcus           .....G....................A...........G.AAAT...........C..............
4 Potamochoerus porcus           ..........................A....A.........AAT..........................
6 Potamochoerus porcus           .....G....................A...........G.AAAT...........C..............
1 Hylochoerus meinertzhageni     .....G....................A...TA.........AAT....T......C............G.
4 Hylochoerus meinertzhageni     .....G....................A....A.........AAT....T......C............G.
5 Hylochoerus meinertzhageni     .....G......C.............A.............AAAT...T.......C..............
3 Phacochoerus africanus         .....G....C.........C.....A....A.........AAT..........................
4 Phacochoerus africanus         .....G....................A....A.........AAT..........................
5 Phacochoerus africanus         .....G....................A....A.........AAT..........................
7 Phacochoerus africanus         ...–TG.G..................A....A........AAAT..........................
4 Phacochoerus aethiopicus       .........................AAA...A........AAAT...........CA.............
6 Phacochoerus aethiopicus       ...................G......C....A........AAAT.....................T....
7 Phacochoerus aethiopicus       ..........................A....A.........AAT...........A.A............
1 2 Babyrousa babyrussa          ..........................A....A.........AAT...........A.A..........G.
2 2 Babyrousa babyrussa          ....TG.....T..............A....A.........AAT...........CA.............
3 2 Babyrousa babyrussa          ..................G.......A....A.........AAT...........A.A............
4 2 Babyrousa babyrussa          ....TG.....T..............A....A.........AAT...........CA.............
1 24 Babyrousa babyrussa         ..........................A....A.T.......AAT...........C..............
3 24 Babyrousa babyrussa         ....TG....................A....A..A......AAT...........C..............
4 24 Babyrousa babyrussa         ....TG....................A....A.........AAT..........................
5 24 Babyrousa babyrussa         .....G....................A....A.........AAT..........................
6 24 Babyrousa babyrussa         .....G....................A....A.....A..AAAT..........................
7 24 Babyrousa babyrussa         .....G....................A....A.........AAT........A..C..............
9 24 Babyrousa babyrussa         ....TG.....T..............A....A.........AAT...........CA.............

                                          920       930       940       950       960       970       980
                                 ....|....|....|....|....|....|....|....|....|....|....|....|....|....|
Seq1 Ssrcofa8 chromosome1        TATGGACCTGCCACCTGGACAAAGGATGGCTCTCGGGGATATCGCACCCCTATC----------------
Seq2 Ssrcofa8 chromosome2        ..................G..G...........T....................----------------
Seq3 Ssrcofa8 chromosome2        ..................G..G...........T.........A..........----------------
Seq4 Ssrcofa8 chromosome6        ....A.............G..G...........T....................----------------
Seq5 Ssrcofa8 chromosome7        ..................G..G...........T.A.......A..........----------------
Seq6 Ssrcofa8 chromosome9        .....G............G..G...........T....................----------------
Seq7 Ssrcofa8 chromosomeX        .....G............G..G.........T.T.........A.......G..----------------
```

```
Seq8 Ssrcofa8 chromosomeX        .....G............G..G...........T............T.......----------------
Sus scrofa(AF356698)             .....G............G..G..........-----------A..........----------------
3 Sus scrofa                     ..................G..G...........T.A.......A..........----------------
7 Sus scrofa                     .....G............G..G...........T.........A..........----------------
1 Sus barbatus barbatus          ..................G..G...........T.A.......A..........----------------
2 Sus barbatus barbatus          ..................G..G....G......T.........A.......G..----------------
7 Sus barbatus oi                ..................G..G...........T..T......A.......G..----------------
5 Sus barbatus oi                ..................G..G...........T....................----------------
10 Sus barbatus oi               ..................G..G...........T....................----------------
1 Sus verrucosus                 .....G............G..G...........T...T....T...........----------------
3 Sus verrucosus                 ..................G..G...........T..T......A.......G..----------------
4 Sus verrucosus                 .....G..C.........G..G...........T....................----------------
10 Sus verrucosus                .....G............G..G...........T....................----------------
1 Sus celebensis                 .................................T....................----------------
5 Sus celebensis                 ..................G..G...........T.........T..........----------------
7 Sus celebensis                 ..................G..G...........T.........A..........----------------
8 Potamochoerus larvatus         ..................G..............T.........A..........----------------
10 Potamochoerus larvatus        .....G............G..G...........T....................----------------
2 Potamochoerus porcus           .....G.........C..G..G...........T....................----------------
4 Potamochoerus porcus           .....G............G..G...........T.................G..----------------
6 Potamochoerus porcus           .....G.........C..G..G...........T....................----------------
1 Hylochoerus meinertzhageni     ..................G..G...........T..................C.----------------
4 Hylochoerus meinertzhageni     ..................G..G...........T..................C.----------------
5 Hylochoerus meinertzhageni     ..................G..G...........T.........A..........----------------
3 Phacochoerus africanus         ....C........A..A.G..G...........T....................----------------
4 Phacochoerus africanus         .....G............G..G...........T........T........G..----------------
5 Phacochoerus africanus         .....G............G..G...........T........T........G..----------------
7 Phacochoerus africanus         ..................G..G.........T.T........T...........----------------
4 Phacochoerus aethiopicus       .....G............G..G...........T....................----------------
6 Phacochoerus aethiopicus       ..................G..G...........T........T...........----------------
7 Phacochoerus aethiopicus       .....G............G..G...........T....................----------------
1 2 Babyrousa babyrussa          .....G............G..G...........T.A.......A..........----------------
2 2 Babyrousa babyrussa          .....G.A..........G..G...........T...A.....A..........----------------
3 2 Babyrousa babyrussa          .....G............G..G...........T.A.......A..........----------------
4 2 Babyrousa babyrussa          .....G.A..........G..G...........T...A.....A..........----------------
1 24 Babyrousa babyrussa         ..............A...G..G...........T.................G..----------------
3 24 Babyrousa babyrussa         ..............A...G..G...........T.........A.......G..----------------
4 24 Babyrousa babyrussa         .....G............GT.G...........T.........A.......G..----------------
5 24 Babyrousa babyrussa         ..................G..G...........T....................----------------
6 24 Babyrousa babyrussa         ..................G..G...........T...A.............G..TATATATACTGAACCG
7 24 Babyrousa babyrussa         .....G............G..G...........T.......C............----------------
9 24 Babyrousa babyrussa         .....G.A..........G..G...........T...A.....A..........----------------

                                          990       1000      1010      1020      1030      1040      1050
                                 ....|....|....|....|....|....|....|....|....|....|....|....|....|....|

Seq1 Ssrcofa8 chromosome1        ----------------------------------------------------------------------
```

Seq2 Ssrcofa8 chromosome2	--
Seq3 Ssrcofa8 chromosome2	--
Seq4 Ssrcofa8 chromosome6	--
Seq5 Ssrcofa8 chromosome7	--
Seq6 Ssrcofa8 chromosome9	--
Seq7 Ssrcofa8 chromosomeX	--
Seq8 Ssrcofa8 chromosomeX	--
Sus scrofa(AF356698)	--
3 *Sus scrofa*	--
7 *Sus scrofa*	--
1 *Sus barbatus barbatus*	--
2 *Sus barbatus barbatus*	--
7 *Sus barbatus oi*	--
5 *Sus barbatus oi*	--
10 *Sus barbatus oi*	--
1 *Sus verrucosus*	--
3 *Sus verrucosus*	--
4 *Sus verrucosus*	--
10 *Sus verrucosus*	--
1 *Sus celebensis*	--
5 *Sus celebensis*	--
7 *Sus celebensis*	--
8 *Potamochoerus larvatus*	--
10 *Potamochoerus larvatus*	--
2 *Potamochoerus porcus*	--
4 *Potamochoerus porcus*	--
6 *Potamochoerus porcus*	--
1 *Hylochoerus meinertzhageni*	--
4 *Hylochoerus meinertzhageni*	--
5 *Hylochoerus meinertzhageni*	--
3 *Phacochoerus africanus*	--
4 *Phacochoerus africanus*	--
5 *Phacochoerus africanus*	--
7 *Phacochoerus africanus*	--
4 *Phacochoerus aethiopicus*	--
6 *Phacochoerus aethiopicus*	--
7 *Phacochoerus aethiopicus*	--
1 2 *Babyrousa babyrussa*	--
2 2 *Babyrousa babyrussa*	--
3 2 *Babyrousa babyrussa*	--
4 2 *Babyrousa babyrussa*	--
1 24 *Babyrousa babyrussa*	--
3 24 *Babyrousa babyrussa*	--
4 24 *Babyrousa babyrussa*	--
5 24 *Babyrousa babyrussa*	--
6 24 *Babyrousa babyrussa*	**CATCATCAGACTACATGTGGTTTTGGAACTTATAACAAATGACACTGCAAGGGCCTTGACTATAGAGGAT**

```
7 24 Babyrousa babyrussa          ----------------------------------------------------------------------
9 24 Babyrousa babyrussa          ----------------------------------------------------------------------

                                          1060      1070      1080      1090      1100      1110      1120
                                  ....|....|....|....|....|....|....|....|....|....|....|....|....|....|
Seq1 Ssrcofa8 chromosome1         -----------------------------TATATGCTGAGCCACATCCTCAGACTACAGGCGGTTTTAGA
Seq2 Ssrcofa8 chromosome2         -----------------------------.C........A..G....A...................G..
Seq3 Ssrcofa8 chromosome2         -----------------------------..........A.......T............TA.....G..
Seq4 Ssrcofa8 chromosome6         -----------------------------..........A..G....A...................G..
Seq5 Ssrcofa8 chromosome7         -----------------------------..........A.......A...................G..
Seq6 Ssrcofa8 chromosome9         -----------------------------..........A..G....A...................G..
Seq7 Ssrcofa8 chromosomeX         -----------------------------..........A.......A...................G..
Seq8 Ssrcofa8 chromosomeX         -----------------------------........A.A..G....A...................G..
Sus scrofa(AF356698)              -----------------------------..........A.TG....A...................G..
3 Sus scrofa                      -----------------------------..........A.......A...................G..
7 Sus scrofa                      -----------------------------.C........A.......A............T......G..
1 Sus barbatus barbatus           -----------------------------..........A.......A...................G..
2 Sus barbatus barbatus           -----------------------------..........A..G....A...................G..
7 Sus barbatus oi                 -----------------------------..........A.......A...................G..
5 Sus barbatus oi                 -----------------------------..........A..G....A..G.........T......G..
10 Sus barbatus oi                -----------------------------..........A..G....A............T......G..
1 Sus verrucosus                  -----------------------------..........A.AG....A...................G..
3 Sus verrucosus                  -----------------------------..........A......TA...................G..
4 Sus verrucosus                  -----------------------------..........A.......A........T...T......G..
10 Sus verrucosus                 -----------------------------..........A..G....A...................G..
1 Sus celebensis                  -----------------------------...G..................................GA.
5 Sus celebensis                  -----------------------------..........A.TG....A...................G..
7 Sus celebensis                  -----------------------------....C.....A..G....A............T......G..
8 Potamochoerus larvatus          -----------------------------..........A..G....A...................GA.
10 Potamochoerus larvatus         -----------------------------..........A..G....A...................G..
2 Potamochoerus porcus            -----------------------------..........A..G....A...................G..
4 Potamochoerus porcus            -----------------------------..........A..G....A...A........T......G..
6 Potamochoerus porcus            -----------------------------..........A..G....A...................G..
1 Hylochoerus meinertzhageni      -----------------------------..........A..G....A............T......G..
4 Hylochoerus meinertzhageni      -----------------------------..........A..G....A............T......G..
5 Hylochoerus meinertzhageni      -----------------------------..........A..G....A..G................GA.
3 Phacochoerus africanus          -----------------------------..........A..G....A...........A.A.....GA.
4 Phacochoerus africanus          -----------------------------..........A..G....A...........A..C....G..
5 Phacochoerus africanus          -----------------------------..........A..G....A...........A..C....G..
7 Phacochoerus africanus          -----------------------------..........A..G....A.............A.....G..
4 Phacochoerus aethiopicus        -----------------------------..........A.TG....A...................G..
6 Phacochoerus aethiopicus        -----------------------------..........A..G....A.............A.....G..
7 Phacochoerus aethiopicus        -----------------------------...G......A.TG....A.............T.....G..
1 2 Babyrousa babyrussa           -----------------------------....C.....A.......A...................G..
2 2 Babyrousa babyrussa           -----------------------------..........A..G....A............AA.....G..
3 2 Babyrousa babyrussa           -----------------------------....C.....A.......A...................G..
```

```
4 2 Babyrousa babyrussa         -----------------------------..........A..G....A............AA.....G..
1 24 Babyrousa babyrussa        -----------------------------.G........A.......A......A............G..
3 24 Babyrousa babyrussa        -----------------------------..........A..G....A...................G..
4 24 Babyrousa babyrussa        -----------------------------..........A..G....A...................G..
5 24 Babyrousa babyrussa        -----------------------------..........A..G....A...................G..
6 24 Babyrousa babyrussa        GGCTCTTGGGAATATCGCACCCCTGTCTA.....A....A..G....A..........TAT......G..
7 24 Babyrousa babyrussa        -----------------------------..........A..G....A............T......G..
9 24 Babyrousa babyrussa        -----------------------------..........A..G....A............AA.....G..

                                        1130      1140      1150      1160      1170      1180      1190
                                ....|....|....|....|....|....|....|....|....|....|....|....|....|....|
Seq1 Ssrcofa8 chromosome1       ACTTATAACAAATGACACTACAAGGGCCTTGACTATATTGGCCCAACAACAAACTAAAATGCACAGTACA
Seq2 Ssrcofa8 chromosome2       .............A.....G.......G.........C...........A............G....G..
Seq3 Ssrcofa8 chromosome2       ...................GA..C..............................................
Seq4 Ssrcofa8 chromosome6       ...................G.........................................T.....G..
Seq5 Ssrcofa8 chromosome7       ...................G.......................................C..G....GA.
Seq6 Ssrcofa8 chromosome9       ...................G...............................................G..
Seq7 Ssrcofa8 chromosomeX       ...................G.............................T..........A......GA.
Seq8 Ssrcofa8 chromosomeX       ...................G...................................G...........G..
Sus scrofa(AF356698)            ...................G.........................................A........
3 Sus scrofa                    ...................G......-................................CA.G....GA.
7 Sus scrofa                    ....G..............G....................AA......G............TG..A.GTC
1 Sus barbatus barbatus         ...................G.......................................C.TG....GA.
2 Sus barbatus barbatus         ...................G...............................................G..
7 Sus barbatus oi               ............C..............T..................T.............A......G..
5 Sus barbatus oi               ...................GA..C..............................................
10 Sus barbatus oi              ...................GA..C..............................................
1 Sus verrucosus                ...................G...............................................G..
3 Sus verrucosus                ...........................T..................T.............A......G..
4 Sus verrucosus                ...................G...............................................G..
10 Sus verrucosus               ............C......G...............................................G..
1 Sus celebensis                .............A.....G..................................................
5 Sus celebensis                ............G......G...A......................................G....G..
7 Sus celebensis                ...................GA..C..............................................
8 Potamochoerus larvatus        ...................G........................................A.G....G..
10 Potamochoerus larvatus       ...................G...............................................G..
2 Potamochoerus porcus          ...................G........................................A......G..
4 Potamochoerus porcus          ......-............G........C......................................G..
6 Potamochoerus porcus          ...................G...............................................G..
1 Hylochoerus meinertzhageni    ...................G..........C........................-......G....G..
4 Hylochoerus meinertzhageni    ...................G..........C........................-......G....G..
5 Hylochoerus meinertzhageni    ...................G................G.........................G....G..
3 Phacochoerus africanus        ..........G.........................................G....C....G..A.G..
4 Phacochoerus africanus        ...................G..............................G................G..
5 Phacochoerus africanus        ...................G..............................G................G..
7 Phacochoerus africanus        ...................G.................G........A....................G..
```

```
4 Phacochoerus aethiopicus      ...........G................................................A......G..
6 Phacochoerus aethiopicus      ...................G.................G..A..........................G..
7 Phacochoerus aethiopicus      ...................G...........................G...................G..
1 2 Babyrousa babyrussa         .............A.....G............A..................................G..
2 2 Babyrousa babyrussa         ...................G...................................G...........G..
3 2 Babyrousa babyrussa         .............A.....G............A..................................G..
4 2 Babyrousa babyrussa         ...................G...................................G...........G..
1 24 Babyrousa babyrussa        .............A.....G...............................................G..
3 24 Babyrousa babyrussa        ...................G........................................A......GA.
4 24 Babyrousa babyrussa        ...................G.G........................G....................G..
5 24 Babyrousa babyrussa        ...................G..............................C................G..
6 24 Babyrousa babyrussa        ...................G......................T...................G....G..
7 24 Babyrousa babyrussa        ...................G..........A....................................G..
9 24 Babyrousa babyrussa        ...................G...............................................G..

                                         1200      1210      1220      1230      1240      1250      1260
                                ....|....|....|....|....|....|....|....|....|....|....|....|....|....|
Seq1 Ssrcofa8 chromosome1       ATCTACCAAAATCGCTTGGCCTTAGATTATTTACTTGCTTCTGAAGGA-GGGGTTTGTGGAAAATTTAAC
Seq2 Ssrcofa8 chromosome2       .......................G........................-.........A...........
Seq3 Ssrcofa8 chromosome2       ......A................G..................A..A..-.....G...............
Seq4 Ssrcofa8 chromosome6       .......................G........----............-.....................
Seq5 Ssrcofa8 chromosome7       .............A.........G.................C......-.....................
Seq6 Ssrcofa8 chromosome9       .........T...A.........G........................-.....................
Seq7 Ssrcofa8 chromosomeX       ......A.....T..........G......C...........A.....-.....................
Seq8 Ssrcofa8 chromosomeX       .......................G........................-.....................
Sus scrofa(AF356698)            G............A.........G........................-..........A...T......
3 Sus scrofa                    .............A.........G.................C......-.....................
7 Sus scrofa                    ................G......G........................-.A....G....G.........
1 Sus barbatus barbatus         .............A.........G.................C......-.....................
2 Sus barbatus barbatus         .....T......T.........AG........................-.....................
7 Sus barbatus oi               ......A.....T..........G......C..----...........-.........AA..........
5 Sus barbatus oi               ......A................G..................A..A..-.....G...............
10 Sus barbatus oi              ......A................G..................A.....-.....G...............
1 Sus verrucosus                .........T...A.........G........................-.....................
3 Sus verrucosus                ......A.....T..........G......C..----...........-.........AA..........
4 Sus verrucosus                ......A......A.........G....G.............A.....-.....................
10 Sus verrucosus               .........T...A.........G........................-.....................
1 Sus celebensis                .......................G........................-.....................
5 Sus celebensis                .......................G........C.........A.....-.....................
7 Sus celebensis                ......A................G..................A.....-.....G...............
8 Potamochoerus larvatus        G......................G........................-..........A.....-----
10 Potamochoerus larvatus       .......................G........................-...................G.
2 Potamochoerus porcus          ......A................G..................A.....-.....................
4 Potamochoerus porcus          .............A.........G........................-.........AA..........
6 Potamochoerus porcus          .......................G............C...........G................-----
1 Hylochoerus meinertzhageni    .......................G........................-.....................
```

```
4 Hylochoerus meinertzhageni  .......................G........................-.....................
5 Hylochoerus meinertzhageni  .................T.....G........................-..........A..........
3 Phacochoerus africanus      ............T..........G..................A.....-..........A..........
4 Phacochoerus africanus      .............A.........G........................-.........AA.......G..
5 Phacochoerus africanus      .............A.........G........................-.........AA.......G..
7 Phacochoerus africanus      .............A.........GC.......................-.....................
4 Phacochoerus aethiopicus    .......................G........................-.....................
6 Phacochoerus aethiopicus    .............T.........GC.......................-.....................
7 Phacochoerus aethiopicus    .........T...A.........G........................-.....................
1 2 Babyrousa babyrussa       .............A.........G........................-.....................
2 2 Babyrousa babyrussa       .......................G........................-.....................
3 2 Babyrousa babyrussa       .............A.........G........................-.....................
4 2 Babyrousa babyrussa       .......................G........................-.....................
1 24 Babyrousa babyrussa      .......................G........................-.....................
3 24 Babyrousa babyrussa      ......A.....T..........G......C...........A.....-.....................
4 24 Babyrousa babyrussa      ......A.....T......T...G......C.................-.T...................
5 24 Babyrousa babyrussa      ...C.........A.........G........................-.....................
6 24 Babyrousa babyrussa      .......................G........................-.....................
7 24 Babyrousa babyrussa      .....T.......A.........G........................-.........AA....-----
9 24 Babyrousa babyrussa      .......................G............A...........-.T..C................

                                      1270      1280      1290      1300      1310      1320      1330
                              ....|....|....|....|....|....|....|....|....|....|....|....|....|....|
Seq1 Ssrcofa8 chromosome1     TTAAGTAATTGCTGTCTACAAATAGATGACACTGGAAAGGTGGTAAAAAAAA------------------
Seq2 Ssrcofa8 chromosome2     ................................C............G..G...------------------
Seq3 Ssrcofa8 chromosome2     ................................C...............G...------------------
Seq4 Ssrcofa8 chromosome6     ................................C............G..G...------------------
Seq5 Ssrcofa8 chromosome7     ................................C...................----------AAAAAAAT
Seq6 Ssrcofa8 chromosome9     ......................-.A.....-.C............G..G...------------------
Seq7 Ssrcofa8 chromosomeX     ...C.........A..................C............G..G...------------------
Seq8 Ssrcofa8 chromosomeX     ................................C.A.................------------------
Sus scrofa(AF356698)          .............................................G..G...------------------
3 Sus scrofa                  ................................C...................AAAAAAAAAAAAAAAAAT
7 Sus scrofa                  ..G........T..............G..T............A..G..G...------------------
1 Sus barbatus barbatus       ................................C...................----------AAAAAAAT
2 Sus barbatus barbatus       ................................C............G..G..G-----------------A
7 Sus barbatus oi             ....A....C.......T.........A.T....A.................----------------AA
5 Sus barbatus oi             ................................CA..............G...------------------
10 Sus barbatus oi            ................................C...............G...------------------
1 Sus verrucosus              ................................CA...........G..G...------------------
3 Sus verrucosus              .................T.........A......A.................-----------------A
4 Sus verrucosus              ................................C............G..G...------------------
10 Sus verrucosus             ......................-.A.....-.C............G..G...------------------
1 Sus celebensis              .....................................C.......G..G...------------------
5 Sus celebensis              ..................G..........................G..G...------------------
7 Sus celebensis              .......................G........CA..............G...------------------
```

```
8 Potamochoerus larvatus      ..................................A..........G......------------------
10 Potamochoerus larvatus     ..........A..A..................C............G..G...------------------
2 Potamochoerus porcus        ................................C............G..G...------------------
4 Potamochoerus porcus        ..................................A..........G..G...------------------
6 Potamochoerus porcus        ..................................A..........G......------------------
1 Hylochoerus meinertzhageni  ................................C............G..G...------------------
4 Hylochoerus meinertzhageni  ................................C............G..G...------------------
5 Hylochoerus meinertzhageni  .............................................G......------------------
3 Phacochoerus africanus      ....A...........................CAT..........G......------------------
4 Phacochoerus africanus      ..................................A..........G..G...------------------
5 Phacochoerus africanus      ..................................A..........G..G...------------------
7 Phacochoerus africanus      ..G.............................CA...........G..G...------------------
4 Phacochoerus aethiopicus    ........................A....................G..G...------------------
6 Phacochoerus aethiopicus    ................................CA...........G..G...------------------
7 Phacochoerus aethiopicus    ................................CA...........G..G...------------------
1 2 Babyrousa babyrussa       .............................................G..G...------------------
2 2 Babyrousa babyrussa       ................C.........................A..G..G...------------------
3 2 Babyrousa babyrussa       .............................................G..G...------------------
4 2 Babyrousa babyrussa       ................C.........................A..G..G...------------------
1 24 Babyrousa babyrussa      ...G..............................A.............G...------------------
3 24 Babyrousa babyrussa      ......................G...........A..........G..G...------------------
4 24 Babyrousa babyrussa      .............................................G......------------------
5 24 Babyrousa babyrussa      .........................G........A..........G..G...------------------
6 24 Babyrousa babyrussa      ................................C............G......-----------------A
7 24 Babyrousa babyrussa      ...........................A......A.................------------------
9 24 Babyrousa babyrussa      ....................G........................G..G...------------------

                                     1340      1350      1360      1370      1380      1390      1400
                              ....|....|....|....|....|....|....|....|....|....|....|....|....|....|
Seq1 Ssrcofa8 chromosome1     TTGCTGACCACATGACTAAACTAGCCCATGTA-CCTGTACAAACTTGGAAAGGATAGGATGCAGACAGTT
Seq2 Ssrcofa8 chromosome2     .........T......................-......................G.A..A........C
Seq3 Ssrcofa8 chromosome2     ........TG......................-..............A.......G.............C
Seq4 Ssrcofa8 chromosome6     .........G......................-......................G.............C
Seq5 Ssrcofa8 chromosome7     .........G...A........T......A..-......................G....A........C
Seq6 Ssrcofa8 chromosome9     ........TG............T.........-.........G............G.............C
Seq7 Ssrcofa8 chromosomeX     .........T............T........G-....C...TC...........CG.............C
Seq8 Ssrcofa8 chromosomeX     .........G...A........T.........-..............A.......G....AT......CC
Sus scrofa(AF356698)          ............A.........T.........-......................G....A........C
3 Sus scrofa                  .........G...A........T......A..-......................G....A........C
7 Sus scrofa                  .........G............T.....C..G-.................G....G---------....C
1 Sus barbatus barbatus       .............A........T......A..-...........................A........C
2 Sus barbatus barbatus       .........G......................-......................G.............C
7 Sus barbatus oi             ........T.............T......A.C-....C.................G....A........C
5 Sus barbatus oi             ........TG......................-......................G.............C
10 Sus barbatus oi            ........TG......................-..............A.......G.............C
1 Sus verrucosus              ........TG............T.........-.........G............G.............C
```

```
3 Sus verrucosus                ........T.............T......A.C-....C.................G....A........C
4 Sus verrucosus                ....C...TG.G..........T.........-......................G....A........C
10 Sus verrucosus               ........TG............TT........-......................G.............C
1 Sus celebensis                .........G.................G....-....................................C
5 Sus celebensis                .........G......................-......................G....A........C
7 Sus celebensis                ........TG......................-..............A.......G.............C
8 Potamochoerus larvatus        .........G............T.........-......................G....AT.......C
10 Potamochoerus larvatus       .........G......................-......................G.............C
2 Potamochoerus porcus          .........G......................-......................G....A....G...C
4 Potamochoerus porcus          ........TG............T........G-......................G.............C
6 Potamochoerus porcus          .........G............T.........-......................G....AT.......C
1 Hylochoerus meinertzhageni    .........G......................-......................G..........T..C
4 Hylochoerus meinertzhageni    .........G......................-......................G..........T..C
5 Hylochoerus meinertzhageni    ........TG............T.........-......................G....AT.......C
3 Phacochoerus africanus        ...............................G-....C.................G.............C
4 Phacochoerus africanus        ................................-......................G.............C
5 Phacochoerus africanus        ................................-......................G.............C
7 Phacochoerus africanus        .........G......................-......................G..........C..C
4 Phacochoerus aethiopicus      ................................-....C.................G.............C
6 Phacochoerus aethiopicus      ........TG.........G.CT.........-......................G.............C
7 Phacochoerus aethiopicus      ........TG.........G.CT.........-......................G.............C
1 2 Babyrousa babyrussa         ........TG............T.........-......................G.............C
2 2 Babyrousa babyrussa         .........TT...........T........G-.....G................G.............C
3 2 Babyrousa babyrussa         ........TG............T.........-......................G.............C
4 2 Babyrousa babyrussa         .........TT...........T........G-.....G................G.............C
1 24 Babyrousa babyrussa        .........G.......C....T......A.G-....C.................G....A......A..
3 24 Babyrousa babyrussa        ........TG..........T.T........G-....C.................G....A........C
4 24 Babyrousa babyrussa        ................................-......................G.............C
5 24 Babyrousa babyrussa        .........G......................-......................G.............C
6 24 Babyrousa babyrussa        .........G......................-....A.................G......G.----.C
7 24 Babyrousa babyrussa        .........GA...........T........GC...A..................G....A........C
9 24 Babyrousa babyrussa        .........G............T.........-......................G...........A.C

                                         1410      1420      1430      1440
                                ....|....|....|....|....|....|....|....|....
Seq1 Ssrcofa8 chromosome1       TCTTTGGTAAATGGTTCTCTTGGTTGGGAAGAATTAAGACAATA
Seq2 Ssrcofa8 chromosome2       ......................A......G.............G
Seq3 Ssrcofa8 chromosome2       .............................G.............G
Seq4 Ssrcofa8 chromosome6       .............................G.............G
Seq5 Ssrcofa8 chromosome7       ......................C......GA............G
Seq6 Ssrcofa8 chromosome9       .............................G.............G
Seq7 Ssrcofa8 chromosomeX       ................T.............A............G
Seq8 Ssrcofa8 chromosomeX       .............................GA............G
Sus scrofa(AF356698)            .............................GA............G
3 Sus scrofa                    ......................C......GA............G
7 Sus scrofa                    ..........G..................G.............G
```

1 *Sus barbatus barbatus*	......................C......GA............G
2 *Sus barbatus barbatus*	.............................G.............G
7 *Sus barbatus oi*	.............................GA............G
5 *Sus barbatus oi*	.............................G.............G
10 *Sus barbatus oi*	.............................G.............G
1 *Sus verrucosus*	..........................A..G.....C.......G
3 *Sus verrucosus*	.............................GA............G
4 *Sus verrucosus*	..........G..................G.............G
10 *Sus verrucosus*	.............................G.............G
1 *Sus celebensis*	....
5 *Sus celebensis*	............A................GA............G
7 *Sus celebensis*	.............................G.............G
8 *Potamochoerus larvatus*	.....................A.......G.............G
10 *Potamochoerus larvatus*	.............................G.............C
2 *Potamochoerus porcus*	..................T..........G.............G
4 *Potamochoerus porcus*	.............................G............C.
6 *Potamochoerus porcus*	.....................A.......G.............G
1 *Hylochoerus meinertzhageni*	..............C..............G.............G
4 *Hylochoerus meinertzhageni*	..............C..............G.............G
5 *Hylochoerus meinertzhageni*	.....................A.......G.............G
3 *Phacochoerus africanus*	.............................G.............G
4 *Phacochoerus africanus*	.............................G..............
5 *Phacochoerus africanus*	.............................G..............
7 *Phacochoerus africanus*	....................CA.......G.............G
4 *Phacochoerus aethiopicus*	.................................G.........G
6 *Phacochoerus aethiopicus*	.............................G.............G
7 *Phacochoerus aethiopicus*	....................-........G.............G
1 2 *Babyrousa babyrussa*	.............................G.............G
2 2 *Babyrousa babyrussa*	............................GG.............G
3 2 *Babyrousa babyrussa*	.............................G.............G
4 2 *Babyrousa babyrussa*	............................GG.............G
1 24 *Babyrousa babyrussa*	..............................A............G
3 24 *Babyrousa babyrussa*	..............................A............G
4 24 *Babyrousa babyrussa*	.............................G...-.......G.G
5 24 *Babyrousa babyrussa*	..---------------............G.............G
6 24 *Babyrousa babyrussa*	...........C.................G.............G
7 24 *Babyrousa babyrussa*	..---------------............G.............G
9 24 *Babyrousa babyrussa*	...........CA.................A............G
